# Mechanochemical Piezoelectric Catalysis: Advancing the Hydrogenation of Alkenes

**DOI:** 10.1002/advs.76446

**Published:** 2026-07-06

**Authors:** Zixi Ai, Mengting Liu, Xiaohong Wang, Xiaochun He, Xuemei Zhang, Zhong Lian

**Affiliations:** ^1^ State Key Laboratory of Biotherapy and Cancer Center West China Hospital Sichuan University Chengdu China

**Keywords:** alkenes, hydrogenation, mechanochemistry, piezoelectric catalysis

## Abstract

Alkene hydrogenation reactions are essential processes widely utilized across diverse scientific fields for the production of pharmaceuticals, natural products, and various functional materials. Current strategies (e.g., metal hydrogen atom transfer [mHAT], photo/electrochemistry) consistently necessitate compromises, invariably involving transition metals, pressurized H_2_, or solvent‐intensive conditions. Herein, we report a mechanochemical hydrogenation reaction of both activated and unactivated alkenes via piezoelectric catalysis under conditions that are free from transition‐metals, H_2_ gas, and solvents. This approach exhibits good compatibility with various functional groups and demonstrates a broad substrate scope. In addition, the hydrogenation of bioactive molecule derivatives for modification purposes highlights its potential applications in the pharmaceutical industry. Based on mechanistic investigations, we propose a potential mechanism for the alkene hydrogenation via piezoelectric catalysis.

## Graphical abstract

This study presents mechanochemical piezoelectric catalysis for alkene hydrogenation, eliminating the need for transition metals, H_2_, and solvents. The method effectively hydrogenates diverse activated and unactivated alkenes and shows potential for modifying bioactive molecules in pharmaceuticals. Mechanistic investigations further clarify a novel reaction pathway.

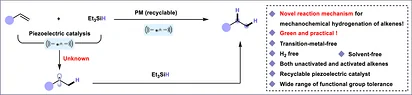

The Nobel Prize‐winning process of alkene hydrogenation [1] not only plays a crucial role in academic research but also serves as a cornerstone of industrial production [2, 3]. Despite advances in conventional methodologies, most systems employs a combination of expensive transition‐metal catalysts and hydrogen gas (Figure 1ai) [4, 5], which raises safety concerns due to the flammable and explosive nature of hydrogen [6]. Notable advances have been achieved in alkene radical hydrogenation via metal hydrogen atom transfer (mHAT, Figure 1aii) [7, 8]. In addition, transition‐metal hydrides can also be produced through electron‐initiated photochemical and electrochemical methods [9, 10, 11, 12]. These strategies, while replacing H_2_ with hydride donors, still depend on transition‐metal catalysts or stoichiometric strong oxidants, limiting functional group tolerance.

**FIGURE 1:**
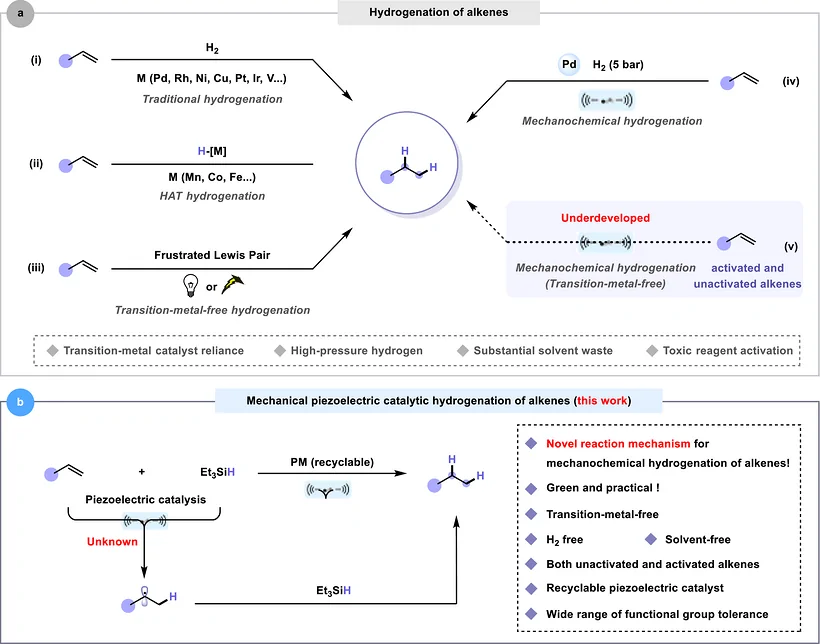
(a) Hydrogenation of alkenes; (b) This work: Mechanical piezoelectric catalytic hydrogenation of alkenes.

The Stephan group's development of frustrated Lewis pairs (FLPs), have paved the way toward metal‐free catalytic hydrogenation (Figure 1aiii) [13, 14], yet this method still involves handling flammable H_2_ and requires activation by toxic reagents such as chlorosilanes, boranes, or selenium [15]. Recent advancements in non‐metal mediated alkene reduction have incorporated photochemical and electrochemical strategies (Figure 1aiii) [16, 17, 18, 19, 20, 21, 22, 23]. For example, the Guo group identified a thioxanthone‐TfOH complex as a photoredox catalyst exclusively for the hydrogenation of *α*,*β*‐unsaturated alkenes [20]. Qiu et al. enabled the electrochemical hydrogenation or deuteration of alkenes using water or heavy water as the hydrogen/deuterium source [21, 22]. Although various hydrogen sources, such as hydrazine, water, and Hantzsch ester, have been utilized in these systems, such methods are generally performed in solvent media, generating substantial waste. Furthermore, unactivated alkenes, with highly negative reduction potentials (*E*_red_ < −3.0 V vs. SCE), remain particularly challenging substrates for direct reduction. These limitations motivate the development of a transition‐metal‐free, H_2_‐free, and solvent‐free hydrogenation strategy applicable to both activated and unactivated alkenes.

Mechanoredox catalysis has recently emerged as a solvent‐minimal platform for synthetic chemistry, significantly reducing solvent waste while offering an environmentally benign framework [23, 24, 25, 26, 27, 28, 29, 30, 31, 32, 33, 34, 35, 36, 37, 38, 39, 40, 41, 42, 43, 44]. This approach occasionally enables reaction pathways inaccessible in traditional solvent‐based environments [45, 46, 47, 48, 49, 50]. In the context of mechanochemical alkene hydrogenation, Borchardt et al. demonstrated the efficient hydrogenation of alkenes using specially manufactured palladium‐coated milling vessels with H_2_ gas, achieving high conversion rates under mild conditions (Figure 1aiv) [51]. Inspired by the cost‐effectiveness, availability, and low toxicity of Et_3_SiH as a hydrogen source [52, 53], we propose a transition metal‐free protocol employing commercially available piezoelectric material barium titanate (BaTiO_3_) as a catalyst, and Et_3_SiH as the hydrogen donor for alkene hydrogenation within ordinary stainless‐steel milling jars. As illustrated in Figure 1b, our design introduces a novel hydrogenation pathway in which mechanical milling enhances the interaction between the alkene and Et_3_SiH via piezoelectric catalysis, facilitating carbon‐centered radical formation through single‐electron transfer. This radical subsequently reacts with another Et_3_SiH molecule to afford the hydrogenated product. This work establishes a transition‐metal‐free, H_2_‐free, and solvent‐free hydrogenation of both activated and unactivated alkenes via mechanoredox catalysis, facilitated by a recyclable piezoelectric catalyst.

In the initial phase of our research, we employed 4‐vinylbiphenyl **1a** as the model reactant to investigate reaction conditions for alkene hydrogenation via mechanochemical piezoelectric catalysis. A variety of piezoelectric materials were utilized as catalysts, including BaTiO_3_ (1–3 µm), BaTiO_3_ (< 1 µm), SrTiO_3_, PdTiO_3_, ZnO, and LiNbO_3_ (Figure 2a). Among these, BaTiO_3_ (1–3 µm) was identified as the most effective catalyst. In contrast, only trace product formation was observed in the absence of BaTiO_3_ (Table S3), confirming that the piezoelectric catalyst is essential for this transformation. Subsequently, we assessed various silanes, such as Et_3_SiH, *n*Bu_3_SiH, PhSiMe_2_H, *t*BuSiMe_2_H, and PhSiH_3_ (Figure 2b). The results indicated that triethylsilane was the most efficient hydrogen source, while the other silanes produced the target product with varying yields, ranging from 37% to 78%. We further explored the influence of different additives on the transformation. The additives tested included KO*t*Bu, NaOMe, NaO*t*Bu, CsF, KOH, and Li_3_PO_4_ (Figure 2c). Among these, KO*t*Bu emerged as the most favorable additive for facilitating the reaction. In summary, under mechanical grinding conditions, we successfully obtained the hydrogenation product **3a** with an 80% yield, using BaTiO_3_ (1–3 µm) as the piezoelectric catalyst, 2.0 equivalents of Et_3_SiH as the hydrogen source, and 30 mmol% KO*t*Bu as the additive. To further elucidate the role of piezoelectric materials in this reaction, we conducted additional experiments using non‐piezoelectric materials or inorganic salts, such as NaCl, MgSO_4_, Na_2_SO_4_, and SiO_2_, in place of piezoelectric BaTiO_3_. None of these substitutions produced the desired product (**3a**), highlighting the essential role of piezoelectric materials in the mechanochemical hydrogenation reaction (Figure 2d). In addition, stirring the same reactants in various solvents, including ethanol, acetonitrile, and tetrahydrofuran (THF) at 60°C for 24 h, resulted in only minimal quantities of the hydrogenated product **3a** (Figure 2e). Moreover, no target product **3a** was detected when dichloroethane (DCM) or dimethylformamide (DMF) was utilized as a solvent. These findings underscore the crucial role of mechanical force in promoting the hydrogenation process.

**FIGURE 2:**
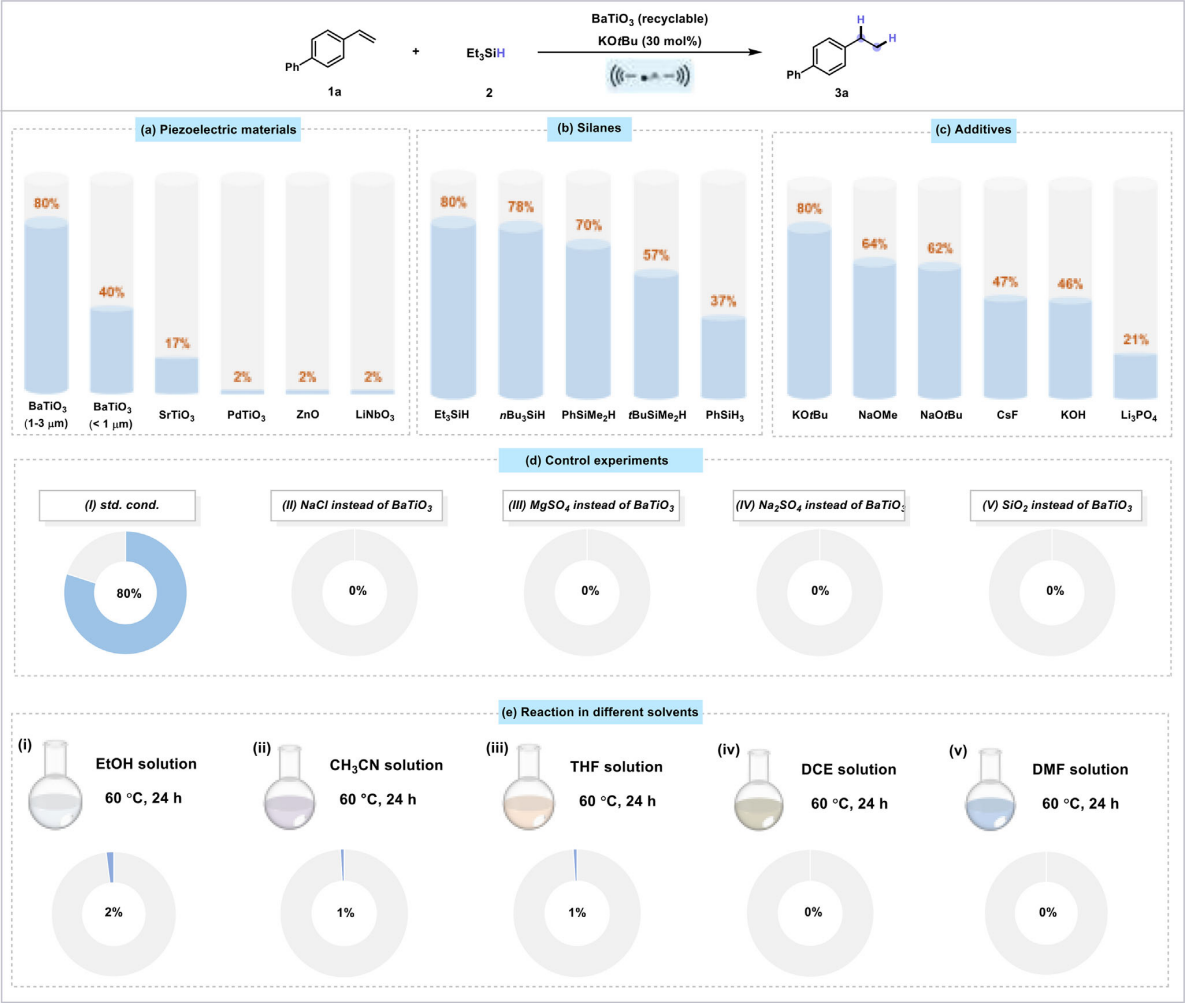
Optimization of reaction conditions.

With the optimized reaction conditions established, we proceeded to investigate the compatibility of this mechanochemical hydrogenation with various styrenes bearing different functional groups (Scheme 1). A styrene derivative containing an electron‐donating ─NMe_2_ group on the phenyl ring successfully yielded the corresponding hydrogenated product **3b**. In addition, 2‐vinylnaphthalene and 9‐vinylanthracene, which lack substituents on the phenyl ring, underwent hydrogenation with high efficiencies (**3c** and **3d**). *α*‐substituted styrenes, such as *α*‐arylstyrene (**3e** and **3f**), *α*‐cyclohexylstyrene (**3** **g**), *α*‐ethylstyrene (**3** **h**), and *α*‐methylstyrene (**3i**), were all compatible with the reaction. Moreover, *α*‐arylpyridine (**3j**) and *α*‐methylvinylnaphthalene (**3k**) successfully yielded the target hydrogenated products. Subsequently, we examined the reactivity of internal olefins, particularly focusing on *β*‐substituted styrene derivatives. Our results indicated that *β*‐alkyl substituted styrene efficiently generated the desired product with satisfactory yields (**3l**). Notably, internal alkenes, including both *trans*‐ and *cis*‐stilbene, were also successfully converted, affording the corresponding hydrogenated products in 82% and 80% yield, respectively (**3m**). Increasing the milling frequency and reaction time significantly enhanced the transformation efficiency for various polysubstituted alkenes, including *tri*‐ (**3n‐3p**) and even *tetra*‐(**3q**) substituted alkenes.

**SCHEME 1:**
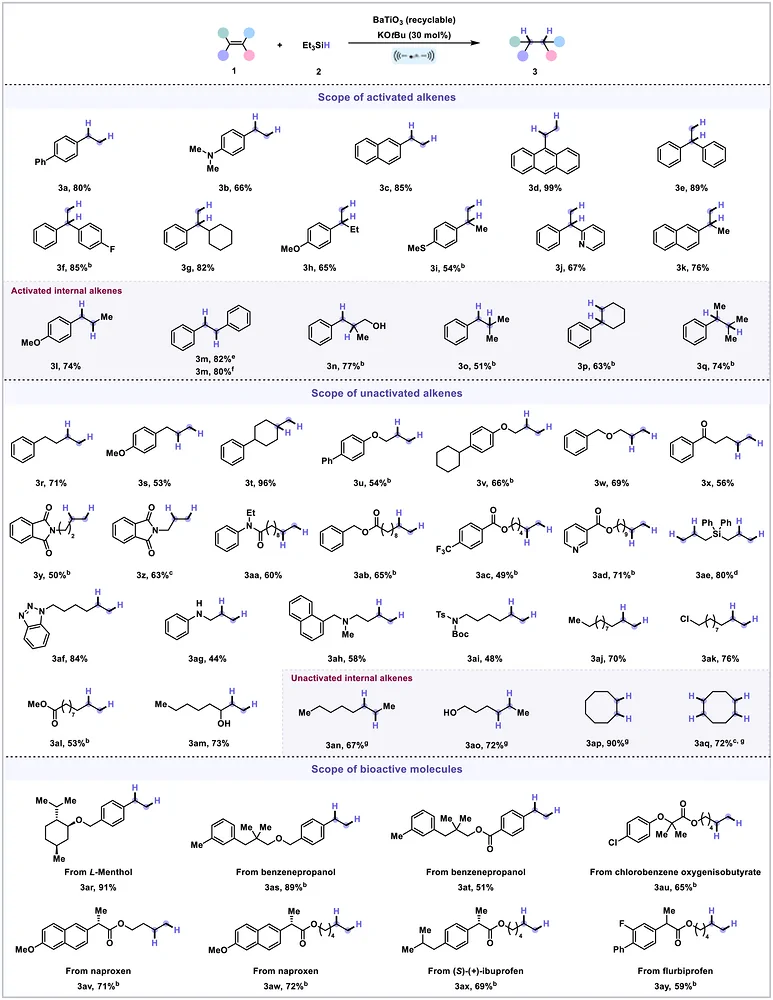
Reaction condition: (a) BaTiO_3_ (1.5 mmol, 5.0 equiv.), KO*t*Bu (0.09 mmol, 0.3 equiv.), alkenes (**1**, 0.3 mmol, 1.0 equiv.), and triethylsilane (**2**, 0.6 mmol, 2.0 equiv.) in a stainless‐steel milling jar (10.0 mL) with two stainless‐steel balls (10 mm, diameter) in air; ball milling for 2 h at 30 Hz; (b) ball milling for 3 h at 35 Hz. (c) Triethylsilane (**2**, 1.2 mmol, 4.0 equiv.) and ball milling for 2 h at 30 Hz. (d) Triethylsilane (**2**, 1.2 mmol, 4.0 equiv.) and ball milling for 2 h at 35 Hz. (e) *Trans*‐stilbene (0.3 mmol, 1.0 equiv.). (f) *cis*‐Stilbene (0.3 mmol, 1.0 equiv.). (g) The yield was determined by GC analysis using *n*‐dodecane as an internal standard.

Furthermore, we shifted our attention to the reactivity of unactivated alkenes in the hydrogenation reaction. A variety of unactivated alkenes featuring diverse functionalities were subjected to mechanical milling conditions. Pleasingly, we observed that 4‐phenylbutene (**3r**), 4‐methoxy substituted allylbenzene (**3s**), methylenecyclohexane derivative (**3t**), and terminal alkenes comprising ether functionality (**3u**‐**3w**) were all tolerated, yielding their corresponding hydrogenation products. Importantly, our mechanochemical strategy demonstrated compatibility with a range of typical functional groups, including ketone (**3x**), phthalimide (**3y** and **3z**), amide (**3aa**), ester (**3ab**‐**3ad**), trifluoromethyl (**3ac**), pyridine (**3ad**), silane (**3ae**), benzotriazole (**3af**), secondary and tertiary amines (**3ag** and **3ah**), and sulfonamide (**3ai**). In addition, terminal alkenes with longer chains (**3aj**), along with those containing functional groups such as chlorine (**3ak**), ester (**3al**), and free hydroxyl (**3am**), produced target products with yields ranging from moderate to good. We further investigated the reactivity of unactivated internal alkenes in this reaction. Results showed that both linear (**3an** and **3ao**) and cyclic (**3ap** and **3aq**) internal alkenes effectively generated the target compounds. These outcomes collectively demonstrate that our mechanochemical hydrogenation strategy exhibits remarkable tolerance toward both activated and unactivated alkenes, as well as a wide array of functional groups, highlighting its significant potential for practical applications.

In light of the significant role of alkene hydrogenation in pharmaceutical science, we showcased the practicality of our mechanochemical hydrogenation strategy for several bioactive molecules. Styrenes derived from pharmaceuticals, including *L*‐menthol (**3ar**) and benzenepropanol (**3as** and **3at**), were successfully transformed into their corresponding hydrogenated products with moderate to excellent yields. Furthermore, unactivated alkenes sourced from chlorobenzene oxygenisobutyrate (**3au**), naproxen (**3av** and **3aw**), (*S*)‐(+)‐ibuprofen (**3ax**), and flurbiprofen (**3ay**) were also converted into the desired products with moderate to good yields, while preserving the integrity of the ester functionality within these molecular frameworks. The outcomes of these experiments pave the way for promising avenues in drug modification.

To illustrate the synthetic utility of our mechanochemical hydrogenation method, we conducted gram‐scale amplification experiments. The hydrogenation of compound **1a** was performed on 2 and 5 mmol scales, resulting in the target product (**3a**) with yields of 77% and 68%, respectively (Figure 3a). In addition, we conducted piezoelectric catalyst recycling experiments (Figure 3b). BaTiO_3_ could be easily filtered, washed, and dried from the reaction mixture after each hydrogenation reaction. The recycled BaTiO_3_ powder was successfully reused in more catalytic cycles, although a gradual decrease in the catalytic activity of BaTiO_3_ was observed. The results indicated a gradual decrease in the catalytic activity of BaTiO_3_, which can be attributed to a gradual reduction in BaTiO_3_ particle size that negatively impacted its piezoelectric properties [54, 55, 56]. The particle size of piezoelectric materials can be increased via sintering and conventional solid‐state processing techniques, facilitating their recycling [57, 58].

**FIGURE 3:**
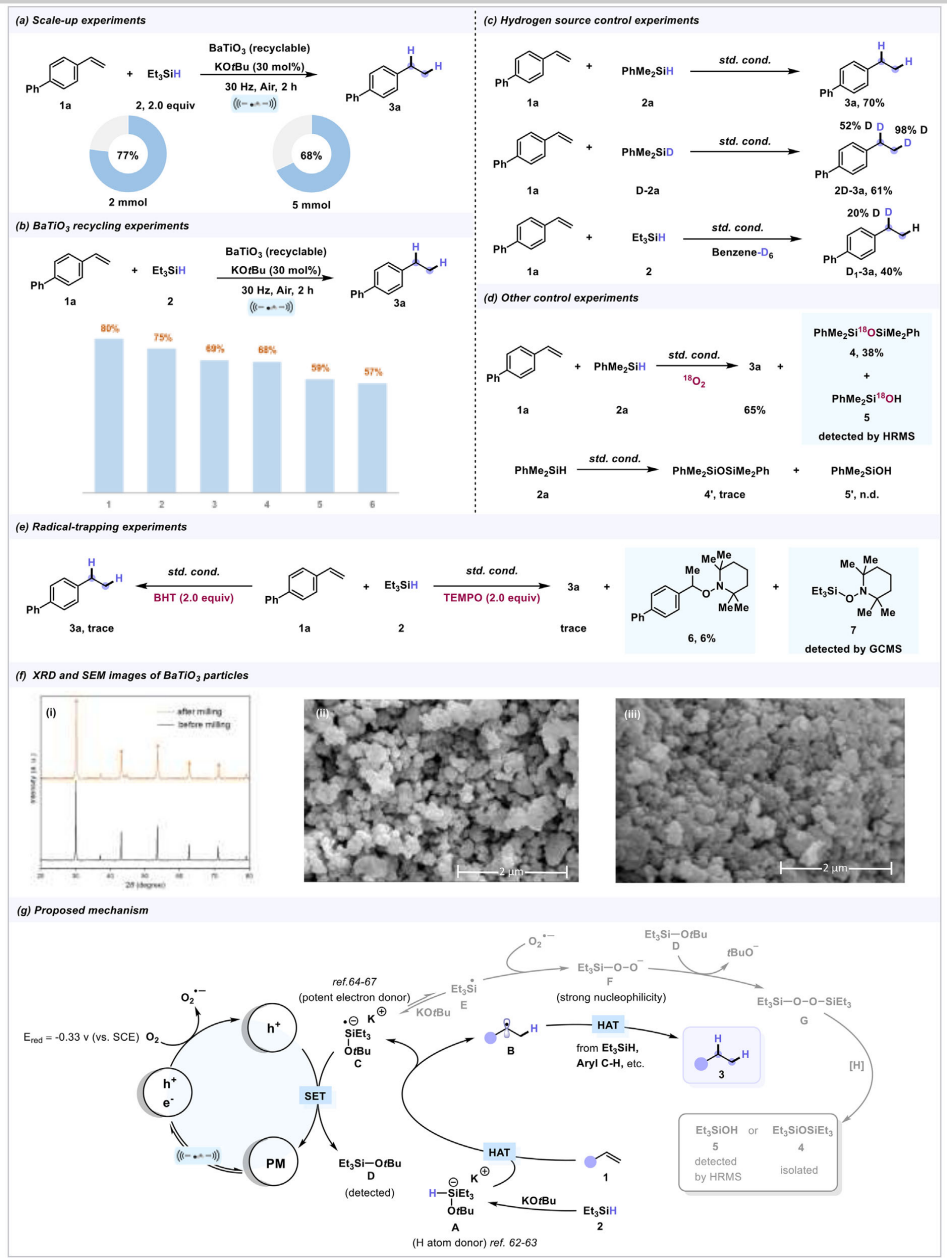
(a) Scale‐up experiments, standard conditions: BaTiO_3_ (1.5 mmol, 5.0 equiv.), KO*t*Bu (0.09 mmol, 0.3 equiv.), alkenes (**1a**, 0.3 mmol, 1.0 equiv.), and triethylsilane (**2**, 0.6 mmol, 2.0 equiv.) in air; ball milling for 2 h at 30 Hz; (b) BaTiO_3_ recycling experiments; (c) Hydrogen source control experiments; (d) Other control experiments; (e) Radical‐trapping experiments; (f) XRD (i) and SEM images of BaTiO_3_ particles, (ii) before reaction; (iii) after reaction; (g) proposed mechanism.

Furthermore, we carried out a series of experiments with alkene **1a** to elucidate the intricacies of the reaction mechanism. First, the use of deuterated silane PhMe_2_SiD in place of PhMe_2_SiH in the reaction led to the desired product **2D‐3a** in a 61% yield, achieving 98% deuterium incorporation at the terminal methyl group and 52% at the methylene position. Employing a different deuterated silane Ph(CD_3_)_2_SiH in the reaction resulted in a deuterated product with no deuterium incorporation at the terminal methyl group, but 10% at the methylene position (see Figure S11). Subsequently, carrying out the model reaction with benzene‐D_6_ also resulted in deuterium incorporation (20%) on the benzylic position of product **D_1_‐3a**, demonstrating that a hydrogen atom from the aromatic ring could also participate in the H‐atom transfer (Figure 3c). These findings suggest that silane serves as the main hydrogen source in the hydrogenation reaction. Under standard experimental conditions, when the milling jar was filled with ^18^O_2_ gas instead of air, compound **4** (Si─^18^O─Si) was obtained in 38% isolated yield, along with the ^18^O‐labeled silanol (**5**), which was detected by high‐resolution mass spectrometry (HRMS). This observation indicates that oxygen in the air facilitates the hydrogenation process. In contrast, a control experiment conducted in the absence of alkene **1a** yielded only trace amounts of **4′** and **5′** was not observed. Thus, these products are confirmed to be specific byproducts generated subsequent to the alkene hydrogenation, ruling out their formation via a background process (Figure 3d). Further investigations included conducting reactions in the presence of radical scavengers (Figure 3e). The introduction of butylated hydroxytoluene (BHT) and 2,2,6,6‐tetramethyl‐1‐piperidinyloxy (TEMPO) completely inhibited the hydrogenation process. The detection of the TEMPO and benzyl radical adduct **6** (isolated in 6% yield), coupled with the GC–MS observation of the TEMPO‐Et_3_Si• adduct (**7**), supports the hypothesis that the reaction proceeds through a radical pathway. Our subsequent analysis of the piezoelectric catalyst, which is the commercially sourced BaTiO_3_ powder, involved high‐resolution x‐ray diffraction (XRD) before and after the mechanical grinding process. The XRD analysis confirmed that the tetragonal phase structure of the BaTiO_3_ particles was preserved after ball milling (Figure 3fi). Further examination using scanning electron microscopy (SEM) revealed significant morphological deformation of the BaTiO_3_ particles. Notably, the average particle size reduced following the hydrogenation reaction due to the mechanical pressure applied during grinding at a frequency of 30 Hz (Figure 3fii, iii).

Based on the above mechanistic experiments, a plausible mechanism for the mechanochemical hydrogenation of alkenes has been proposed (Figure 3g). Mechanical agitation induces polarization in BaTiO_3_ particles, which promotes the single‐electron reduction of oxygen (*E*_red_ = −0.33 V vs. SCE) [59] to generate the superoxide radical anion (O_2_^•−^) [60, 61]. Concurrently, the Et_3_SiH/KO*t*Bu system forms a pentacoordinate silicate (**A**), which donates a hydrogen atom to the alkene substrate (**1**) via hydrogen atom transfer (HAT). This step yields a key benzylic radical intermediate (**B**) along with a silicon radical anion (**C**) [62, 63]. Subsequently, benzylic radical (**B**) could abstract a hydrogen atom from the silane or an aryl C─H bond (e.g., from the starting material, etc.) via an S_H_2 pathway, directly producing product **3**. Meanwhile, the potent electron donor **C** [64, 65, 66, 67] is capable of donating an electron to the hole on BaTiO_3_ (*E*_red_ ≈ −1.9 V vs. SCE), regenerating the BaTiO_3_ catalyst and generating a silyl ether (**D**). The dissociation of *t*BuO^−^ from the silicon radical anion **C** would liberate a silyl radical (**E**) [63], which then combines with the previously superoxide radical anion to form the Si─O─O^−^ intermediate (**F**). Subsequently, the Si─O─O^−^ intermediate (**F**) participates in nucleophilic attack on the silyl ether (**D**), leading to the formation of the peroxysilyl ether (**G**), and the release of *t*BuO^−^. Ultimately, **G** undergoes reduction to produce the observed byproducts: silyl ether (**4**), which was isolated, and silanol (**5**), detected by HRMS.

In summary, we present a groundbreaking advancement in the field of mechanochemical catalysis, showcasing an efficient and streamlined approach to piezoelectric‐catalyzed hydrogenation of both activated and unactivated alkenes. This innovative methodology operates under the remarkable conditions of being free from transition metals, hydrogen gas, and solvents, marking a significant departure from conventional hydrogenation practices. Our findings reveal an extensive substrate compatibility, adeptly accommodating a diverse array of functional groups—including esters, ketones, amides, sulfonamides, silyl groups, and hydroxy functionalities—that typically pose challenges under traditional hydrogenation protocols. Furthermore, in‐depth mechanistic investigations underscore the critical involvement of silane and piezoelectric materials in facilitating the hydrogenation process, elucidating their pivotal roles in enhancing reaction efficiency. This work not only highlights the potential of mechanochemistry in organic synthesis but also paves the way for future explorations of eco‐friendly, metal‐free catalytic methods that can transform the landscape of organic chemistry.

## Author Contributions

**Zixi Ai**: methodology, validation, formal analysis, conceptualization. **Mengting Liu**: methodology, formal analysis. **Xiaohong Wang**: methodology, formal analysis. **Xiaochun He**: conceptualization, investigation, formal analysis, writing – original draft. **Xuemei Zhang**: writing – review and editing, writing – original draft, conceptualization, investigation. **Zhong Lian**: conceptualization, writing – review and editing, project administration.

## Conflicts of Interest

The authors declare no conflicts of interest.

## Supporting information




**Supporting File**: advs76446‐sup‐0001‐SuppMat.pdf.

## Data Availability

The data that supports the findings of this study are available in the Supporting Information of this article.
