# Supplementary material for: Mechanochemical Piezoelectric Catalysis: Advancing the Hydrogenation of Alkenes
Source: Adv Sci (Weinh). 2026 Jul 6;13(54):e76446. doi: 10.1002/advs.76446 (PMC13335898; doi:10.1002/advs.76446)
Supplement: Supplementary file 1 [file ADVS-13-e76446-s001.pdf]

# Supporting Information

## Mechanochemical Piezoelectric Catalysis: Advancing the Hydrogenation of Alkenes

Zixi Ai[1†], Mengting Liu[1†], Xiaohong Wang[1†], Xiaochun He[1*], Xuemei Zhang[1*] & Zhong Lian[1*]

[1]State Key Laboratory of Biotherapy and Cancer Center, West China Hospital, Sichuan University; Chengdu 610041, China

[†]These authors contributed equally to this work and ranked in order of the authors' last names.

*Corresponding author. Email: lianzhong@scu.edu.cn; xuemeizhang@scu.edu.cn; hxc@scu.edu.cn

## Table of Contents

1. General information of instrumentation and chemicals ........ S2
2. General procedure for the synthesis of alkenes ........ S3
3. Optimization of reaction conditions ........ S3
4. Control experiments in different solvents ........ S6
5. General procedure for the hydrogenation of alkenes ........ S6
6. Characterization data of hydrogenation products ........ S7
7. Procedure for the scale-up reaction ........ S17
8. $BaTiO_3$ recycling experiments ........ S17
9. $^{18}O_2$ Labeled trapping experiment ........ S18
10. Hydrogen source control experiments ........ S19
11. Radical-trapping experiments ........ S21
12. Characterization of $BaTiO_3$ particles by SEM ........ S23
13. XRD patterns of $BaTiO_3$ ........ S23
14. References ........ S24
15. NMR spectra ........ S25

## 1. General information of instrumentation and chemicals

Materials were obtained from commercial suppliers and purified by standard procedures unless otherwise noted. *tet*-$BaTiO_3$ (1-3 µm particle size, 99%, product No. A66127), *tet*-$BaTiO_3$ (<1 µm particle size, 99%, product No. A66124), $PbTiO_3$ (product No. A60705), $SrTiO_3$ (0.5-5 µm particle size, 99%, product No. E061762) and $LiNbO_3$ (product No. A60320), ZnO (<1 µm particle size, 99%, product No. A64210) were purchased from Energy Chemical. All reactions were performed using grinding vessels in a Gladman vibration ball mill GT300 or GT600. Both jars and balls were made of stainless steel. Solvents for reactions were purchased from commercial suppliers. $^{1}H$, $^{19}F$, $^{13}C$ NMR spectra were recorded in $CDCl_3$ on Bruker Avance 400 MHz spectrometers. Multiplicity was recorded as follows: s = singlet, brs = broad singlet, d = doublet, t = triplet, q = quartet, m = multiplet. Dodecane was used as an internal standard to determine GC yields. Recycle preparative gel permeation chromatography (GPC) was conducted with a LC-5060P (Serial No. LA5-21345) using ethyl acetate as an eluent. High-resolution mass data were recorded on a high-resolution mass spectrometer in the EI/Q-TOF mode or ESI/Q-TOF mode. The molecular ion $[M]^+$, $[M+H]^+$, $[M-H_2O]^+$, $[M+NH_4]^+$, $[M+Na]^+$ are given in m/z units. Thin Layer Chromatography analysis was performed on silica gel coated glass plates (0.25 mm) with fluorescence indicator UV254. For detection of spots, irradiation of UV light at 254 nm or staining reagent using basic potassium permanganate solution was used. Flash column chromatography was conducted with silica gel (particle size 300-400 mesh, Huanghai) at room temperature and under elevated pressure.

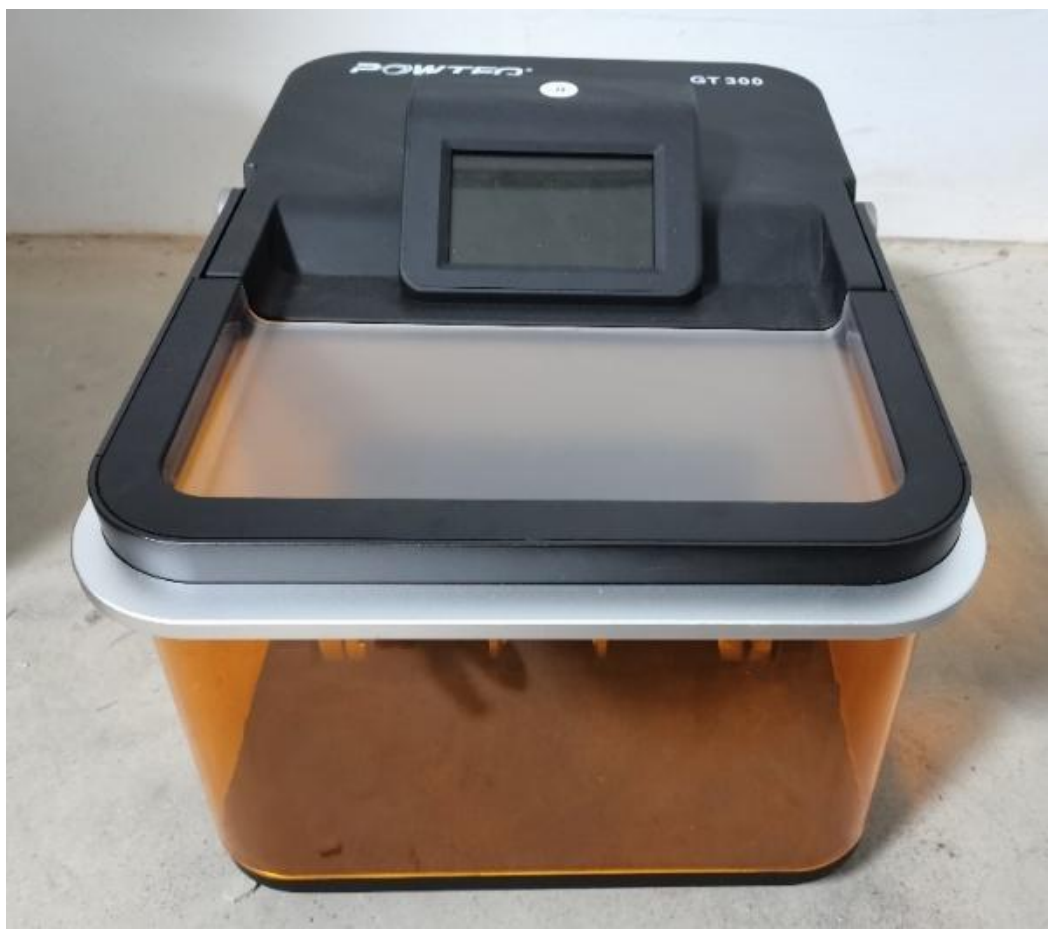

**Figure S1**. Gladman vibration ball mill GT300 used in this study.

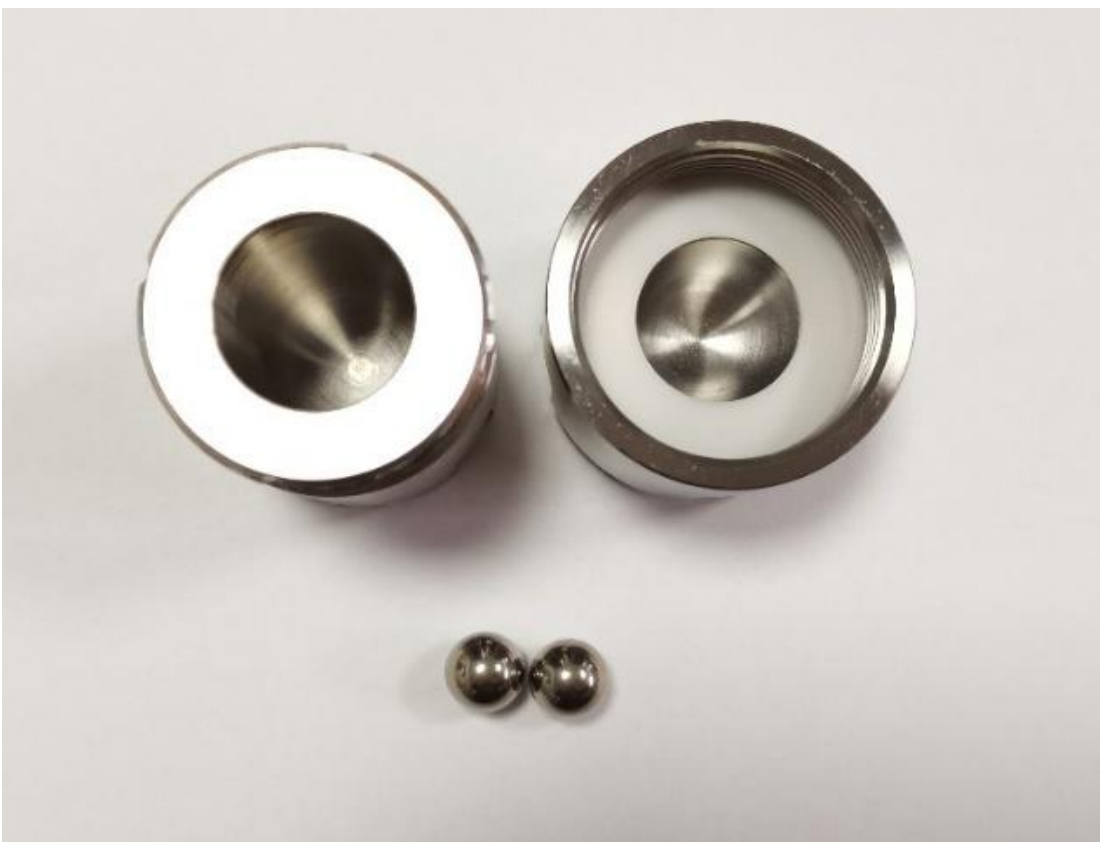

**Figure S2**. 10 mL stainless jars and 10 mm balls used in this study.

## 2. General procedure for the synthesis of alkenes

The following substrates were prepared according to the literature. Other alkenes were purchased from Energy Chemical, Bidepharm or Tansoole without further purification.

A list of the alkenes utilized in this study is presented in Figures S1 and S2. **1f-1j**,[1] **1p**,[2] **1s**,[1] **1t**,[3] **1u**,[3] **1w**,[4] **1x**,[3] **ly**,[3] **1z**,[3] **1aa**,[5] **1ab**,[5] **1ac**,[5] **1ae**,[3] **1ag**,[6] **1ah**,[5] **1aq**,[5] **1ar**,[7] **1as-1ax**[8] were synthesized according to the corresponding literature.

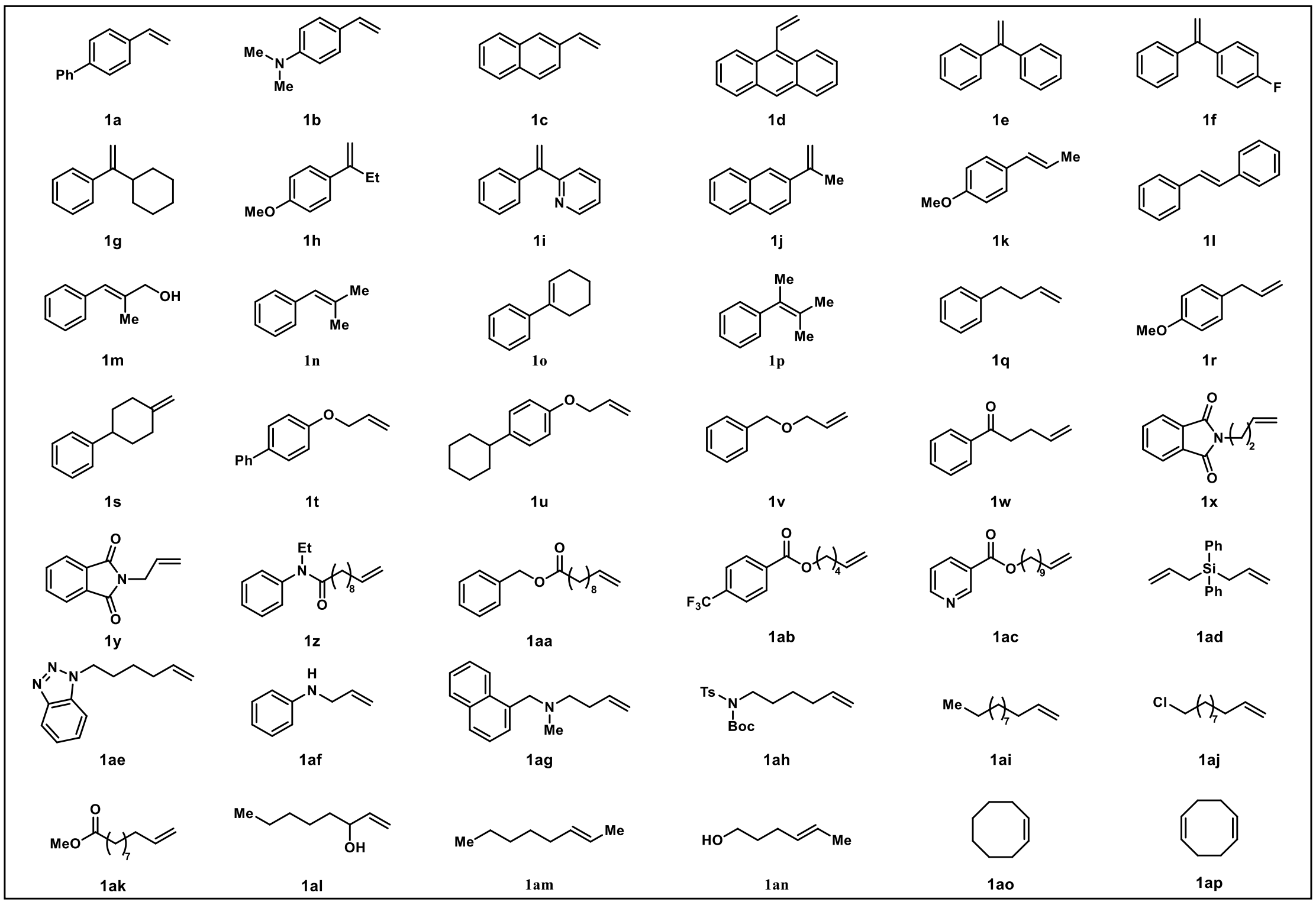


**Figure S3**. List of alkenes used in this study

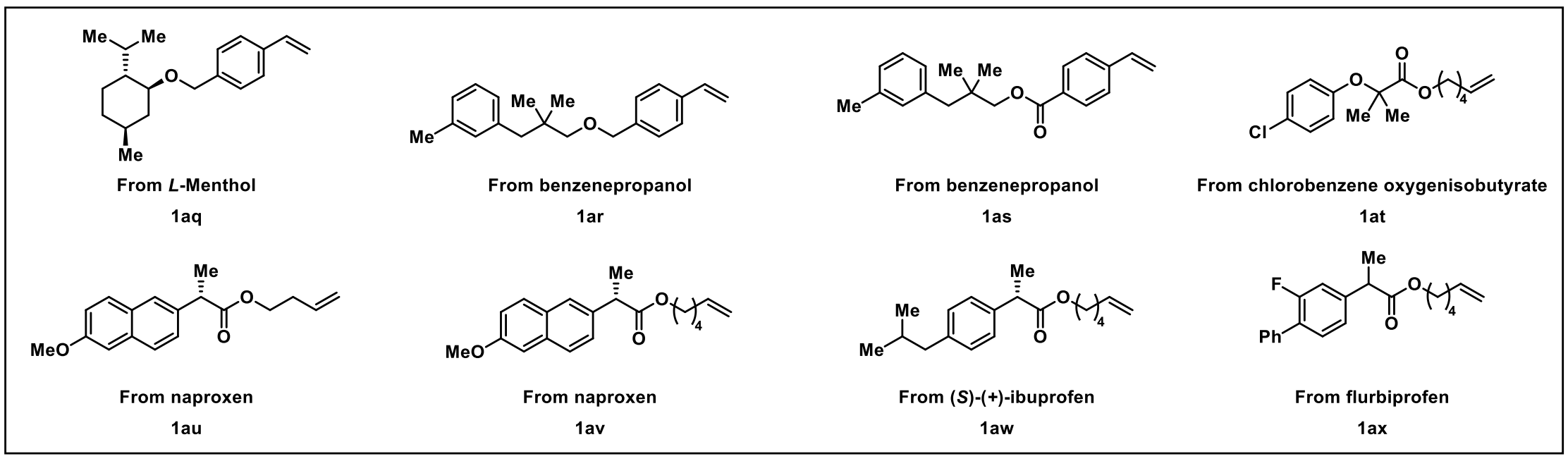


**Figure S4**. List of bioactive molecules used in this study

## 3. Optimization of reaction conditions

Piezoelectric materials (1.5 mmol, 5.0 equiv), additives (0.09 mmol, 0.3 equiv), and *p*-vinylbiphenyl (**1a**, 0.3 mmol, 1.0 equiv) were placed in a 10.0 mL stainless-steel milling jar, along with two stainless-steel balls (10 mm diameter). After adding triethylsilane (**2**, 0.6 mmol, 2.0 equiv) to the solid mixture, the jar was sealed and subjected to milling at 30 Hz under air. After grinding for 2 h, the reaction mixture was washed with ethyl acetate, filtered. The yield of hydrogenation product **3a** was determined by GC analysis using dodecane as an internal standard.

**Table S1. Optimization of reaction conditions.**

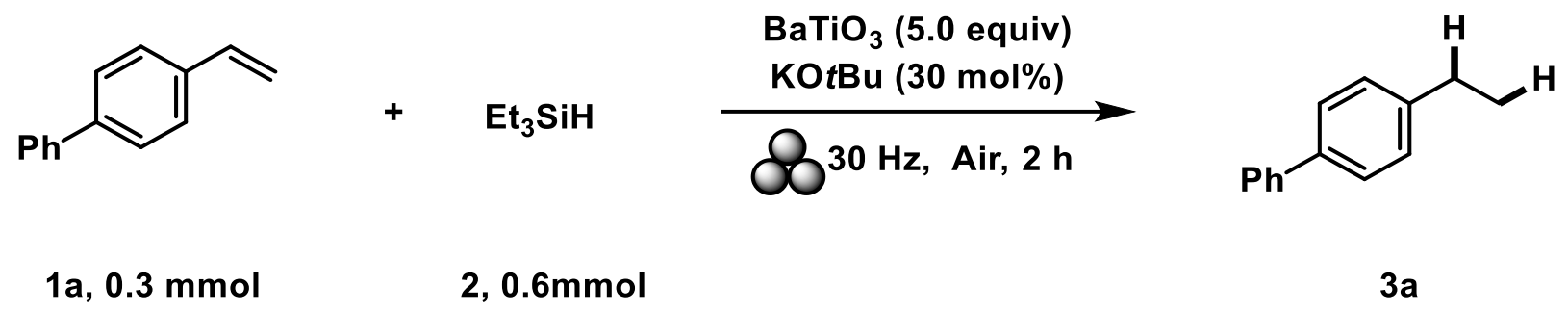


| Entry | Deviation from standard conditions | Yield of **3a** (%) |
|---|---|---|
| 1 | none | 80 |
| 2 | $BaTiO_3$ (<1 μm) instead of $BaTiO_3$ (1-3 μm) | 40 |
| 3 | $SrTiO_3$ instead of $BaTiO_3$ | 17 |
| 4 | $PbTiO_3$ instead of $BaTiO_3$ | 2 |
| 5 | ZnO instead of $BaTiO_3$ | 2 |
| 6 | $LiNbO_3$ instead of $BaTiO_3$ | 2 |
| 7 | NaCl instead of $BaTiO_3$ | 0 |
| 8 | $MgSO_4$ instead of $BaTiO_3$ | 0 |
| 9 | $Na_2SO_4$ instead of $BaTiO_3$ | 0 |
| 10 | $SiO_2$ instead of $BaTiO_3$ | 0 |
| 11 | *n*$Bu_3SiH$ instead of $Et_3SiH$ | 78 |
| 12 | $PhSiMe_2H$ instead of $Et_3SiH$ | 70 |
| 13 | *t*$BuSiMe_2H$ instead of $Et_3SiH$ | 57 |
| 14 | $PhSiH_3$ instead of $Et_3SiH$ | 37 |
| 15 | NaOMe instead of KO*t*Bu | 64 |
| 16 | NaO*t*Bu instead of KO*t*Bu | 62 |
| 17 | CsF instead of KO*t*Bu | 47 |
| 18 | KOH instead of KO*t*Bu | 46 |
| 19 | $Li_3PO_4$ instead of KO*t*Bu | 21 |
| 20 | Ar instead of air | trace |

**Table S2. Optimization of the frequency**

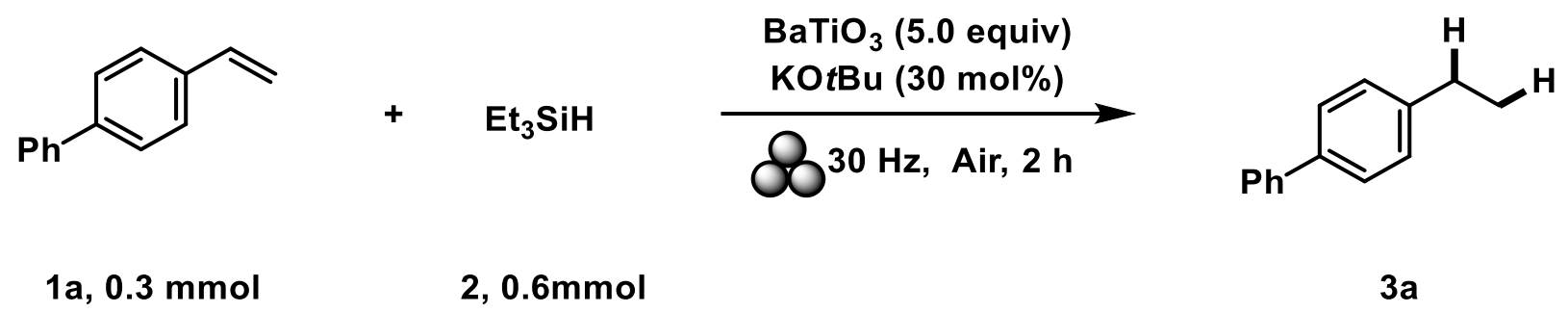


| Entry | Frequency (Hz) | Yield of **3a** (%) |
|---|---|---|
| 1 | 10 Hz | n.d. |
| 2 | 15 Hz | 3 |
| 3 | 20 Hz | 4 |
| 4 | 25 Hz | 33 |
| 5 | 30 Hz | 80 |

Reaction condition: $BaTiO_3$ (1.5 mmol, 5.0 equiv), KO*t*Bu (0.09 mmol, 0.3 equiv), **1a** (0.3 mmol, 1.0 equiv) and triethylsilane (**2**, 0.6 mmol, 2.0 equiv) in a stainless-steel milling jar (10.0 mL) with two stainless-steel balls (10 mm, diameter) in air; ball milling for 2 h at x Hz, the yield was determined by GC analysis.

**Table S3. Optimization of the amount of $BaTiO_3$**

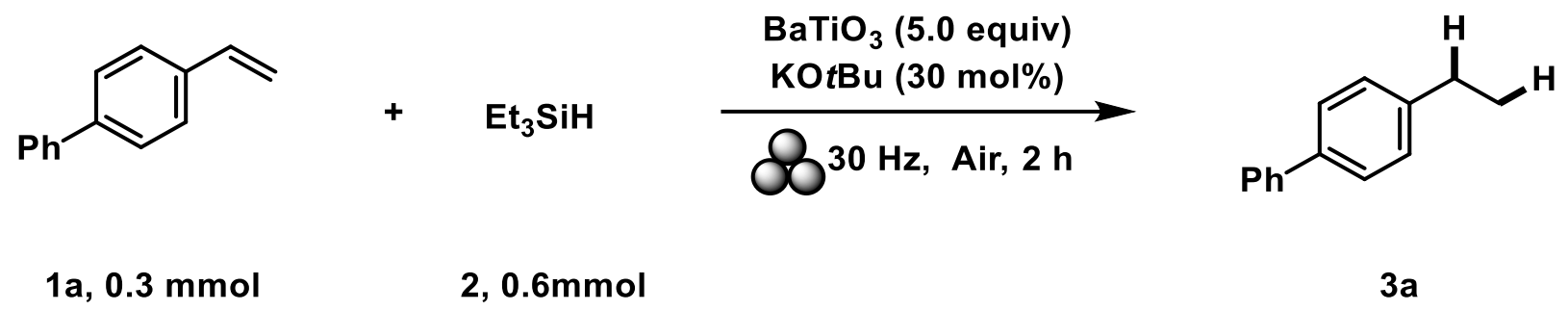


| Entry | $BaTiO_3$ (equiv) | Yield of **3a** (%) |
|---|---|---|
| 1 | without $BaTiO_3$ | trace |
| 2 | 3.0 | 43 |
| 3 | 4.0 | 57 |
| 4 | 5.0 | 80 |
| 5 | 6.0 | 67 |

Reaction condition: $BaTiO_3$ (x equiv), KO*t*Bu (0.09 mmol, 0.3 equiv), **1a** (0.3 mmol, 1.0 equiv) and triethylsilane (**2**, 0.6 mmol, 2.0 equiv) in a stainless-steel milling jar (10.0 mL) with two stainless-steel balls (10 mm, diameter) in air; ball milling for 2 h at 30 Hz, the yield was determined by GC analysis using *n*-dodecane an internal standard.

**Table S4. Optimization of the amount of KO*t*Bu**

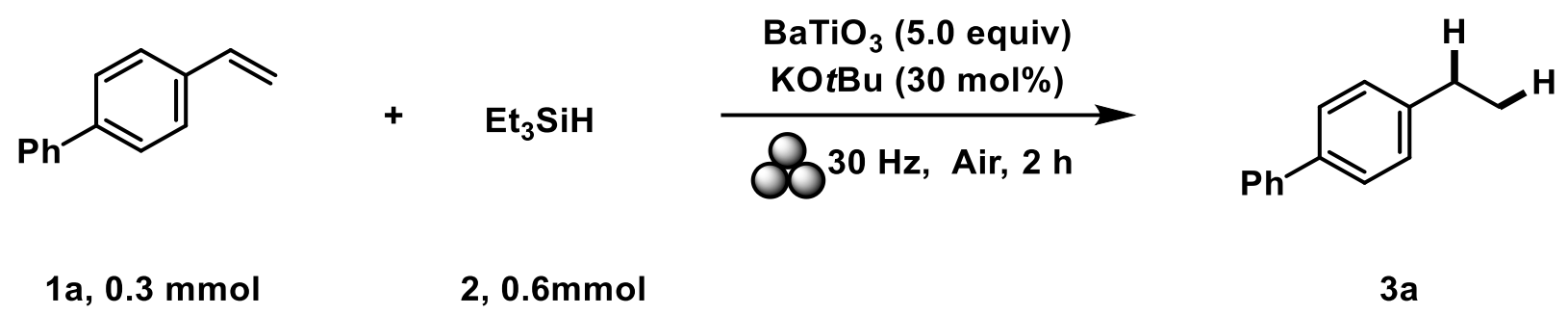


| Entry | KO*t*Bu (mol%) | Yield of **3a** (%) |
|---|---|---|
| 1 | 20 | 59 |
| 2 | 25 | 75 |
| 3 | 30 | 80 |
| 4 | 35 | 63 |

Reaction condition: $BaTiO_3$ (1.5 mmol, 5.0 equiv), KO*t*Bu (x mol%), **1a** (0.3 mmol, 1.0 equiv) and triethylsilane (**2**, 0.6 mmol, 2.0 equiv) in a stainless-steel milling jar (10.0 mL) with two stainless-steel balls (10 mm, diameter) in air; ball milling for 2 h at 30 Hz, the yield was determined by GC analysis using *n*-dodecane an internal standard.

**Table S5. Optimization of the amount of $Et_3SiH$**

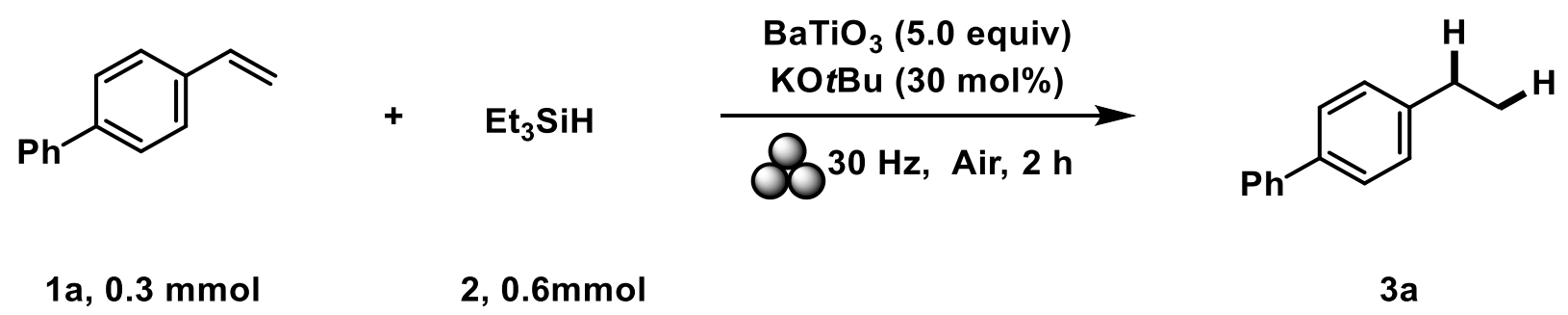


| Entry | $Et_3SiH$ (equiv) | Yield of **3a** (%) |
|---|---|---|
| 1 | 1.5 | 30 |
| 2 | 2.0 | 80 |
| 3 | 2.5 | 65 |

Reaction condition: $BaTiO_3$ (1.5 mmol, 5.0 equiv), KO*t*Bu (0.09 mmol, 0.3 equiv), **1a** (0.3 mmol, 1.0 equiv) and triethylsilane (**2**, x equiv) in a stainless-steel milling jar (10.0 mL) with two stainless-steel balls (10 mm, diameter) in air; ball milling for 2 h at 30 Hz, the yield was determined by GC analysis using *n*-dodecane an internal standard.

**Table S6. Optimization of H Source**

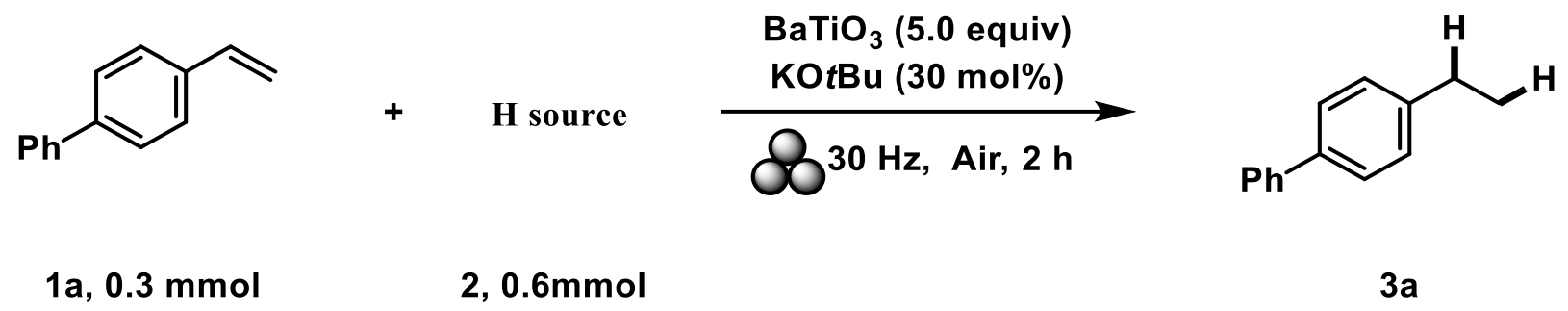


| Entry | H Source | Yield of **3a** (%) |
|---|---|---|
| 1 | $H_2O$ | n.d. |
| 2 | HCOOH | n.d. |
| 3 | $CH_3COOH$ | n.d. |
| 4 | EtOH | 11 |
| 5 | IPA | 9 |

Reaction condition: $BaTiO_3$ (1.5 mmol, 5.0 equiv), KO*t*Bu (0.09 mmol, 0.3 equiv), **1a** (0.3 mmol, 1.0 equiv) and H Source (0.6 mmol, 2.0 equiv) in a stainless-steel milling jar (10.0 mL) with two stainless-steel balls (10 mm, diameter) in air; ball milling for 2 h at 30 Hz, the yield was determined by GC analysis using *n*-dodecane an internal standard.

## 4. Control experiments in different solvents

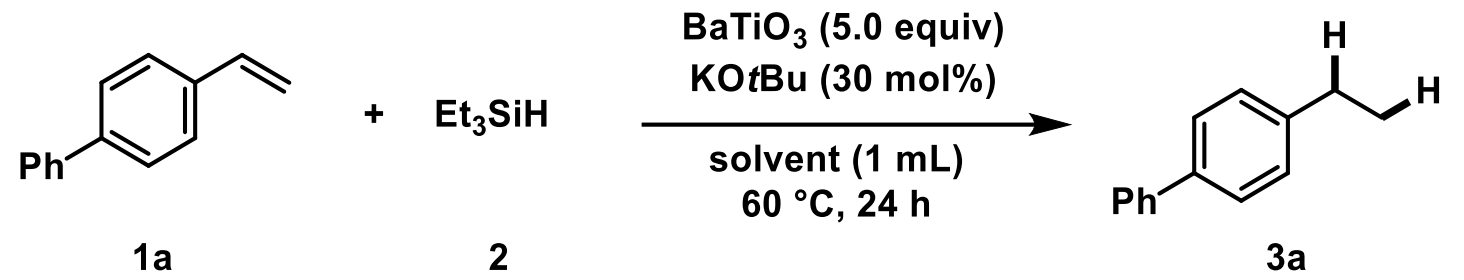


| Entry | Solvent | Yield of **3a** (%) |
|---|---|---|
| 1 | EtOH | 2 |
| 2 | $CH_3CN$ | 1 |
| 3 | THF | 1 |
| 4 | DCE | 0 |
| 5 | DMF | 0 |

$BaTiO_3$ (350 mg, 1.5 mmol), KO*t*Bu (10 mg, 0.09 mmol), *p*-vinylbiphenyl (**1a**, 54 mg, 0.3 mmol), triethylsilane (**2**, 95 μL, 0.6 mmol) and ethanol (1 mL) were placed in a vial. Stir the reactant at 60 °C for 24 hours. The yield of hydrogenation product **3a** was determined by GC analysis using dodecane as an internal standard.

## 5. General procedure for the hydrogenation of alkenes

**General Procedure A**: $BaTiO_3$ (1.5 mmol, 5.0 equiv), KO*t*Bu (0.09 mmol, 0.3 equiv), and various alkenes (**1**, 0.3 mmol, 1.0 equiv) were placed in a 10.0 mL stainless-steel milling jar, along with two stainless-steel balls (10 mm diameter). After adding triethylsilane (**2**, 0.6 mmol, 2.0 equiv) to the solid mixture, the jar was sealed and subjected to milling at 30 Hz under air. After grinding for 2 h, the reaction mixture was washed with ethyl acetate, filtered, and concentrated *in vacuo*. The crude product was purified by column chromatography on silica gel to furnish the desired products.

**General Procedure B**: $BaTiO_3$ (1.5 mmol, 5.0 equiv), KO*t*Bu (0.09 mmol, 0.3 equiv), and various alkenes (**1**, 0.3 mmol, 1.0 equiv) were placed in a 10.0 mL stainless-steel milling jar, along with two stainless-steel balls (10 mm diameter). After adding triethylsilane (**2**, 0.6 mmol, 2.0 equiv) to the solid mixture, the jar was sealed and subjected to milling at 35 Hz under air. After grinding for 3 h, the reaction mixture was washed with ethyl acetate, filtered, and concentrated *in vacuo*. The crude

product was purified by column chromatography on silica gel to furnish the desired products.

## 6. Characterization data of hydrogenation products

**4-ethyl-1,1'-biphenyl (3a)**

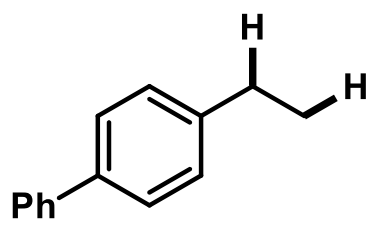


Compound **3a** was prepared following the general procedure A, using corresponding alkene. The crude residue was purified by silica gel column chromatography (Petroleum ether) to afford the desired product (44 mg, 80%) as a colorless oil. $^{1}$H NMR (400 MHz, Chloroform-*d*) δ 7.64 – 7.61 (m, 2H), 7.56 (d, *J* = 8.0 Hz, 2H), 7.46 (t, *J* = 7.6 Hz, 2H), 7.37 (d, *J* = 7.2 Hz, 1H), 7.31 (d, *J* = 8.0 Hz, 2H), 2.74 (q, *J* = 7.6 Hz, 2H), 1.34 – 1.30 (m, 3H). $^{13}$C NMR (101 MHz, Chloroform-*d*) δ 143.5, 141.3, 138.7, 128.8, 128.4, 127.2, 127.19, 127.12, 28.6, 15.7. HR-MS (EI): m/z calcd for $[M]^+$ $C_{14}H_{14}^+$, 182.1096; found 182.1091.

**4-ethyl-*N*,*N*-dimethylaniline (3b)**

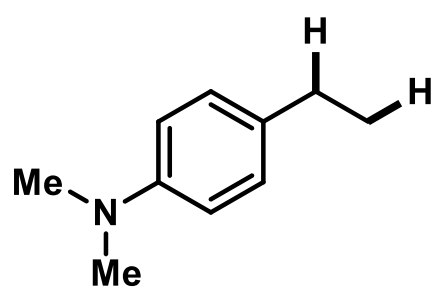


Compound **3b** was prepared following the general procedure A, using corresponding alkene. The crude residue was purified by silica gel column chromatography (Hexane / ethyl acetate = 20/1) to afford the desired product (30 mg, 66%) as a yellow oil. $^{1}$H NMR (400 MHz, Chloroform-*d*) δ 7.14 – 7.09 (m, 2H), 6.77 – 6.71 (m, 2H), 2.93 (s, 6H), 2.59 (q, *J* = 7.6 Hz, 2H), 1.23 (t, *J* = 7.6 Hz, 3H). $^{13}$C NMR (101 MHz, Chloroform-*d*) δ 149.1, 132.8, 128.5, 113.3, 41.1, 27.9, 16.0. HR-MS (ESI): m/z calcd for $[M+H]^+$ $C_{10}H_{16}N^+$, 150.1277; found 150.1275.

**2-ethylnaphthalene (3c)**

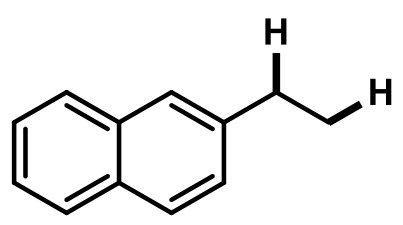


Compound **3c** was prepared following the general procedure A, using corresponding alkene. The crude residue was purified by silica gel column chromatography (Hexane) to afford the desired product (40 mg, 85%) as a colorless oil. $^{1}$H NMR (400 MHz, Chloroform-*d*) δ 7.86 – 7.78 (m, 3H), 7.66 (s, 1H), 7.51 – 7.42 (m, 2H), 7.39 (dd, *J* = 8.4, 1.8 Hz, 1H), 2.85 (q, *J* = 7.6 Hz, 2H), 1.37 (t, *J* = 7.6 Hz, 3H). $^{13}$C NMR (101 MHz, Chloroform-*d*) δ 141.9, 133.8, 132.0, 127.9, 127.7, 127.5, 127.2, 125.9, 125.7, 125.1, 29.2, 15.7. HR-MS (EI): m/z calcd for $[M]^+$ $C_{12}H_{12}^+$, 156.0939; found 156.0930.

**9-ethylanthracene (3d)**

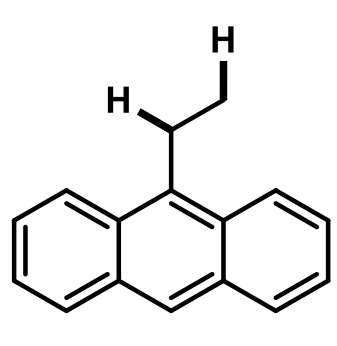


Compound **3d** was prepared following the general procedure A, using corresponding alkene. The crude residue was purified by silica gel column chromatography (Hexane) to afford the desired product (61 mg, 99%) as a yellow solid. $^{1}$H NMR (400 MHz, Chloroform-*d*) δ 8.38 – 8.29 (m, 3H), 8.08 – 8.00 (m, 2H), 7.57 – 7.47 (m, 4H), 3.69 (q, *J* = 7.6 Hz, 2H), 1.49 (t, *J* = 7.6 Hz, 3H). $^{13}$C NMR (101 MHz, Chloroform-*d*) δ 136.8, 131.8, 129.3, 125.6, 125.5, 124.9, 124.4, 21.2, 15.6. HR-MS (EI): m/z calcd for $[M]^+$ $C_{16}H_{14}^+$, 206.1096; found 206.1092.

**1,1-diphenylethane (3e)**

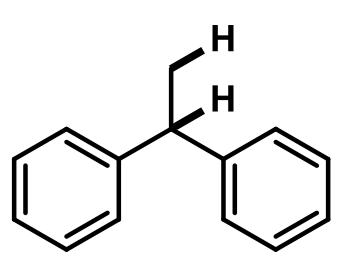


Compound **3e** was prepared following the general procedure A, using corresponding alkene. The crude residue was purified by silica gel column chromatography (Petroleum ether / ethyl acetate = 50/1) to afford the desired product (49 mg, 89%) as a colorless oil. $^{1}$H NMR (400 MHz, Chloroform-*d*) δ 7.23 (d, *J* = 7.4 Hz, 4H), 7.20 – 7.17 (m, 4H), 7.16 – 7.12 (m, 2H), 4.11 (t, *J* = 7.0 Hz, 1H), 1.59 (t, *J* = 7.0 Hz, 3H). $^{13}$C NMR (101 MHz, Chloroform-*d*) δ 146.5, 128.5, 127.7, 126.1, 44.9, 22.0. HR-MS

(EI): m/z calcd for $[M]^+$ $C_{14}H_{14}^+$, 182.1096; found 182.1090.

**1-fluoro-4-(1-phenylethyl)benzene (3f)**

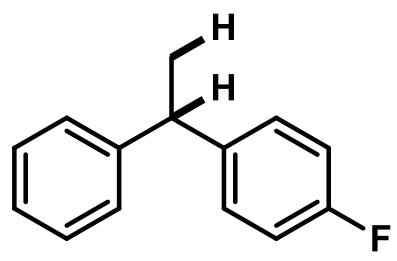

Compound **3f** was prepared following the general procedure B, using corresponding alkene. The crude residue was purified by silica gel column chromatography (Hexane) to afford the desired product (51 mg, 85%) as a colorless oil. $^1$H NMR (400 MHz, Chloroform-*d*) δ 7.31 (t, *J* = 7.6 Hz, 2H), 7.23-7.18 (m, 5H), 7.01-6.94 (m, 2H), 4.16 (q, *J* = 7.4 Hz, 1H), 1.65 (dd, *J* = 7.2, 1.2 Hz, 3H). $^{19}$F NMR (376 MHz, Chloroform-*d*) δ -117.41. $^{13}$C NMR (101 MHz, Chloroform-*d*) δ 162.4 (d, *J* = 245.4 Hz), 146.3, 142.2 (d, *J* = 3.0 Hz), 129.1 (d, *J* = 8.1 Hz), 128.6, 127.6, 126.3, 115.2 (d, *J* = 21.2 Hz), 44.1, 22.1. HR-MS (EI): m/z calcd for $[M]^+$ $C_{14}H_{13}F^+$, 200.1001; found 200.0999.

**(1-cyclohexylethyl)benzene (3g)**

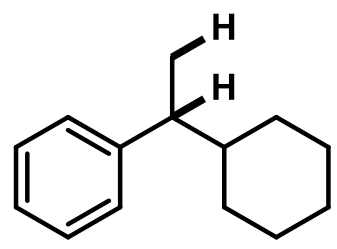

Compound **3g** was prepared following the general procedure A, using corresponding alkene. The crude residue was purified by silica gel column chromatography (Hexane) to afford the desired product (46 mg, 82%) as a colorless oil. $^1$H NMR (400 MHz, Chloroform-*d*) δ 7.23 (d, *J* = 7.6 Hz, 2H), 7.16 – 7.09 (m, 3H), 2.41 (p, *J* = 7.2 Hz, 1H), 1.85 (d, *J* = 12.8 Hz, 1H), 1.71 (d, *J* = 13.0 Hz, 1H), 1.58 (d, *J* = 9.8 Hz, 2H), 1.42 – 1.32 (m, 2H), 1.20 (d, *J* = 6.8 Hz, 4H), 1.11 – 1.03 (m, 2H), 0.96 – 0.71 (m, 2H). $^{13}$C NMR (101 MHz, Chloroform-*d*) δ 147.2, 128.1, 127.8, 125.7, 46.0, 44.3, 31.6, 30.7, 26.65, 26.61, 18.9. HR-MS (EI): m/z calcd for $[M]^+$ $C_{14}H_{20}{}^+$, 188.1565; found 188.1558.

**1-(sec-butyl)-4-methoxybenzene (3h)**

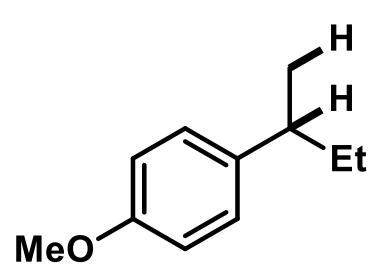

Compound **3h** was prepared following the general procedure A, using corresponding alkene. The crude residue was purified by silica gel column chromatography (Hexane) to afford the desired product (32 mg, 65%) as a colorless oil. $^1$H NMR (400 MHz, Chloroform-*d*) δ 7.05 – 7.00 (m, 2H), 6.79 – 6.74 (m, 2H), 3.71 (s, 3H), 2.47 (q, *J* = 7.0 Hz, 1H), 1.48 (q, *J* = 7.2 Hz, 2H), 1.13 (d, *J* = 6.8 Hz, 3H), 0.74 (t, *J* = 7.4 Hz, 3H). $^{13}$C NMR (101 MHz, Chloroform-*d*) δ 157.7, 139.9, 128.0, 113.7, 55.3, 40.9, 31.4, 22.1, 12.3. HR-MS (EI): m/z calcd for $[M]^+$ $C_{11}H_{16}O^+$, 164.1201; found 164.1197.

**(4-isopropylphenyl)(methyl)sulfane (3i)**

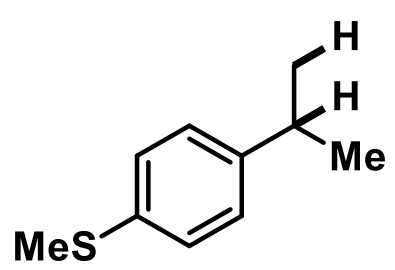

Compound 3i was prepared following the general procedure B, using corresponding alkene. The crude residue was purified by silica gel column chromatography (Petroleum ether) to afford the desired product (27 mg, 54%) as a yellow oil. $^1$H NMR (400 MHz, Chloroform-*d*) δ 7.24 – 7.20 (m, 2H), 7.16 (d, *J* = 8.2 Hz, 2H), 2.88 (p, *J* = 6.8 Hz, 1H), 2.48 (s, 3H), 1.24 (d, *J* = 6.8 Hz, 6H). $^{13}$C NMR (101 MHz, Chloroform-d)δ 150.5, 136.5, 128.9, 126.7, 32.6, 25.2, 15.6. HR-MS (EI): m/z calcd for $[M]^+$ $C_{10}H_{13}S^+$, 166.0816; found 166.0819.

**2-(1-phenylethyl)pyridine (3j)**

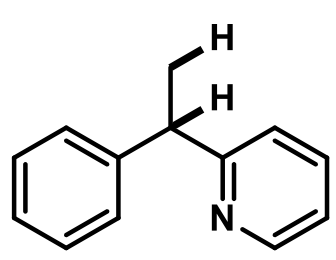

Compound **3j** was prepared following the general procedure A, using corresponding alkene. The crude residue was purified by silica gel column chromatography (Hexane / ethyl acetate = 10/1) to afford the desired product (37 mg, 67%) as a colorless oil. $^1$H NMR (400 MHz, Chloroform-*d*) δ 8.57-8.56 (m, 1H), 7.58-7.54 (m, 1H), 7.33 – 7.28 (m, 4H), 7.23-7.17 (m, 1H), 7.14 – 7.07 (m, 2H), 4.30 (q, *J* = 7.2 Hz, 1H), 1.71 (d, *J* = 7.2 Hz, 3H). $^{13}$C NMR (101 MHz, Chloroform-*d*) δ 165.1, 149.2, 145.1,

136.6, 128.6, 127.8, 126.4, 122.2, 121.4, 47.4, 20.8. HR-MS (ESI): m/z calcd for $[M+H]^+$ $C_{13}H_{14}N^+$, 184.1121; found 184.1119.

**2-isopropylnaphthalene (3k)**

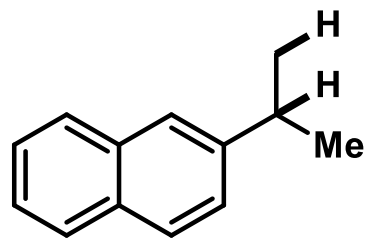

Compound **3k** was prepared following the general procedure A, using corresponding alkene. The crude residue was purified by silica gel column chromatography (Hexane) to afford the desired product (39 mg, 76%) as a colorless oil. $^1H$ NMR (400 MHz, Chloroform-*d*) δ 7.80 (s, 3H), 7.66 (s, 1H), 7.48 – 7.38 (m, 3H), 3.08 (p, *J* = 6.8 Hz, 1H), 1.35 (d, *J* = 6.8 Hz, 6H). $^{13}C$ NMR (101 MHz, Chloroform-*d*) δ 146.4, 133.7, 132.2, 127.9, 127.7, 125.92, 125.87, 125.2, 124.2, 34.3, 24.1. HR-MS (EI): m/z calcd for $[M]^+$ $C_{13}H_{14}^+$, 170.1096; found 170.1096.

**1-methoxy-4-propylbenzene (3l)**

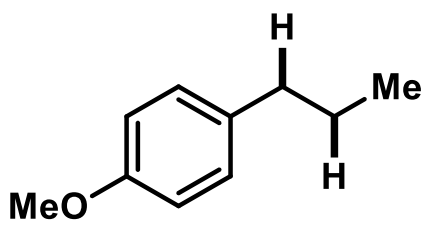

Compound **3l** was prepared following the general procedure A, using corresponding alkene. The crude residue was purified by silica gel column chromatography (Petroleum ether) to afford the desired product (33 mg, 74%) as a yellow oil. $^1H$ NMR (400 MHz, Chloroform-*d*) δ 7.12 – 7.07 (m, 2H), 6.85 – 6.81 (m, 2H), 3.79 (s, 3H), 2.53 (t, *J* = 7.6 Hz, 2H), 1.64 – 1.58 (m, 2H), 0.93 (t, *J* = 7.2 Hz, 3H). $^{13}C$ NMR (101 MHz, Chloroform-*d*) δ 157.7, 134.9, 129.4, 113.7, 55.3, 37.2, 24.9, 13.9. HR-MS (EI): m/z calcd for $[M]^+$ $C_{10}H_{14}O^+$, 150.1045; found 150.1044.

**1,2-diphenylethane (3m)**

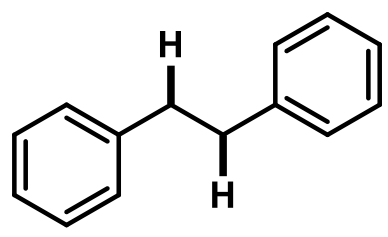

Compound **3m** was prepared following the general procedure A, using corresponding alkene. The crude residue was purified by silica gel column chromatography (Hexane) to afford the desired product (45 mg, 82%) as a colorless oil. $^1H$ NMR (400 MHz, Chloroform-*d*) δ 7.36 – 7.30 (m, 4H), 7.26-7.22 (m, 6H), 2.97 (s, 4H). $^{13}C$ NMR (101 MHz, Chloroform-*d*) δ 141.8, 128.5, 128.4, 126.0, 38.0. HR-MS (EI): m/z calcd for $[M]^+$ $C_{14}H_{14}^+$, 182.1096; found 182.1087.

**2-methyl-3-phenylpropan-1-ol (3n)**

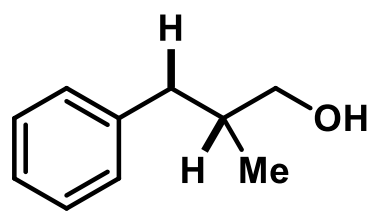

Compound **3n** was prepared following the general procedure B, using corresponding alkene. The crude residue was purified by silica gel column chromatography (Petroleum ether) to afford the desired product (35 mg, 77%) as a yellow oil. $^1H$ NMR (400 MHz, Chloroform-*d*) δ 7.35 – 7.28 (m, 2H), 7.24 – 7.17 (m, 3H), 3.59 – 3.47 (m, 2H), 2.79 (dd, *J* = 13.4, 6.4 Hz, 1H), 2.46 (dd, *J* = 13.4, 8.0 Hz, 1H), 2.02-1.94 (m, 1H), 1.57 (d, *J* = 6.0 Hz, 1H), 0.95 (d, *J* = 6.8 Hz, 3H). $^{13}C$ NMR (101 MHz, Chloroform-*d*) δ 140.7, 129.2, 128.4, 126.0, 67.8, 39.8, 37.9, 16.6. HR-MS (EI): m/z calcd for $[M]^+$ $C_{10}H_{14}O^+$, 150.1045; found 150.1043.

**Isobutylbenzene (3o)**

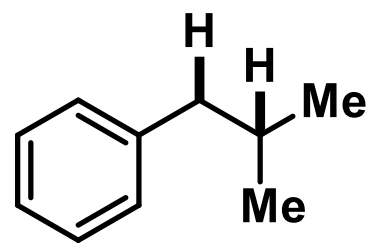

Compound **3o** was prepared following the general procedure B, using corresponding alkene. The crude residue was purified by silica gel column chromatography (Hexane) to afford the desired product (21 mg, 51%) as a colorless oil. $^1H$ NMR (400 MHz, Chloroform-*d*) δ 7.44 – 7.36 (m, 2H), 7.35 – 7.23 (m, 3H), 2.62 (d, *J* = 7.2 Hz, 2H), 2.06 – 1.95 (m, 1H), 1.09 – 1.01 (m, 6H). $^{13}C$ NMR (101 MHz, Chloroform-*d*) δ 141.8, 129.3, 128.2, 125.8, 45.6, 30.4, 22.5. HR-MS (EI): m/z calcd for $[M]^+$ $C_{10}H_{14}^+$, 134.1096; found 134.1094.

**Cyclohexylbenzene (3p)**

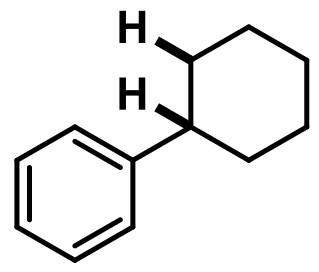

Compound **3p** was prepared following the general procedure B, using corresponding alkene. The crude residue was purified by silica gel column chromatography (Petroleum ether) to afford the desired product (30 mg, 63%) as a colorless oil. $^1$H NMR (400 MHz, Chloroform-*d*) δ 7.35 (t, *J* = 6.8 Hz, 2H), 7.25 (dd, *J* = 18.2, 7.6 Hz, 3H), 2.60 – 2.53 (m, 1H), 2.01 – 1.87 (m, 4H), 1.82 (d, *J* = 11.2 Hz, 1H), 1.55 – 1.42 (m, 4H), 1.38 – 1.27 (m, 1H). $^{13}$C NMR (101 MHz, Chloroform-*d*) δ 148.2, 128.4, 127.0, 125.9, 44.7, 34.6, 27.1, 26.3. HR-MS (EI): m/z calcd for $[M]^+$ $C_{12}H_{16}^+$, 160.1252; found 160.1250.

**(R)-(3-methylbutan-2-yl)benzene（3q）**

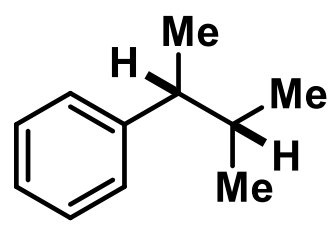

Compound **3q** was prepared following the general procedure B, using corresponding alkene. The crude residue was purified by silica gel column chromatography (Hexane) to afford the desired product (33 mg, 74%) as a colorless oil. $^1$H NMR (400 MHz, Chloroform-*d*) δ 7.27 – 7.21 (m, 2H), 7.13 (t, *J* = 8.8 Hz, 3H), 2.42 - 2.35 (m, 1H), 1.79 – 1.69 (m, 1H), 1.23 – 1.19 (d, *J* = 7.0 Hz, 3H), 0.90 (d, *J* = 6.6 Hz, 3H), 0.72 (d, *J* = 6.6 Hz, 3H). $^{13}$C NMR (101 MHz, Chloroform-*d*) δ 147.2, 128.1, 127.7, 125.8, 46.9, 34.5, 21.3, 20.3, 18.9. HR-MS (EI): m/z calcd for $[M]^+$ $C_{11}H_{16}^+$, 148.1252; found 148.1248.

**butylbenzene (3r)**

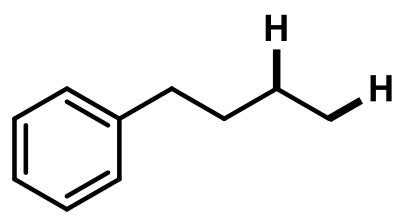

Compound **3r** was prepared following the general procedure A, using corresponding alkene. The crude residue was purified by silica gel column chromatography (Hexane) to afford the desired product (29 mg, 71%) as a colorless oil. $^1$H NMR (400 MHz, Chloroform-*d*) δ 7.35 – 7.28 (m, 2H), 7.25 – 7.18 (m, 3H), 2.68 – 2.63 (m, 2H), 1.70 – 1.61 (m, 2H), 1.46 – 1.35 (m, 2H), 0.98 (td, *J* = 7.4, 3.6 Hz, 3H). $^{13}$C NMR (101 MHz, Chloroform-*d*) δ 143.0, 128.5, 128.3, 125.7, 35.8, 33.8, 22.5, 14.1. HR-MS (EI): m/z calcd for $[M]^+$ $C_{10}H_{14}^+$, 134.1096; found 134.1095.

**1-methoxy-4-propylbenzene (3s)**

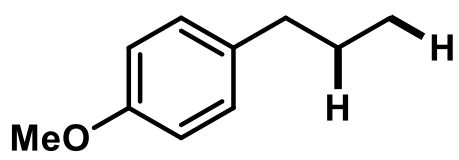

Compound **3s** was prepared following the general procedure A, using corresponding alkene. The crude residue was purified by silica gel column chromatography (Petroleum ether) to afford the desired product (24 mg, 53%) as a yellow oil. $^1$H NMR (400 MHz, Chloroform-*d*) δ 7.12 – 7.07 (m, 2H), 6.85 – 6.81 (m, 2H), 3.79 (s, 3H), 2.53 (t, *J* = 7.6 Hz, 2H), 1.64 – 1.58 (m, 2H), 0.93 (t, *J* = 7.2 Hz, 3H). $^{13}$C NMR (101 MHz, Chloroform-*d*) δ 157.7, 134.9, 129.4, 113.7, 55.3, 37.2, 24.9, 13.9. HR-MS (EI): m/z calcd for $[M]^+$ $C_{10}H_{14}O^+$, 150.1045; found 150.1044.

**(4-methylcyclohexyl)benzene (3t)**

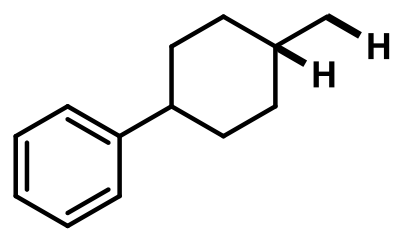

Compound **3t** was prepared following the general procedure A, using corresponding alkene. The crude residue was purified by silica gel column chromatography (Hexane) to afford the desired product (50 mg, 96%) as a yellow solid. $^1$H NMR (400 MHz, Chloroform-*d*) δ 7.31-7.27 (m, 2H), 7.24 – 7.14 (m, 3H), 2.49-2.21 (m, 1H), 1.92 – 1.77 (m, 4H), 1.53 – 1.41 (m, 3H), 1.13-1.06 (m, 2H), 0.94 (d, *J* = 6.4 Hz, 3H). $^{13}$C NMR (101 MHz, Chloroform-*d*) δ 148.0, 128.4, 127.0, 125.9, 44.3, 35.7, 34.4, 32.5, 22.8. HR-MS (EI): m/z calcd for $[M]^+$ $C_{13}H_{18}^+$, 174.1409; found 174,1406.

**4-propoxy-1,1'-biphenyl (3u)**

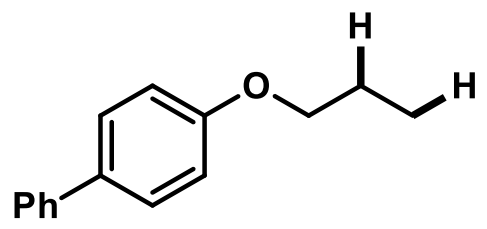

Compound **3u** was prepared following the general procedure B, using corresponding alkene. The crude residue was purified by silica gel column chromatography (Hexane) to afford the desired product (34 mg, 54%) as a white solid. $^1$H NMR (400 MHz, Chloroform-*d*) δ 7.59 – 7.52 (m, 4H), 7.43 (t, *J* = 7.6 Hz, 2H), 7.32 (t, *J* = 7.2 Hz, 1H), 7.03 – 6.97 (m, 2H), 3.99 (t, *J* = 6.6 Hz, 2H), 1.90-1.81 (m, 2H), 1.08 (t, *J* = 7.4 Hz, 3H). $^{13}$C NMR (101 MHz, Chloroform-*d*) δ 158.8, 141.0, 133.7, 128.8, 128.2, 126.8, 126.7, 114.9, 69.7, 22.7, 10.7. HR-MS (ESI): m/z calcd for $[M+Na]^+$ $C_{15}H_{16}NaO^+$, 235.1093; found 235.1092.

**1-cyclohexyl-4-propoxybenzene (3v)**

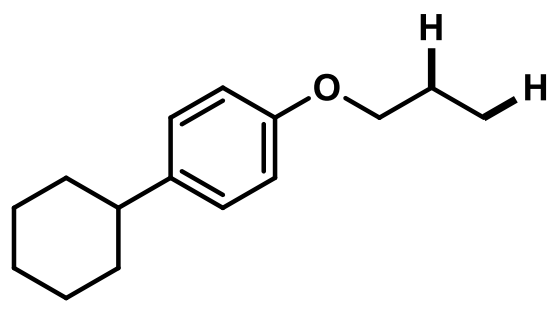

Compound **3v** was prepared following the general procedure B, using corresponding alkene. The crude residue was purified by silica gel column chromatography (Hexane) to afford the desired product (43 mg, 66%) as a colorless oil. $^1$H NMR (400 MHz, Chloroform-*d*) δ 7.13 (d, *J* = 8.4 Hz, 2H), 6.85 (d, *J* = 8.2 Hz, 2H), 3.91 (t, *J* = 6.6 Hz, 2H), 2.46 (s, 1H), 1.88 – 1.74 (m, 7H), 1.45 – 1.37 (m, 4H), 1.29 – 1.23 (m, 1H), 1.04 (t, *J* = 7.4 Hz, 3H). $^{13}$C NMR (101 MHz, Chloroform-*d*) δ 157.3, 140.3, 127.7, 114.4, 69.6, 43.8, 34.8, 27.1, 26.3, 22.8, 10.7. HR-MS (ESI): m/z calcd for $[M+H]^+$ $C_{15}H_{23}O^+$, 219.1743; found 219.1744.

**(propoxymethyl)benzene (3w)**

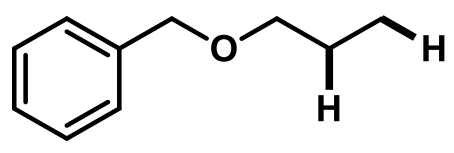

Compound **3w** was prepared following the general procedure A, using corresponding alkene. The crude residue was purified by silica gel column chromatography (Hexane) to afford the desired product (31 mg, 69%) as a colorless oil. $^1$H NMR (400 MHz, Chloroform-*d*) δ 7.27 (d, *J* = 4.4 Hz, 4H), 7.22 – 7.17 (m, 1H), 4.43 (s, 2H), 3.36 (t, *J* = 6.8 Hz, 2H), 1.57 (q, *J* = 7.2 Hz, 2H), 0.87 (t, *J* = 7.4 Hz, 3H). $^{13}$C NMR (101 MHz, Chloroform-*d*) δ 138.8, 128.5, 127.7, 127.6, 72.9, 72.2, 23.1, 10.7. HR-MS (EI): m/z calcd for $[M]^+$ $C_{10}H_{14}O^+$, 150.1045; found 150.1038.

**1-phenylpentan-1-one (3x)**

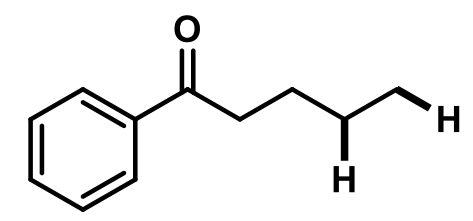

Compound **3x** was prepared following the general procedure A, using corresponding alkene. The crude residue was purified by silica gel column chromatography (Petroleum ether) to afford the desired product (27 mg, 56%) as a yellow oil. $^1$H NMR (400 MHz, Chloroform-*d*) δ 7.98 – 7.91 (m, 2H), 7.55-7.50 (m, 1.6 Hz, 1H), 7.46-7.41 (m, 2H), 2.97-2.93 (m, 2H), 1.75-1.67 (m, 2H), 1.45 – 1.35 (m, 2H), 0.94 (t, *J* = 7.4 Hz, 3H). $^{13}$C NMR (101 MHz, Chloroform-*d*) δ 200.7, 137.1, 133.0, 128.6, 128.1, 38.4, 26.5, 22.6, 14.0. HR-MS (EI): m/z calcd for $[M]^+$ $C_{11}H_{14}O^+$, 162.1045; found 162.1041.

**2-butyl-1H-indene-1,3(2H)-dione (3y)**

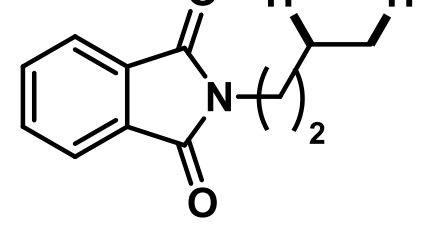

Compound **3y** was prepared following the general procedure B, using corresponding alkene. The crude residue was purified by silica gel column chromatography (Hexane / ethyl acetate = 20/1) to afford the desired product (30 mg, 50%) as a colorless oil. $^1$H NMR (400 MHz, Chloroform-*d*) δ 7.86 – 7.80 (m, 2H), 7.70 (dt, *J* = 5.4, 2.4 Hz, 2H), 3.70 – 3.66 (m, 2H), 1.70 – 1.62 (m, 2H), 1.41 – 1.32 (m, 2H), 0.94 (td, *J* = 7.4, 1.6 Hz, 3H). $^{13}$C NMR (101 MHz, Chloroform-*d*) δ 168.6, 133.9, 132.2,

123.2, 37.9, 30.7, 20.1, 13.7. HR-MS (ESI): m/z calcd for $[M+H]^+$ $C_{12}H_{14}NO_2^+$, 204.1019; found 204.1013.

**2-propylisoindoline-1,3-dione (3z)**

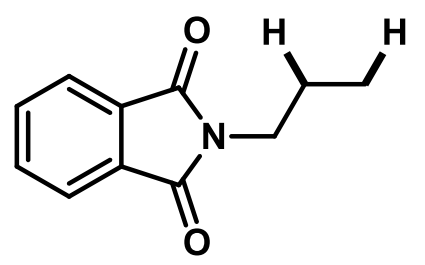

Compound **3z** was prepared following the general procedure A, using corresponding alkene. The crude residue was purified by silica gel column chromatography (Hexane/ethyl acetate = 20/1) to afford the desired product (36 mg, 63%) as a colorless oil. $^1$H NMR (400 MHz, Chloroform-*d*) δ 7.83 – 7.80 (m, 2H), 7.71 – 7.68 (m, 2H), 3.63 (t, *J* = 7.2 Hz, 2H), 1.75 – 1.64 (m, 2H), 0.93 (t, *J* = 7.4 Hz, 3H). $^{13}$C NMR (101 MHz, Chloroform-*d*) δ 168.6, 133.9, 132.2, 123.2, 39.7, 22.0, 11.4. HR-MS (EI): m/z calcd for $[M]^+$ $C_{11}H_{11}NO_2^+$, 189.0790; found 189.0779.

***N*-ethyl-*N*-phenylundecanamide (3aa)**

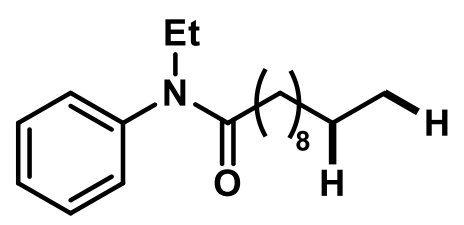

Compound **3aa** was prepared following the general procedure A, using corresponding alkene. The crude residue was purified by silica gel column chromatography (Petroleum ether) to afford the desired product (52 mg, 60%) as a colorless oil. $^1$H NMR (400 MHz, Chloroform-*d*) δ 7.41 (dd, *J* = 8.2, 6.6 Hz, 2H), 7.37 – 7.31 (m, 1H), 7.16 – 7.10 (m, 2H), 3.73 (q, *J* = 7.2 Hz, 2H), 1.99 (t, *J* = 7.6 Hz, 2H), 1.53 (p, *J* = 7.0 Hz, 2H), 1.28 – 1.12 (m, 14H), 1.09 (t, *J* = 7.0 Hz, 3H), 0.86 (t, *J* = 6.8 Hz, 3H). $^{13}$C NMR (101 MHz, Chloroform-*d*) δ 172.8, 142.7, 129.7, 128.6, 127.8, 44.0, 34.6, 32.0, 29.6, 29.5, 29.39, 29.36, 25.6, 22.7, 14.2, 13.2. HR-MS (ESI): m/z calcd for $[M+H]^+$ $C_{19}H_{32}NO^+$, 290.2478; found 290.2471.

**benzyl undecanoate (3ab)**

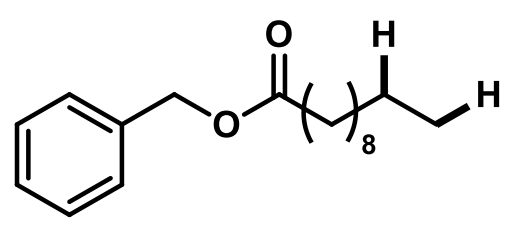

Compound **3ab** was prepared following the general procedure B, using corresponding alkene. The crude residue was purified by silica gel column chromatography (Petroleum ether) to afford the desired product (54 mg, 65%) as a colorless oil. $^1$H NMR (400 MHz, Chloroform-*d*) δ 7.38 – 7.30 (m, 5H), 5.11 (s, 2H), 2.35 (t, *J* = 7.6 Hz, 2H), 1.68 – 1.62 (m, 2H), 1.30 – 1.25 (m, 14H), 0.88 (t, *J* = 6.8 Hz, 3H). $^{13}$C NMR (101 MHz, Chloroform-*d*) δ 173.9, 136.2, 128.6, 128.3, 66.2, 34.4, 32.0, 29.6, 29.5, 29.4, 29.3, 29.2, 25.0, 22.8, 14.2. HR-MS (ESI): m/z calcd for $[M+NH_4]^+$ $C_{18}H_{32}NO_2^+$, 294.2428; found 294.2428.

**hexyl 4-(trifluoromethyl)benzoate (3ac)**

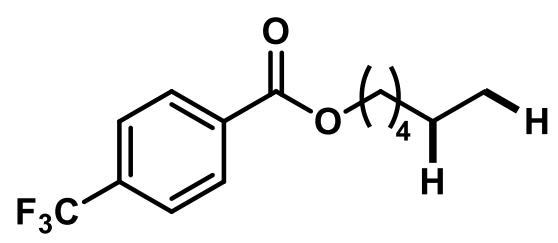

Compound **3ac** was prepared following the general procedure B, using corresponding alkene. The crude residue was purified by silica gel column chromatography (Hexane /DCM=5:1) to afford the desired product (40 mg, 49%) as a colorless oil. $^1$H NMR (400 MHz, Chloroform-*d*) δ 8.15 (d, *J* = 8.2 Hz, 2H), 7.70 (d, *J* = 8.2 Hz, 2H), 4.35 (t, *J* = 6.8 Hz, 2H), 1.83 – 1.72 (m, 2H), 1.49 – 1.40 (m, 2H), 1.38 – 1.29 (m, 4H), 0.92 – 0.88 (m, 3H). $^{19}$F NMR (376 MHz, Chloroform-*d*) δ -63.10. $^{13}$C NMR (101 MHz, Chloroform-*d*) δ 165.6, 134.4(d, *J* = 32.6 Hz), 133.8, 130.0, 125.4(d, *J* = 3.8 Hz), 122.4(d, *J* = 273.9 Hz), 65.8, 31.5, 28.7, 25.7, 22.6, 14.1. HR-MS (EI): m/z calcd for $[M]^+$ $C_{14}H_{17}F_3O^+$, 274.1181; found 274.1185.

**undecyl nicotinate (3ad)**

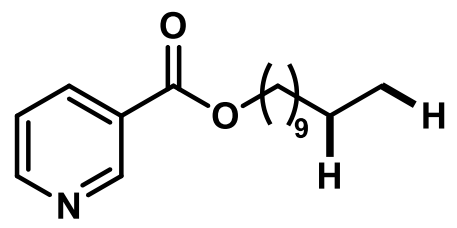

Compound **3ad** was prepared following the general procedure B, using corresponding alkene. The crude residue was purified by silica gel column chromatography (DCM) to afford the desired product (59 mg, 71%) as a

colorless oil. $^1$H NMR (400 MHz, Chloroform-*d*) δ 9.25 – 9.18 (m, 1H), 8.76 (d, *J* = 4.8 Hz, 1H), 8.30 – 8.27 (m, 1H), 7.38 (dd, *J* = 8.0, 4.8 Hz, 1H), 4.34 (t, *J* = 6.8 Hz, 2H), 1.77 (p, *J* = 7.2 Hz, 2H), 1.40 – 1.20 (m, 16H), 0.86 (t, *J* = 6.8 Hz, 3H). $^{13}$C NMR (101 MHz, Chloroform-*d*) δ 165.4, 153.4, 151.0, 137.1, 126.5, 123.4, 65.7, 32.0, 29.67, 29.65, 29.6, 29.4, 29.3, 28.7, 26.1, 22.8, 14.2. HR-MS (ESI): m/z calcd for $[M+H]^+$ $C_{17}H_{28}NO_2^+$, 278.2115; found 278.2112.

**diphenyldipropylsilane (3ae)**

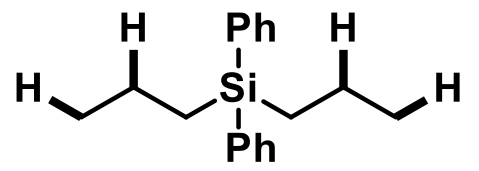


Compound **3ae** was prepared following the general procedure B, using corresponding alkene. The crude residue was purified by silica gel column chromatography (Petroleum ether) to afford the desired product (64 mg, 80%) as a colorless oil. $^1$H NMR (400 MHz, Chloroform-*d*) δ 7.54 – 7.49 (m, 4H), 7.38-7.33 (m, 6H), 1.46 – 1.36 (m, 4H), 1.13 – 1.06 (m, 4H), 0.98 (t, *J* = 7.2 Hz, 6H). $^{13}$C NMR (101 MHz, Chloroform-*d*) δ 136.8, 135.0, 129.1, 127.8, 18.6, 17.5, 15.4. HR-MS (EI): m/z calcd for $[M]^+$ $C_{18}H_{24}Si^+$, 268.1647; found 268.1646.

**1-hexyl-1H-benzo[d][1,2,3]triazole (3af)**

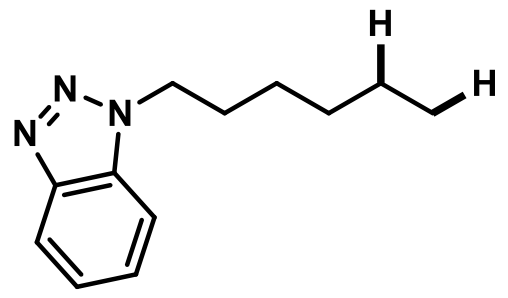


Compound **3af** was prepared following the general procedure A, using corresponding alkene. The crude residue was purified by silica gel column chromatography (Hexane / ethyl acetate = 6/1) to afford the desired product (51 mg, 84%) as a yellow oil. $^1$H NMR (400 MHz, Chloroform-*d*) δ 8.08-8.05 (m, 1H), 7.55 – 7.45 (m, 2H), 7.39-7.35 (m, 1H), 4.64 (t, *J* = 7.2 Hz, 2H), 2.04 – 1.97 (m, 2H), 1.35 – 1.25 (m, 6H), 0.89 – 0.84 (m, 3H). $^{13}$C NMR (101 MHz, Chloroform-*d*) δ 146.1, 133.0, 127.2, 123.8, 120.1, 109.4, 48.3, 31.3, 29.8, 26.5, 22.5, 14.0. HR-MS (ESI): m/z calcd for $[M+H]^+$ $C_{12}H_{18}N_3^+$, 204.1495; found 204.1487.

***N*-propylaniline (3ag)**

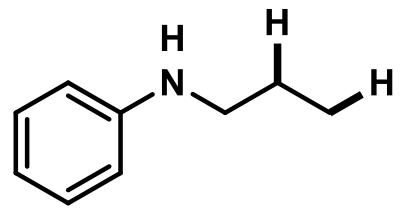


Compound **3ag** was prepared following the general procedure A, using corresponding alkene. The crude residue was purified by silica gel column chromatography (Petroleum ether) to afford the desired product (18 mg, 44%) as a colorless oil. $^1$H NMR (400 MHz, Chloroform-*d*) δ 7.24 – 7.17 (m, 2H), 6.73 – 6.69 (m, 1H), 6.66 – 6.61 (m, 2H), 3.69 (s, 1H), 3.10 (t, *J* = 7.2 Hz, 2H), 1.67 (h, *J* = 7.2 Hz, 2H), 1.02 (t, *J* = 7.4 Hz, 3H). $^{13}$C NMR (101 MHz, Chloroform-*d*) δ 148.6, 129.3, 117.2, 112.8, 45.9, 22.8, 11.8. HR-MS (ESI): m/z calcd for $[M+H]^+$ $C_9H_{14}N^+$, 136.1121; found 136.1116.

***N*-methyl-*N*-(naphthalen-1-ylmethyl)butan-1-amine (3ah)**

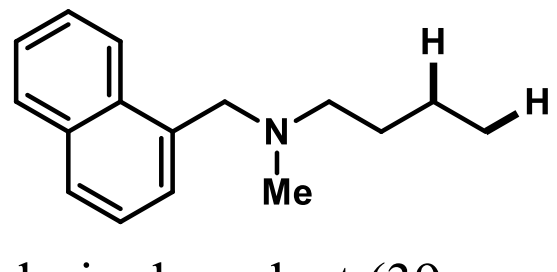


Compound **3ah** was prepared following the general procedure A, using corresponding alkene. The crude residue was purified by silica gel column chromatography (Hexane / ethyl acetate = 10/1) to afford the desired product (39 mg, 58%) as a colorless oil. $^1$H NMR (400 MHz, Chloroform-*d*) δ 8.35 – 8.30 (m, 1H), 7.86 (dd, *J* = 7.8, 1.6 Hz, 1H), 7.78 (dd, *J* = 7.6, 2.0 Hz, 1H), 7.55 – 7.40 (m, 4H), 3.90 (s, 2H), 2.53 – 2.47 (m, 2H), 2.22 (s, 3H), 1.64 – 1.54 (m, 2H), 1.40 – 1.33 (m, 2H), 0.93 (t, *J* = 7.2 Hz, 3H). $^{13}$C NMR (101 MHz, Chloroform-*d*) δ 135.3, 133.9, 132.6, 128.5, 127.9, 127.4, 125.9, 125.6, 125.2, 124.8, 60.9, 58.2, 42.4, 29.7, 20.8, 14.2. HR-MS (ESI): m/z calcd for $[M+H]^+$ $C_{16}H_{22}N^+$, 228.1747; found 228.1742.

**tert-butyl hexyl(tosyl)carbamate (3ai)**

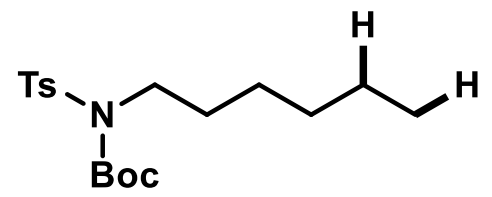

Compound **3ai** was prepared following the general procedure A, using corresponding alkene. The crude residue was purified by silica gel column chromatography (Hexane / ethyl acetate = 20/1) to afford the desired product (51 mg, 48%) as a yellow oil. $^1$H NMR (400 MHz, Chloroform-*d*) δ 7.77 (d, *J* = 8.0 Hz, 2H), 7.29 (d, *J* = 8.0 Hz, 2H), 3.84 – 3.76 (m, 2H), 2.43 (s, 3H), 1.78-1.70 (m, 2H), 1.33 (s, 15H), 0.93 – 0.85 (m, 3H). $^{13}$C NMR (101 MHz, Chloroform-*d*) δ 151.1, 144.0, 137.7, 129.3, 127.9, 84.1, 47.3, 31.5, 30.2, 28.0, 26.4, 22.7, 21.7, 14.1. HR-MS (ESI): m/z calcd for $[M+NH_4]^+$ $C_{18}H_{33}N_2O_4S^+$,373.2156; found 373.2153.

**dodecane (3aj)**

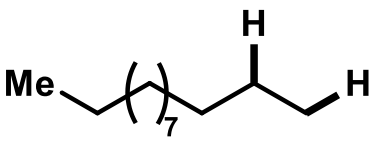

Compound **3aj** was prepared following the general procedure A, using corresponding alkene. The crude residue was purified by silica gel column chromatography (Petroleum ether) to afford the desired product (36 mg, 70%) as a colorless oil. $^1$H NMR (400 MHz, Chloroform-*d*) δ 1.27 (s, 20H), 0.89 (t, *J* = 6.8 Hz, 6H). $^{13}$C NMR (101 MHz, Chloroform-*d*) δ 32.0, 29.81, 29.77, 29.5, 22.8, 14.2. HR-MS (EI): m/z calcd for $[M]^+$ $C_{12}H_{26}^+$, 170.2035; found 170.2035.

**1-chloroundecane (3ak)**

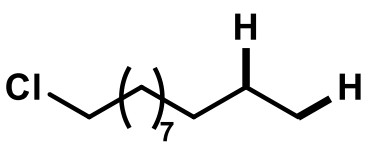

Compound **3ak** was prepared following the general procedure A, using corresponding alkene. The crude residue was purified by silica gel column chromatography (Hexane / ethyl acetate = 20/1) to afford the desired product (43 mg, 76%) as a colorless oil. $^1$H NMR (400 MHz, Chloroform-*d*) δ 3.52 (t, *J* = 6.8 Hz, 2H), 1.76 (dt, *J* = 14.4, 6.8 Hz, 2H), 1.46-1.39 (m, 2H), 1.34-1.27 (m, 14H), 0.88 (t, *J* = 6.8 Hz, 3H). $^{13}$C NMR (101 MHz, Chloroform-*d*) δ 45.2, 32.7, 32.0, 29.7, 29.67, 29.64, 29.4, 29.0, 27.0, 22.8, 14.2. HR-MS (EI): m/z calcd for $[M]^+$ $C_{11}H_{23}Cl^+$, 190.1488; found 190.1484.

**methyl undecanoate (3al)**

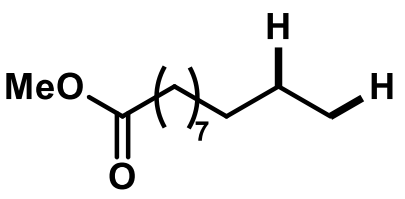

Compound **3al** was prepared following the general procedure B, using corresponding alkene. The crude residue was purified by silica gel column chromatography (Petroleum ether) to afford the desired product (32 mg, 53%) as a colorless oil. $^1$H NMR (400 MHz, Chloroform-*d*) δ 3.65 (s, 3H), 2.28 (t, *J* = 7.6 Hz, 2H), 1.65-1.55 (m, 2H), 1.32-1.20 (m, 14H), 0.86 (t, *J* = 6.8 Hz, 3H). $^{13}$C NMR (101 MHz, Chloroform-*d*) δ 174.4, 51.5, 34.2, 32.0, 29.6, 29.5, 29.4, 29.3, 29.2, 25.0, 22.7, 14.2. HR-MS (ESI): m/z calcd for $[M+H]^+$ $C_{12}H_{25}O_2^+$,201.1849; found 201.1849.

**octan-3-ol (3am)**

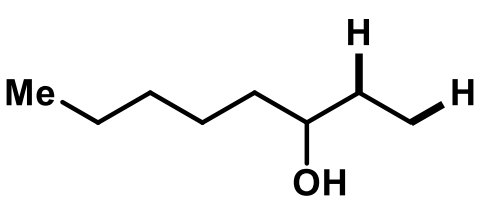

Compound **3am** was prepared following the general procedure A, using corresponding alkene. The crude residue was purified by silica gel column chromatography (Petroleum ether) to afford the desired product (28 mg, 73%) as a colorless oil. $^1$H NMR (400 MHz, Chloroform-*d*) δ 3.54-3.47 (m, 1H), 1.59 – 1.26 (m, 11H), 0.95-0.85 (m, 6H). $^{13}$C NMR (101 MHz, Chloroform-*d*) δ 73.4, 37.0, 32.0, 30.2, 25.4, 22.7, 14.1, 9.9. HR-MS (EI): m/z calcd for $[M-H_2O]^+$ C8H18O$^+$, 112.1252; found 112.1247.

**1-ethyl-4-((((1*S*,2*R*,5*S*)-2-isopropyl-5-methylcyclohexyl)oxy)methyl)benzene (3ar)**

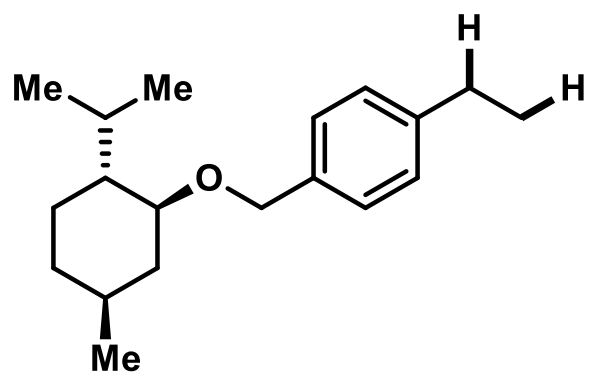


Compound **3ar** was prepared following the general procedure A, using corresponding alkene. The crude residue was purified by silica gel column chromatography (Hexane / ethyl acetate = 20/1) to afford the desired product (75 mg, 91%) as a colorless oil. $^{1}$H NMR (400 MHz, Chloroform-*d*) δ 7.28 (d, *J* = 8.0 Hz, 2H), 7.18 (d, *J* = 7.8 Hz, 2H), 4.64 (d, *J* = 11.2 Hz, 1H), 4.38 (d, *J* = 11.2 Hz, 1H), 3.21-3.15 (m, 1H), 2.65 (q, *J* = 7.6 Hz, 2H), 2.36-2.28 (m, 1H), 2.23-2.18 (m, 1H), 1.70-1.61 (m, 2H), 1.40 – 1.21 (m, 5H), 1.05 – 0.82 (m, 9H), 0.73 (d, *J* = 6.8 Hz, 3H). $^{13}$C NMR (101 MHz, Chloroform-*d*) δ 143.6, 136.4, 128.1, 127.9, 78.7, 70.4, 48.4, 40.4, 34.7, 31.7, 28.7, 25.6, 23.3, 22.5, 21.1, 16.1, 15.8. HR-MS (ESI): m/z calcd for $[M+NH_4]^+$ $C_{19}H_{34}NO^+$,292.2635; found 292.2630.

**1-(3-((4-ethylbenzyl)oxy)-2,2-dimethylpropyl)-3-methylbenzene (3as)**

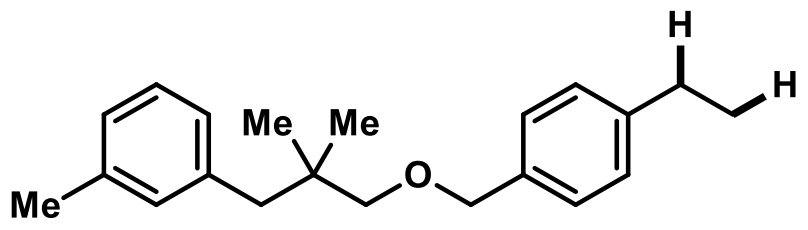


Compound **3as** was prepared following the general procedure B, using corresponding alkene. The crude residue was purified by silica gel column chromatography (Hexane / ethyl acetate = 20/1) to afford the desired product (79 mg, 89%) as a colorless oil. $^{1}$H NMR (400 MHz, Chloroform-*d*) δ 7.32 (d, *J* = 8.0 Hz, 2H), 7.21 (d, *J* = 8.0 Hz, 2H), 7.16-7.13 (m, 1H), 7.02 (d, *J* = 7.6 Hz, 1H), 6.99 – 6.93 (m, 2H), 4.51 (s, 2H), 3.09 (s, 2H), 2.68 (q, *J* = 7.6 Hz, 2H), 2.60 (s, 2H), 2.33 (s, 3H), 1.27 (t, *J* = 7.6 Hz, 3H), 0.92 (s, 6H). $^{13}$C NMR (101 MHz, Chloroform-*d*) δ 143.5, 139.2, 137.2, 136.3, 131.5, 127.9, 127.8, 127.7, 127.6, 126.6, 78.2, 73.1, 44.9, 35.8, 28.7, 24.9, 21.6, 15.7. HR-MS (ESI): m/z calcd for $[M+H]^+$ $C_{21}H_{29}O^+$,297.2213; found 297.2213.

**2,2-dimethyl-3-(m-tolyl)propyl 4-ethylbenzoate (3at)**

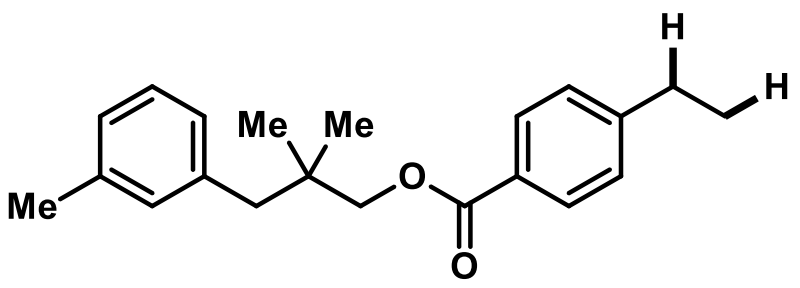


Compound **3at** was prepared following the general procedure A, using corresponding alkene. The crude residue was purified by silica gel column chromatography (Hexane / ethyl acetate = 20/1) to afford the desired product (47 mg, 51%) as a colorless oil. $^{1}$H NMR (400 MHz, Chloroform-*d*) δ 8.02 – 7.98 (m, 2H), 7.31 – 7.28 (m, 2H), 7.18-7.14 (m, 1H), 7.04-7.01 (m, 1H), 6.96-6.94 (m, 2H), 4.01 (s, 2H), 2.73 (q, *J* = 7.6 Hz, 2H), 2.67 (s, 2H), 2.31 (s, 3H), 1.28 (t, *J* = 7.6 Hz, 3H), 1.02 (s, 6H). $^{13}$C NMR (101 MHz, Chloroform-*d*) δ 166.7, 149.9, 138.1, 137.5, 131.4, 129.8, 128.1, 127.9, 127.6, 127.0, 72.0, 45.3, 35.4, 29.1, 24.7, 21.5, 15.4. HR-MS (ESI): m/z calcd for $[M+H]^+$ $C_{21}H_{26}O_2^+$, 311.2006; found 311.2003.

**hexyl 2-(4-chlorophenoxy)-2-methylpropanoate (3au)**

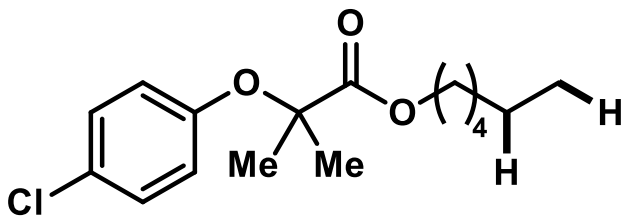


Compound **3au** was prepared following the general procedure B, using corresponding alkene. The crude residue was purified by silica gel column chromatography (Hexane / ethyl acetate = 20/1) to afford the desired product (58 mg, 65%) as a colorless oil. $^{1}$H NMR (400 MHz, Chloroform-*d*) δ 7.21 – 7.16 (m, 2H), 6.80 – 6.75 (m, 2H), 4.15 (t, *J* = 6.6 Hz, 2H), 1.58 (s, 8H), 1.24 (t, *J* = 3.4 Hz, 6H), 0.89 – 0.84 (m, 3H). $^{13}$C NMR (101 MHz, Chloroform-*d*) δ 174.2, 154.2, 129.2, 127.2, 120.4, 65.8, 79.5, 31.4, 28.5, 25.5, 25.4, 22.6, 14.0. HR-MS (ESI): m/z calcd for $[M+H]^+$ $C_{16}H_{24}ClO_3^+$, 299.1408; found 299.1401.

**butyl (*S*)-2-(6-methoxynaphthalen-2-yl)propanoate (3av)**

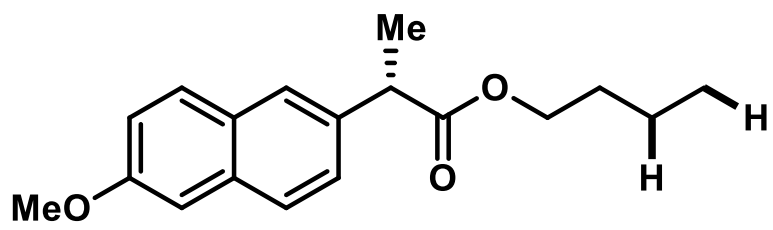

Compound **3av** was prepared following the general procedure B, using corresponding alkene. The crude residue was purified by silica gel column chromatography (Hexane / ethyl acetate = 20/1) to afford the desired product (61 mg, 71%) as a white solid. $^{1}$H NMR (400 MHz, Chloroform-*d*) δ 7.72 – 7.66 (m, 3H), 7.41 (dd, *J* = 8.4, 2.0 Hz, 1H), 7.16 – 7.10 (m, 2H), 4.11-4.04 (m, 2H), 3.91 (s, 3H), 3.84 (q, *J* = 7.2 Hz, 1H), 1.59-1.52 (m, 5H), 1.34 – 1.24 (m, 2H), 0.86 (t, *J* = 7.4 Hz, 3H). $^{13}$C NMR (101 MHz, Chloroform-*d*) δ 174.9, 157.7, 135.9, 133.7, 129.4, 129.0, 127.2, 126.4, 126.0, 119.0, 105.6, 64.7, 55.4, 45.6, 30.7, 19.1, 18.6, 13.7. HR-MS (ESI): m/z calcd for $[M+NH_4]^+$ $C_{18}H_{26}NO_3^+$,304.1907; found 304.1905.

**hexyl (*S*)-2-(6-methoxynaphthalen-2-yl)propanoate (3aw)**

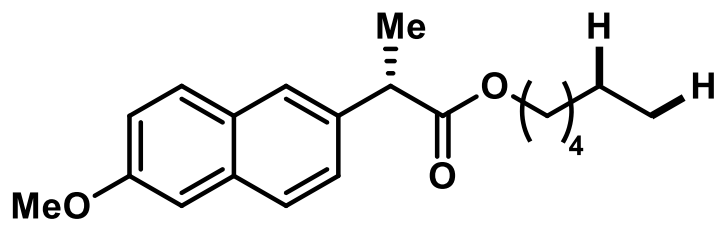

Compound **3aw** was prepared following the general procedure B, using corresponding alkene. The crude residue was purified by silica gel column chromatography (Hexane / ethyl acetate = 20/1) to afford the desired product (68 mg, 72%) as a yellow oil. $^{1}$H NMR (400 MHz, Chloroform-*d*) δ 7.72 – 7.66 (m, 3H), 7.41 (dd, *J* = 8.4, 2.0 Hz, 1H), 7.16 – 7.10 (m, 2H), 4.07 (t, *J* = 6.8 Hz, 2H), 3.91 (s, 3H), 3.84 (q, *J* = 7.2 Hz, 1H), 1.62 – 1.53 (m, 5H), 1.27 – 1.18 (m, 6H), 0.85 – 0.79 (m, 3H). $^{13}$C NMR (101 MHz, Chloroform-*d*) δ 174.9, 157.7, 136.0, 133.8, 129.4, 129.0, 127.2, 126.4, 126.0, 119.0, 105.7, 65.0, 55.4, 45.6, 31.4, 28.6, 25.5, 22.5, 18.6, 14.0. HR-MS (ESI): m/z calcd for $[M+NH_4]^+$ $C_{20}H_{30}NO_3^+$, 332.2220; found 332.2218.

**hexyl (*S*)-2-(4-isobutylphenyl)propanoate (3ax)**

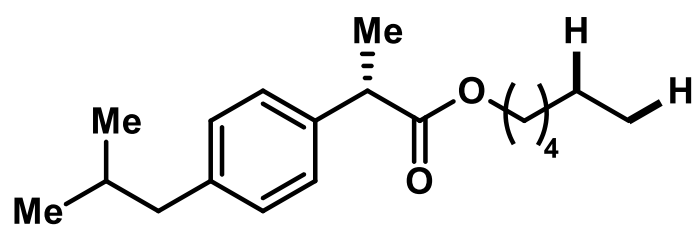

Compound **3ax** was prepared following the general procedure B, using corresponding alkene. The crude residue was purified by silica gel column chromatography (Hexane / ethyl acetate = 20/1) to afford the desired product (60 mg, 69%) as a colorless oil. $^{1}$H NMR (400 MHz, Chloroform-*d*) δ 7.22 – 7.17 (m, 2H), 7.11 – 7.06 (m, 2H), 4.05 (t, *J* = 6.8 Hz, 2H), 3.68 (q, *J* = 7.2 Hz, 1H), 2.44 (d, *J* = 7.2 Hz, 2H), 1.89 – 1.79 (m, 1H), 1.48 (d, *J* = 7.2 Hz, 3H), 1.30 – 1.21 (m, 8H), 0.90 – 0.84 (m, 9H). $^{13}$C NMR (101 MHz, Chloroform-*d*) δ 175.0, 140.5, 138.0, 129.4, 127.2, 64.9, 45.3, 45.1, 31.4, 30.3, 28.6, 25.5, 22.6, 22.5, 18.5, 14.1. HR-MS (ESI): m/z calcd for $[M+H]^+$ $C_{19}H_{31}O_2^+$, 291.2319; found 291.2306.

**hexyl 2-(2-fluoro-[1,1'-biphenyl]-4-yl)propanoate (3ay)**

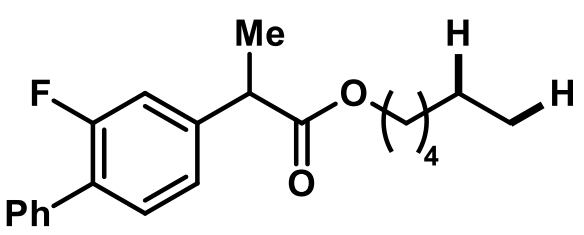

Compound **3ay** was prepared following the general procedure B, using corresponding alkene. The crude residue was purified by silica gel column chromatography (Hexane / ethyl acetate = 20/1) to afford the desired product (58 mg, 59%) as a colorless oil. $^{1}$H NMR (400 MHz, Chloroform-*d*) δ 7.57-7.52 (m, 2H), 7.47 – 7.34 (m, 4H), 7.20 – 7.11 (m, 2H), 4.11 (t, *J* = 6.8 Hz, 2H), 3.76 (q, *J* = 7.2 Hz, 1H), 1.64-1.59 (m, 2H), 1.55 (d, *J* = 7.2 Hz, 3H), 1.32 – 1.22 (m, 6H), 0.93 – 0.83 (m, 3H). $^{19}$F NMR (376 MHz, Chloroform-*d*) δ -105.2. $^{13}$C NMR (101 MHz, Chloroform-*d*) δ 174.2, 159.8 (d, *J* = 248.4 Hz), 142.1(d, *J* = 8.1 Hz), 135.6, 130.8(d, *J* = 4.0 Hz), 129.1(d, *J* = 3.0 Hz), 128.5, 127.9, 127.7, 123.7(d, *J* = 3.0 Hz), 115.3(d, *J* = 24.2 Hz), 65.2, 45.2, 31.4, 28.6, 25.6, 22.6, 18.4, 14.1. HR-MS (ESI): m/z calcd for $[M+NH_4]^+$ $C_{21}H_{29}FNO_2^+$, 346.2177; found 346.2173.

***Unsuccessful substrates***

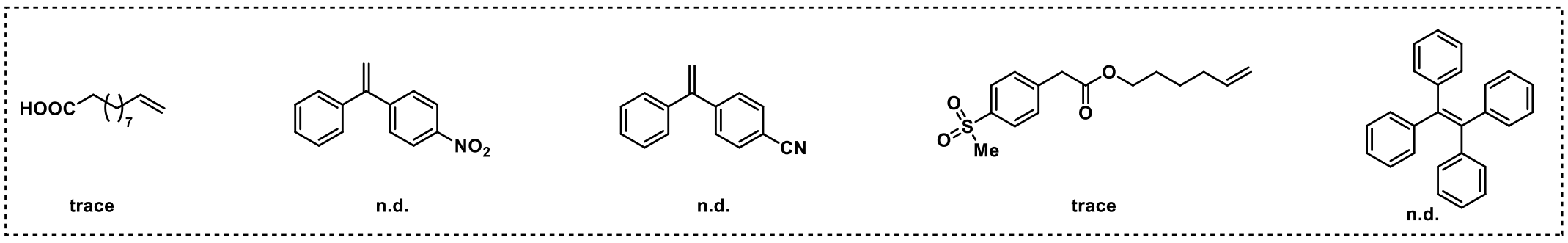


## 7. Procedure for the scale-up reaction

$BaTiO_3$ (2.33 g, 10.0 mmol), KO*t*Bu (67 mg, 0.6 mmol), and *p*-vinylbiphenyl (**1a**, 365 mg, 2.0 mmol) were placed in a 50.0 mL stainless-steel milling jar, along with thirty stainless steel balls (7 mm in diameter) in an air atmosphere. After adding triethylsilane (**2**, 0.63 mL, 4 mmol) to the solid mixture, the jar was sealed and subjected to milling at 30 Hz under air. After grinding for 2 h, the reaction mixture was washed with ethyl acetate, filtered. The yield of **3a** (77% yield) was determined by gas chromatography (GC) using dodecane as an internal standard. Furthermore, *p*-vinylbiphenyl (**1a**) was processed on a 5-mmol scale in a 50 mL stainless steel ball-milling jar containing fifty 7 mm diameter stainless steel balls, which yielded the hydrogenation product (**3a**, 68%) as shown in Figure S5.

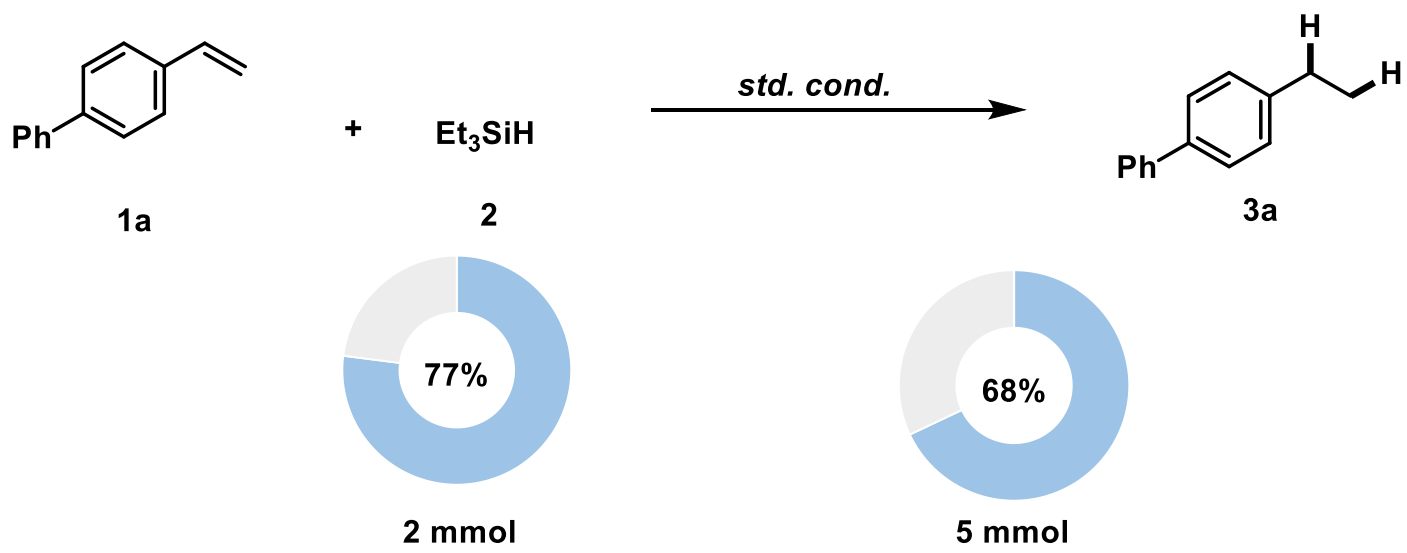


**Figure S5.** Scale-up experiments

## 8. $BaTiO_3$ recycling experiments

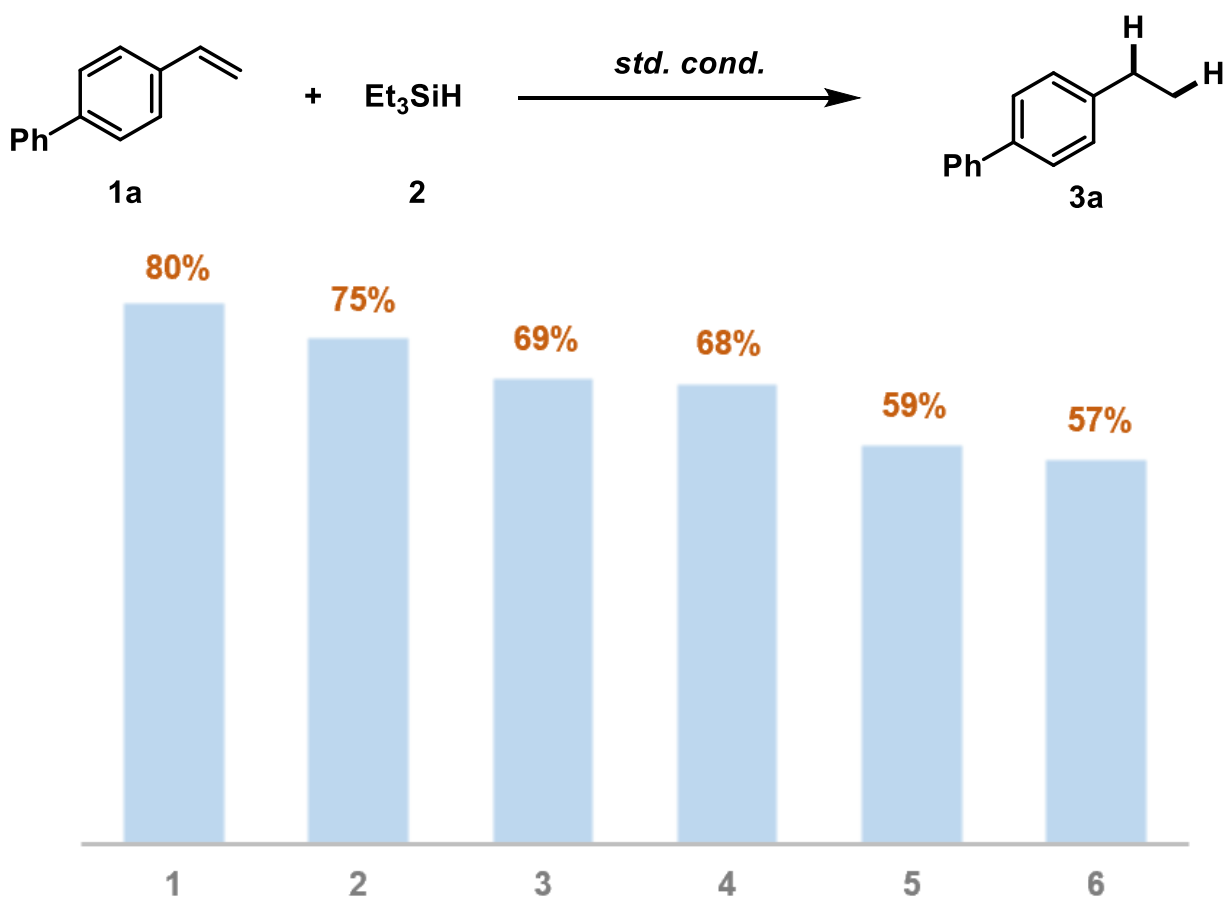


**Figure S6.** $BaTiO_3$ recycling experiments

$BaTiO_3$ (1.5 mmol, 5.0 equiv), KO*t*Bu (10 mg, 0.09 mmol), and *p*-vinylbiphenyl (**1a**, 54 mg, 0.3 mmol) were placed in a 10.0 mL stainless-steel milling jar, along with two stainless-steel balls (10 mm diameter). After adding triethylsilane (**2**, 95 µL, 0.6 mmol) to the solid mixture, the jar was sealed and subjected to milling at 30 Hz under air. After grinding for 2 h, the reaction mixture was washed with ethyl acetate. $BaTiO_3$ was filtrated and washed with ethyl acetate, and dried over under

reduce pressure for 3 h. Then can be reused for hydrogenation of *p*-vinylbiphenyl (**1a**, 0.3 mmol) with triethylsilane (**2**, 0.6 mmol) under the same reaction conditions for at least six times (Figure S6**)**. The yield of hydrogenation product **3a** was determined by GC analysis using dodecane as an internal standard.

## 9. Other control experiment

### 9.1 $^{18}O_2$ Labeled trapping experiment

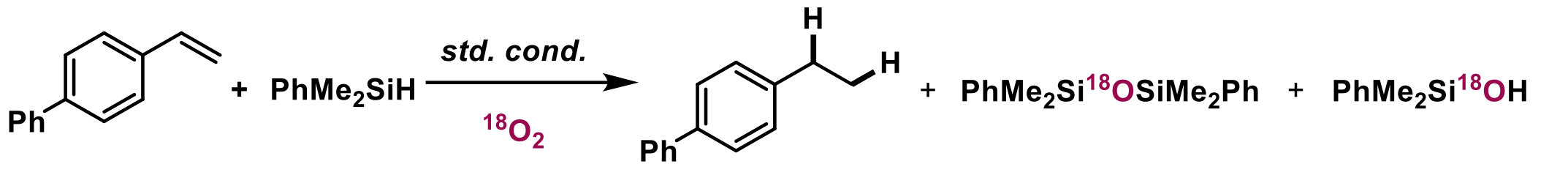


1a 2a 3a, 65% 4, 38% 5 detected by HRMS

$BaTiO_3$ (350 mg, 1.5 mmol), KO*t*Bu (10 mg, 0.09 mmol) and *p*-vinylbiphenyl (**1a**, 54 mg, 0.3 mmol) were placed in a 10.0 mL stainless-steel milling jar, along with two stainless-steel balls (10 mm diameter). After adding $PhMe_2SiH$ (**2a**, 82 mg, 0.6 mmol) to the solid mixture, the jar was sealed and subjected to milling at 30 Hz under $^{18}O_2$. After grinding for 2 h, the reaction mixture was washed with ethyl acetate. The yield of **3a** was determined by GC analysis using dodecane as an internal standard. The yield of **4** was isolated as a colorless oil using petroleum as eluent (16 mg, 38%). $^1$H NMR (400 MHz, Chloroform-*d*) δ 7.66 – 7.64 (m, 4H), 7.46 – 7.44 (m, 6H), 0.47 – 0.40 (m, 12H). HR-MS (EI): m/z calcd for $[M]^+$ $C_{16}H_{22}{}^{18}OSi_2{}^+$, 288.1252; found 288.1249.
The reaction solution was monitored by High-Resolution Mass (HRMS). silanol (**5**) were detected by HRMS. $^{18}$O-labeled silanol (**5**) HRMS (EI): m/z calcd for $[M]^+$ $C_8H_{12}{}^{18}OSi^+$, 154.0700; found 154.0694.

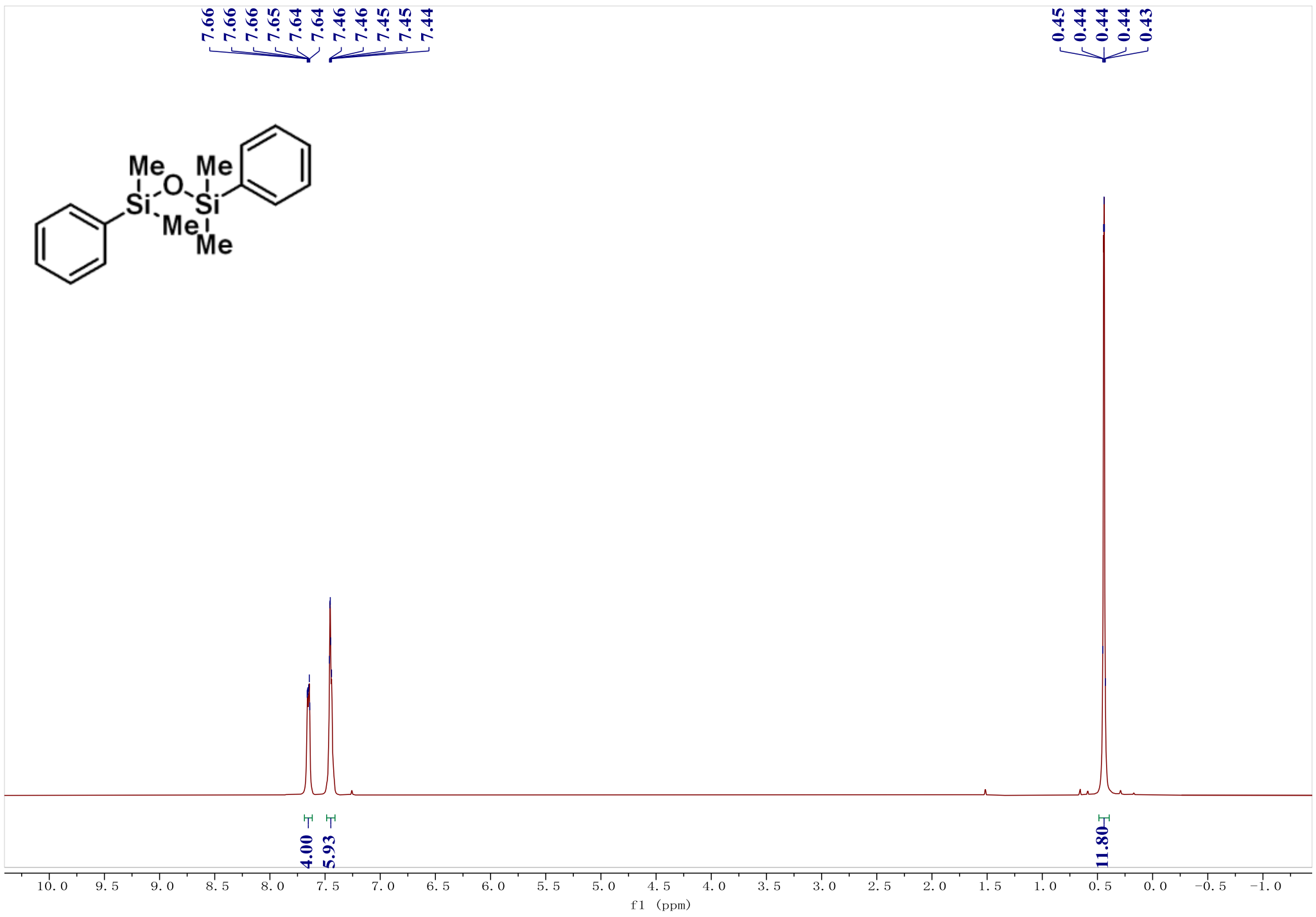


**Figure S7.** $^1$H NMR (400 MHz, $CDCl_3$) spectrum of compound **4**

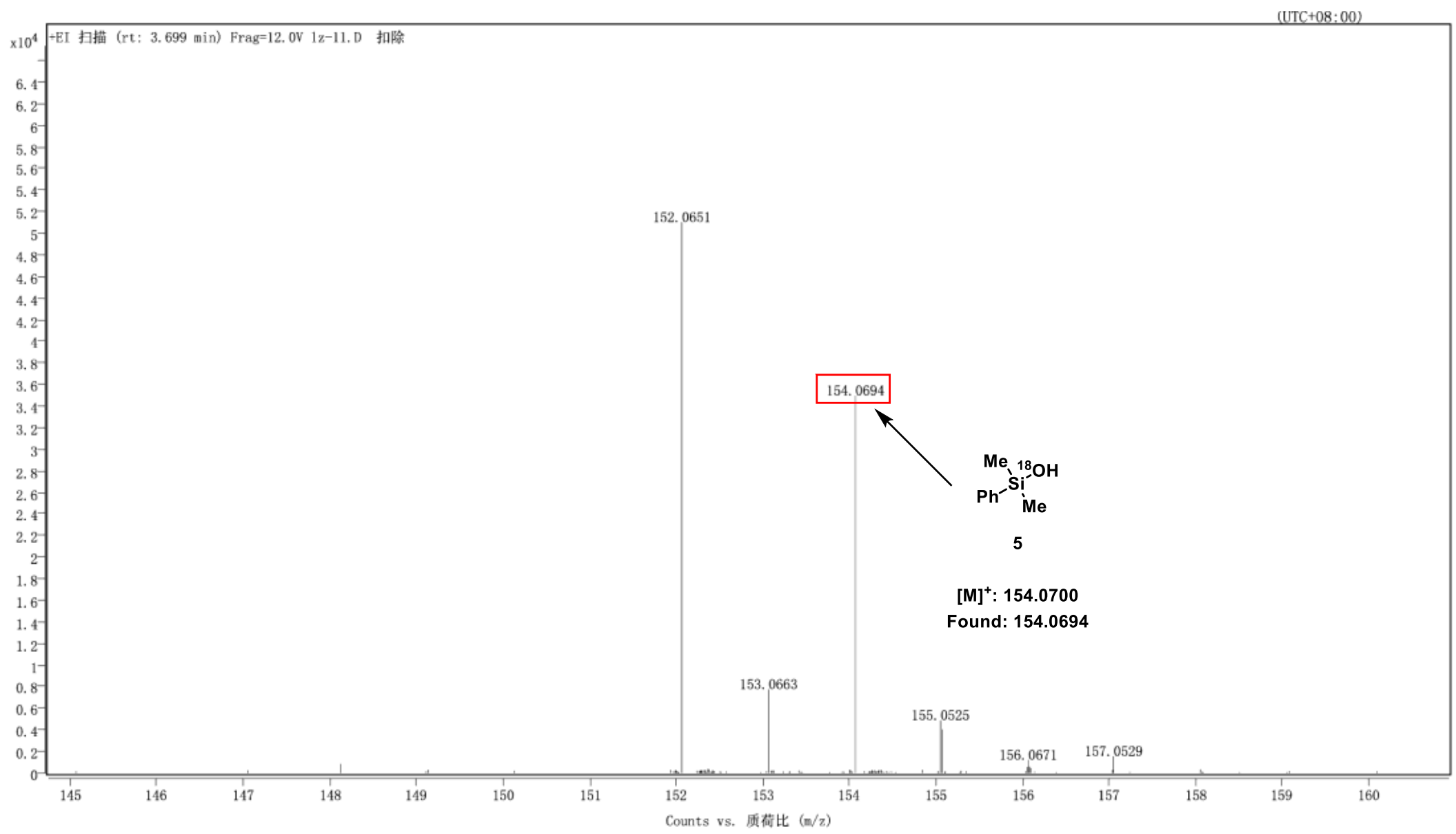


**Figure S8.** HRMS spectrum of **5**

## 9.2 Control experiment

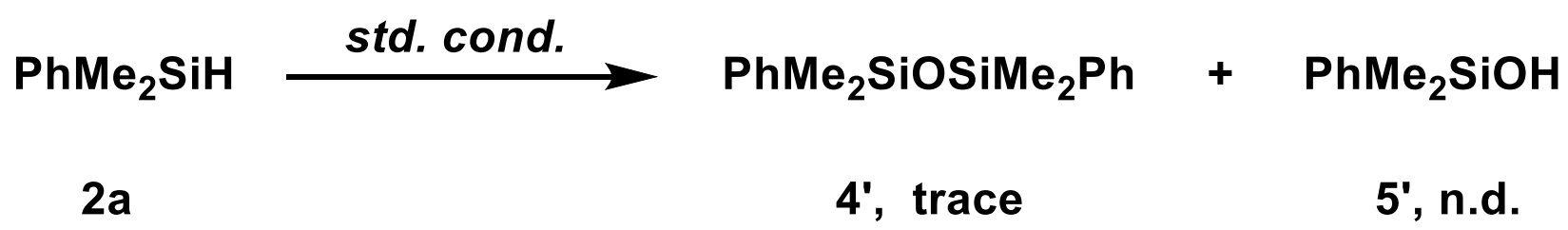


$BaTiO_3$ (350 mg, 1.5 mmol), KO*t*Bu (10 mg, 0.09 mmol) were placed in a 10.0 mL stainless-steel milling jar, along with two stainless-steel balls (10 mm diameter). After adding $PhMe_2SiH$ (**2a**, 82 mg, 0.6 mmol) to the solid mixture, the jar was sealed and subjected to milling at 30 Hz under air. After grinding for 2 h, the reaction mixture was washed with ethyl acetate. **4'** was determined by GC-MS.

## 10. Hydrogen source control experiments

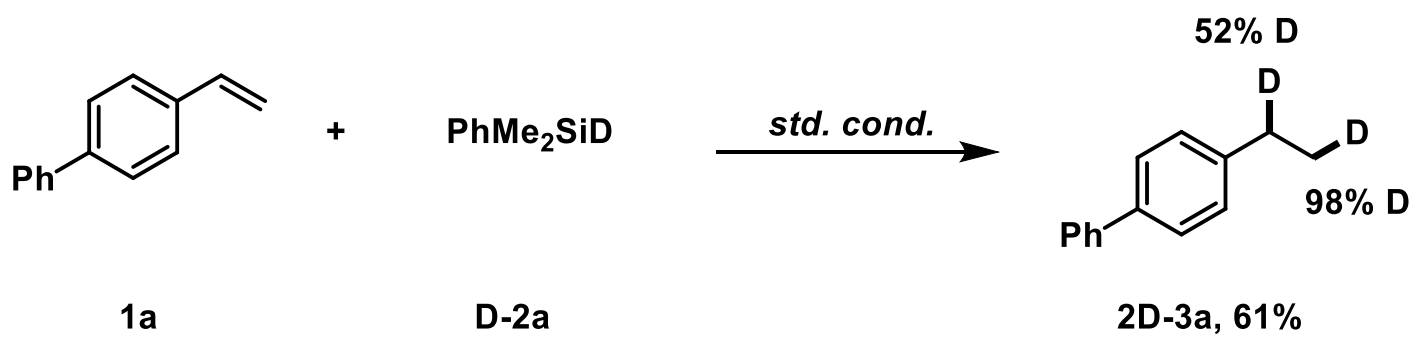


$BaTiO_3$ (350 mg, 1.5 mmol), KO*t*Bu (10 mg, 0.09 mmol) and *p*-vinylbiphenyl (**1a**, 54 mg, 0.3 mmol) were placed in a 10.0 mL stainless-steel milling jar, along with two stainless-steel balls (10 mm diameter). After adding $PhMe_2SiD$ (**D-2a**, 82 mg, 0.6 mmol) to the solid mixture, the jar was sealed and subjected to milling at 30 Hz under air. After grinding for 2 h, the reaction mixture was washed with ethyl acetate. The yield of **2D-3a** was determined by GC analysis using dodecane as an internal standard. **2D-3a**: $^1$H NMR (400 MHz, Chloroform-*d*) δ 7.55 – 7.48 (m, 2H), 7.47 – 7.40 (m, 2H), 7.25 – 7.17 (m, 2H), 7.26 – 7.16 (m, 3H), 2.65 – 2.57 (m, 1H), 1.22 – 1.16 (m, 2H). $^{13}$C NMR (101 MHz, Chloroform-*d*) δ 143.5, 141.3, 138.7, 128.8, 128.4, 127.2, 127.1, 127.1, 28.6, 28.5, 28.5, 15.7.

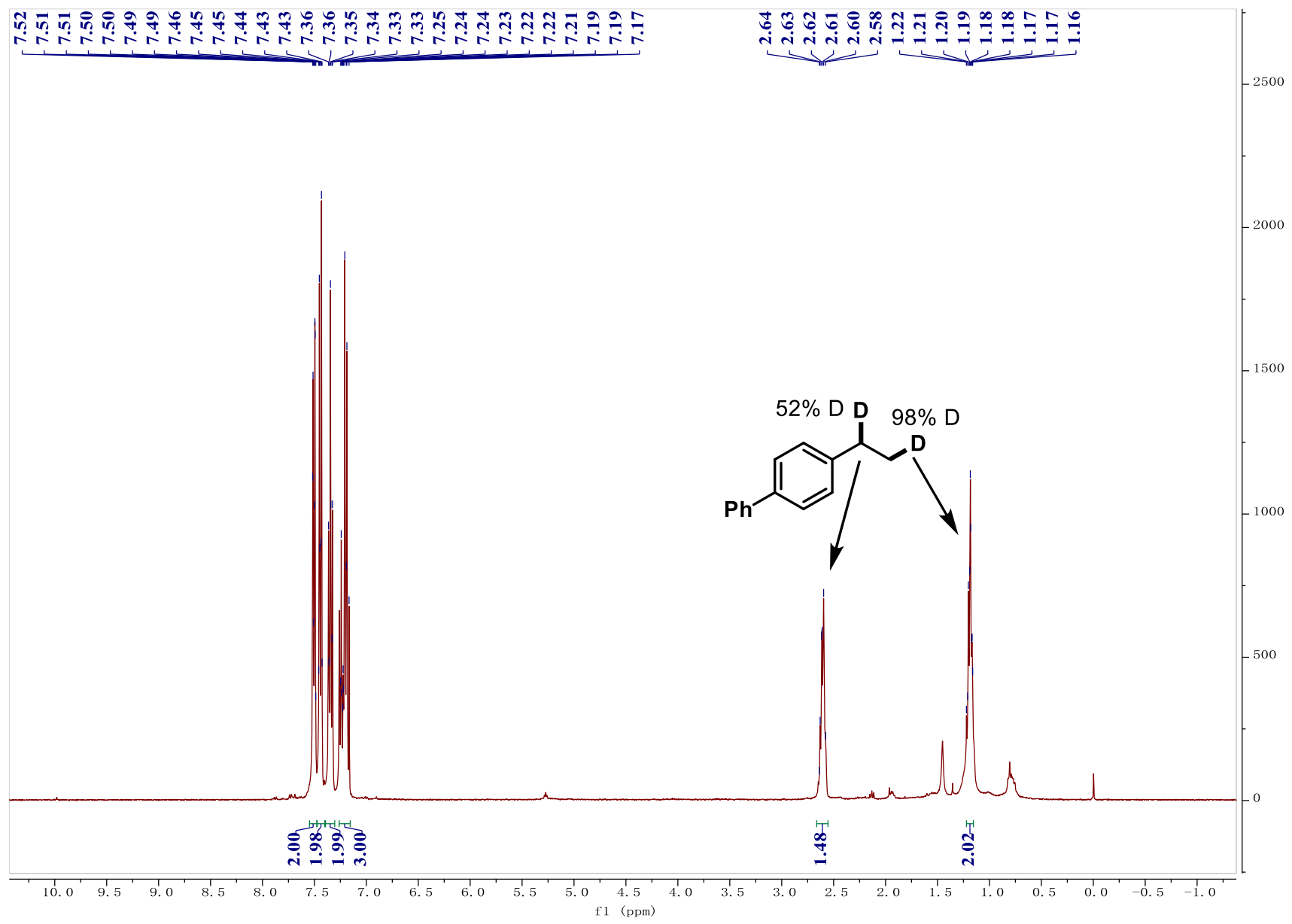


**Figure S9.** $^1$H NMR (400 MHz, $CDCl_3$) spectrum of compound **2D-3a**

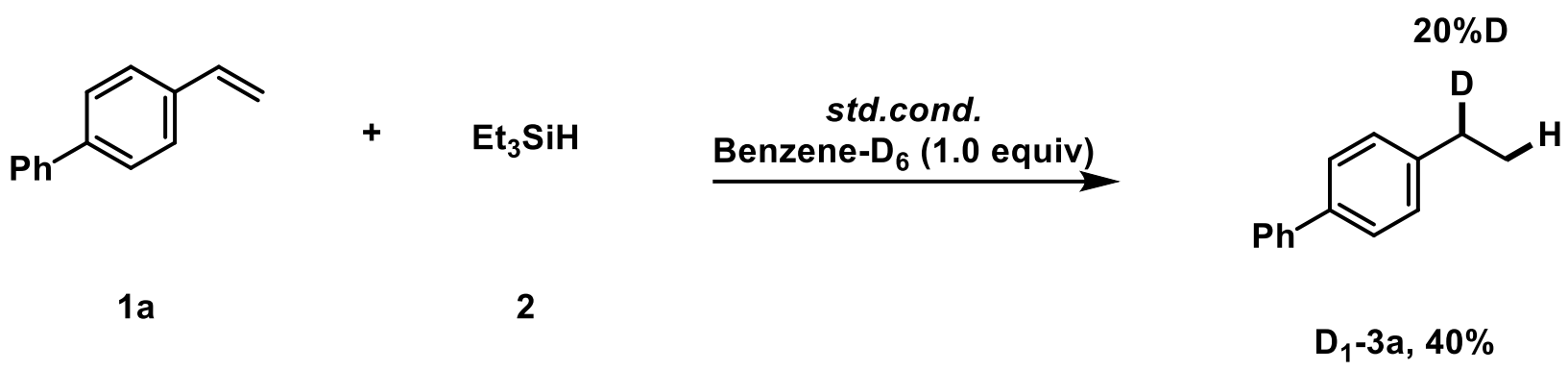


$BaTiO_3$ (350 mg, 1.5 mmol), KO*t*Bu (10 mg, 0.09 mmol) and *p*-vinylbiphenyl (**1a**, 54 mg, 0.3 mmol) were placed in a 10.0 mL stainless-steel milling jar, along with two stainless-steel balls (10 mm diameter). After adding triethylsilane (**2**, 95 µL, 0.6 mmol) and benzene-D$_6$ (25 mg, 0.3 mmol) to the solid mixture, the jar was sealed and subjected to milling at 30 Hz under air. After grinding for 2 h, the reaction mixture was washed with ethyl acetate. The yield of **D$_1$-3a** was determined by GC analysis using dodecane as an internal standard.

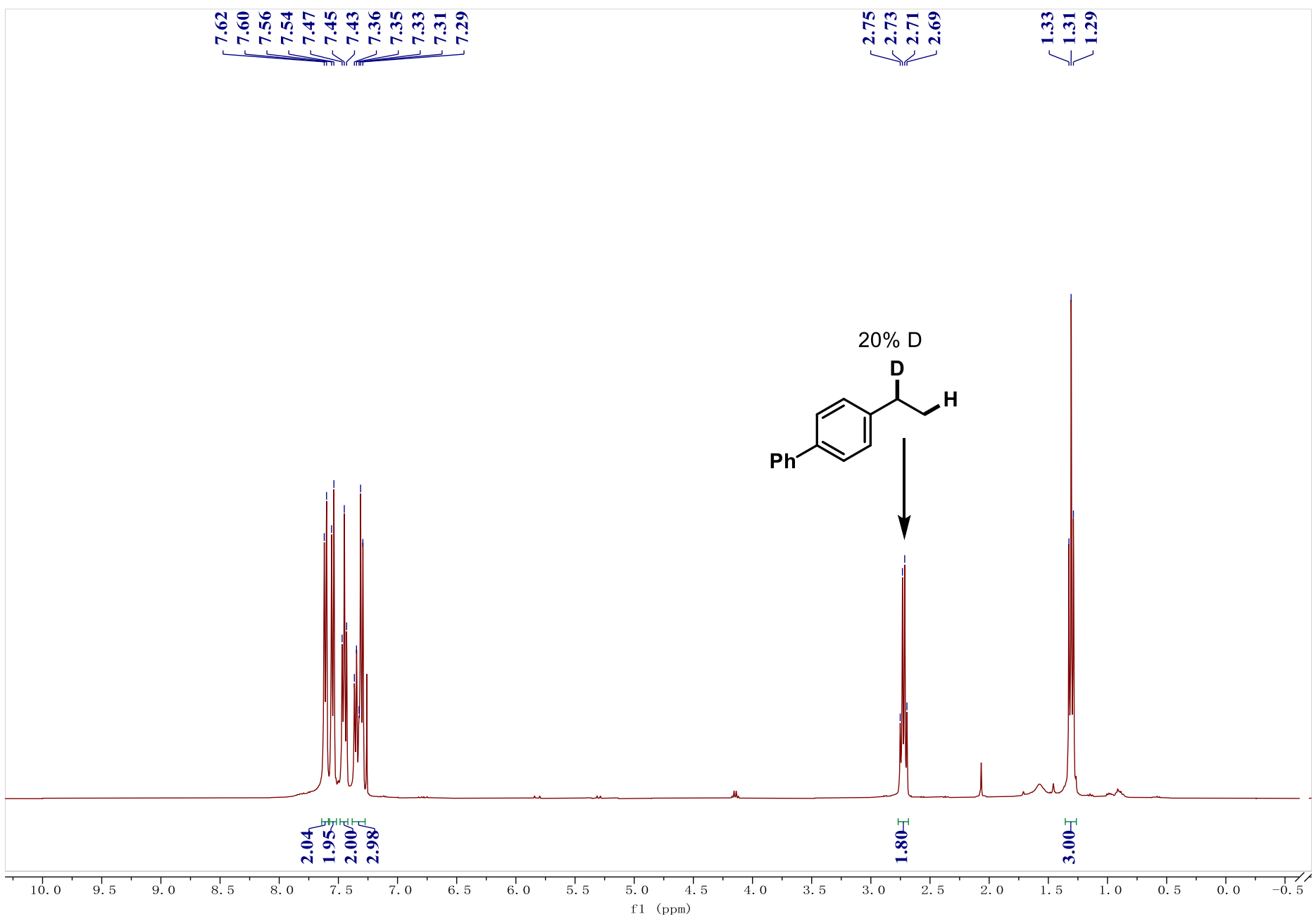


**Figure S10.** $^1$H NMR (400 MHz, $CDCl_3$) spectrum of compound **D$_1$-3a**

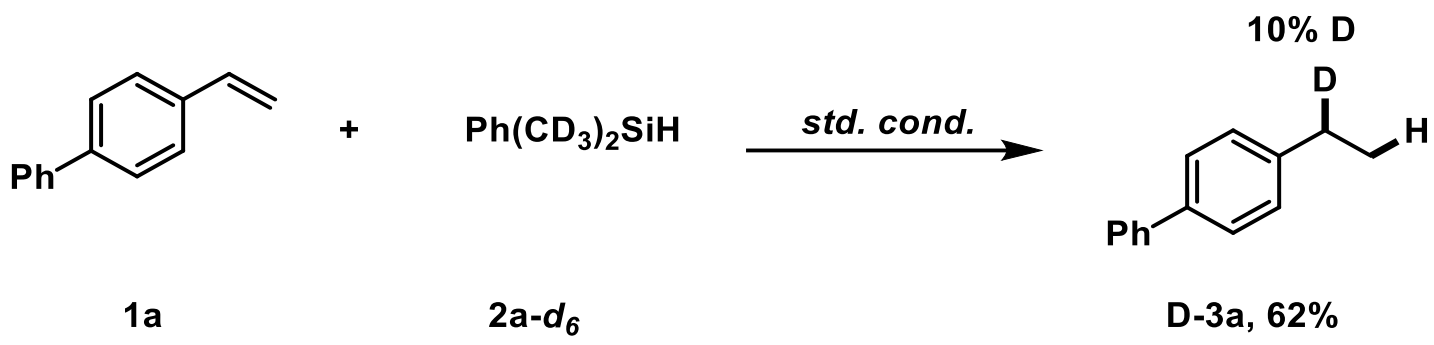


$BaTiO_3$ (350mg, 1.5 mmol), KO*t*Bu (10 mg, 0.09 mmol) and *p*-vinylbiphenyl (**1a**, 54 mg, 0.3 mmol) were placed in a 10.0 mL stainless-steel milling jar, along with two stainless-steel balls (10 mm diameter). After adding $Ph(CD_3)_2SiH$ (**2a-*d*$_6$**, 82 mg, 0.6 mmol) to the solid mixture, the jar was sealed and subjected to milling at 30 Hz under air. After grinding for 2 h, the reaction mixture was washed with ethyl acetate. The yield of **D-3a** was determined by GC analysis using dodecane as an internal standard.

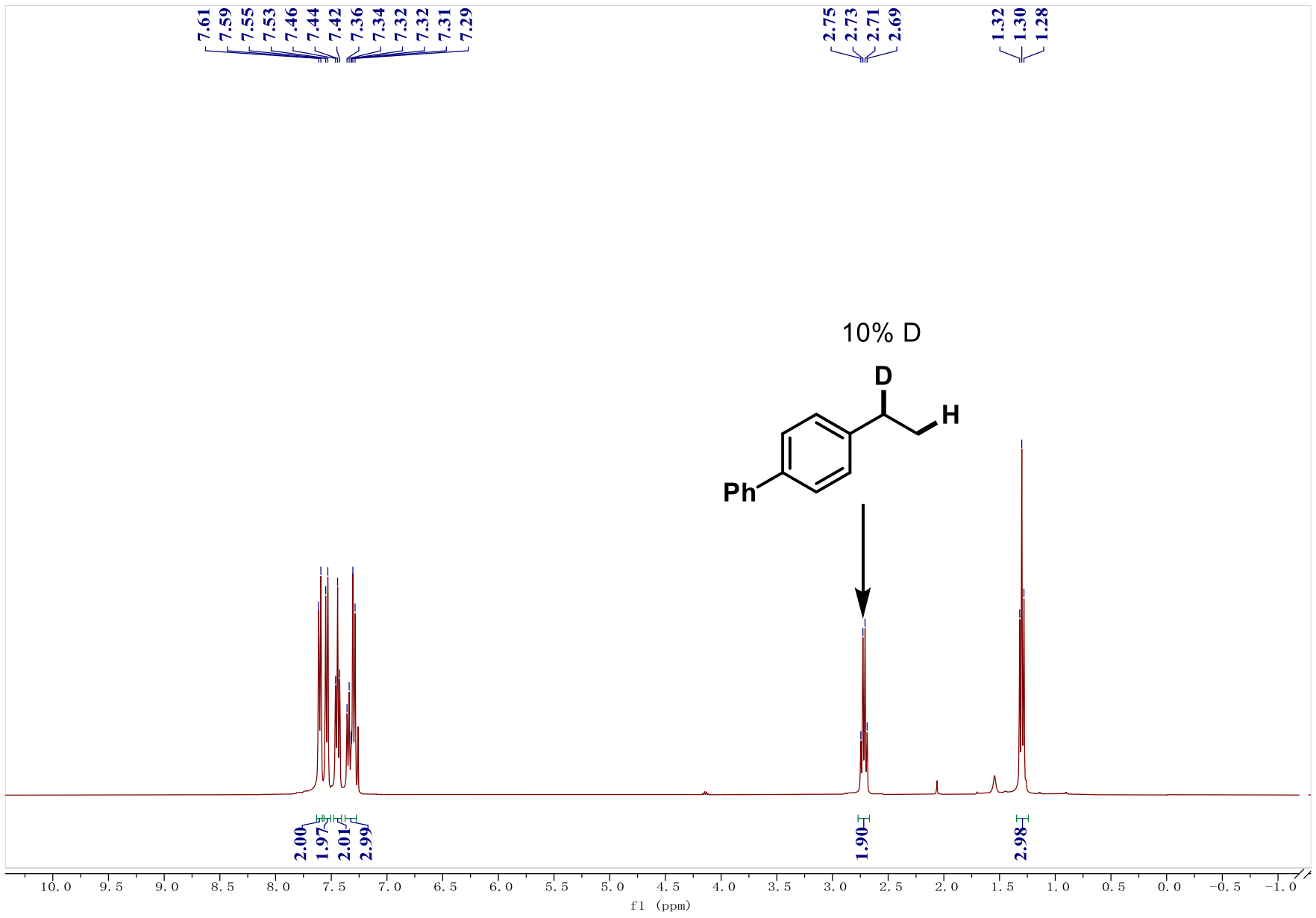

**Figure S11.** $^1H$ NMR (400 MHz, $CDCl_3$) spectrum of compound **D-3a**

## 11. Radical-trapping experiments

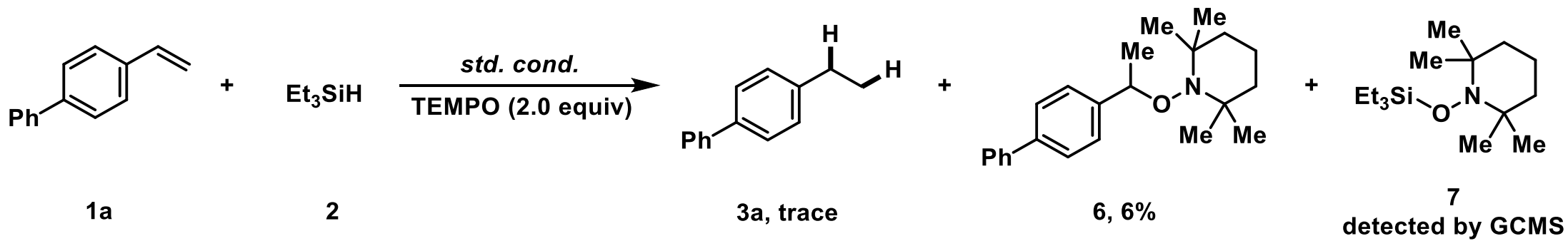


$BaTiO_3$ (350 mg, 1.5 mmol), KO*t*Bu (10 mg, 0.09 mmol), *p*-vinylbiphenyl (**1a**, 54 mg, 0.3 mmol) and 2,2,6,6-tetramethylpiperidinyloxy (TEMPO, 0.6 mmol) were placed in a 10.0 mL stainless-steel milling jar, along with two stainless-steel balls (10 mm diameter). After adding triethylsilane (**2**, 95 μL, 0.6 mmol) to the solid mixture, the jar was sealed and subjected to milling at 30 Hz under air. After grinding for 2 h, the reaction mixture was washed with ethyl acetate. The yield of hydrogenation product **3a** was determined by GC analysis using dodecane as an internal standard. The benzyl radical combined with TEMPO (**6**) was isolated as a white solid using petroleum/ethyl acetate (50:1) as eluent (6 mg, 6%). $^1H$ NMR (400 MHz, Chloroform-*d*) δ 7.62 (d, $J$ = 8.0 Hz, 2H), 7.56 (d, $J$ = 6.8 Hz, 2H), 7.47 – 7.38 (m, 4H), 7.36 – 7.31 (m, 1H), 4.85 (q, $J$ = 6.8 Hz, 1H), 1.53 (d,

$J$ = 6.8 Hz, 6H), 1.40 – 1.27 (m, 6H), 1.19 (s, 3H), 1.07 (s, 3H), 0.73 (s, 3H). $^{13}$C NMR (101 MHz, Chloroform-*d*) δ 145.0, 141.2, 139.7, 128.8, 127.2, 127.1, 126.9, 82.9, 59.8, 40.5, 23.7, 20.5, 17.3. The triethylsilyl radical combined with TEMPO (**7**) was detected by GCMS.

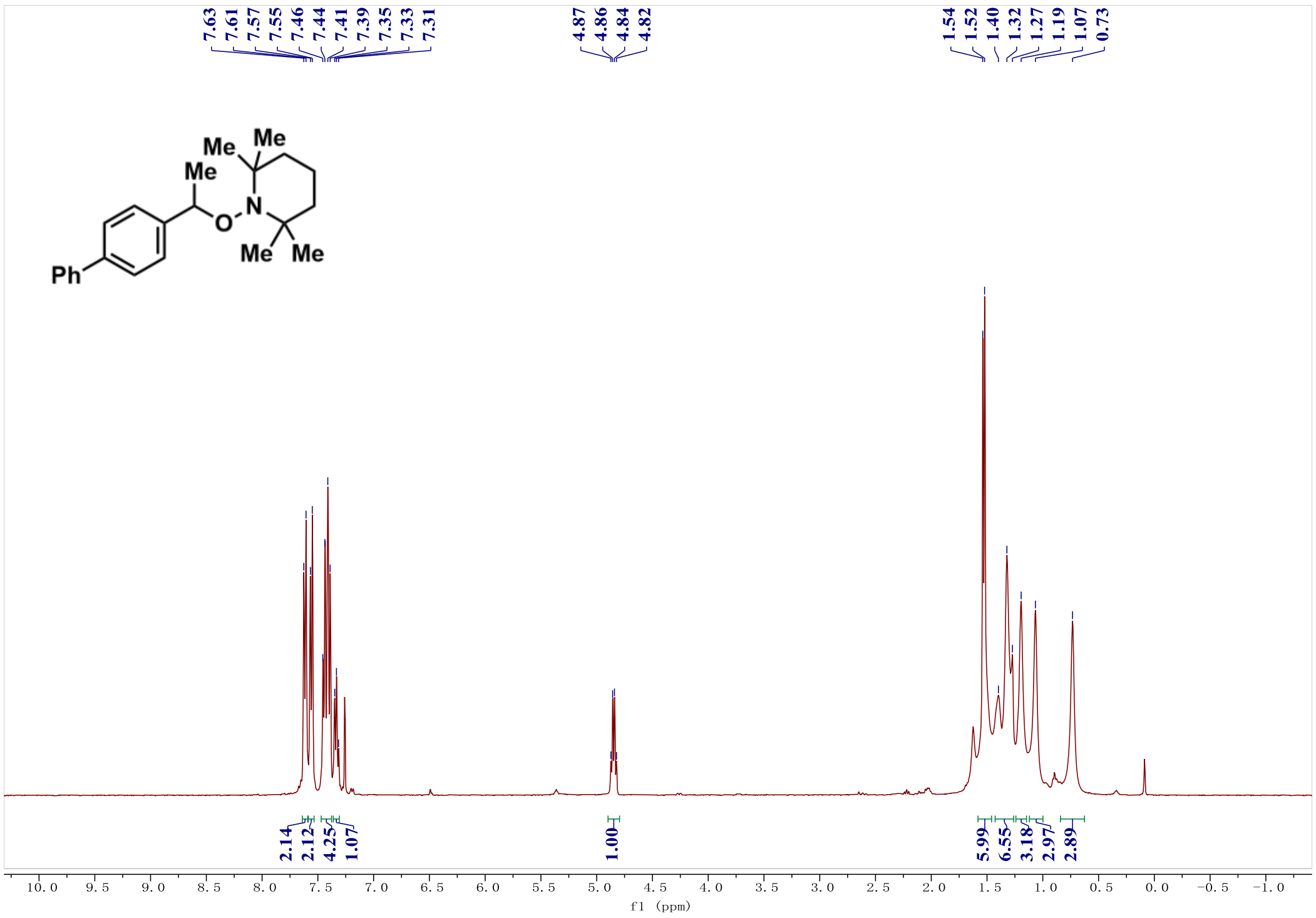

**Figure S12.** $^1$H NMR (400 MHz, $CDCl_3$) spectrum of compound **6**

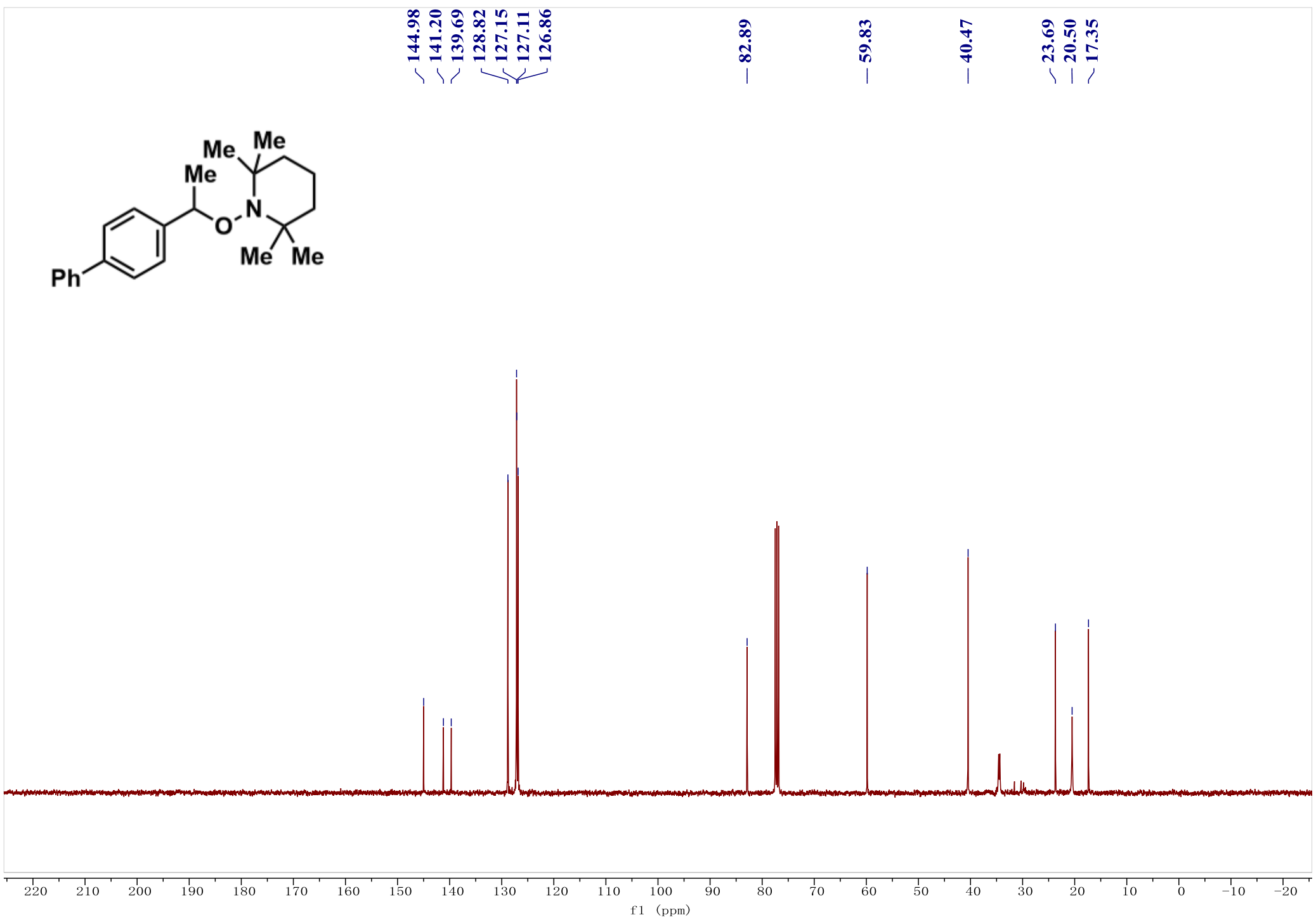

**Figure S13.** $^{13}$C NMR (101 MHz, $CDCl_3$) spectrum of compound **6**

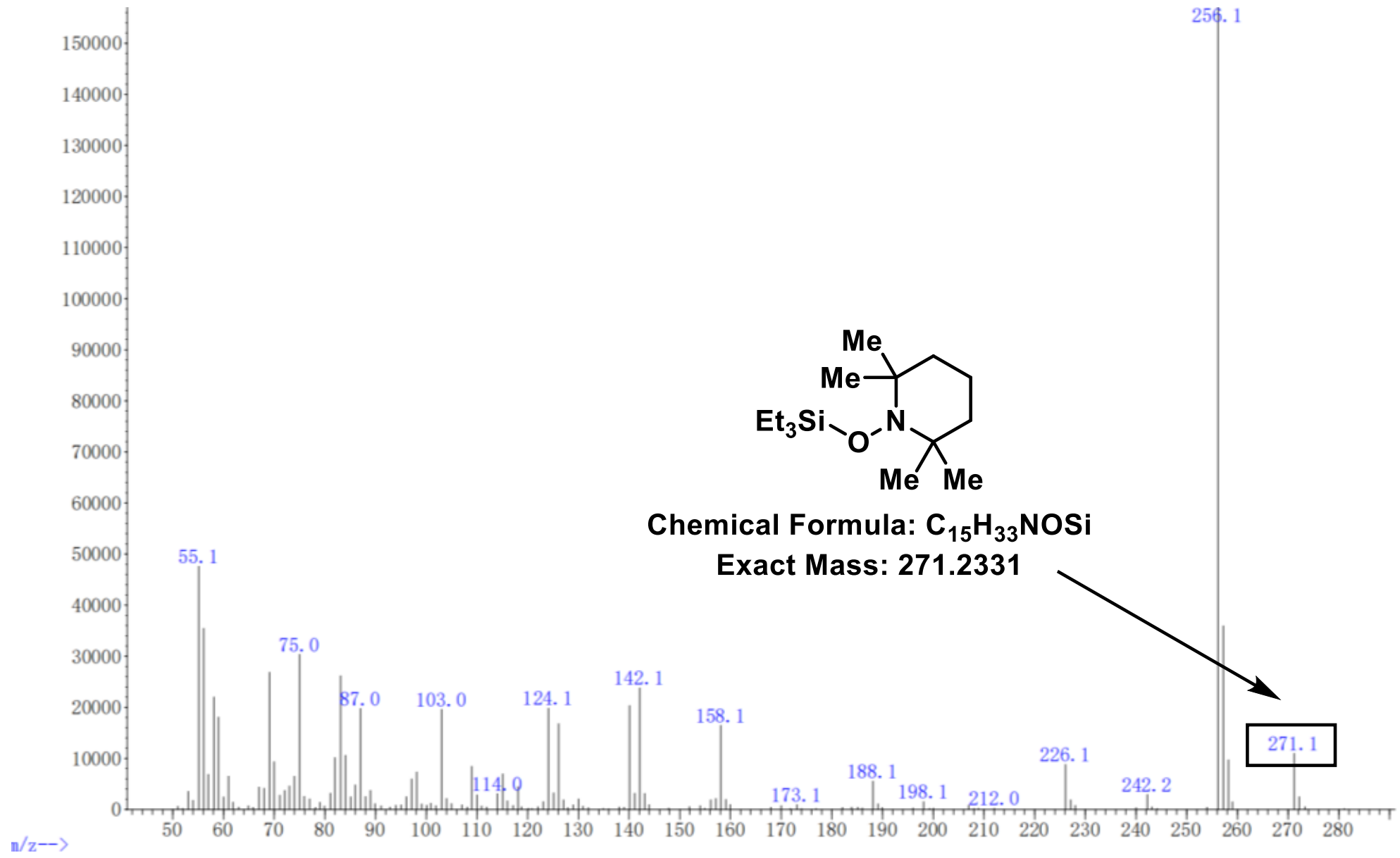


**Figure S14.** GCMS spectrum of **7**

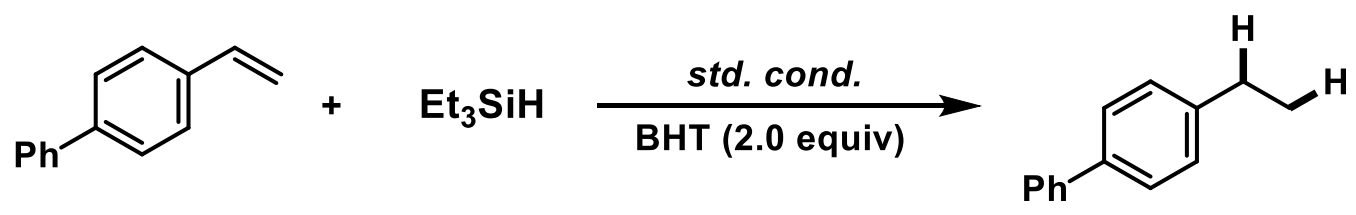


**1a** **2** **3a, trace**

$BaTiO_3$ (350 mg, 1.5 mmol), KO*t*Bu (10 mg, 0.09 mmol), *p*-vinylbiphenyl (**1a**, 54 mg, 0.3 mmol) and butylated hydroxytoluene (BHT, 0.6 mmol) were placed in a 10.0 mL stainless-steel milling jar, along with two stainless-steel balls (10 mm diameter). After adding triethylsilane (**2**, 95 μL, 0.6 mmol) to the solid mixture, the jar was sealed and subjected to milling at 30 Hz under air. After grinding for 2 h, the reaction mixture was washed with ethyl acetate. The yield of hydrogenation product **3a** was determined by GC analysis using dodecane as an internal standard.

## 12. XRD patterns of $BaTiO_3$

The sample of $BaTiO_3$ after ball mill was prepared at 30 Hz under air in a stainless-steel milling jar (10.0 mL) with two stainless-steel balls (10 mm, diameter). The images were shown in Figure S15.

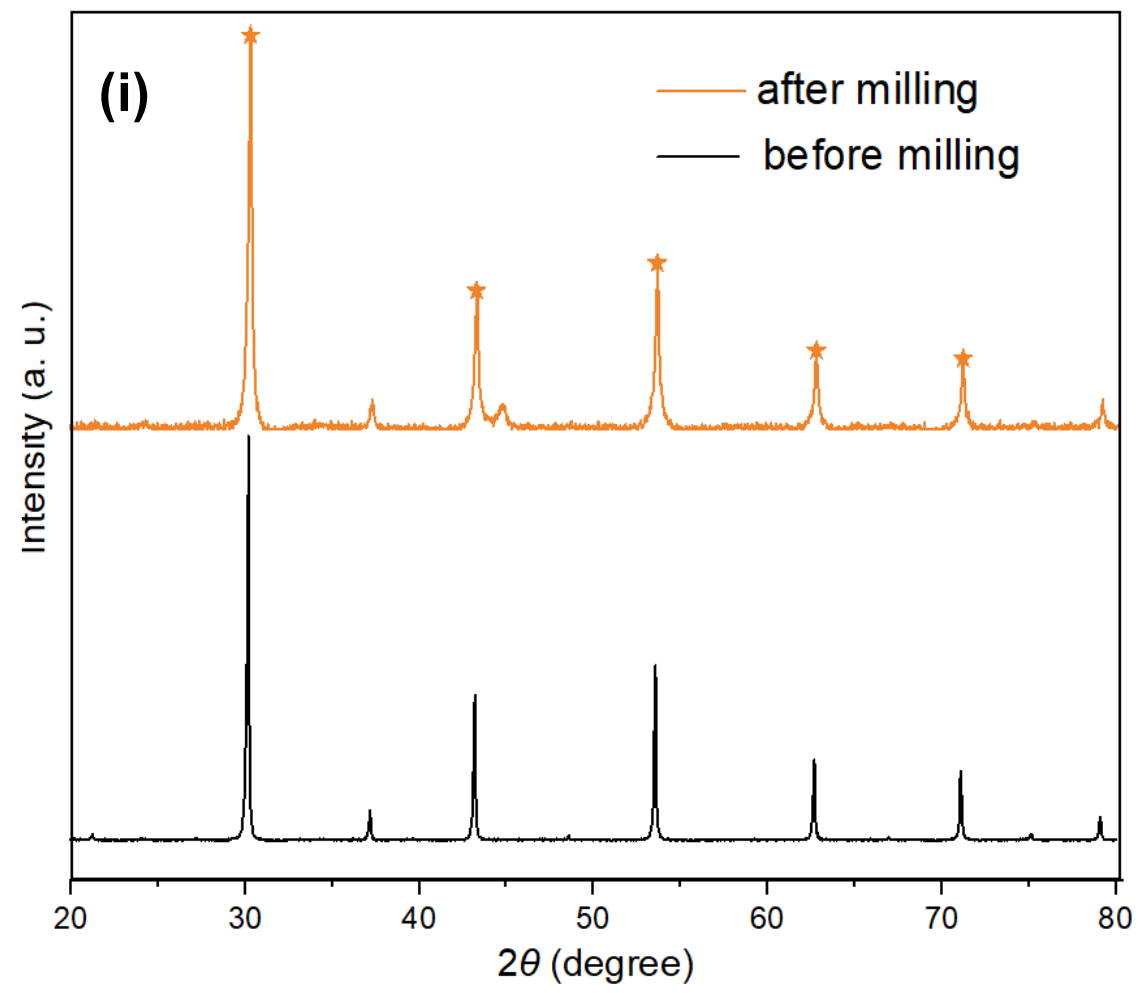


**Figure S15.** XRD images of $BaTiO_3$ particles before ball milling and after ball milling

## 13. Characterization of $BaTiO_3$ particles by SEM

The sample of $BaTiO_3$ after ball mill was prepared by at 30 Hz under air in a stainless-steel milling jar (10.0 mL) with two stainless-steel balls (10 mm, diameter). Additional images were shown in Figure S16.

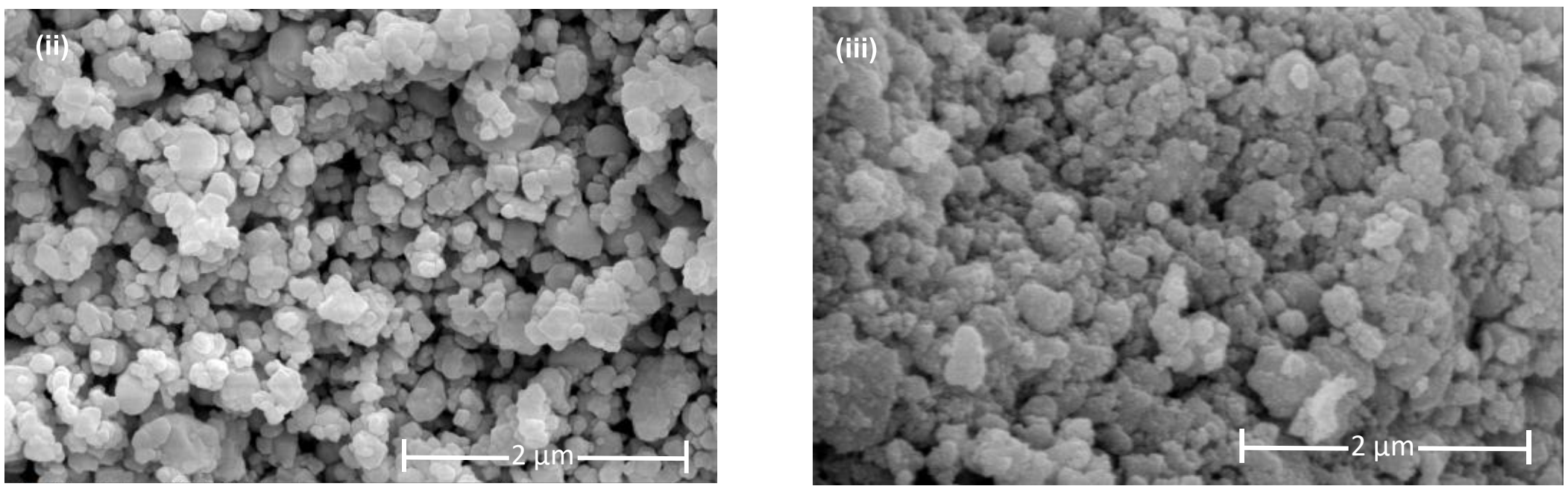


**Figure S16.** SEM images of $BaTiO_3$ particles before ball milling (ii) and after ball milling (iii)

## 14. References


[1] T. Vom Stein, M. Peréz, R. Dobrovetsky, D. Winkelhaus, C. B. Caputo, D. W. Stephan, Electrophilic fluorophosphonium cations in frustrated Lewis pair hydrogen activation and catalytic hydrogenation of olefins. *Angew. Chem. Int. Ed.* **2015**, *54*, 10178-10182.

[2] Y. Wang, P. Bao, X. Dong, Y. Lan, Y. Chen, Thiophenol-Catalyzed Radical Hydroformylation of Unactivated Sterically Hindered Alkenes. *J. Am. Chem. Soc.* **2025**, *147*, 31662–31670.

[3] F. You, X. Zhang, X. Wang, G. Guo, Q. Wang, H. Song, R. Qu, Z. Lian, Mechanochemical vicinal dibromination of unactivated alkenes and alkynes using piezoelectric material as a redox catalyst. *Org. Lett.* **2024**, *26*, 4240-4245.

[4] J. Waser, H. Nambu, E. M. Carreira, Cobalt-catalyzed hydroazidation of olefins: convenient access to alkyl azides. *J. Am. Chem. Soc.* **2005**, *127*, 8294-8295.

[5] H. Shigehisa, T. Aoki, S. Yamaguchi, N. Shimizu, K. Hiroya, Hydroalkoxylation of unactivated olefins with carbon radicals and carbocation species as key intermediates. *J. Am. Chem. Soc.* **2013**, *135*, 10306-10309.

[6] W. Xu, X. Cong, K. An, S. J. Lou, Z. Li, M. Nishiura, T. Murahashi, Z. Hou, Regio-and diastereoselective formal [2+2] cycloaddition of allenes with amino-functionalized alkenes by rare-earth-catalyzed C($sp^2$)-H activation. *Angew. Chem. Int. Ed*. **2022**, *61*, e202210624.

[7] M. Tang, S. Han, S. Huang, S. Huang, L. G. Xie, Carbosulfenylation of alkenes with organozinc reagents and dimethyl (methylthio) sulfonium trifluoromethanesulfonate. *Org. Lett.* **2020**, *22*, 9729-9734.

[8] Y. Zhao, S. Ge, Synergistic hydrocobaltation and borylcobaltation enable regioselective migratory triborylation of unactivated alkenes. *Angew. Chem. Int. Ed*. **2022**, *61*, e202116133.

## 15. NMR spectra

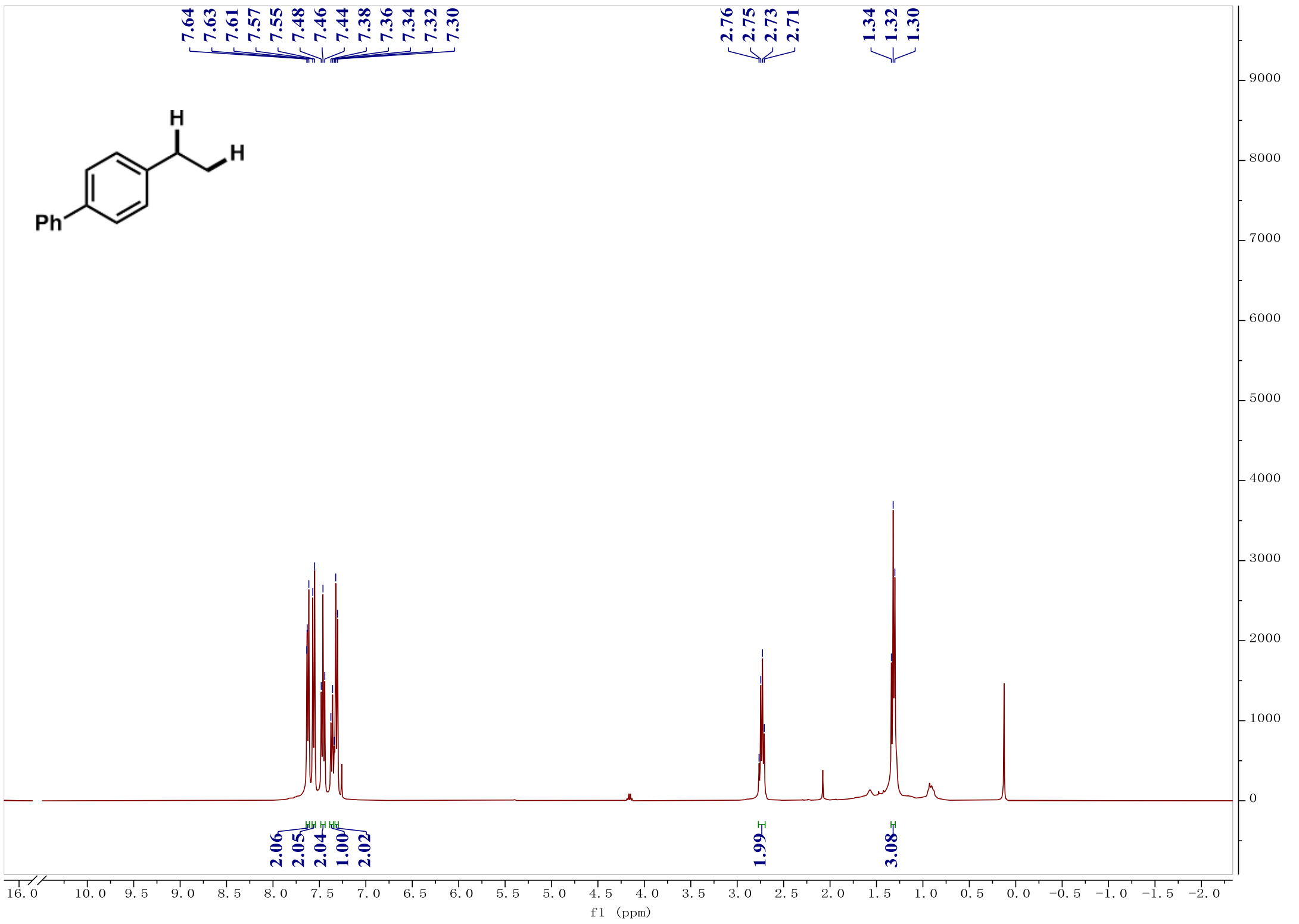

**$^{1}$H NMR** (400 MHz, $CDCl_3$) Spectrum of Compound **3a**

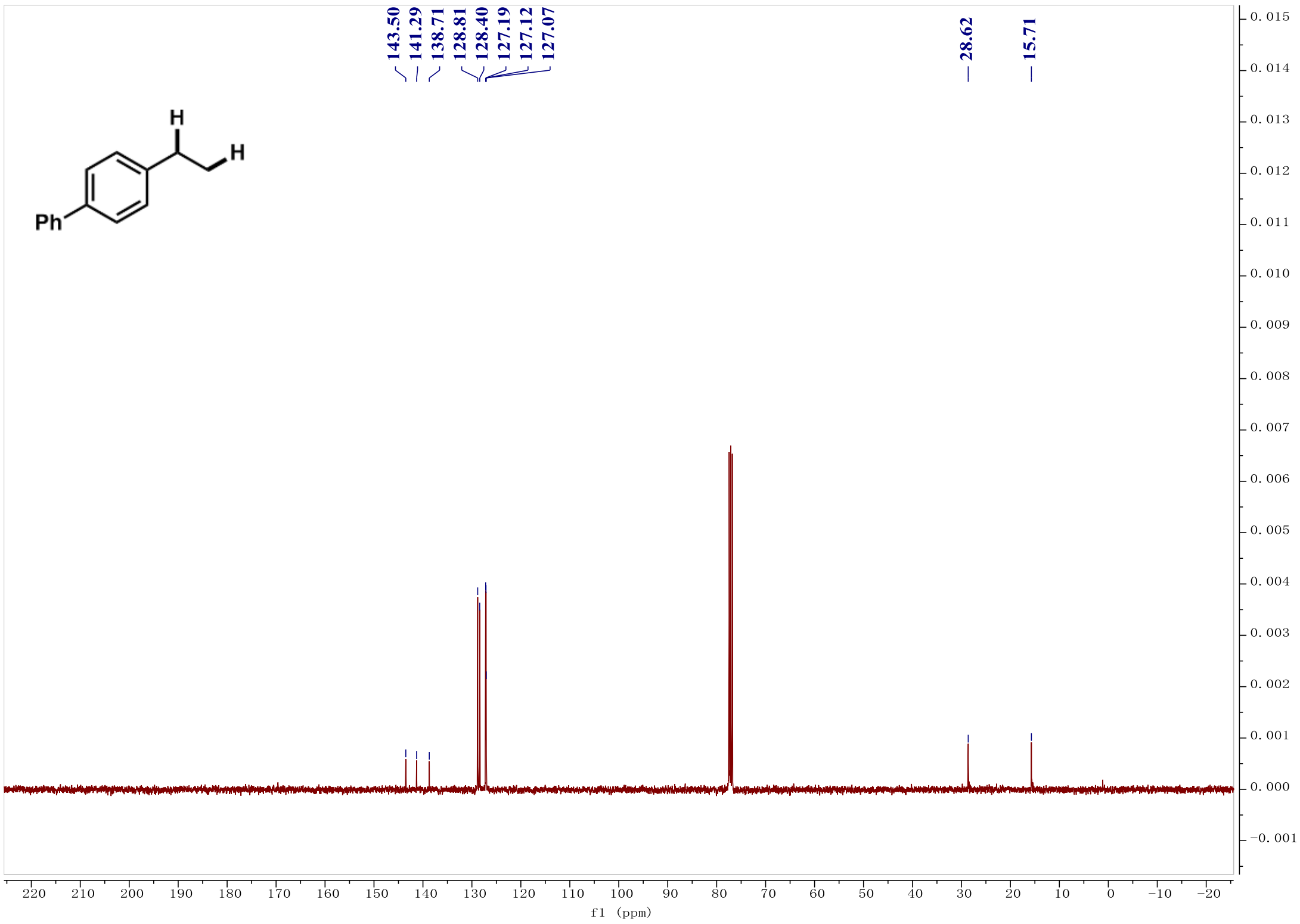

**$^{13}$C NMR** (101 MHz, $CDCl_3$) Spectrum of Compound **3a**

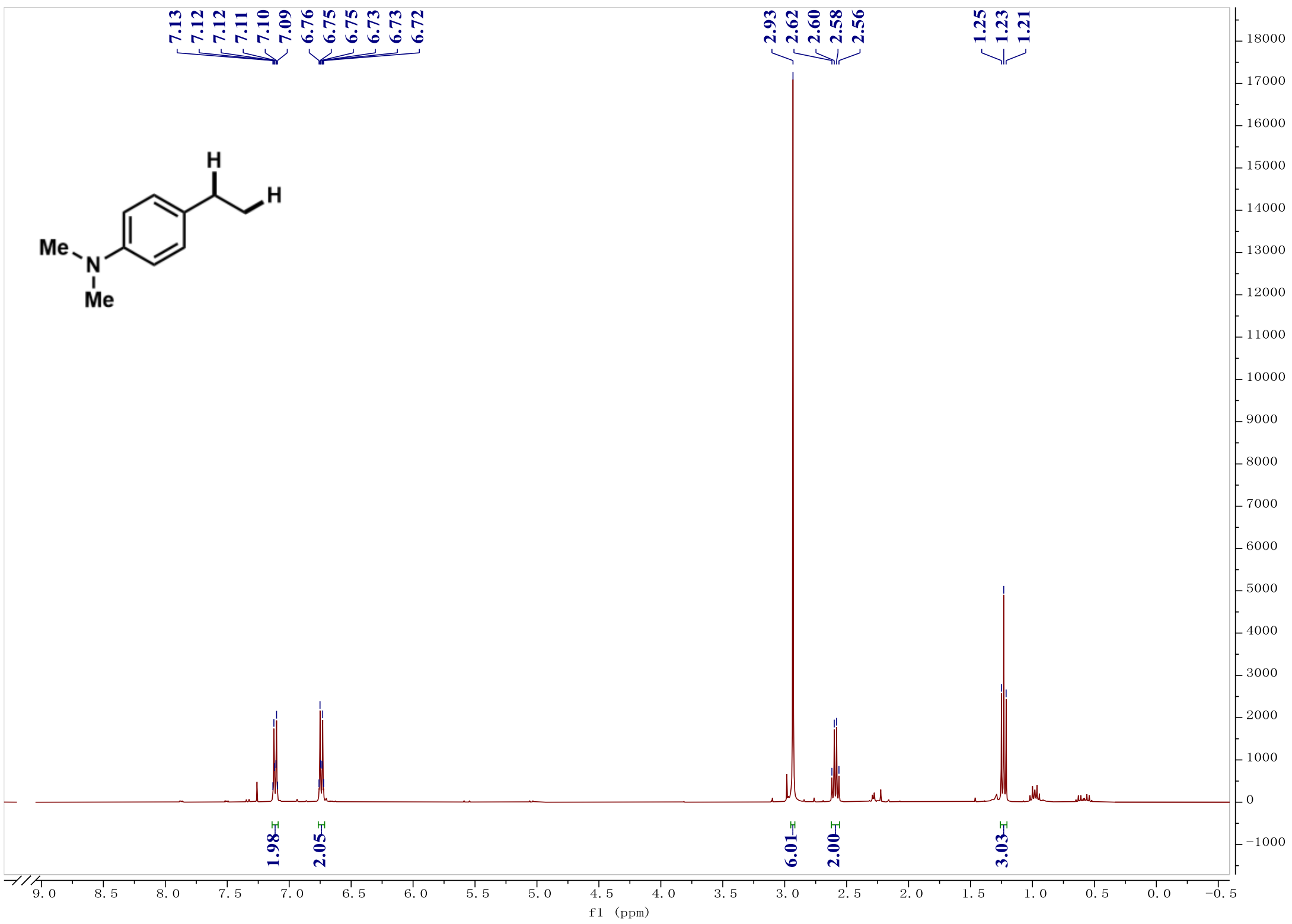


**$^{1}$H NMR** (400 MHz, $CDCl_3$) Spectrum of Compound **3b**

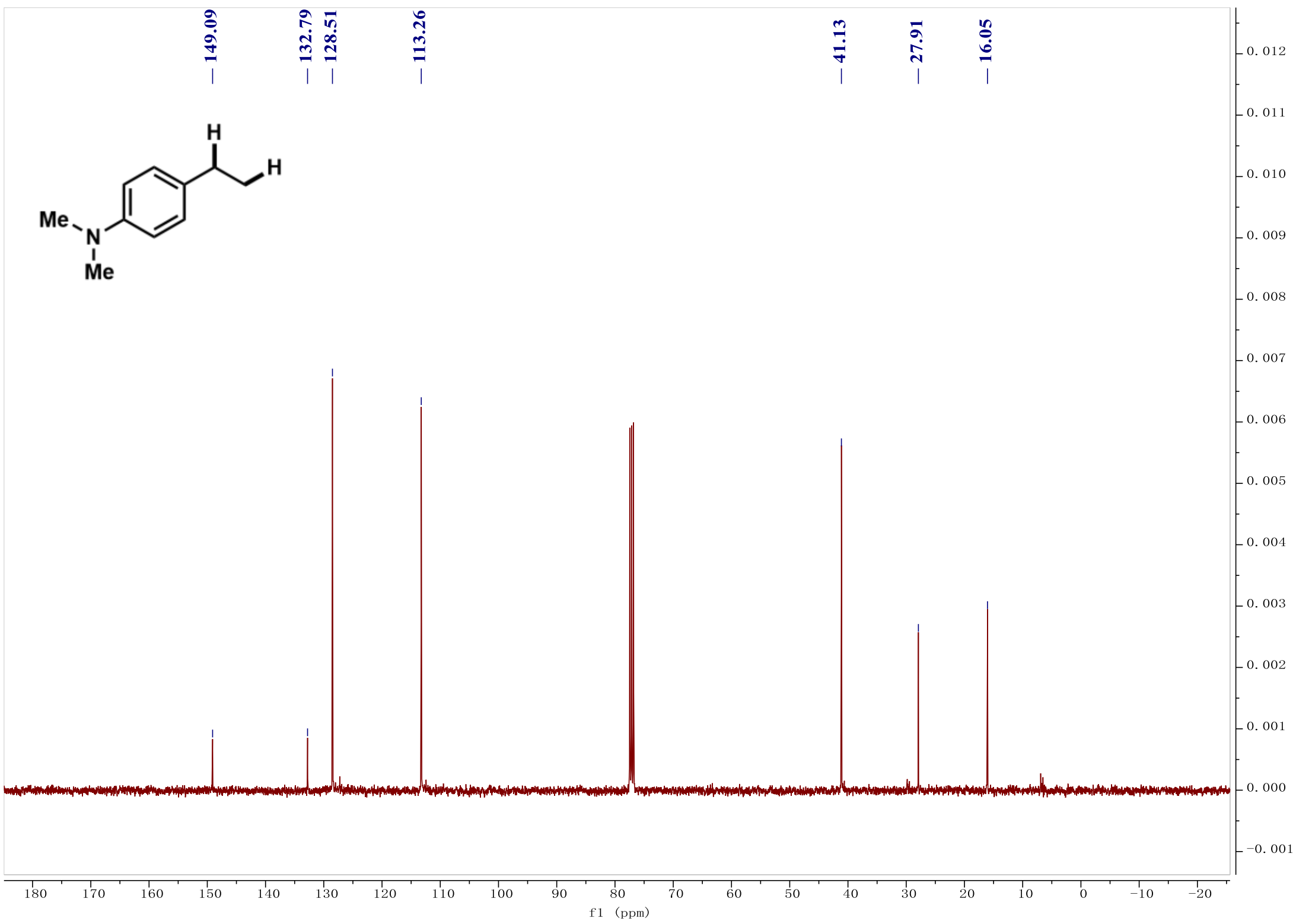


**$^{13}$C NMR** (101 MHz, $CDCl_3$) Spectrum of Compound **3b**

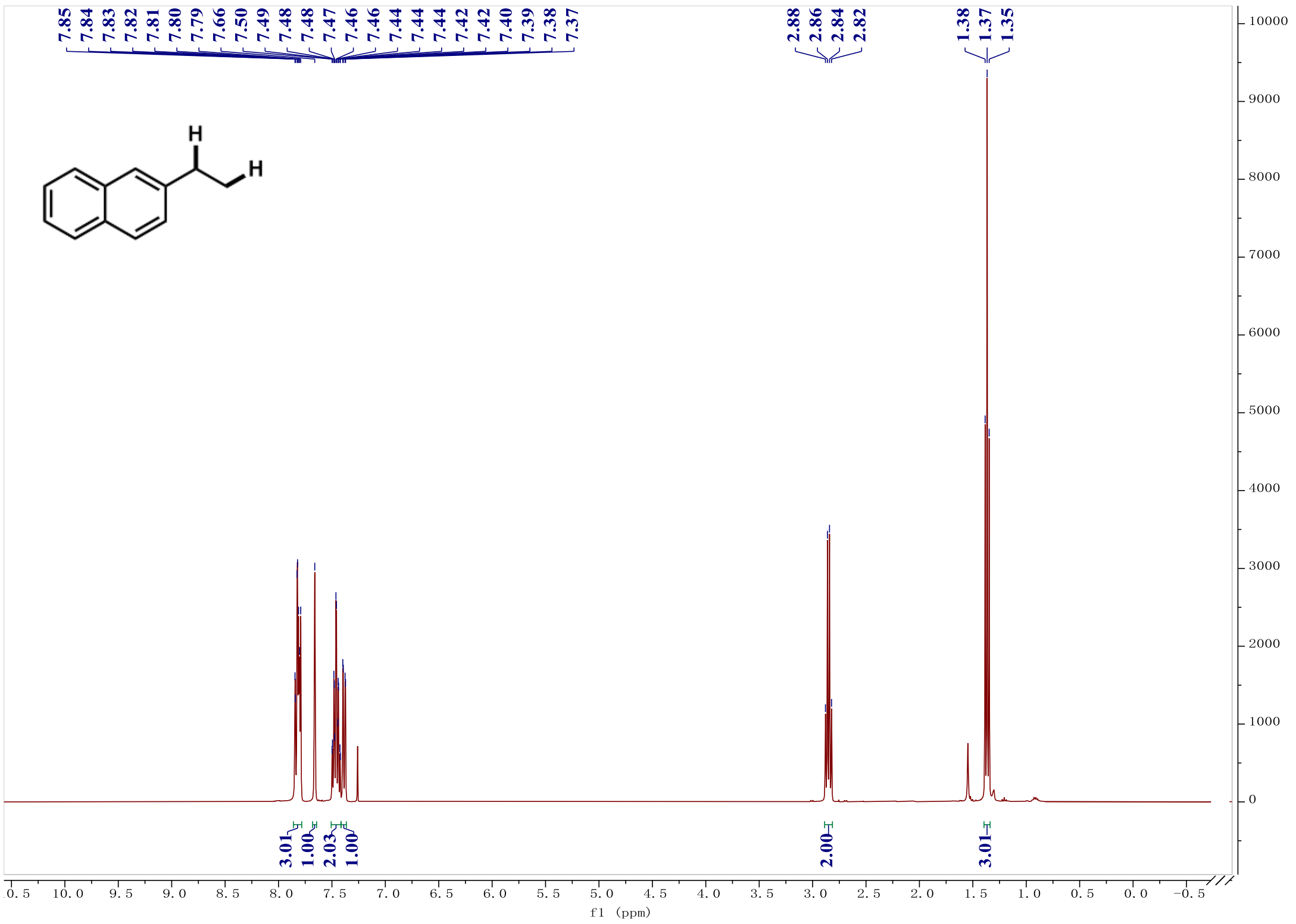


**¹H NMR** (400 MHz, $CDCl_3$) Spectrum of Compound **3c**

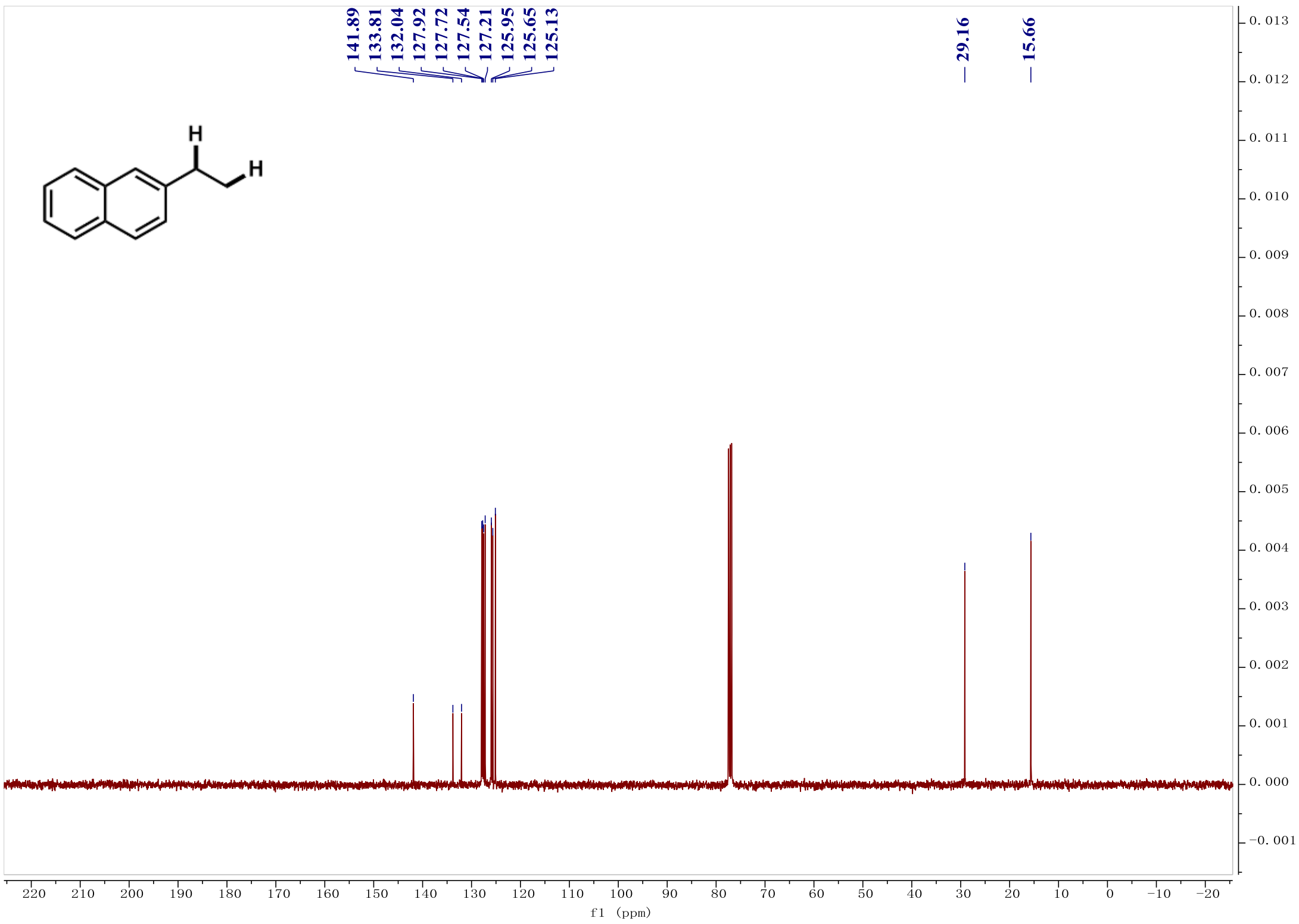


**¹³C NMR** (101 MHz, $CDCl_3$) Spectrum of Compound **3c**

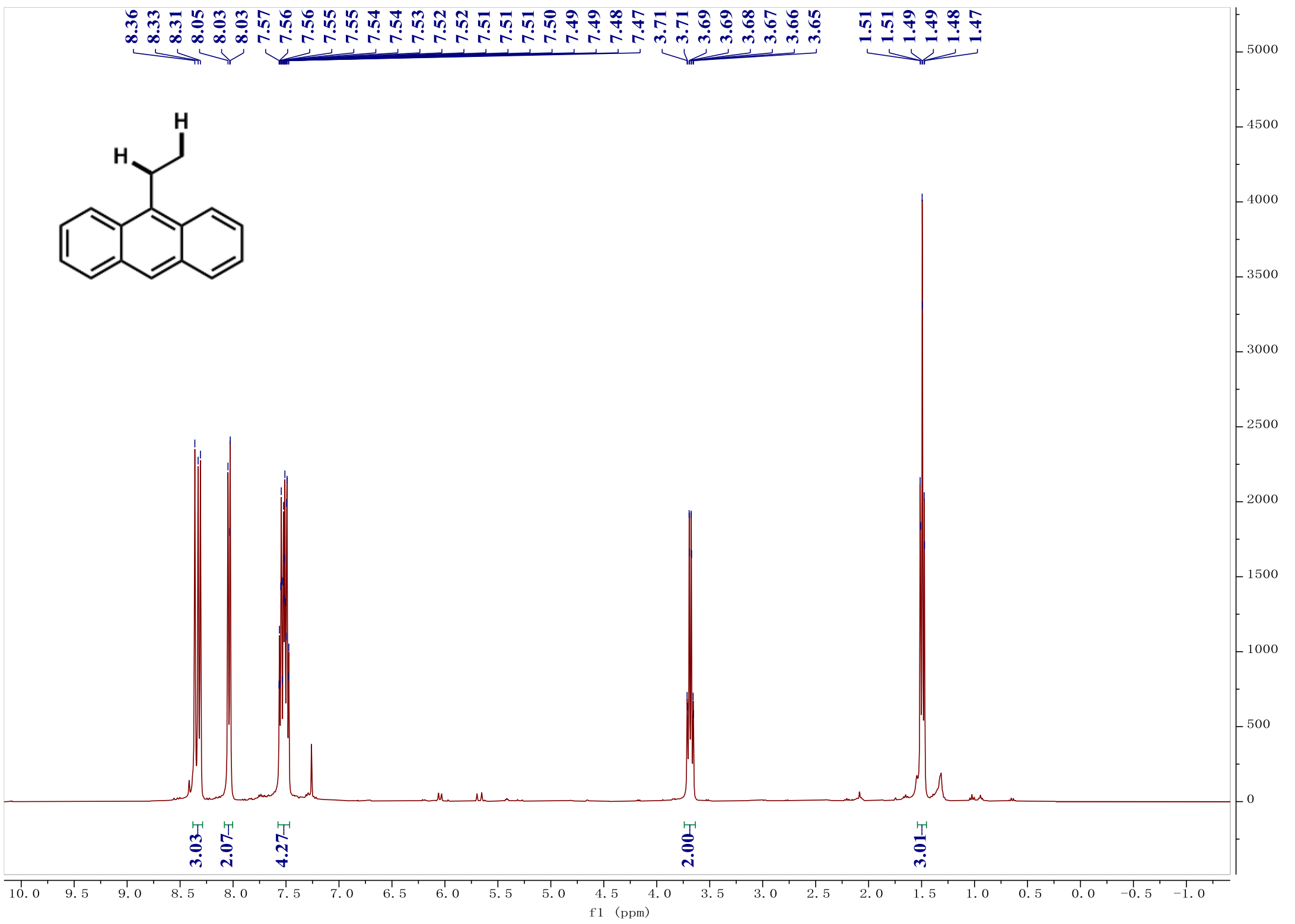

$^{1}$**H NMR** (400 MHz, CDCl$_3$) Spectrum of Compound **3d**

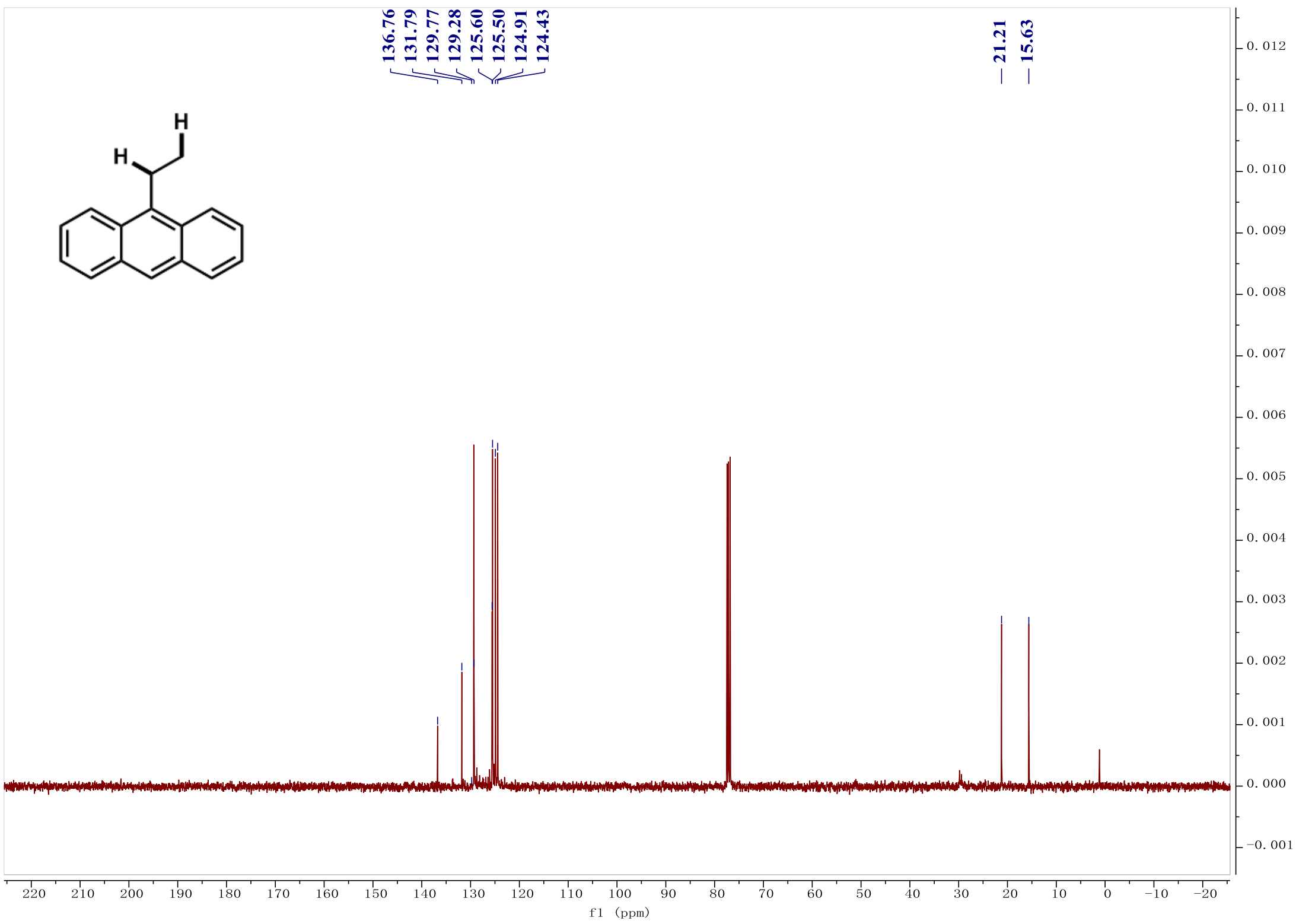

$^{13}$**C NMR** (101 MHz, CDCl$_3$) Spectrum of Compound **3d**

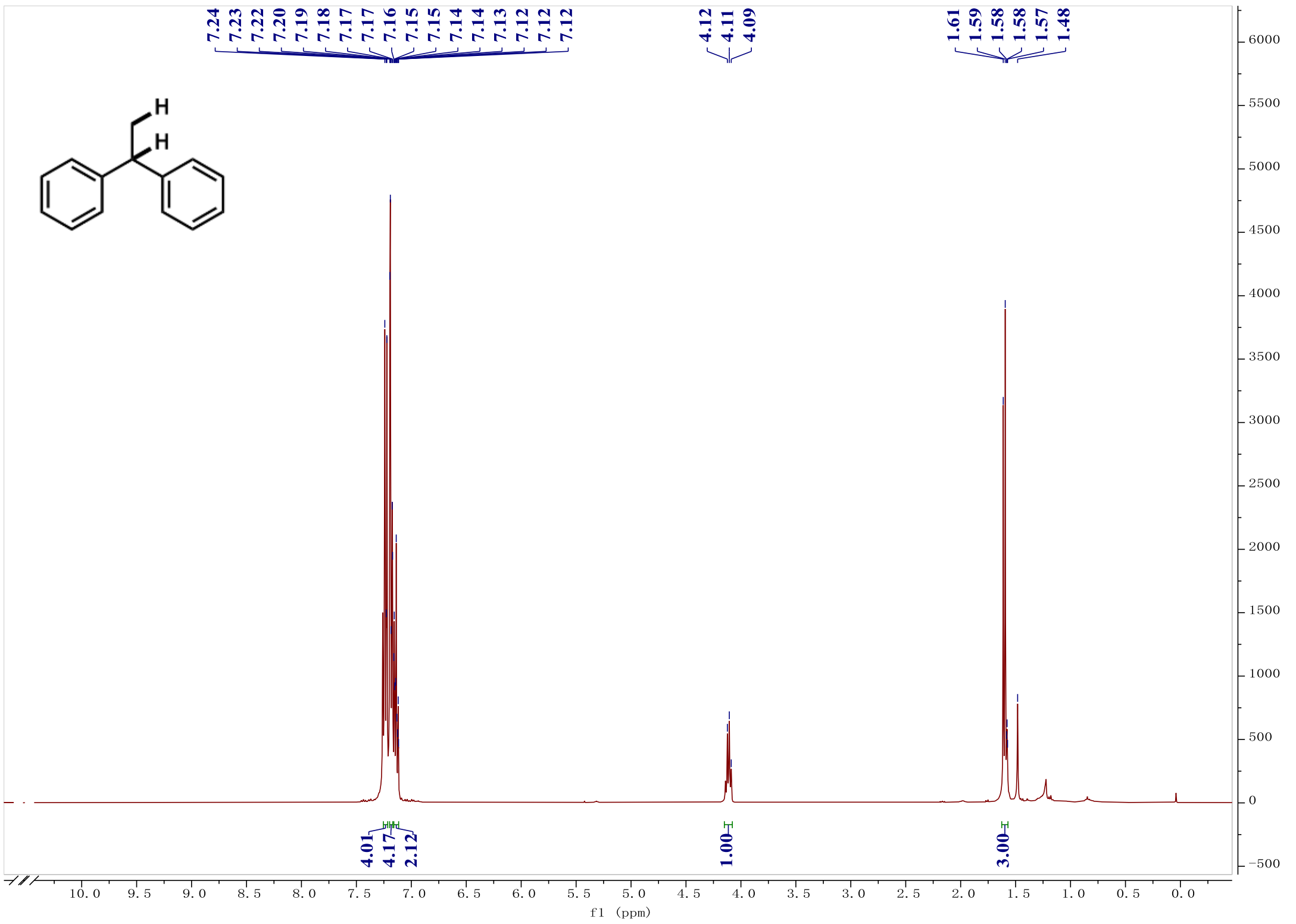

$^1$H NMR (400 MHz, $CDCl_3$) Spectrum of Compound **3e**

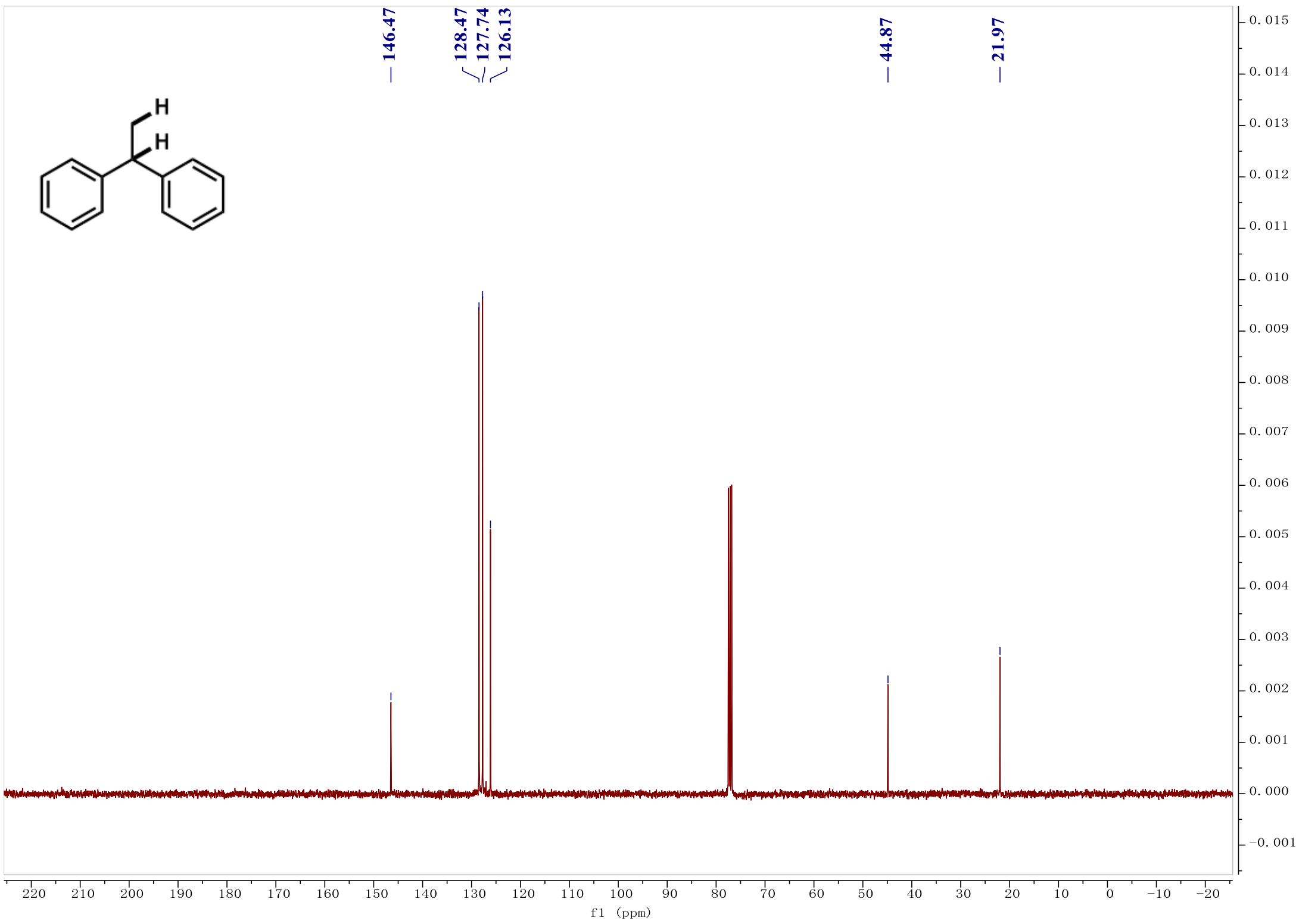

$^{13}$C NMR (101 MHz, $CDCl_3$) Spectrum of Compound **3e**

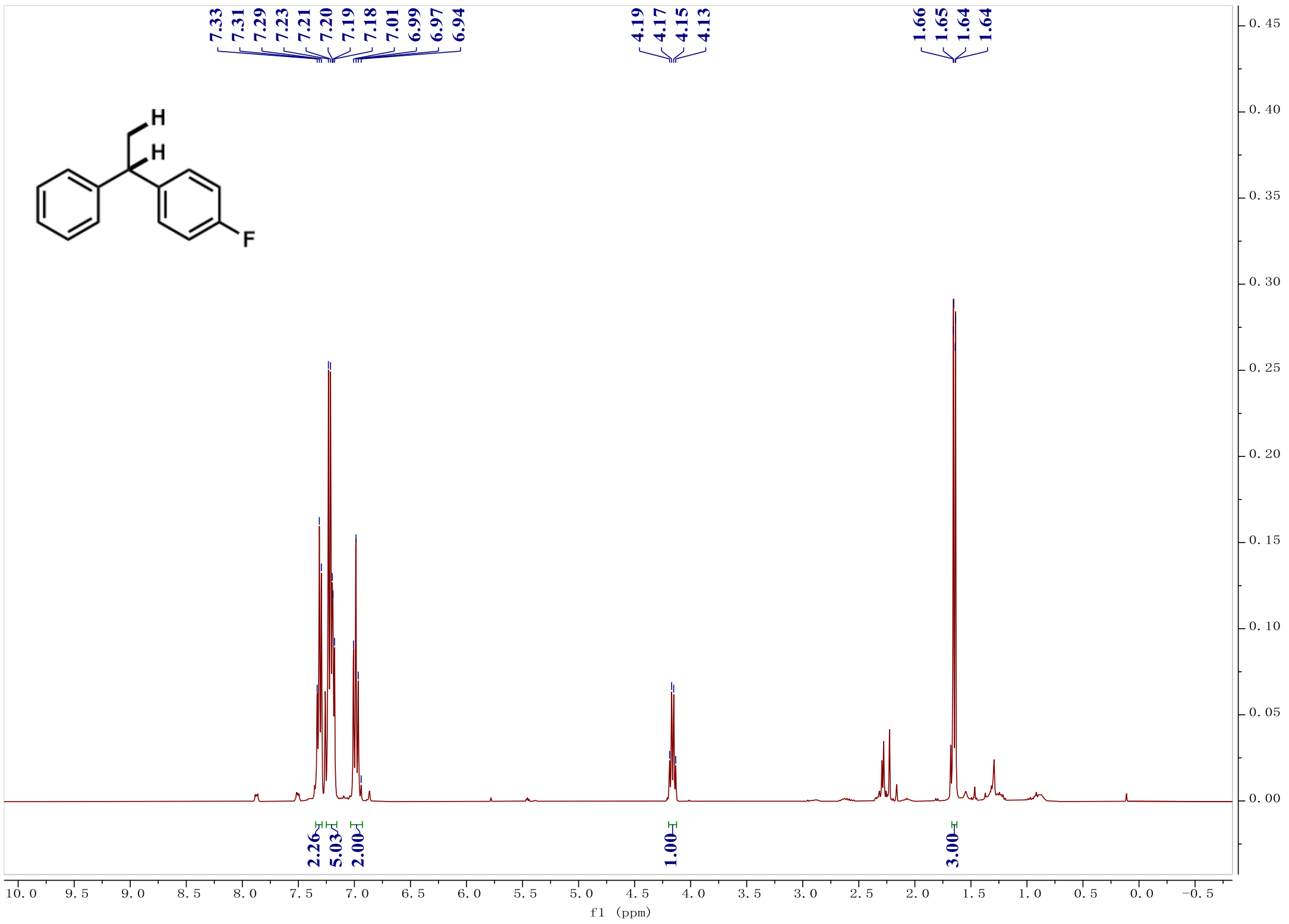


$^{1}$**H NMR** (400 MHz, CDCl$_3$) Spectrum of Compound **3f**

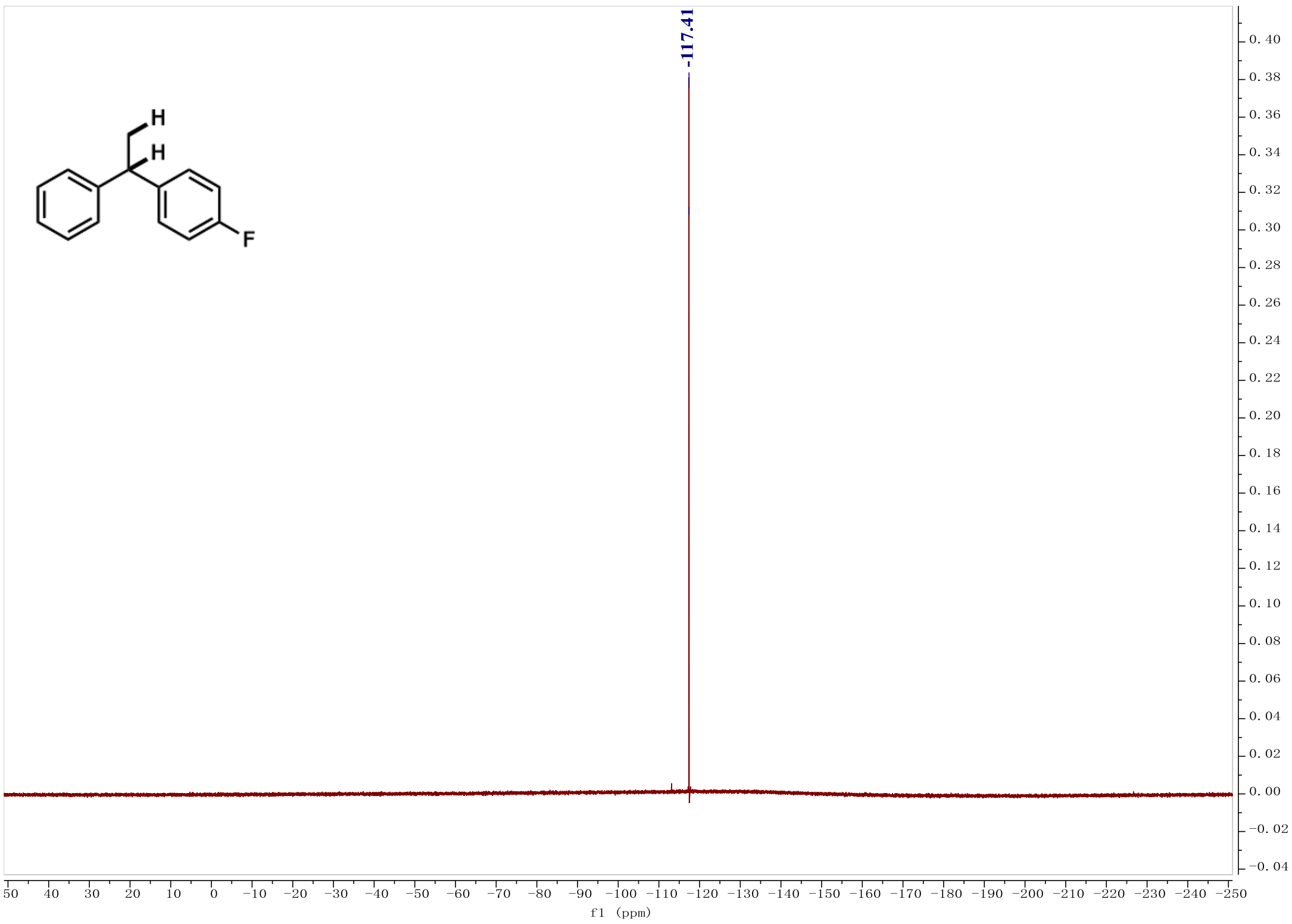


$^{19}$**F NMR** (376 MHz, CDCl$_3$) Spectrum of Compound **3f**

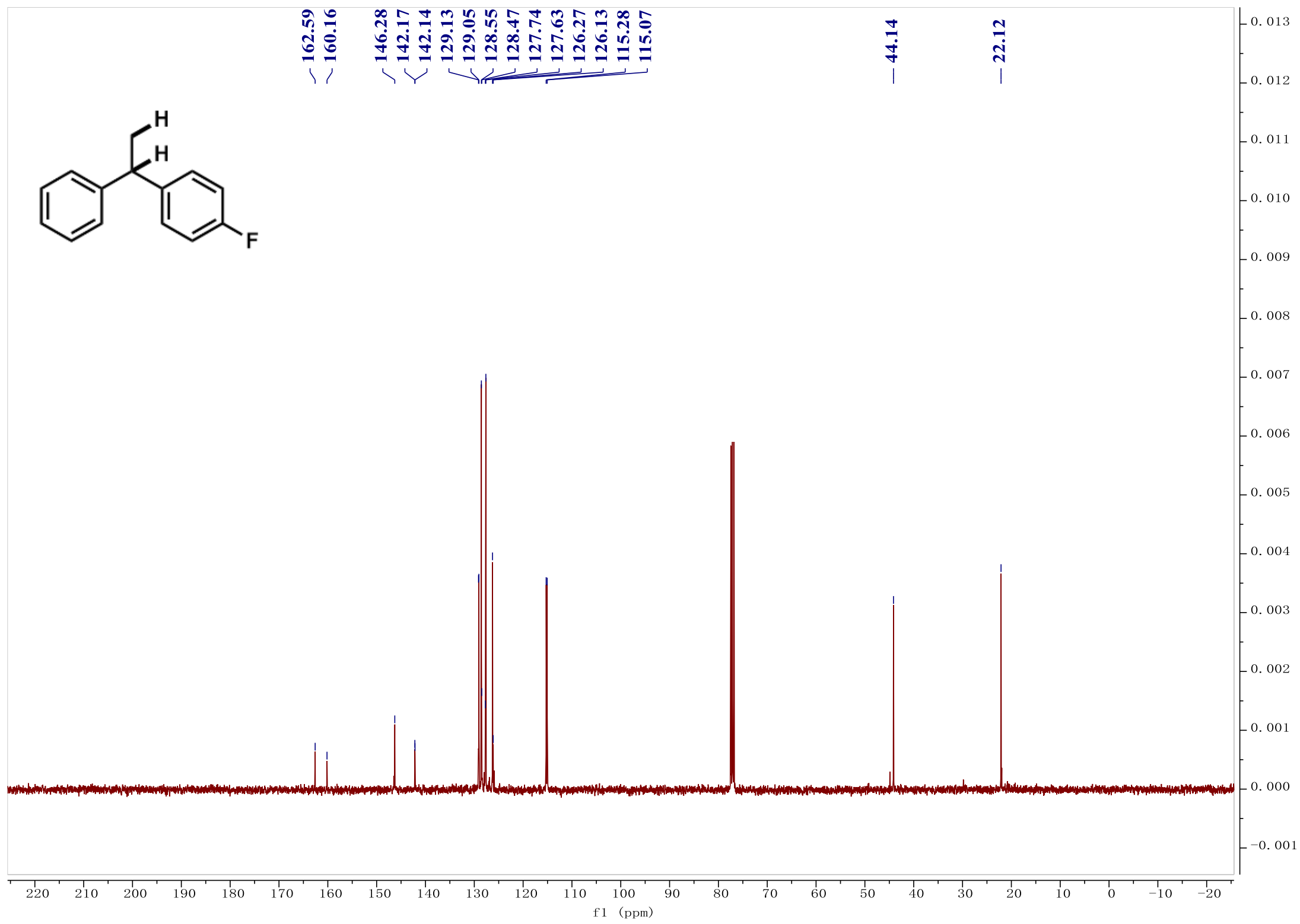

**$^{13}$C NMR** (101 MHz, $CDCl_3$) Spectrum of Compound **3f**

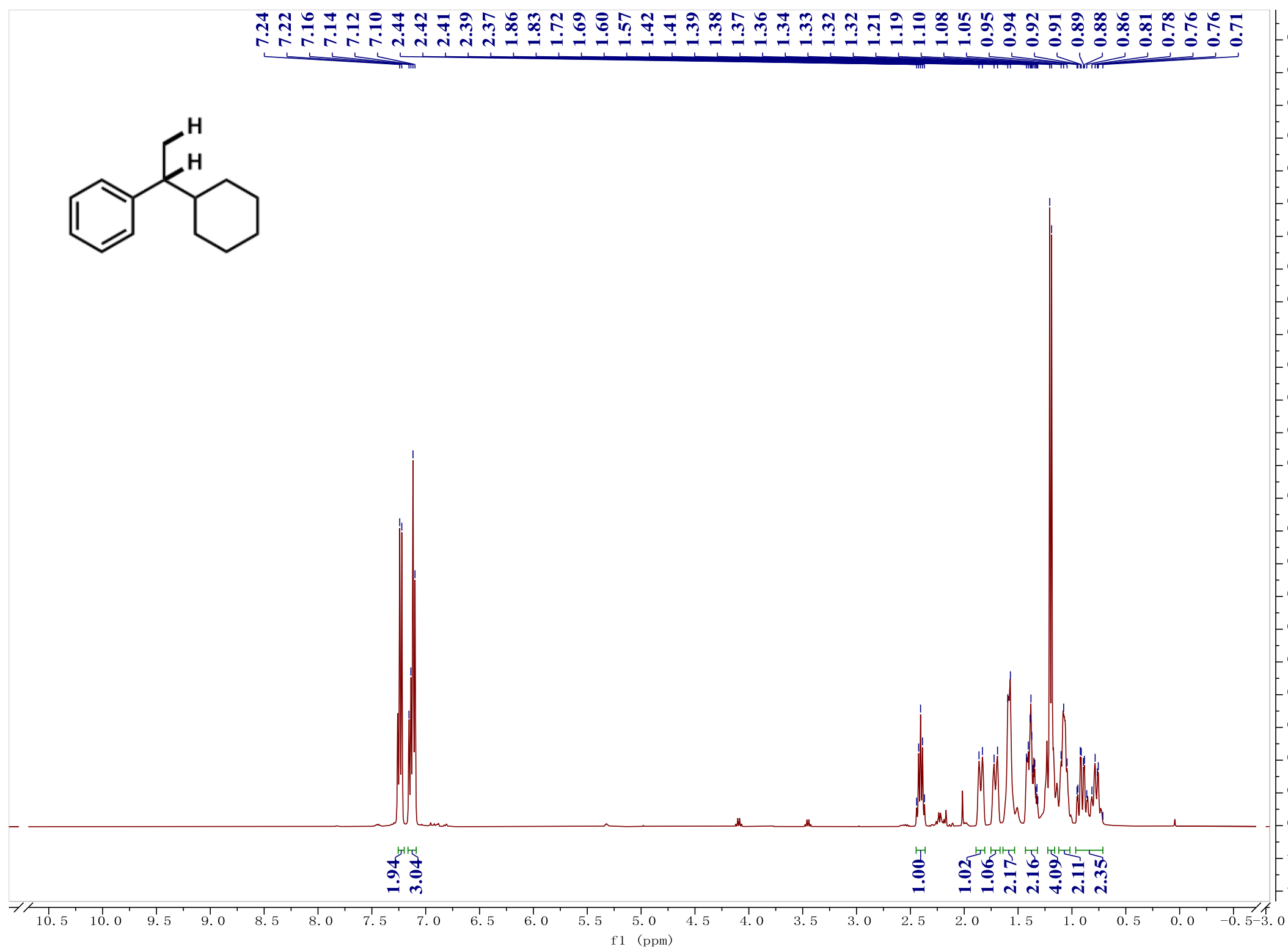

**$^{1}$H NMR** (400 MHz, $CDCl_3$) Spectrum of Compound **3g**

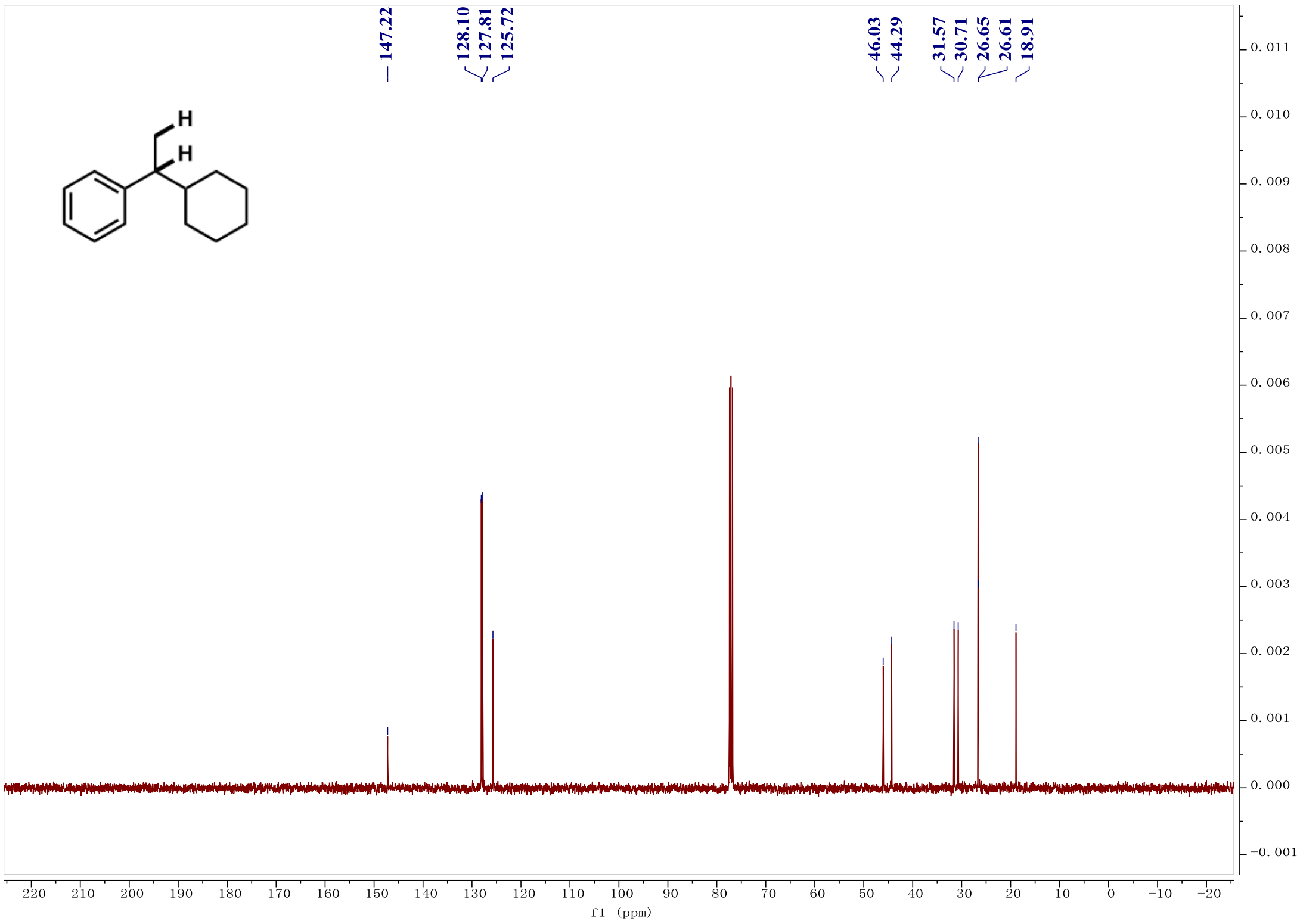

$^{13}$**C NMR** (101 MHz, $CDCl_3$) Spectrum of Compound **3g**

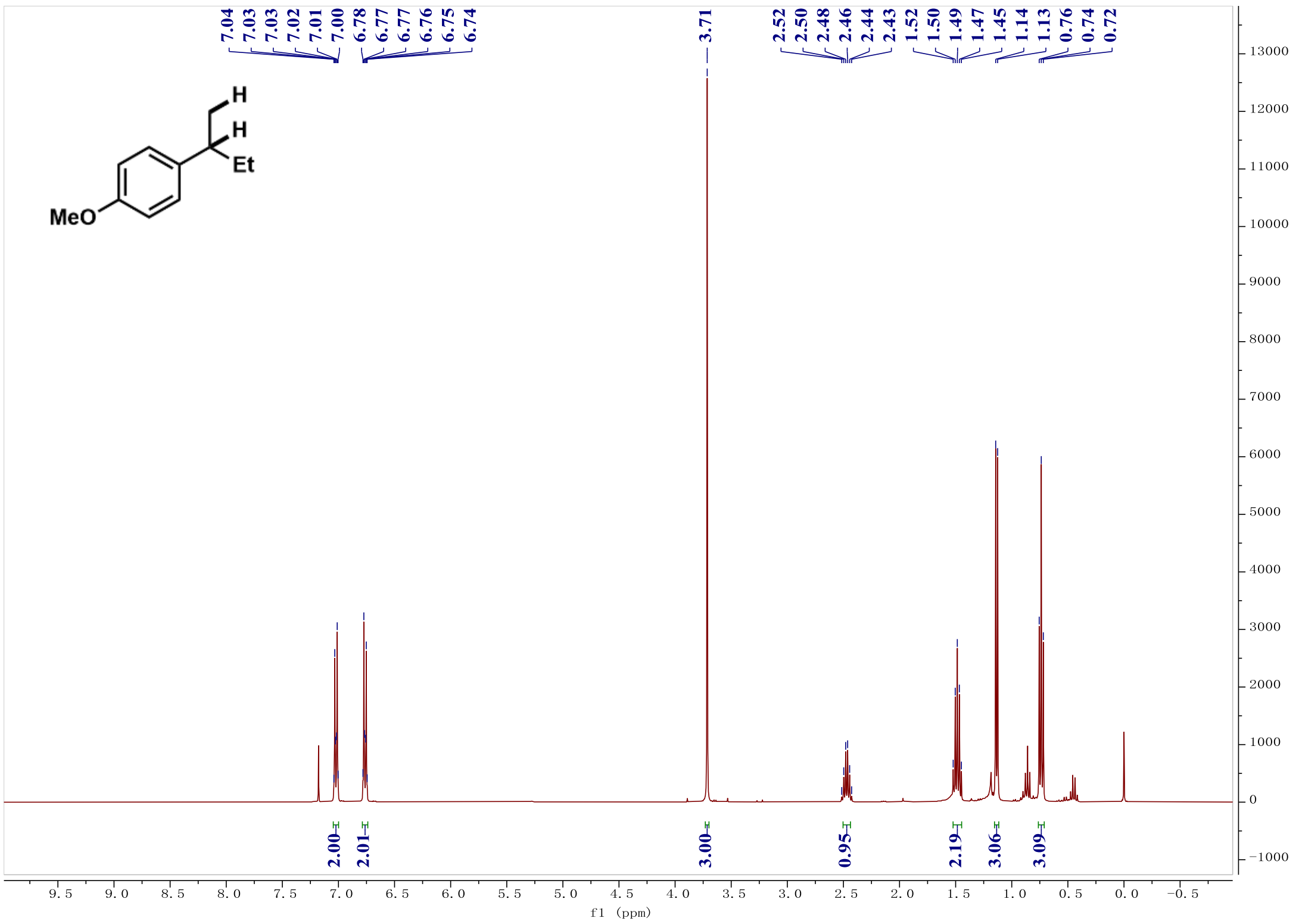

$^{1}$**H NMR** (400 MHz, $CDCl_3$) Spectrum of Compound **3h**

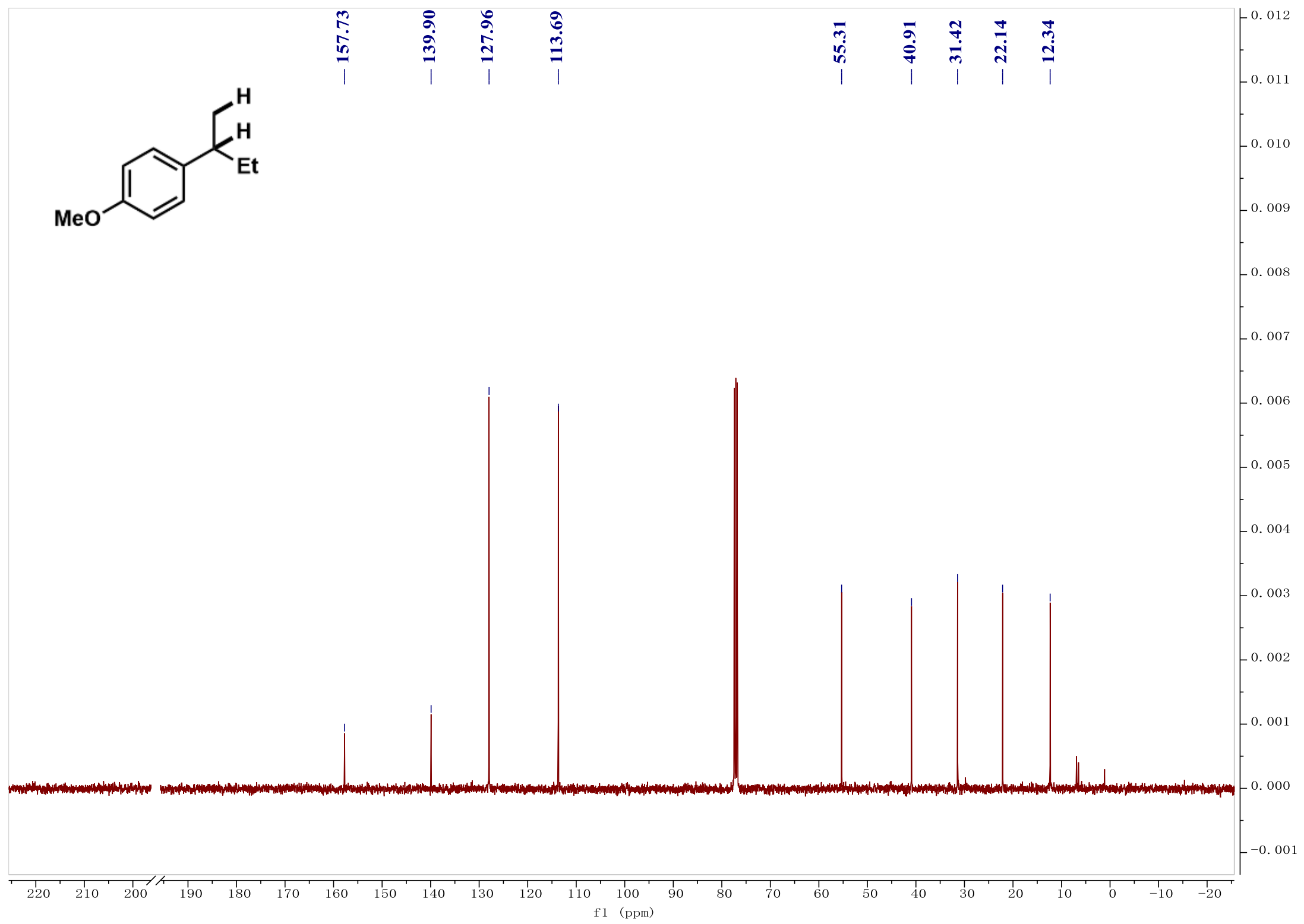


**$^{13}$C NMR** (101 MHz, $CDCl_3$) Spectrum of Compound **3h**

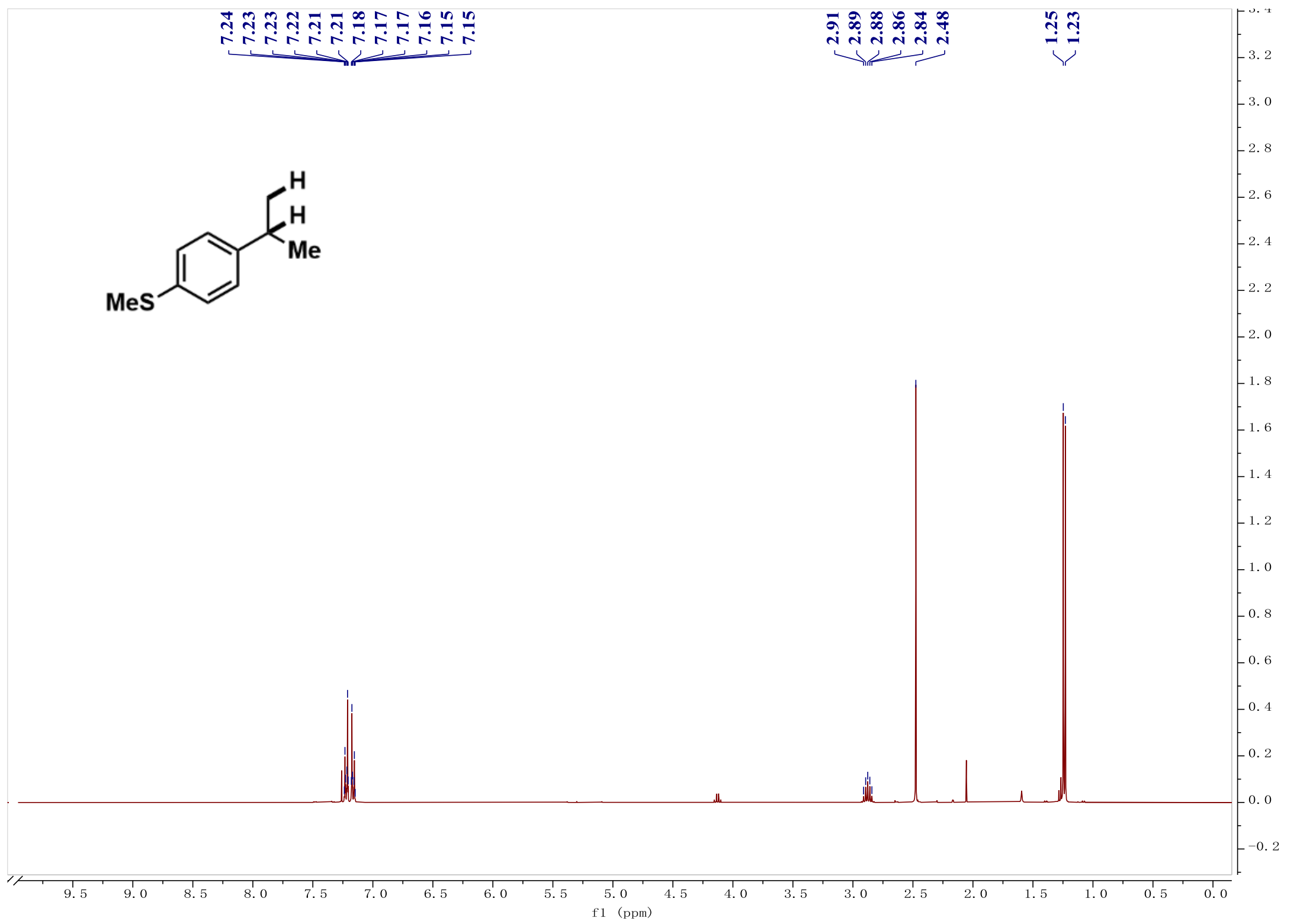


**$^{1}$H NMR** (400 MHz, $CDCl_3$) Spectrum of Compound **3i**

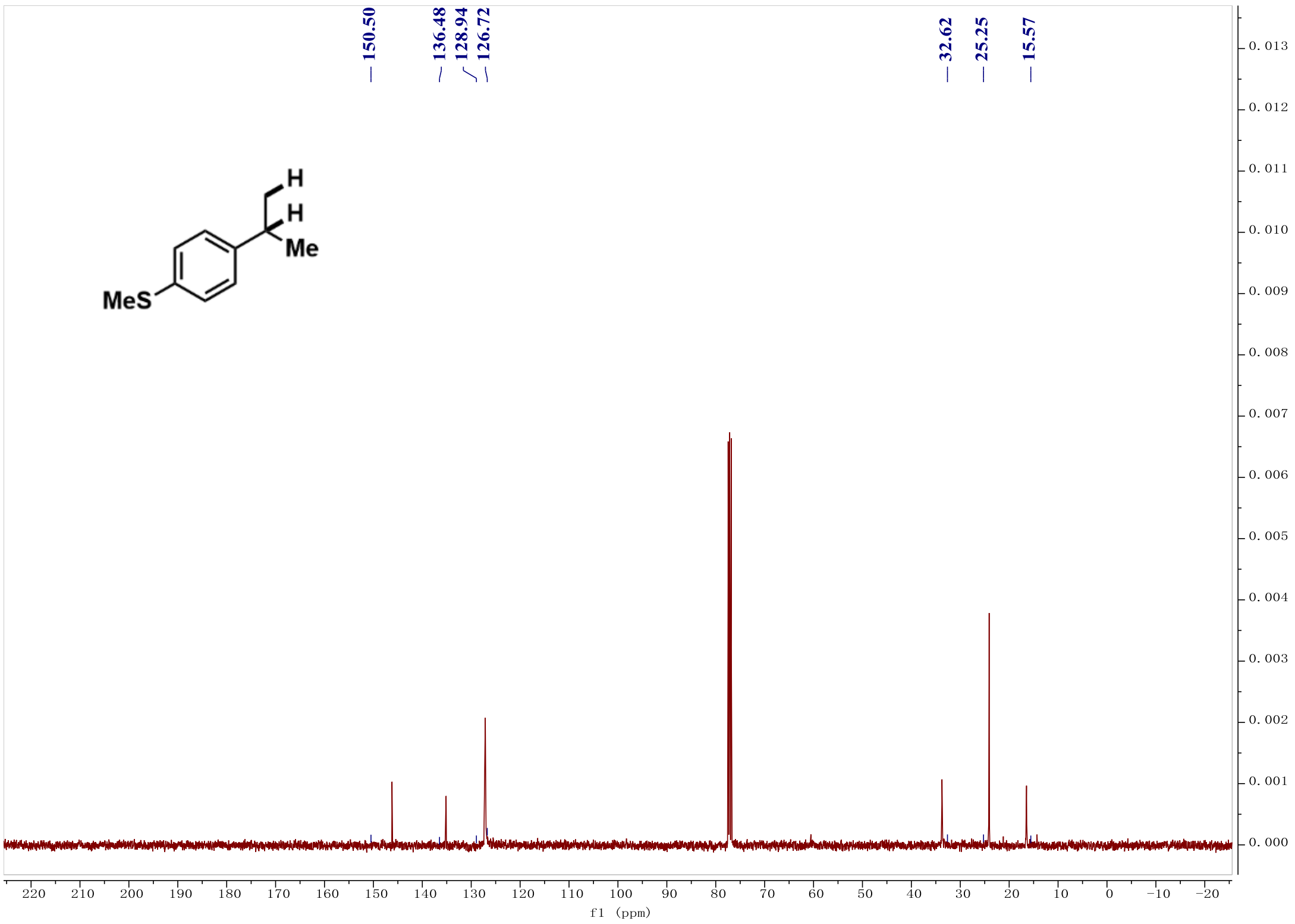

**$^{13}$C NMR** (101 MHz, $CDCl_3$) Spectrum of Compound **3i**

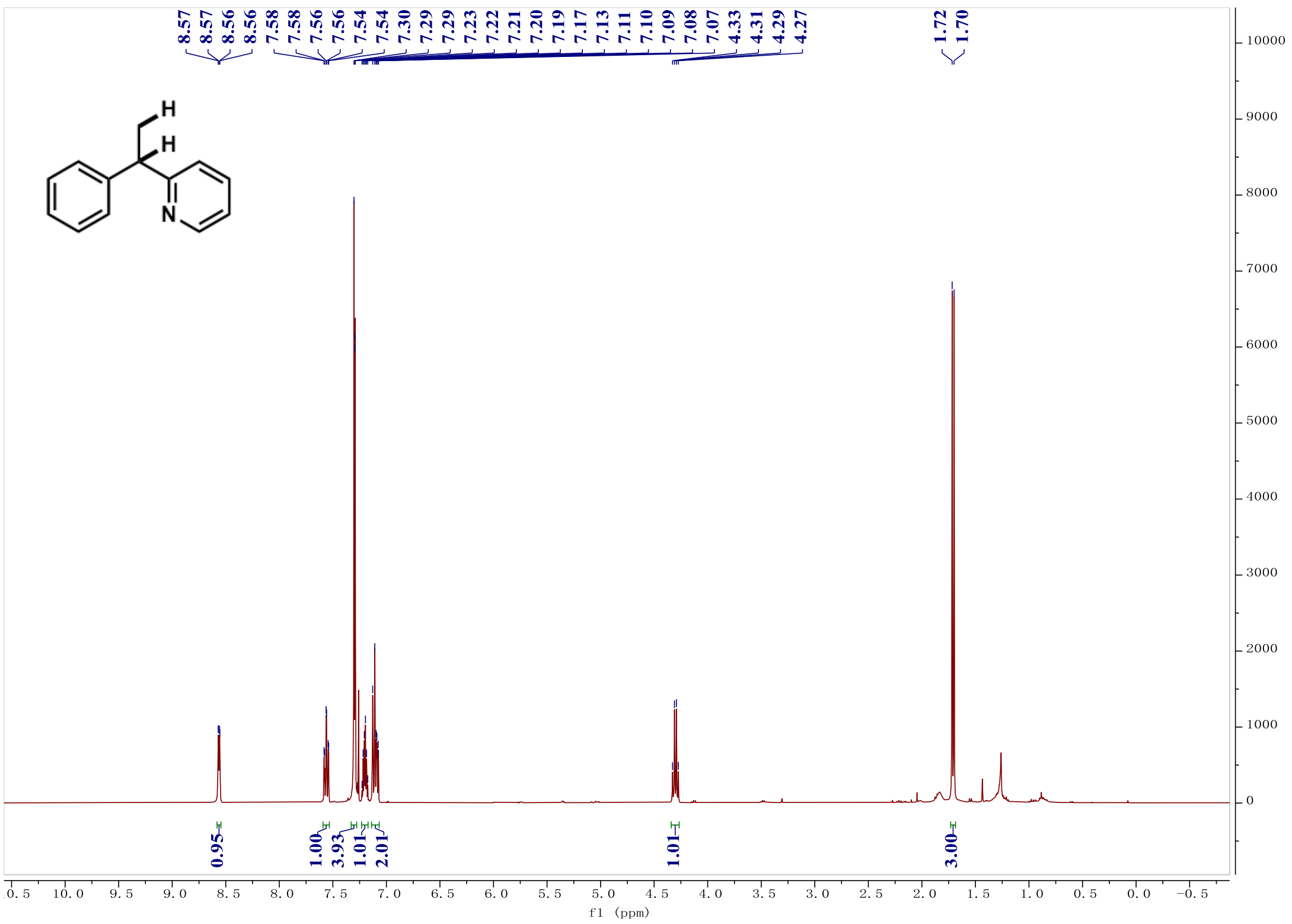

**$^{1}$H NMR** (400 MHz, $CDCl_3$) Spectrum of Compound **3j**

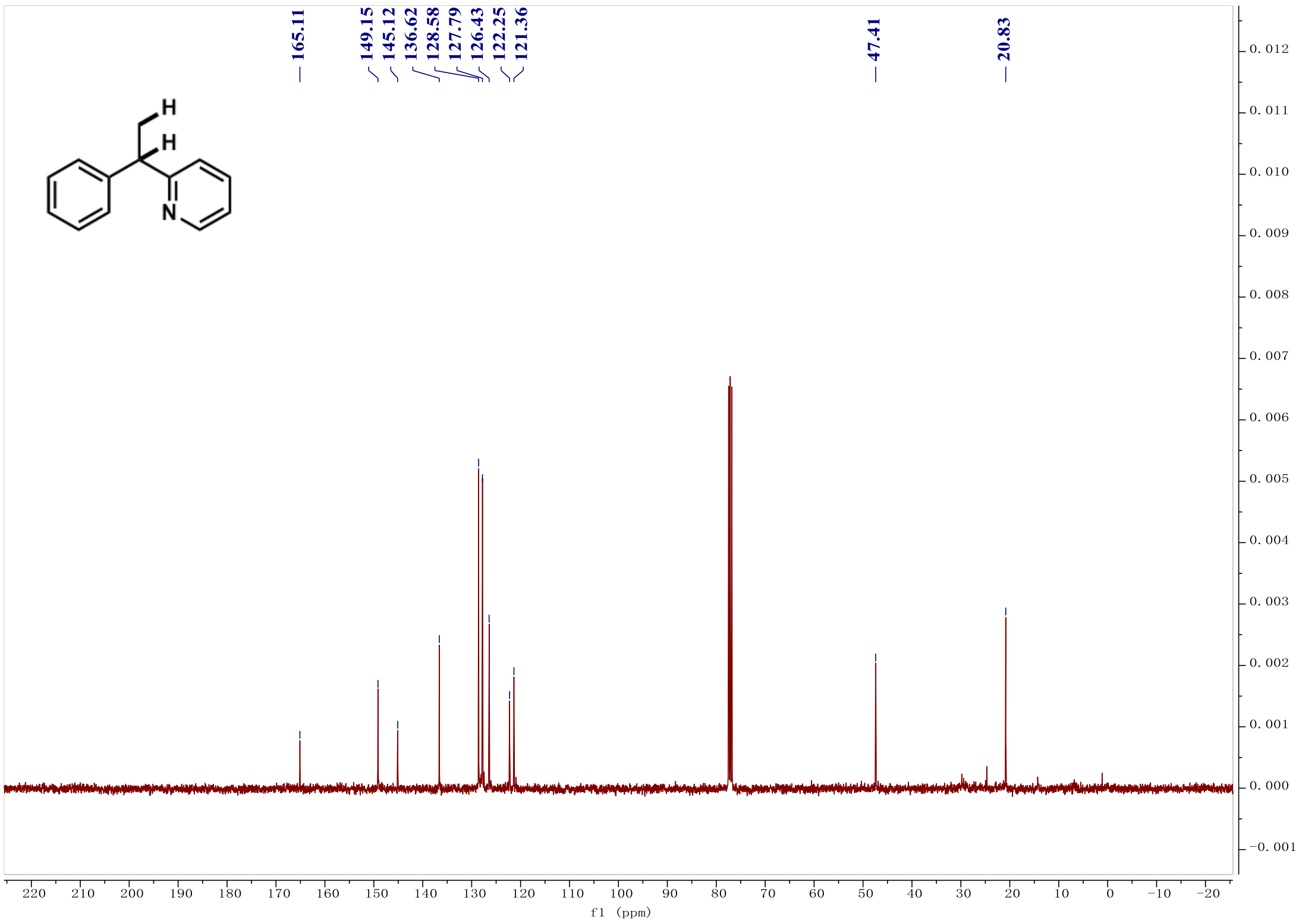


$^{13}$C **NMR** (101 MHz, $CDCl_3$) Spectrum of Compound **3j**

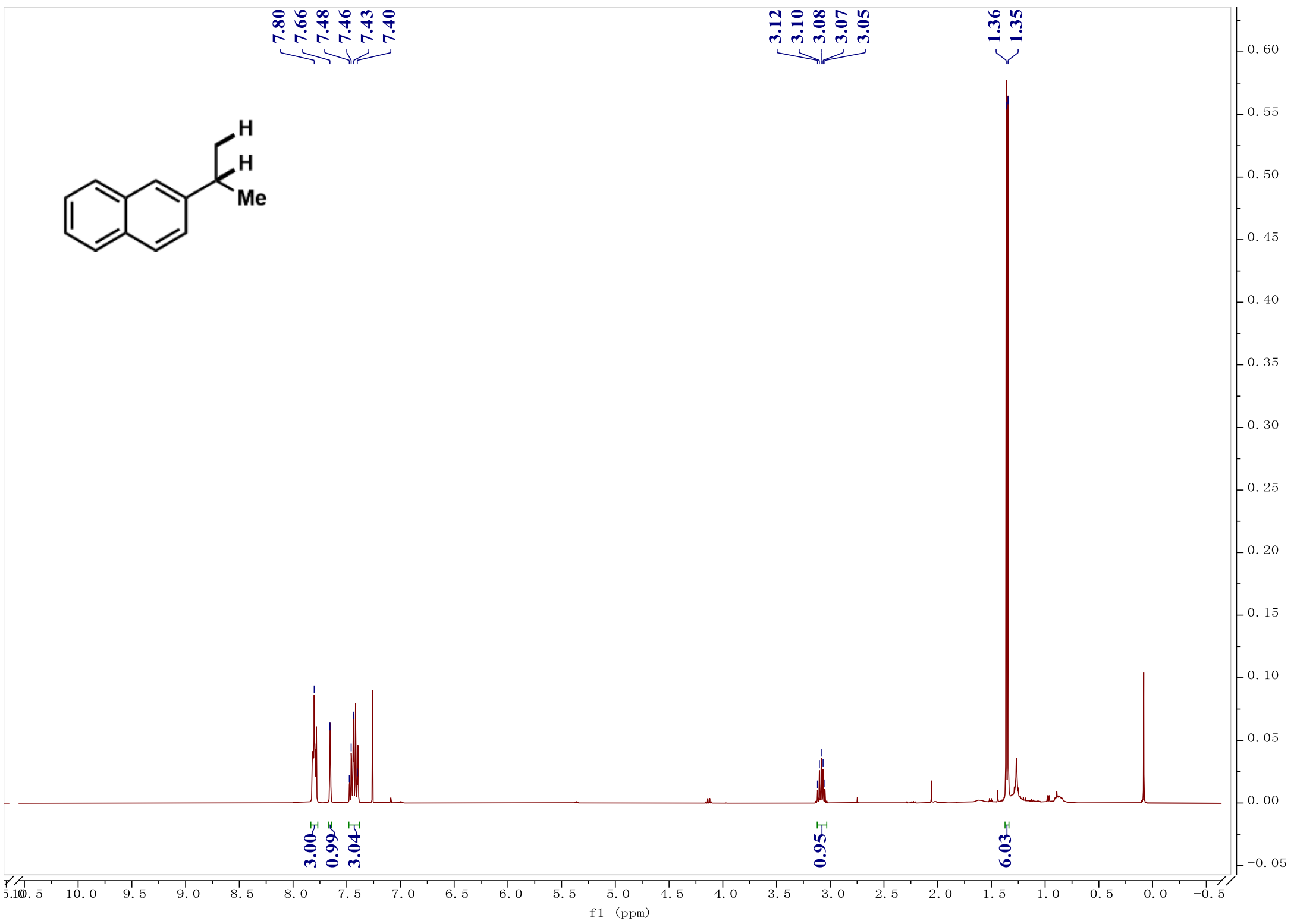


$^{1}$H **NMR** (400 MHz, $CDCl_3$) Spectrum of Compound **3k**

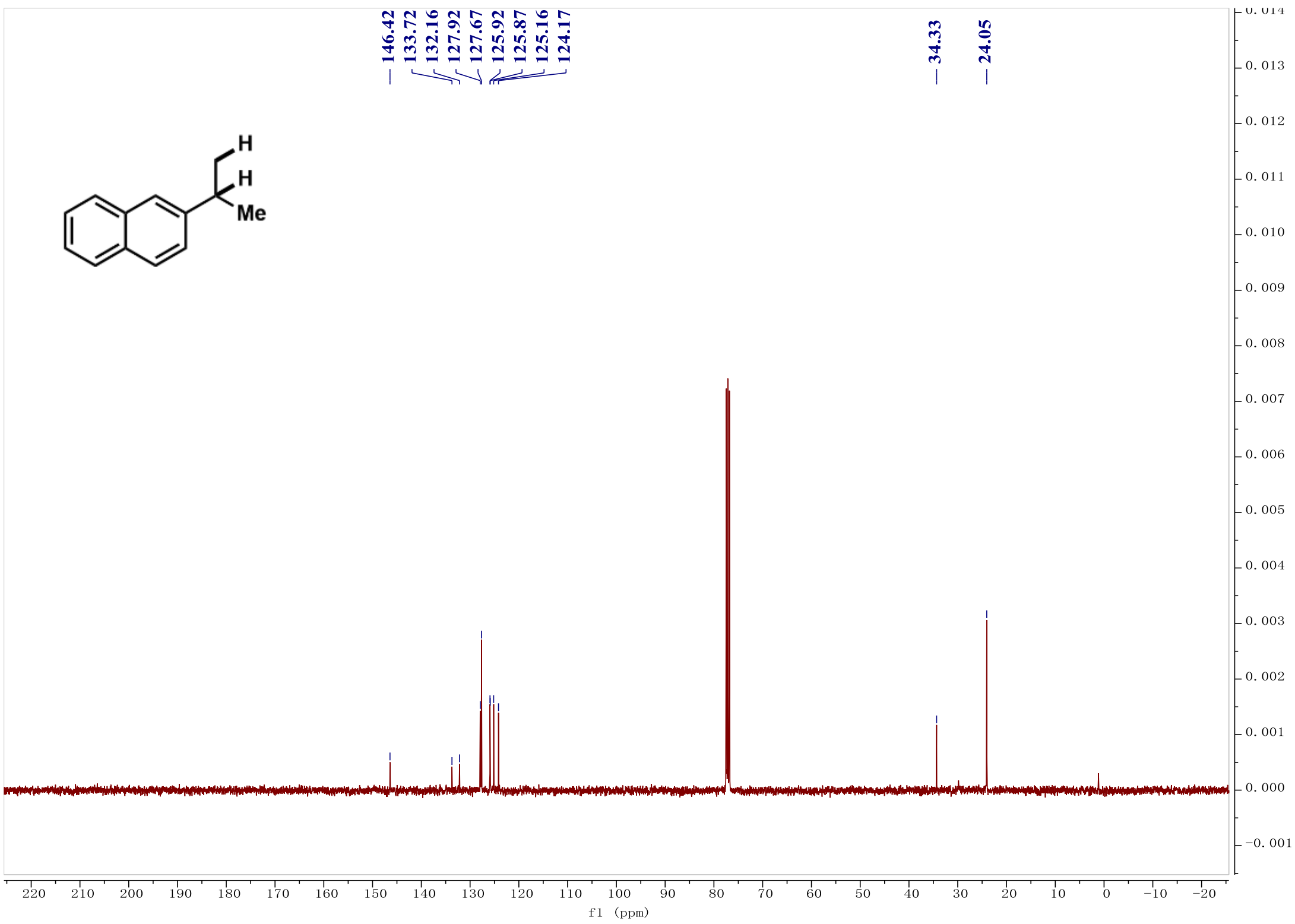

**$^{13}$C NMR** (101 MHz, $CDCl_3$) Spectrum of Compound **3k**

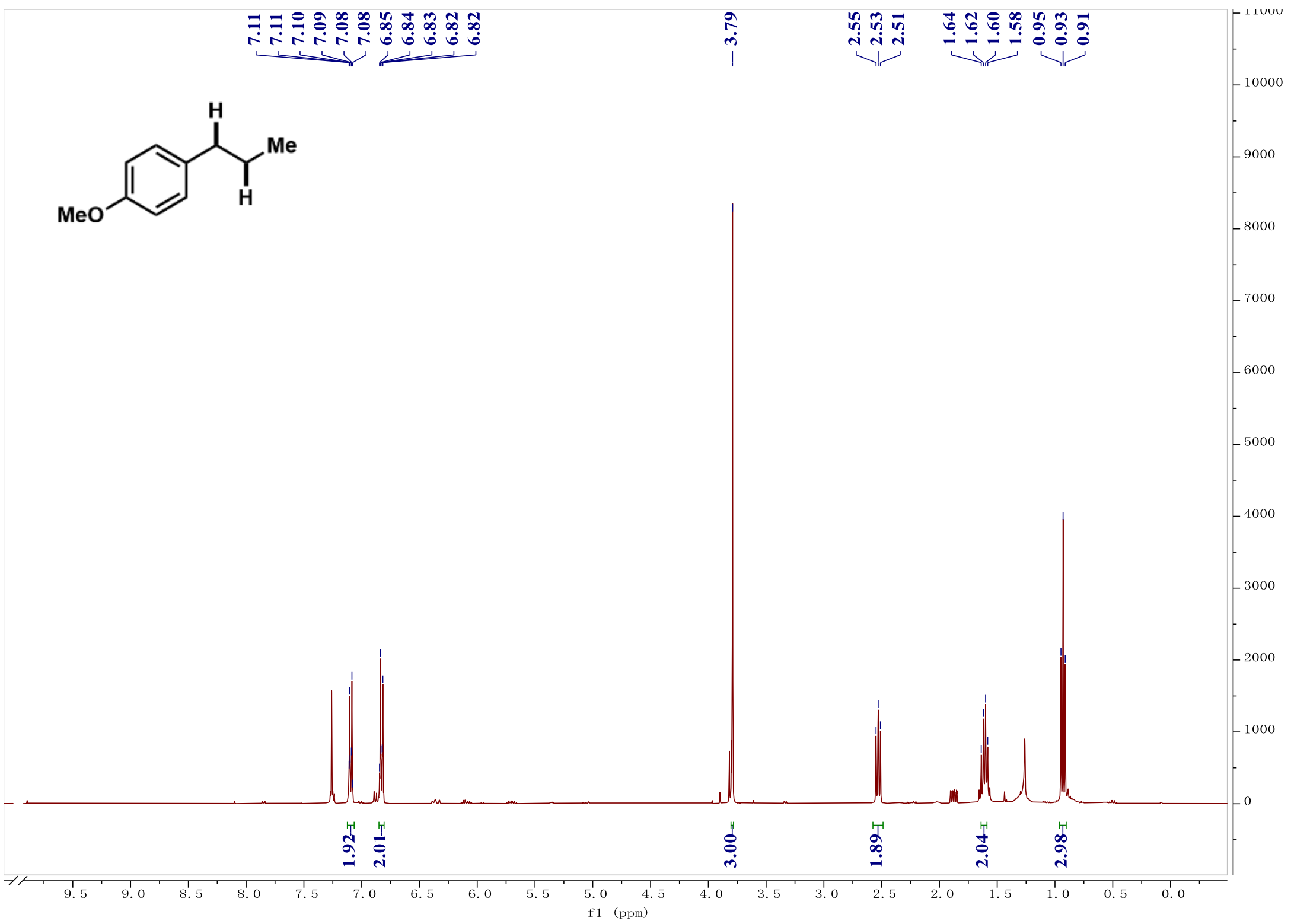

**$^{1}$H NMR** (400 MHz, $CDCl_3$) Spectrum of Compound **3l**

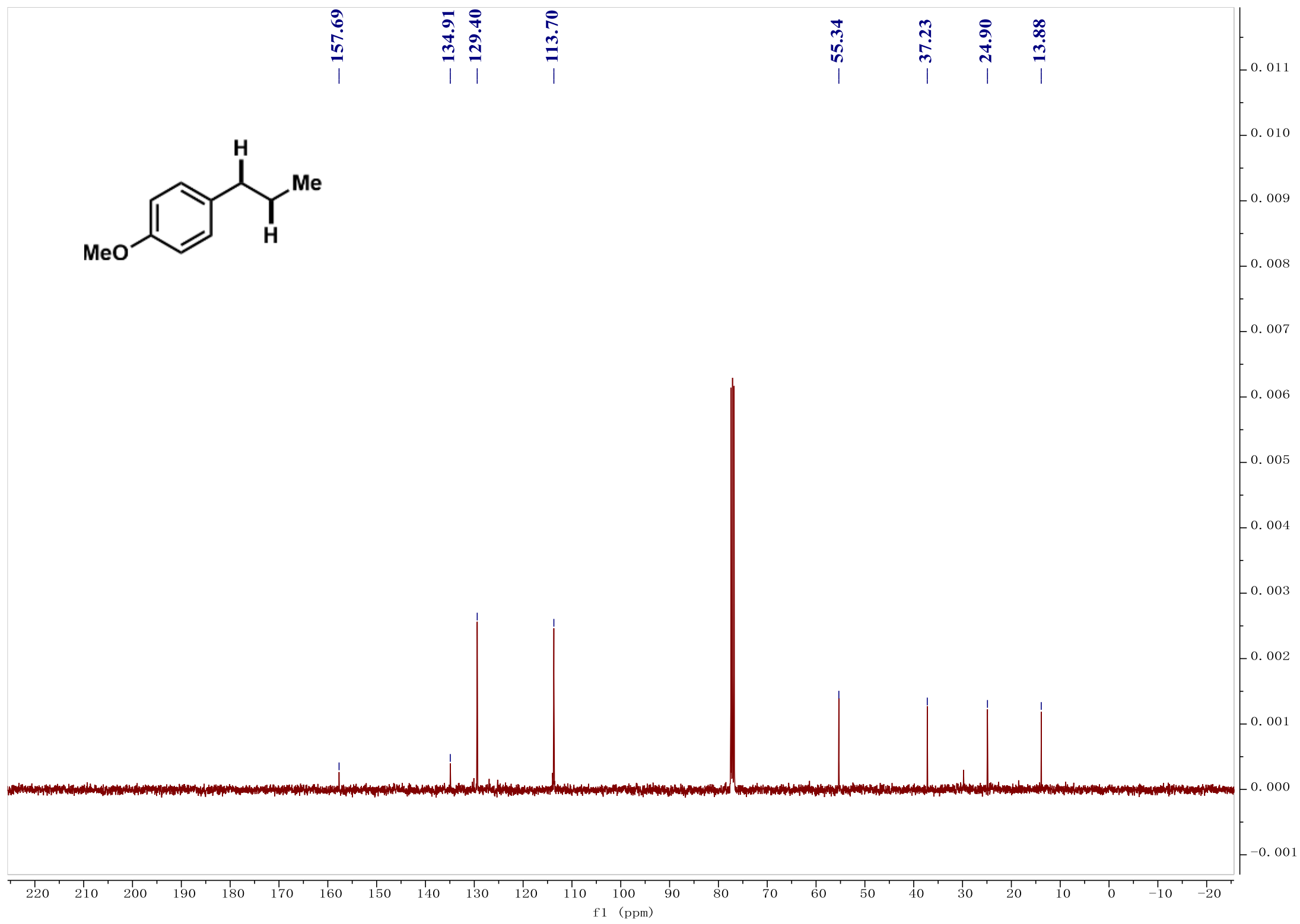


$^{13}$**C NMR** (101 MHz, $CDCl_3$) Spectrum of Compound **3l**

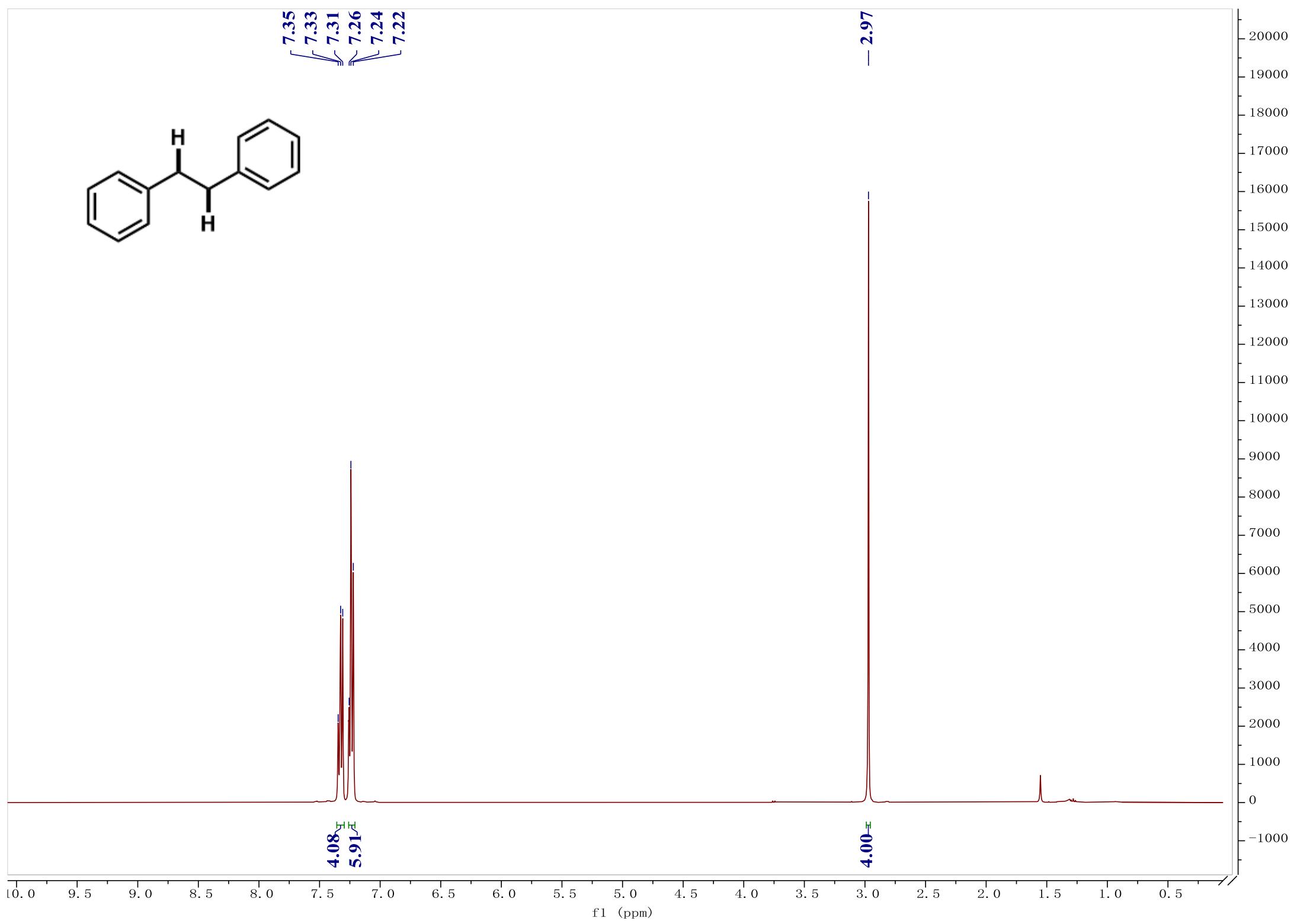


$^{1}$**H NMR** (400 MHz, $CDCl_3$) Spectrum of Compound **3m**

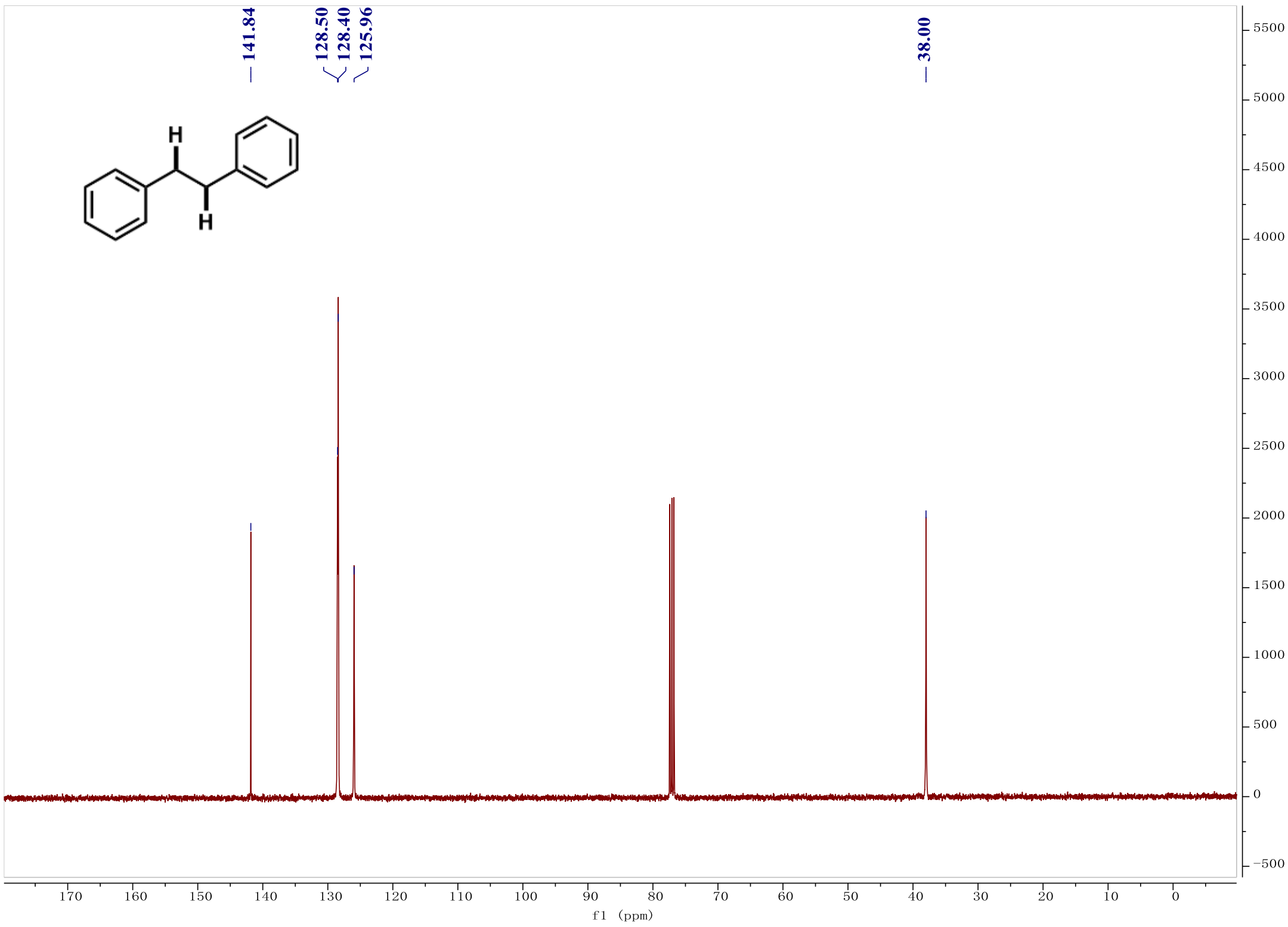


$^{13}$C NMR (101 MHz, $CDCl_3$) Spectrum of Compound **3m**

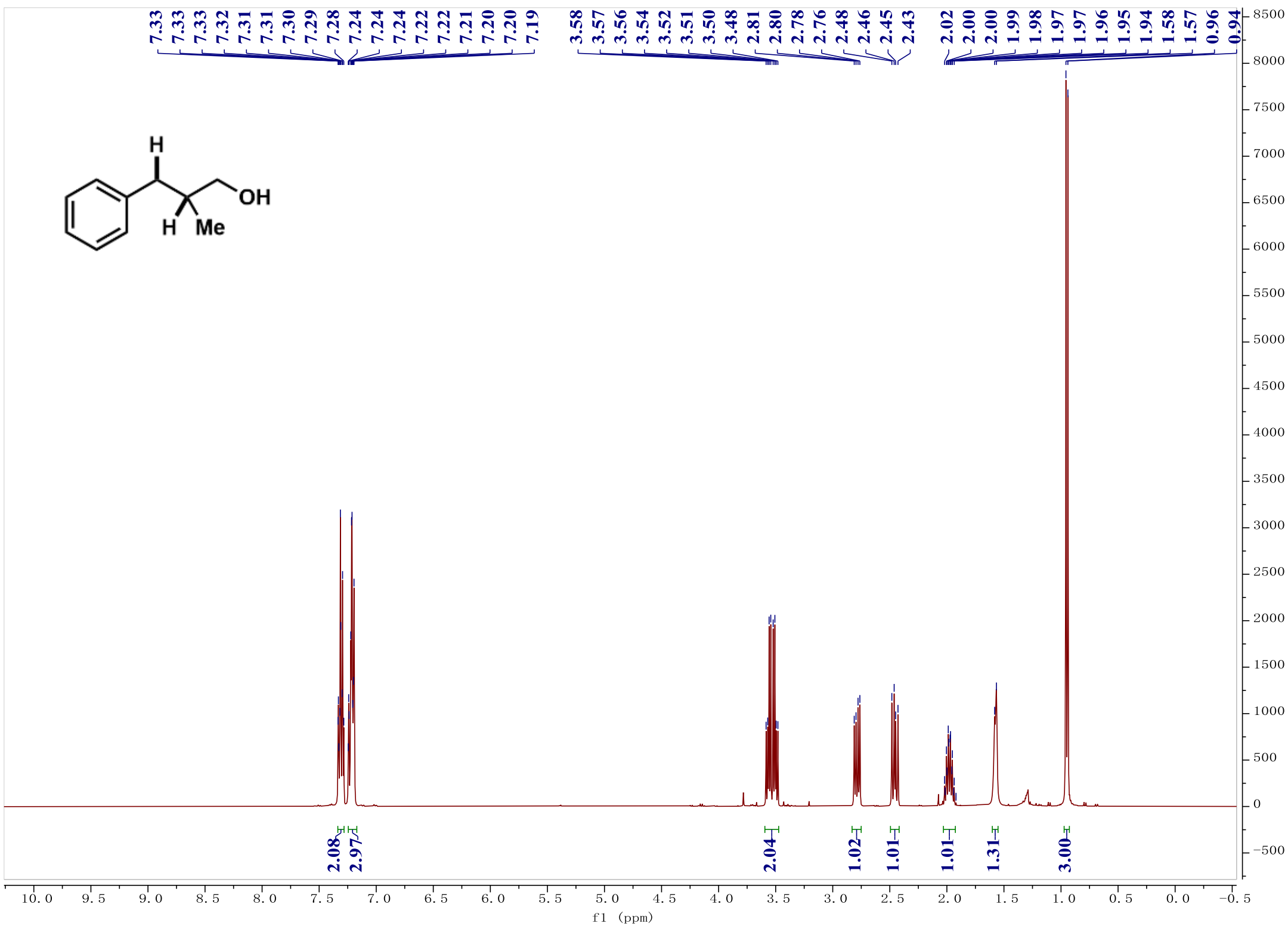


$^{1}$H NMR (400 MHz, $CDCl_3$) Spectrum of Compound **3n**

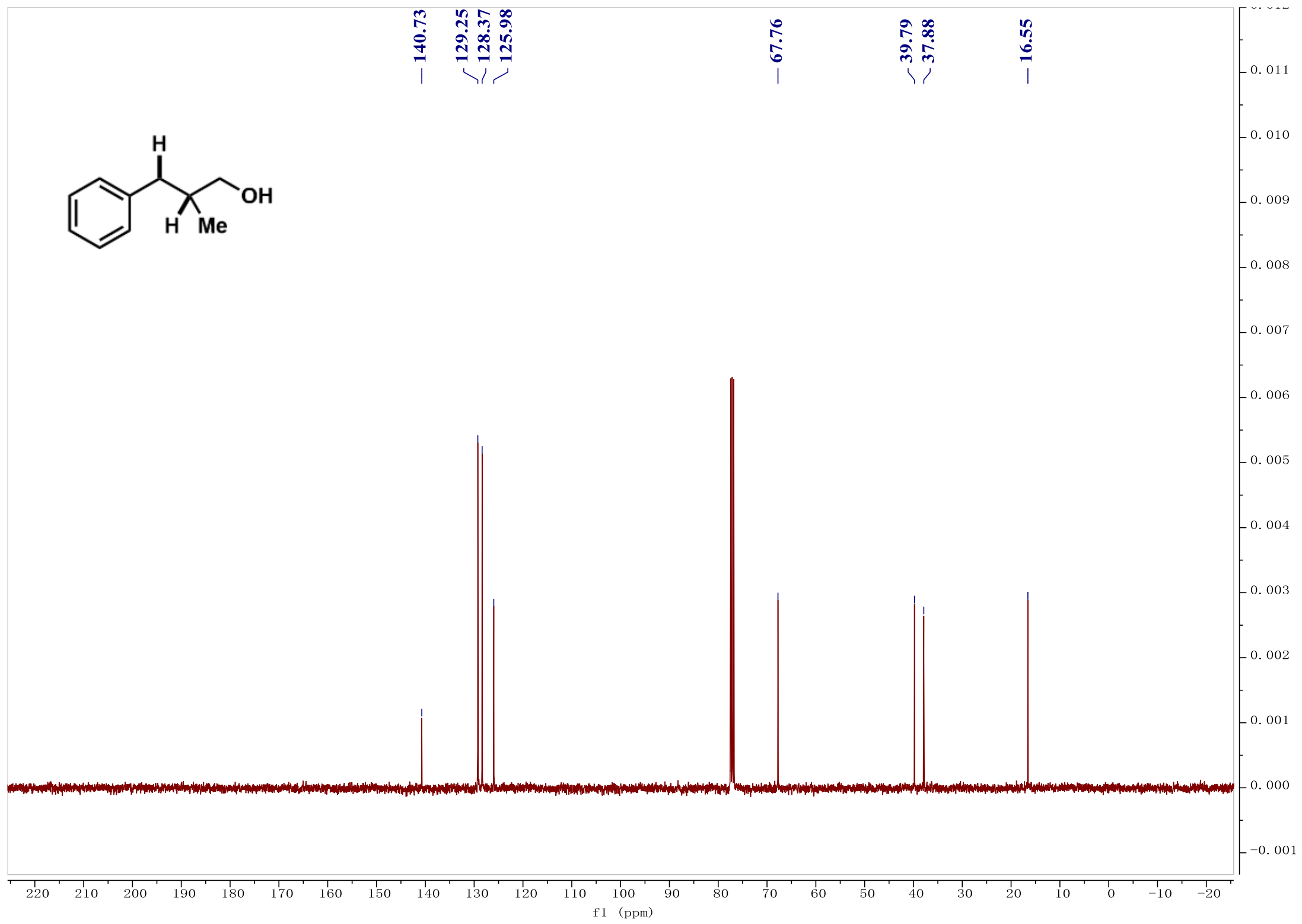

$^{13}$**C NMR** (101 MHz, $CDCl_3$) Spectrum of Compound **3n**

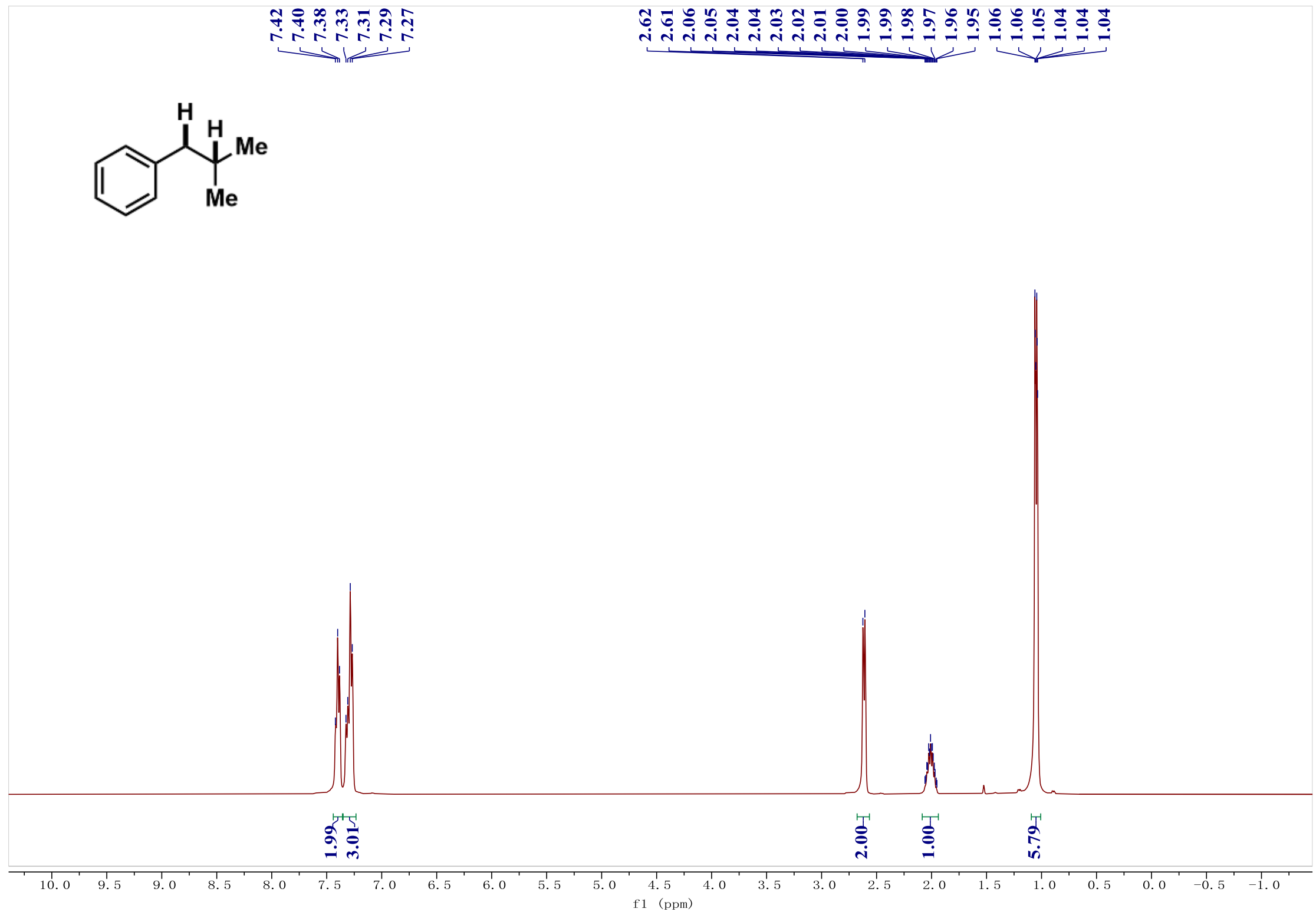

$^{1}$**H NMR** (400 MHz, $CDCl_3$) Spectrum of Compound **3o**

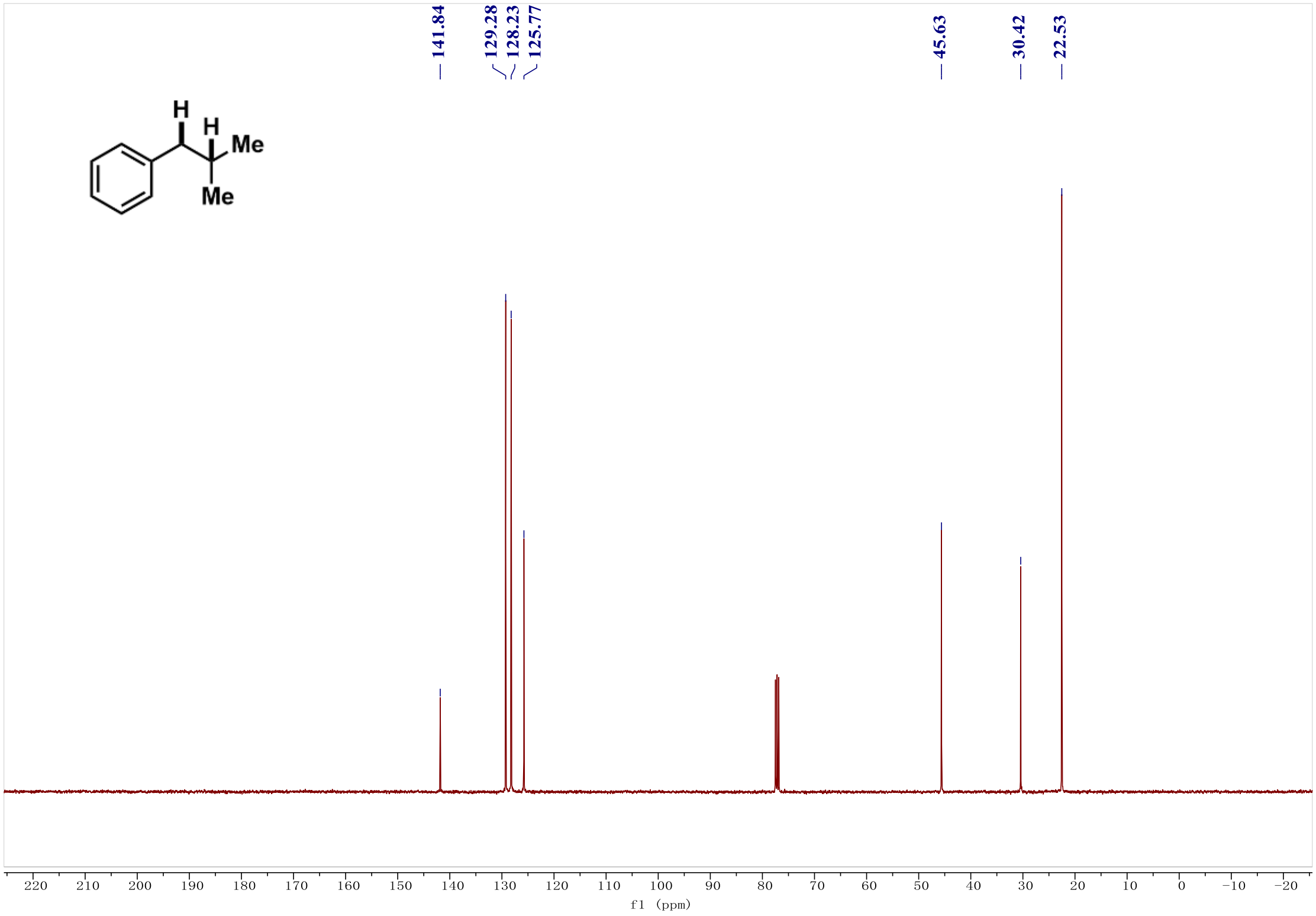


$^{13}$C NMR (101 MHz, CDCl$_3$) Spectrum of Compound **3o**

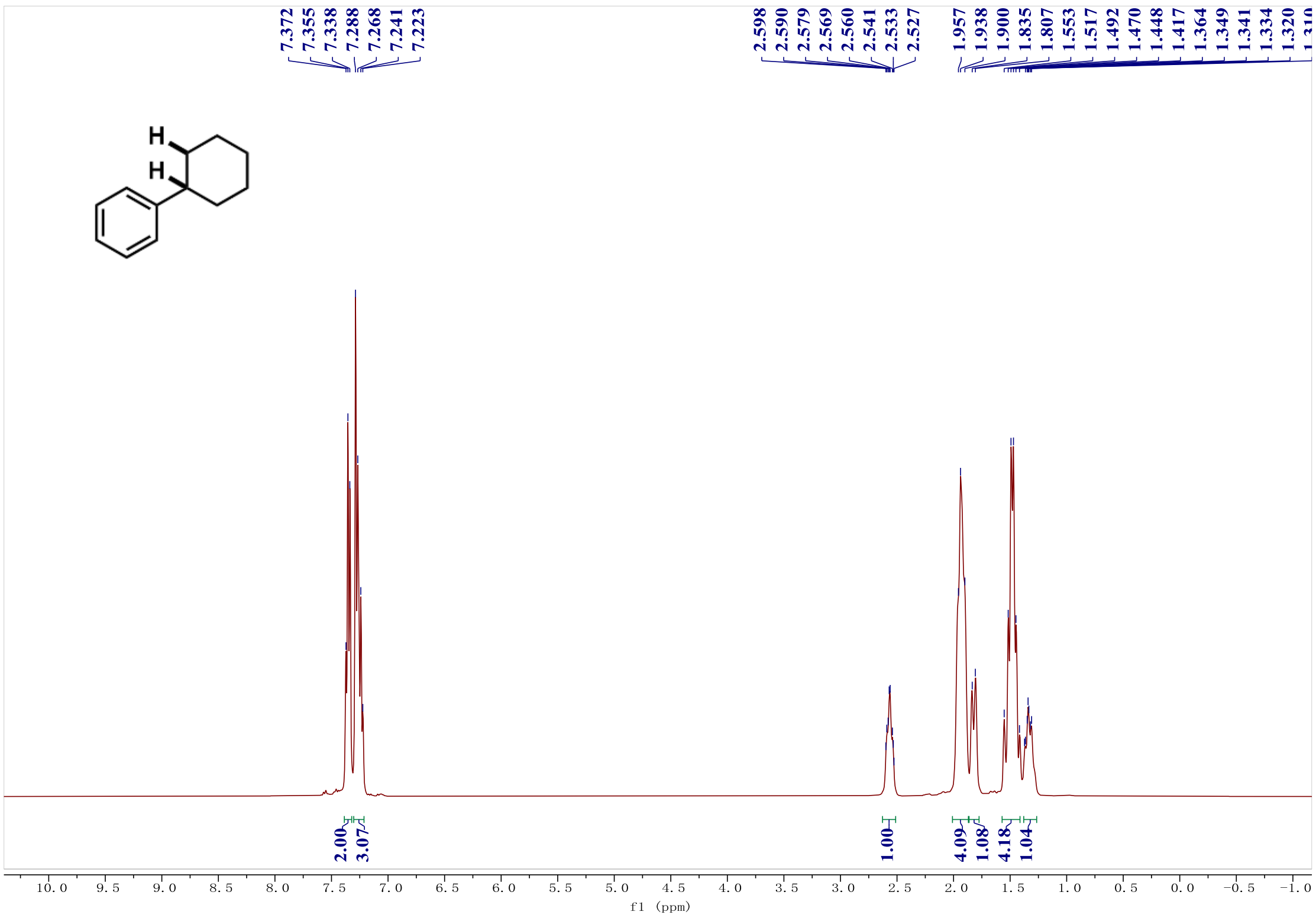


$^{1}$H NMR (400 MHz, CDCl$_3$) Spectrum of Compound **3p**

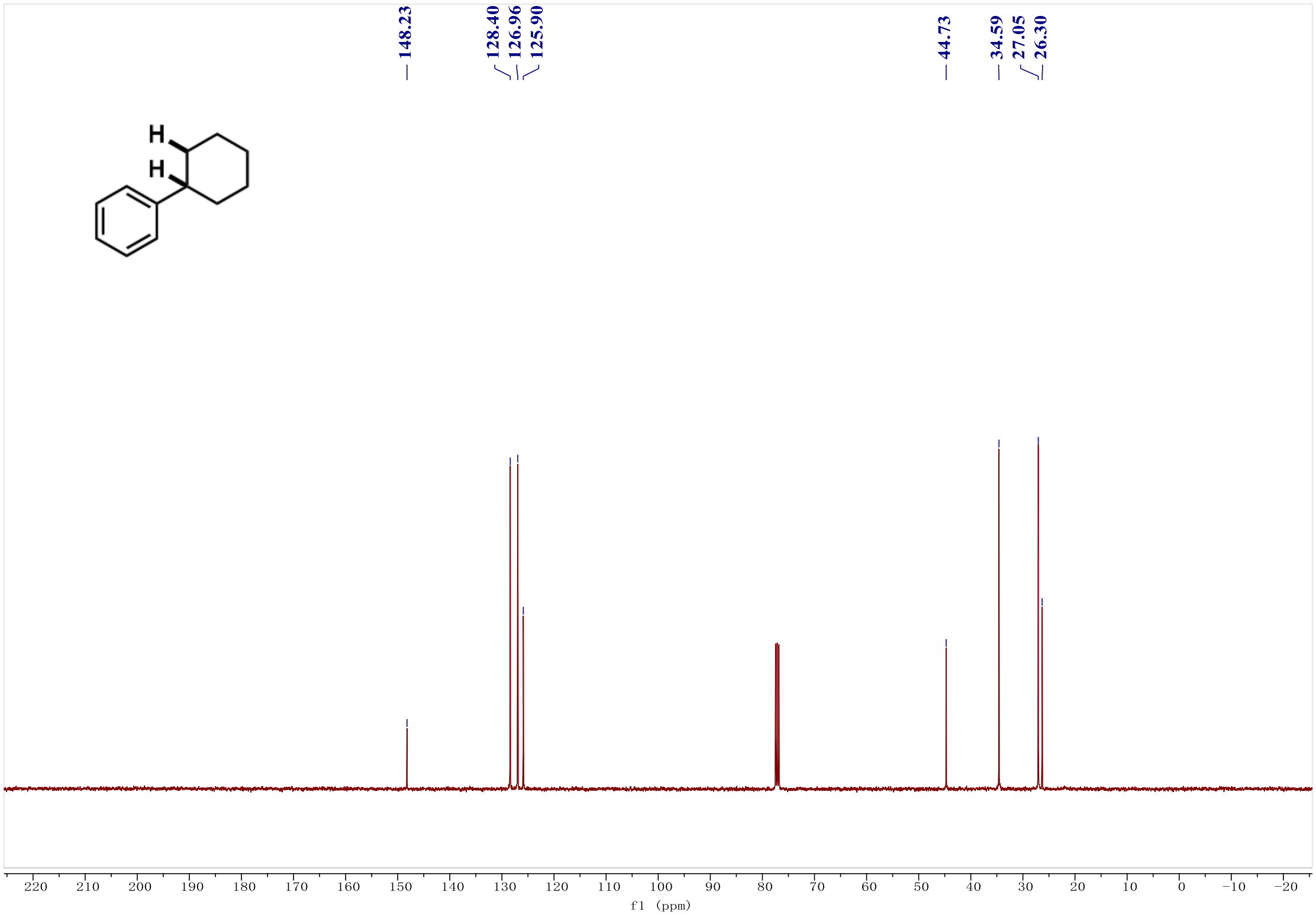

**$^{13}$C NMR** (101 MHz, $CDCl_3$) Spectrum of Compound **3p**

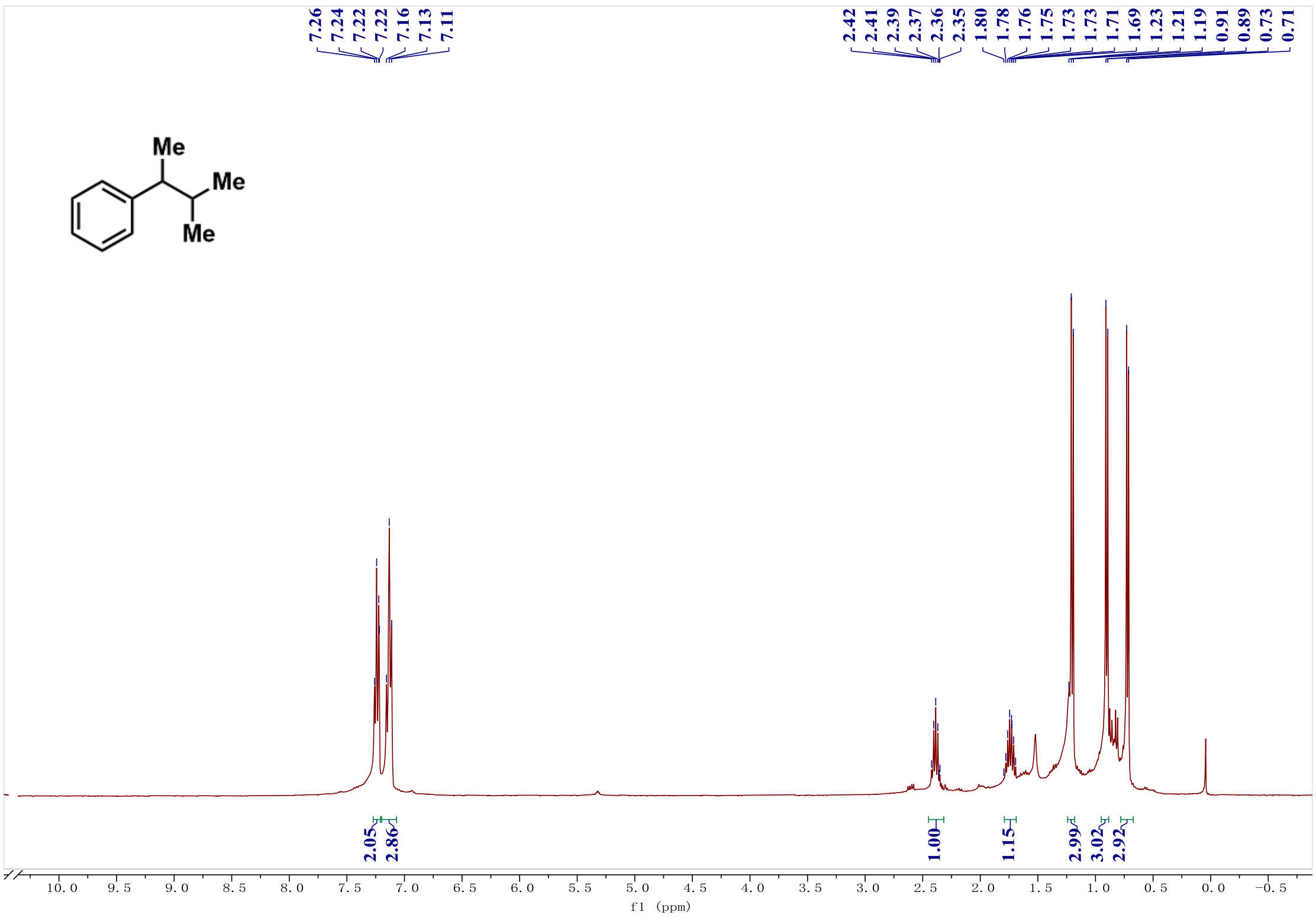

**$^{1}$H NMR** (400 MHz, $CDCl_3$) Spectrum of Compound **3q**

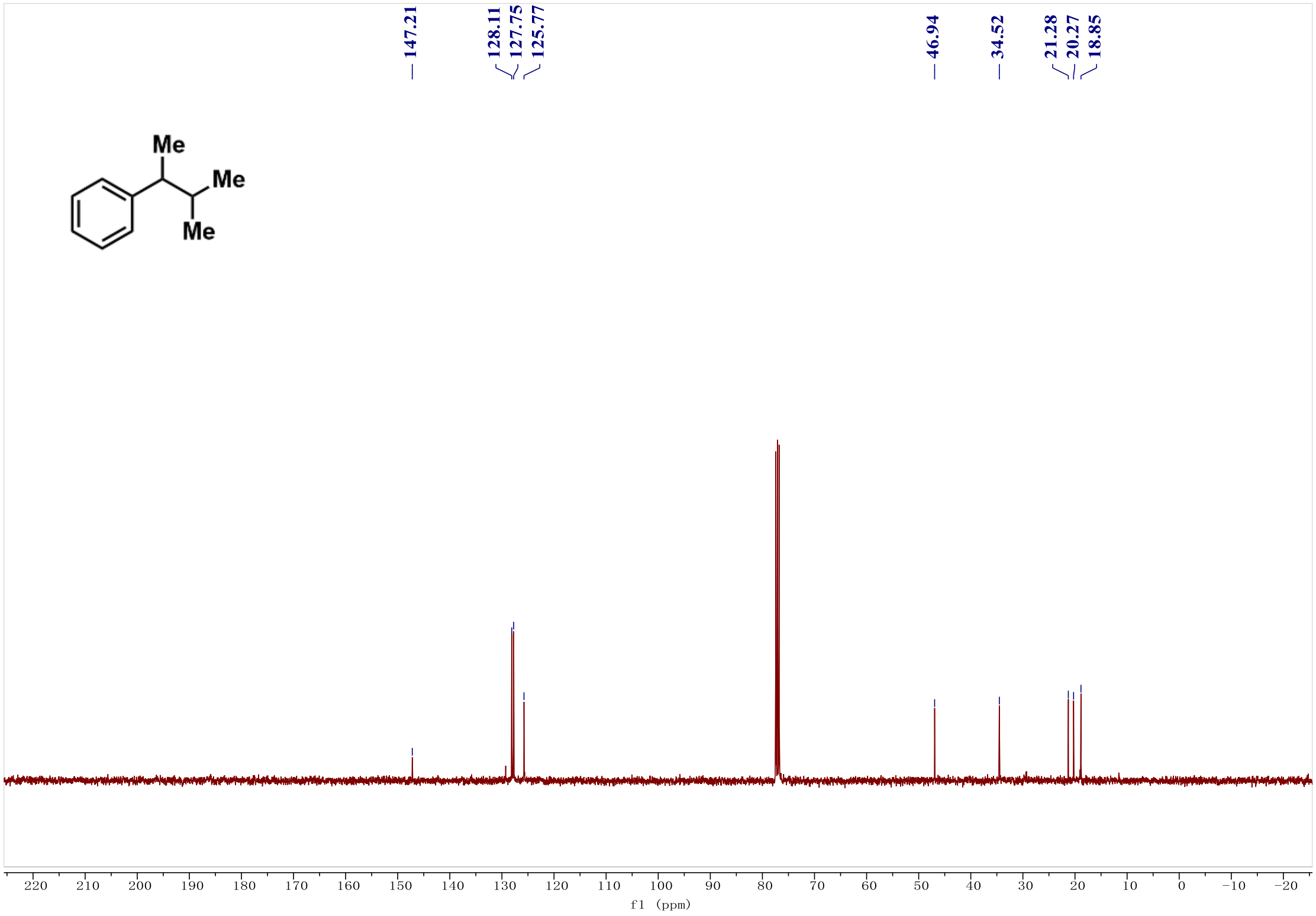

$^{13}$**C NMR** (101 MHz, $CDCl_3$) Spectrum of Compound **3q**

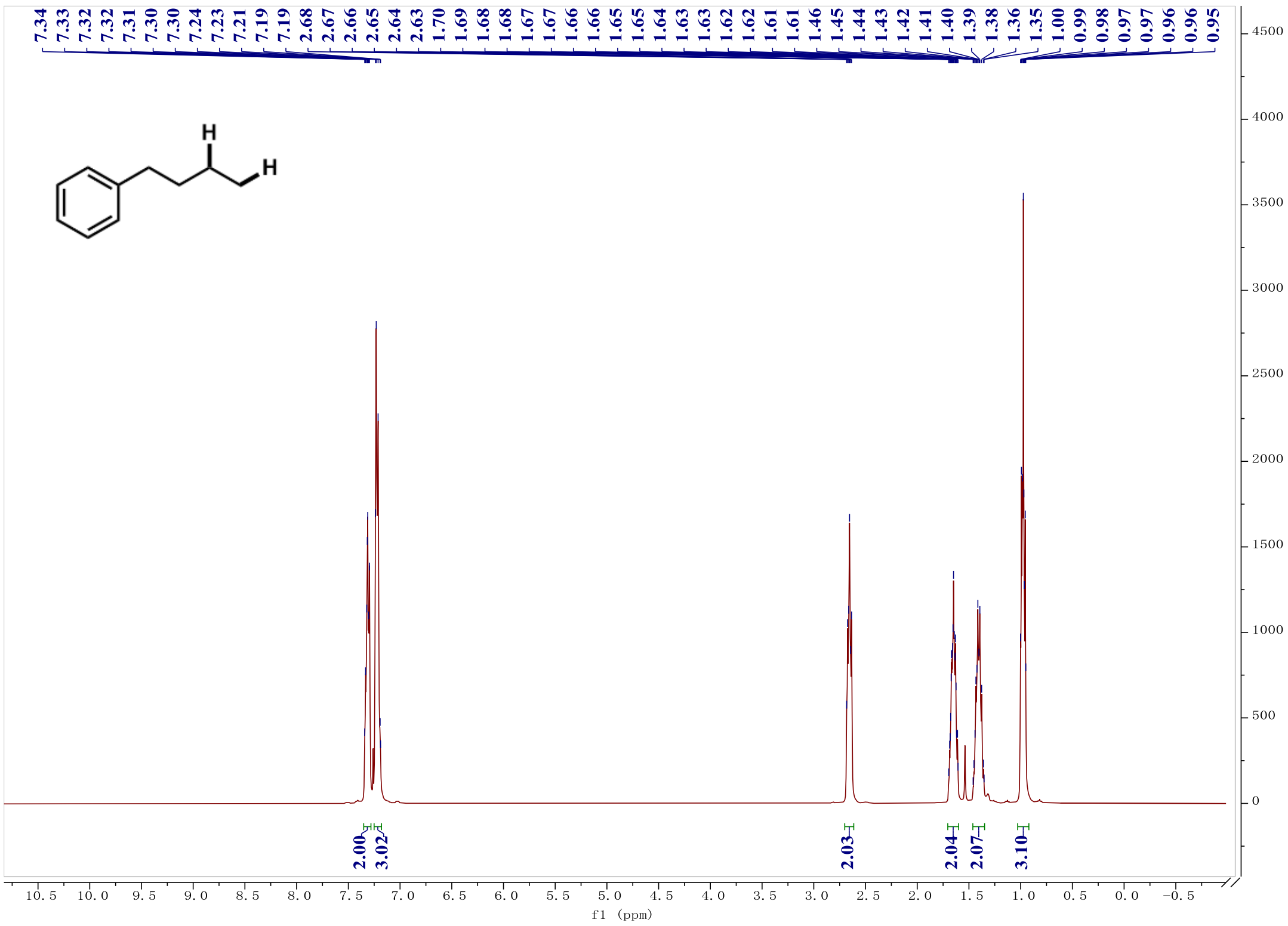

$^{1}$**H NMR** (400 MHz, $CDCl_3$) Spectrum of Compound **3r**

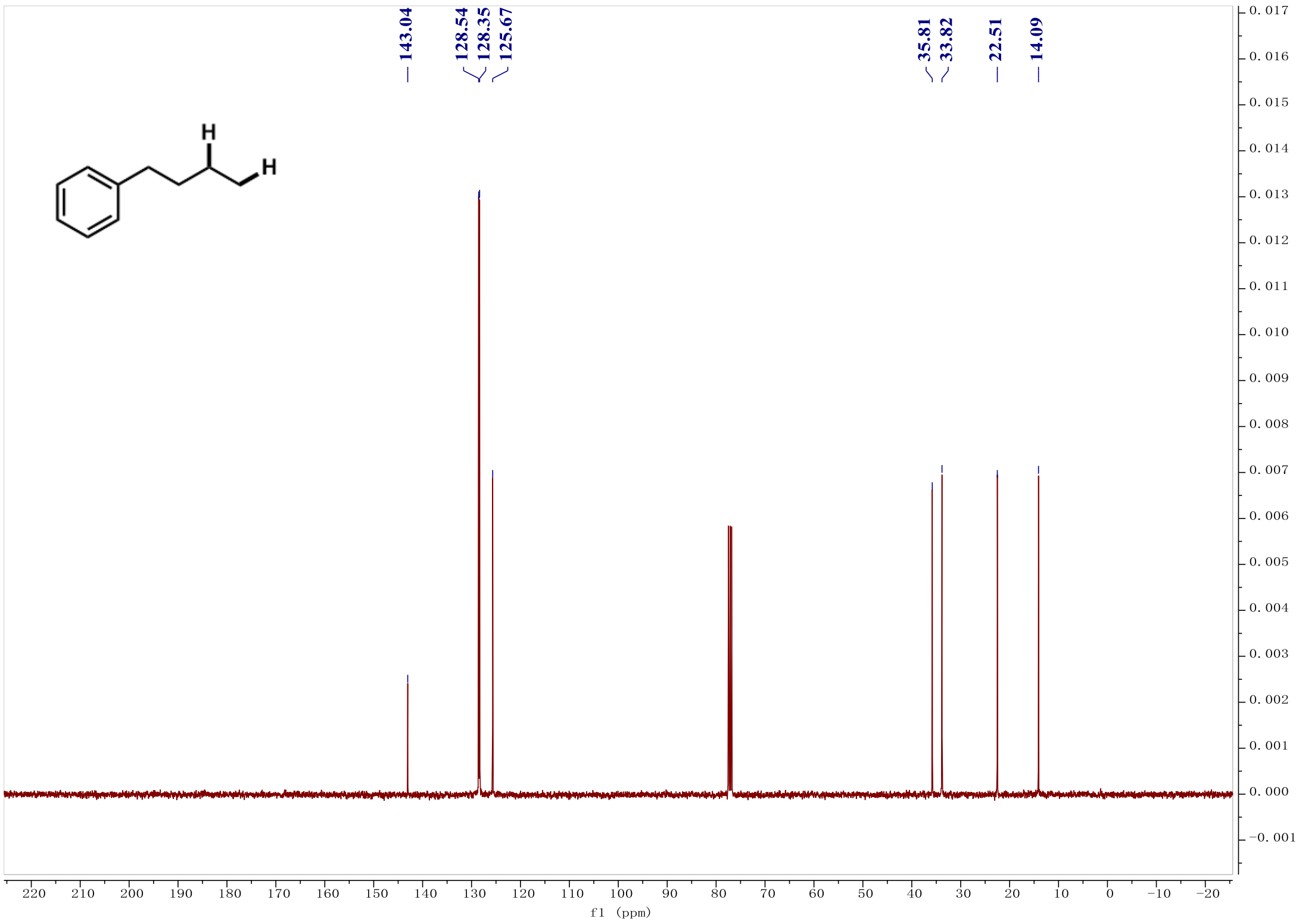

**$^{13}$C NMR** (101 MHz, $CDCl_3$) Spectrum of Compound **3r**

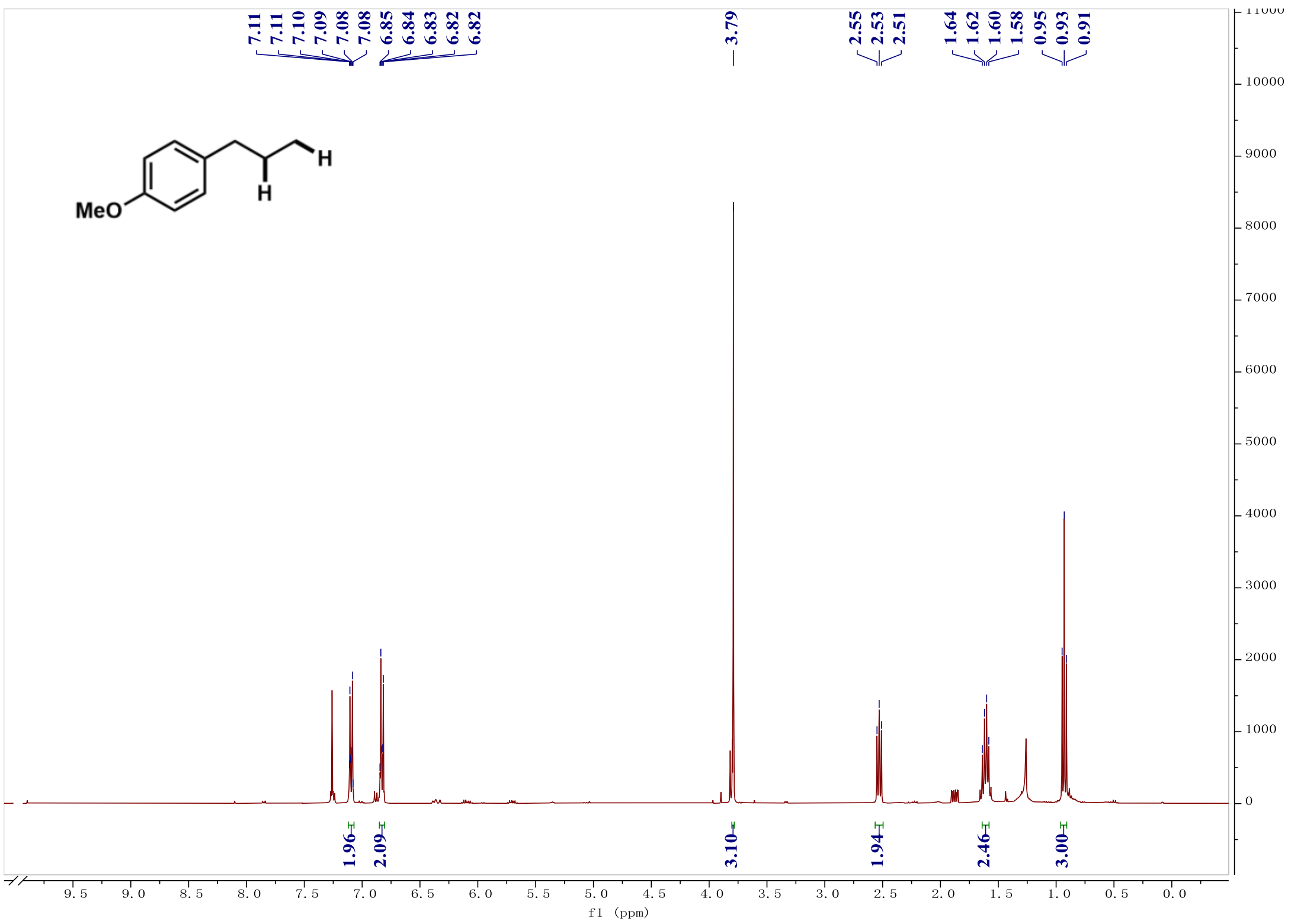

**$^{1}$H NMR** (400 MHz, $CDCl_3$) Spectrum of Compound **3s**

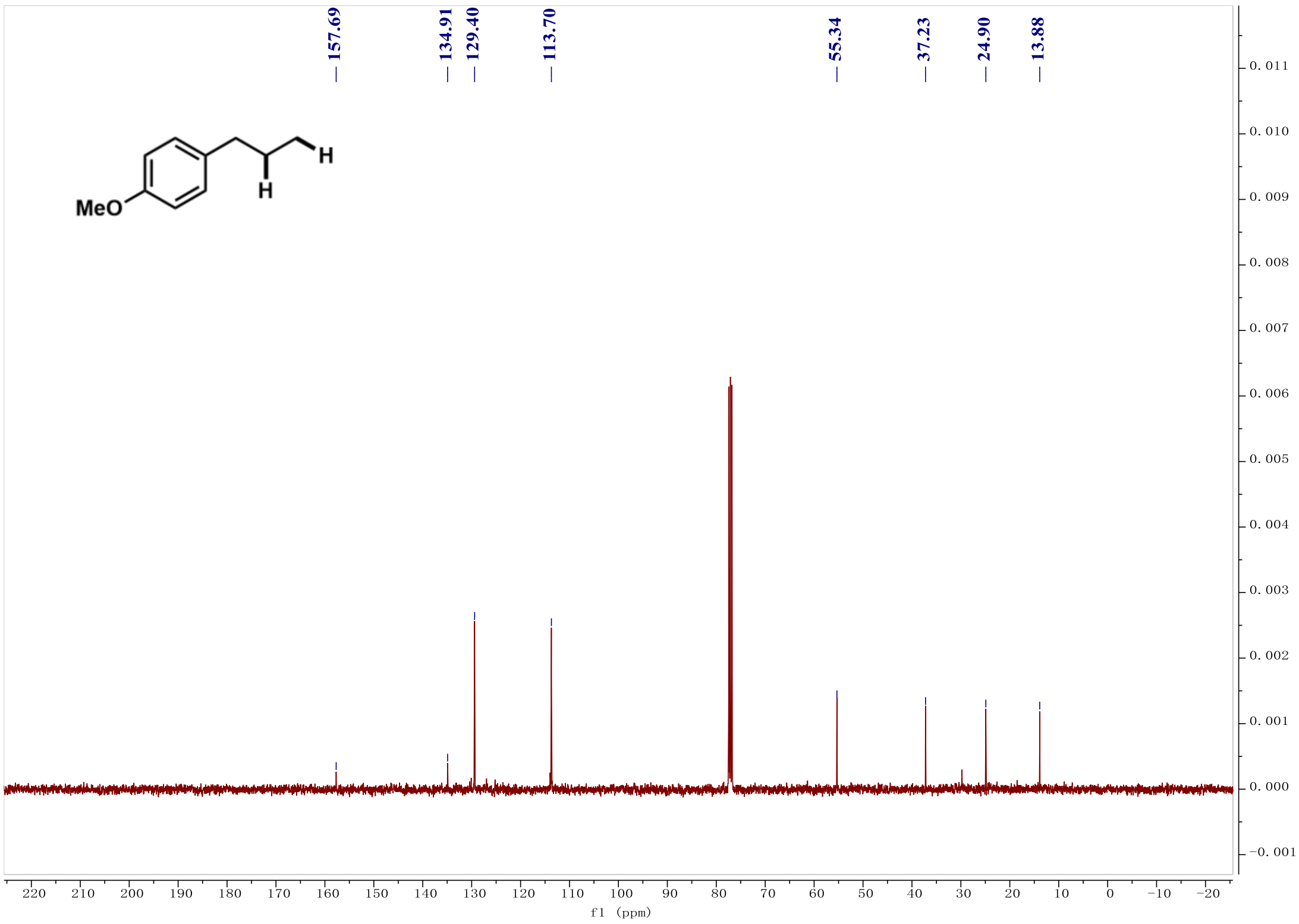


**$^{13}$C NMR** (101 MHz, $CDCl_3$) Spectrum of Compound **3s**

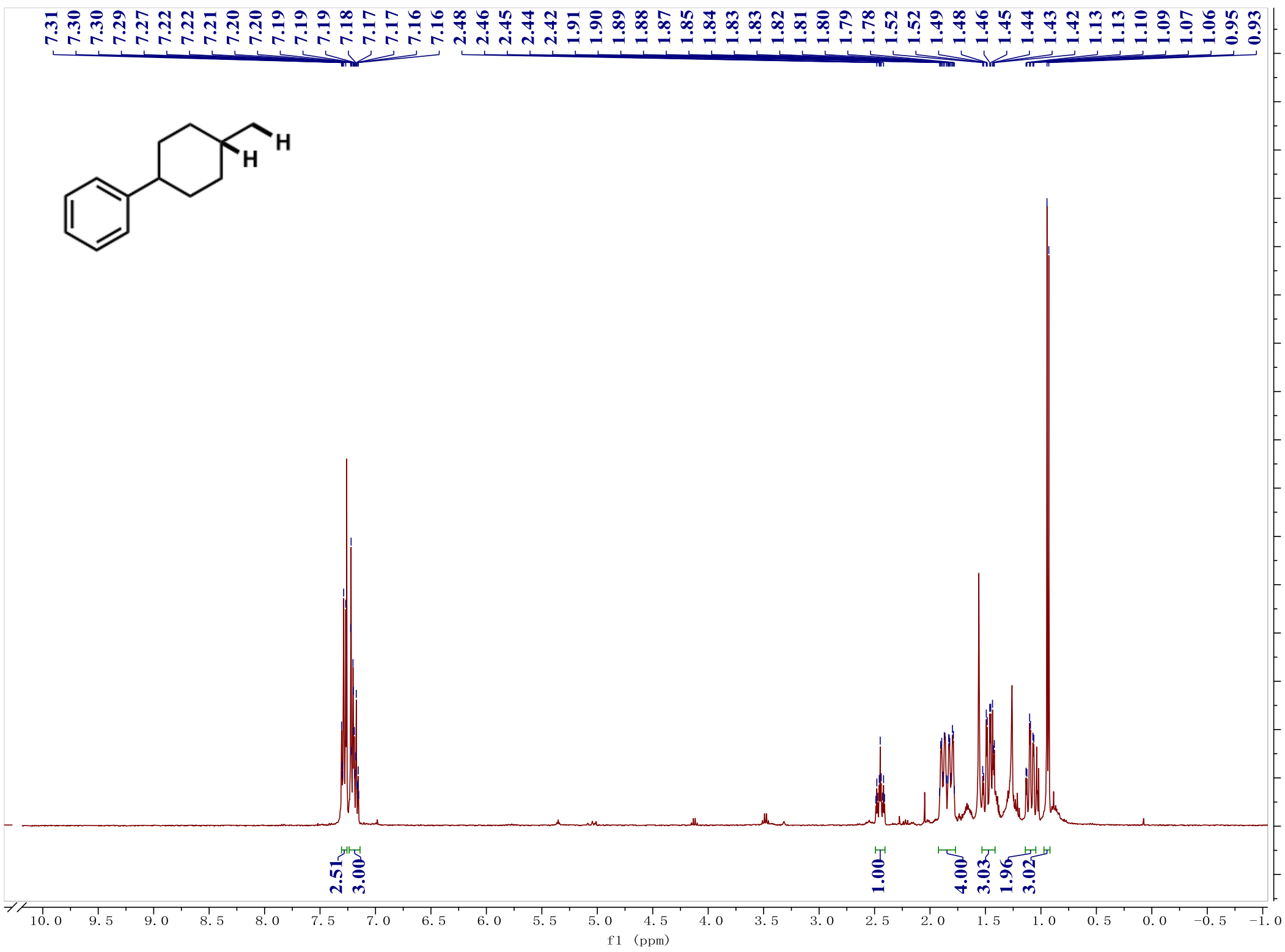


**$^{1}$H NMR** (400 MHz, $CDCl_3$) Spectrum of Compound **3t**

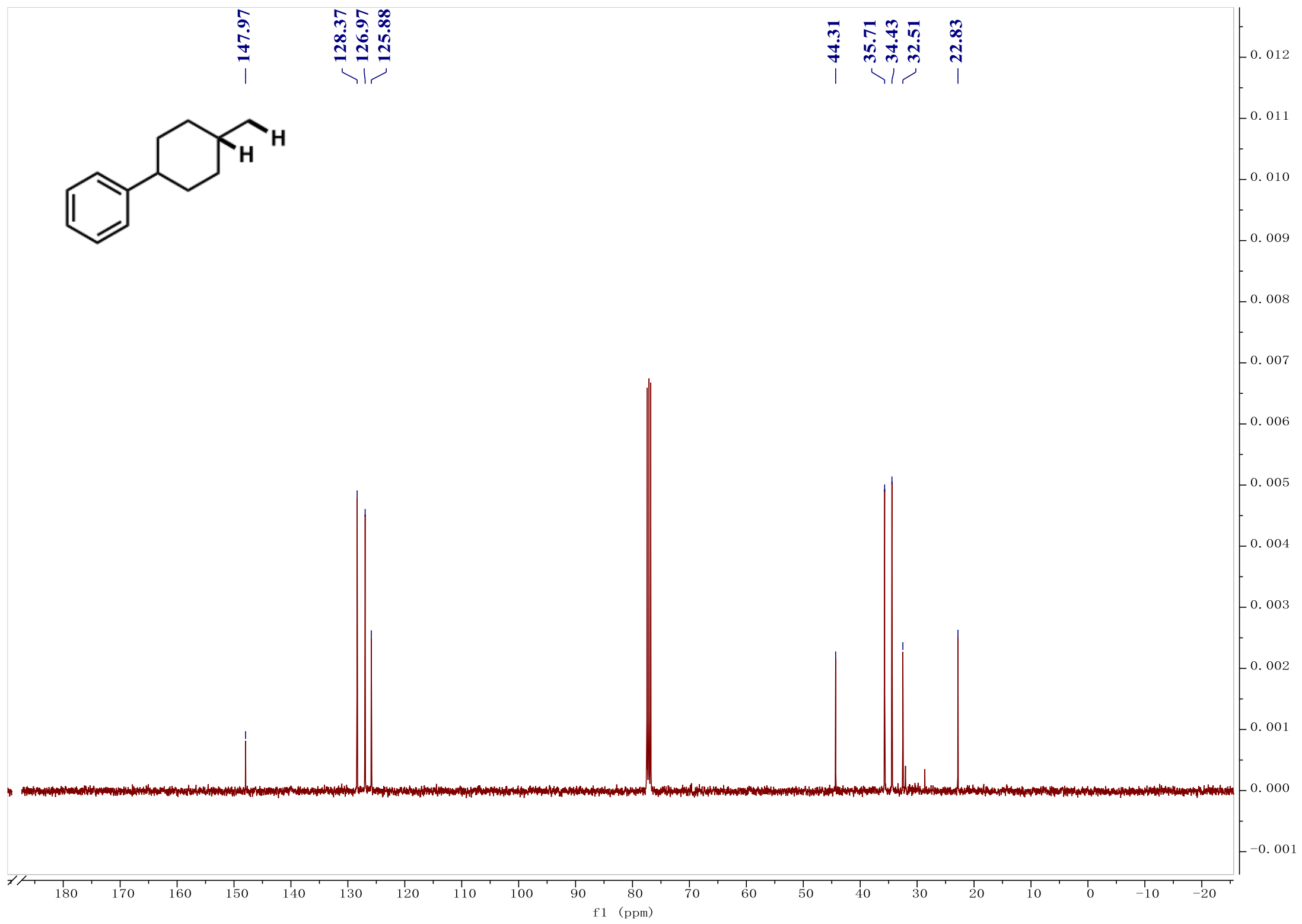


$^{13}$C **NMR** (101 MHz, CDCl$_3$) Spectrum of Compound **3t**

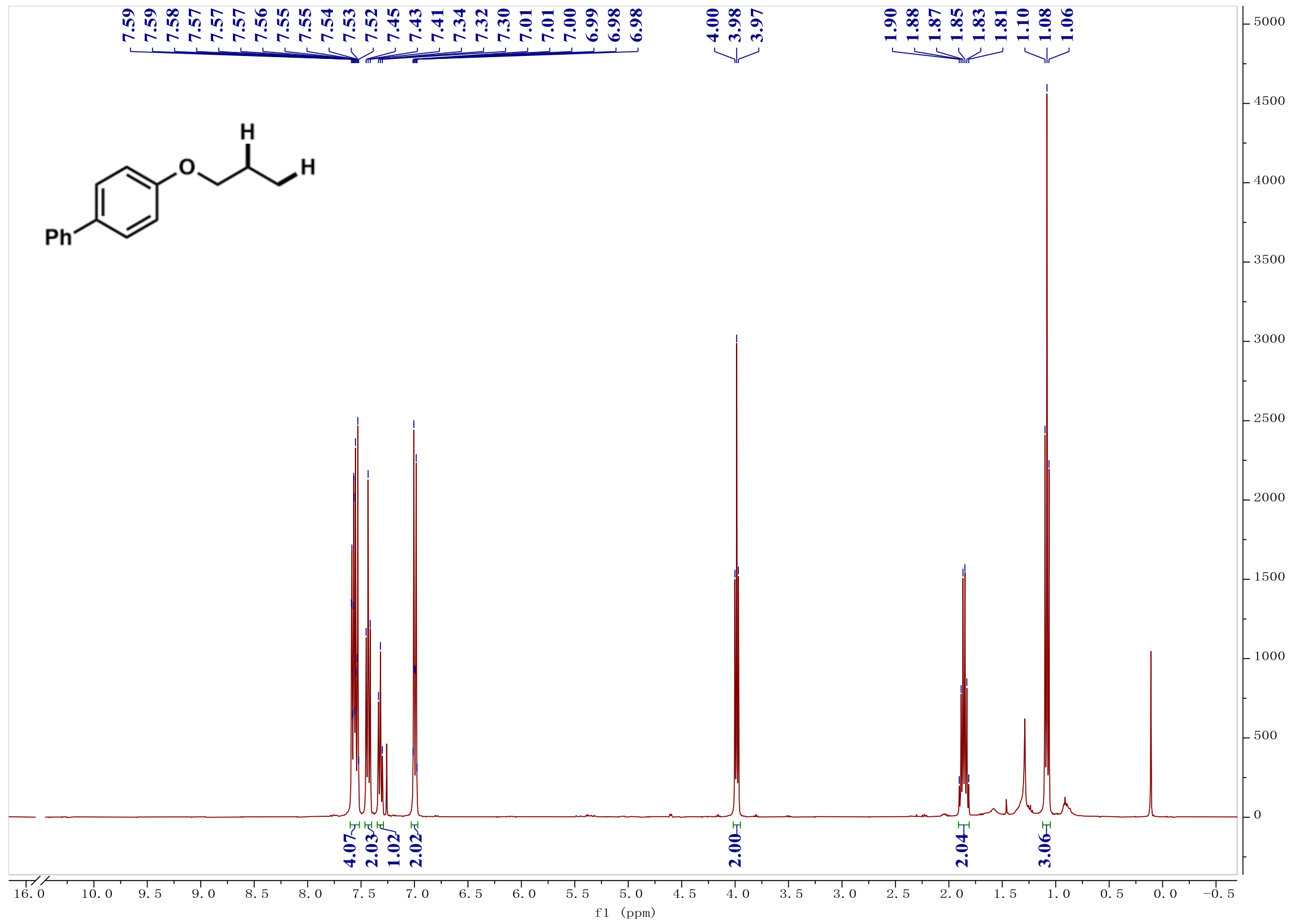


$^{1}$H **NMR** (400 MHz, CDCl$_3$) Spectrum of Compound **3u**

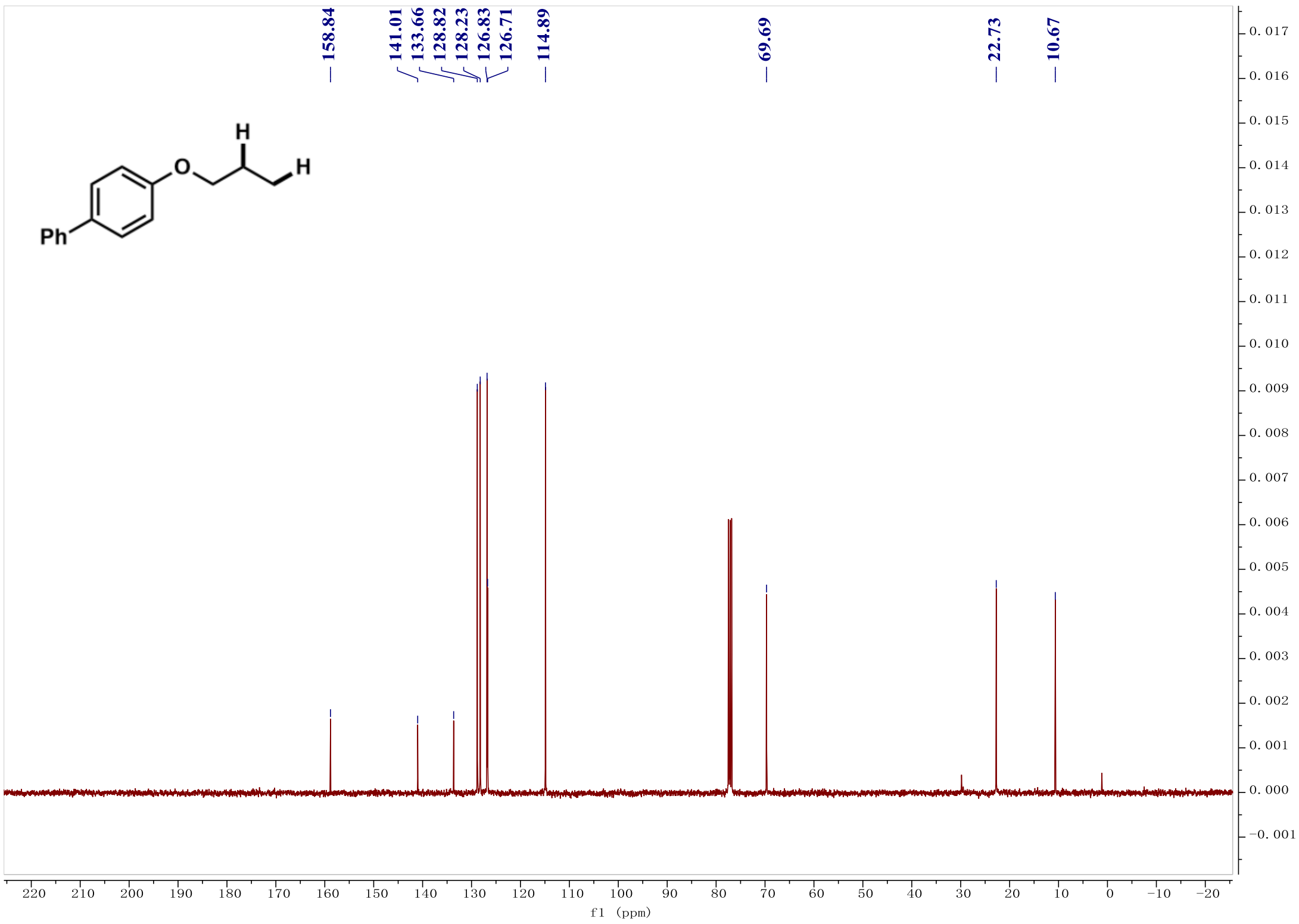

$^{13}$C NMR (101 MHz, $CDCl_3$) Spectrum of Compound **3u**

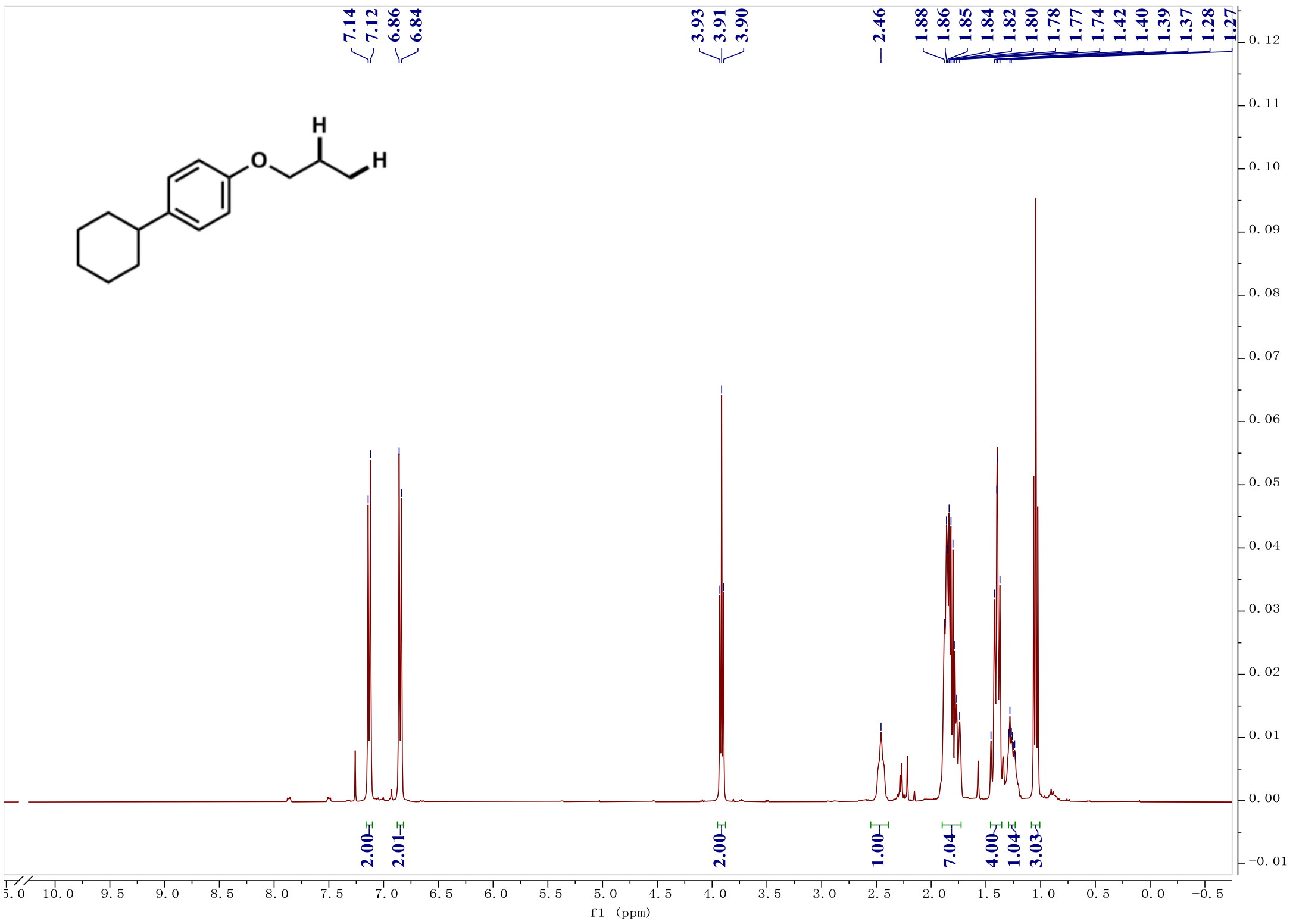

$^{1}$H NMR (400 MHz, $CDCl_3$) Spectrum of Compound **3v**

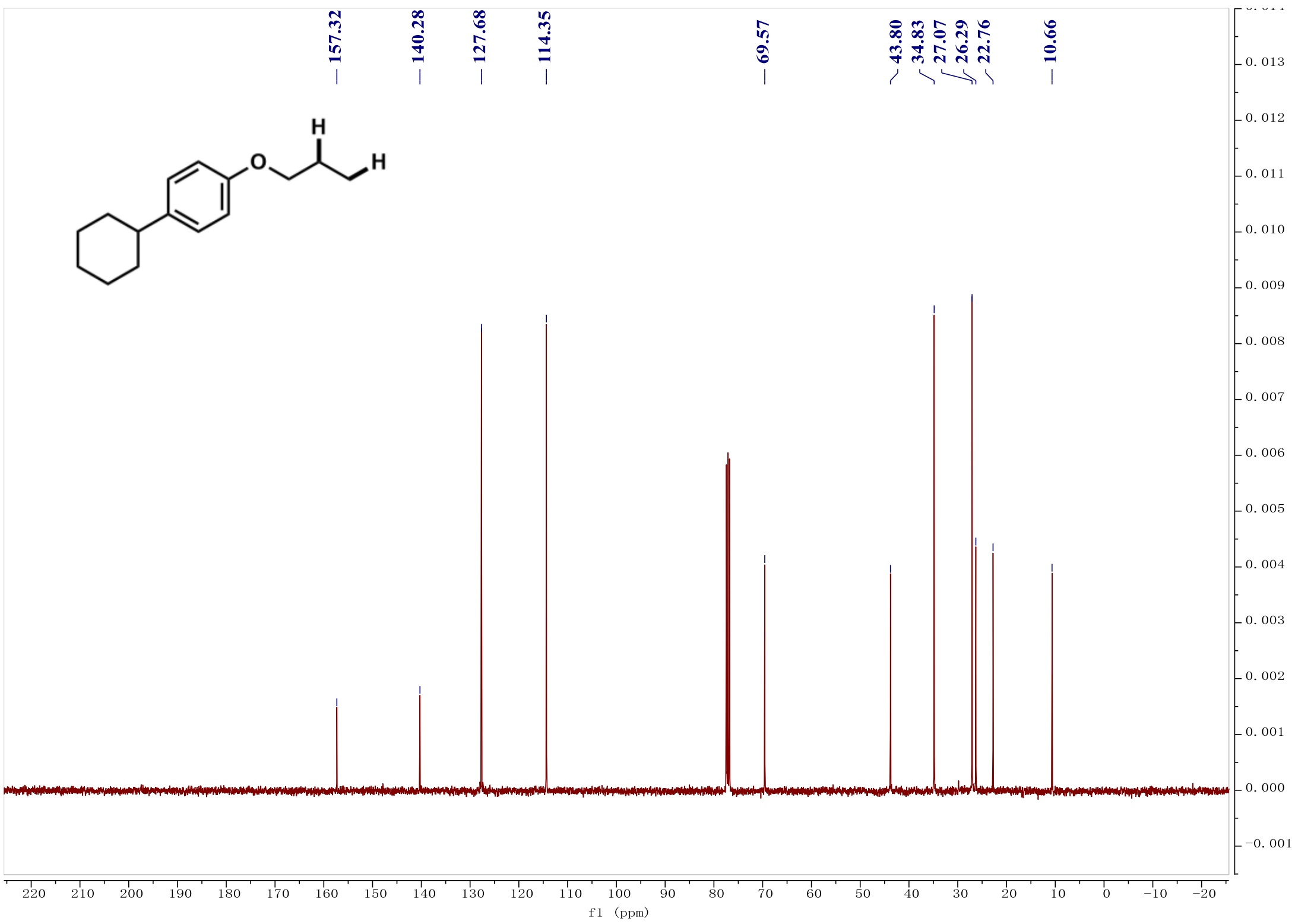


**$^{13}$C NMR** (101 MHz, $CDCl_3$) Spectrum of Compound **3v**

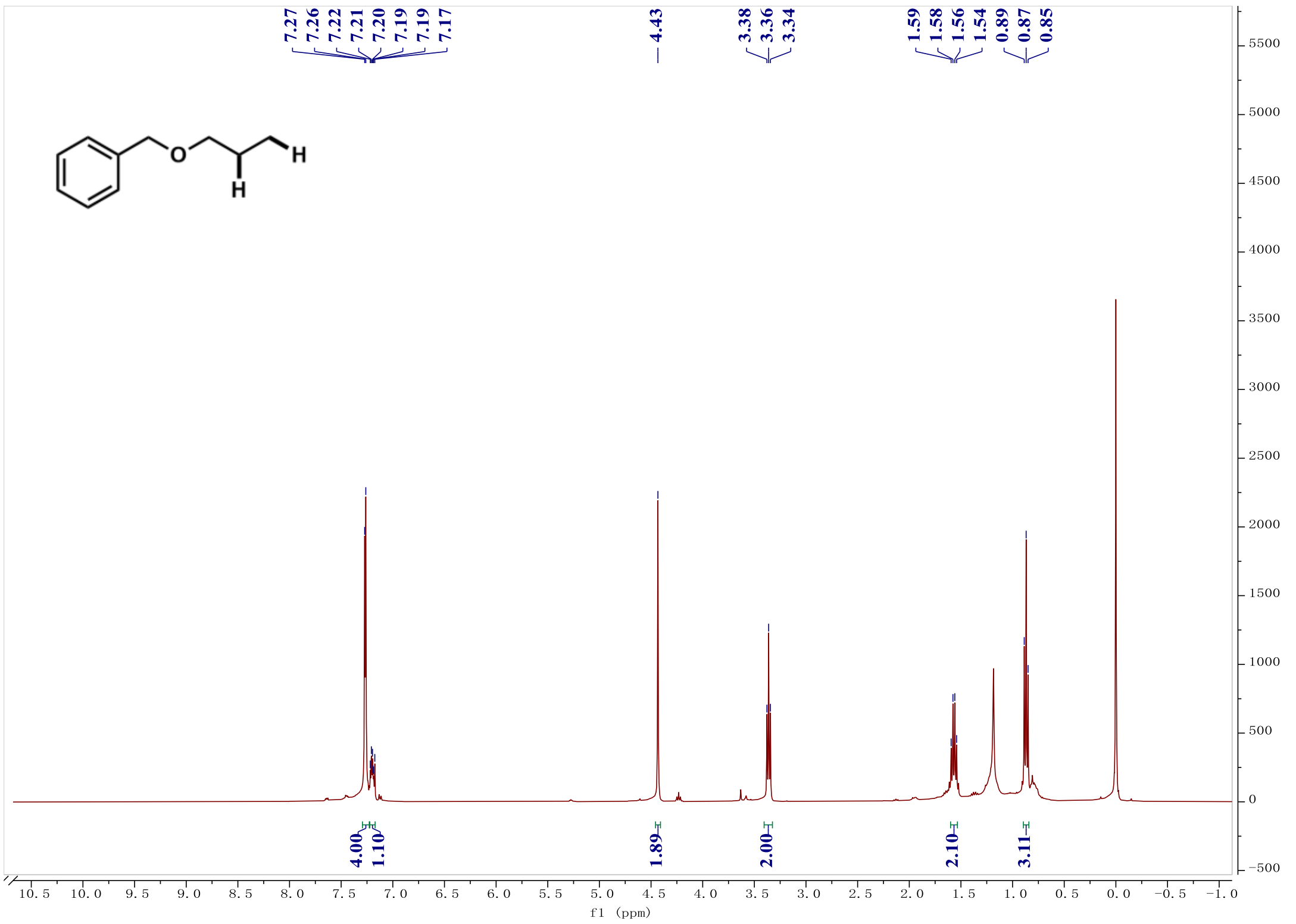


**$^{1}$H NMR** (400 MHz, $CDCl_3$) Spectrum of Compound **3w**

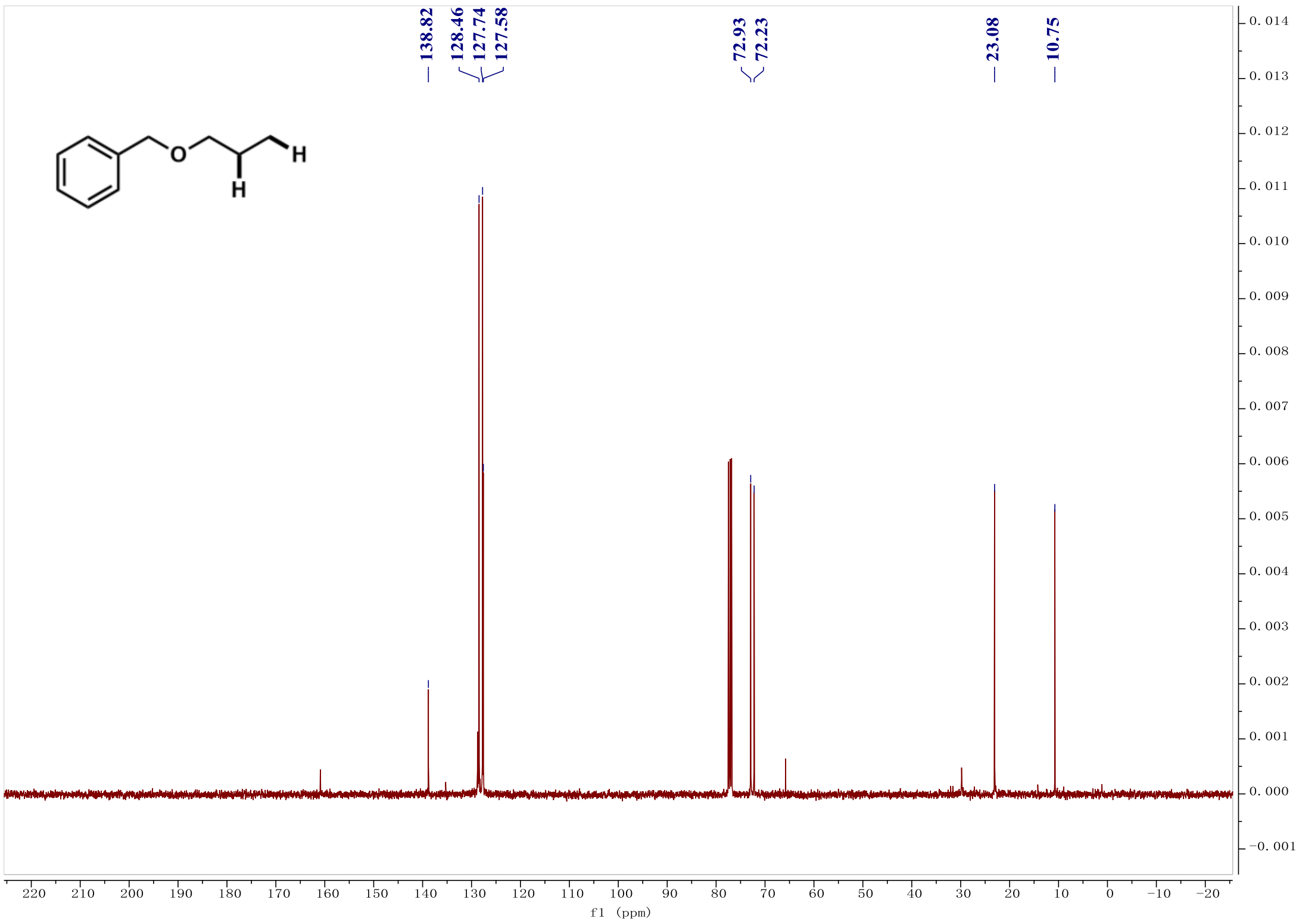


$^{13}$C **NMR** (101 MHz, $CDCl_3$) Spectrum of Compound **3w**

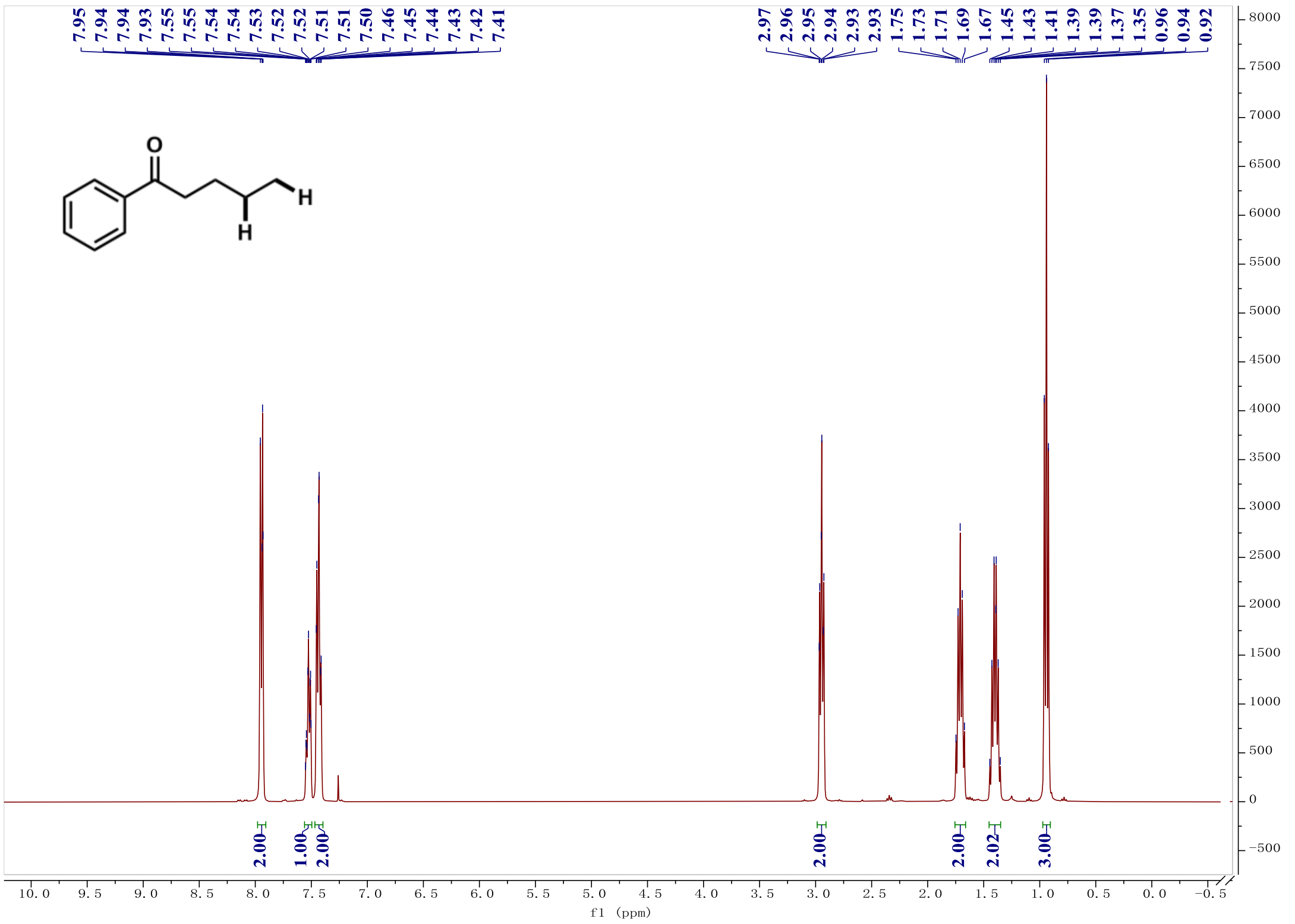


$^{1}$H **NMR** (400 MHz, $CDCl_3$) Spectrum of Compound **3x**

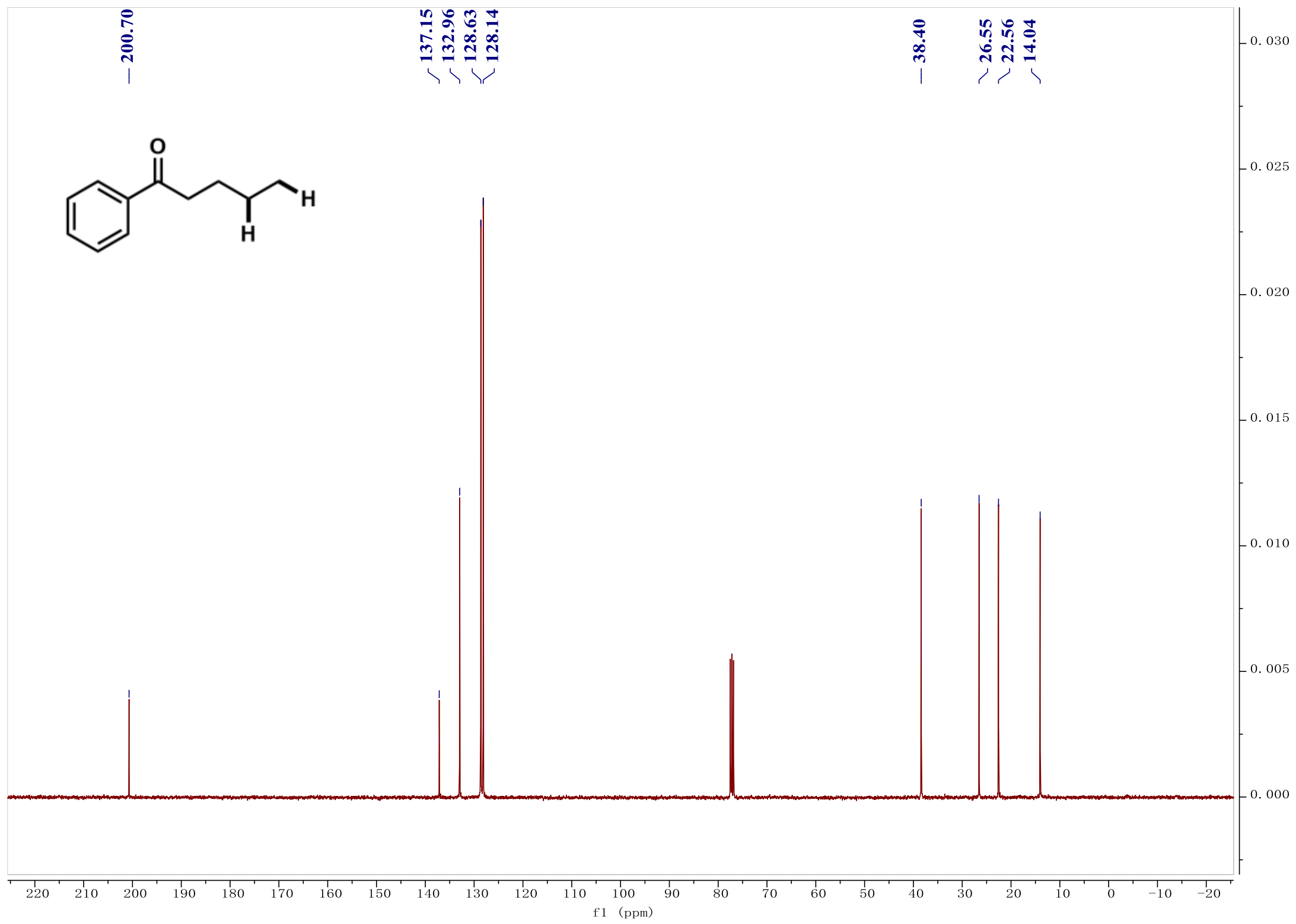


**$^{13}$C NMR** (101 MHz, CDCl$_3$) Spectrum of Compound **3x**

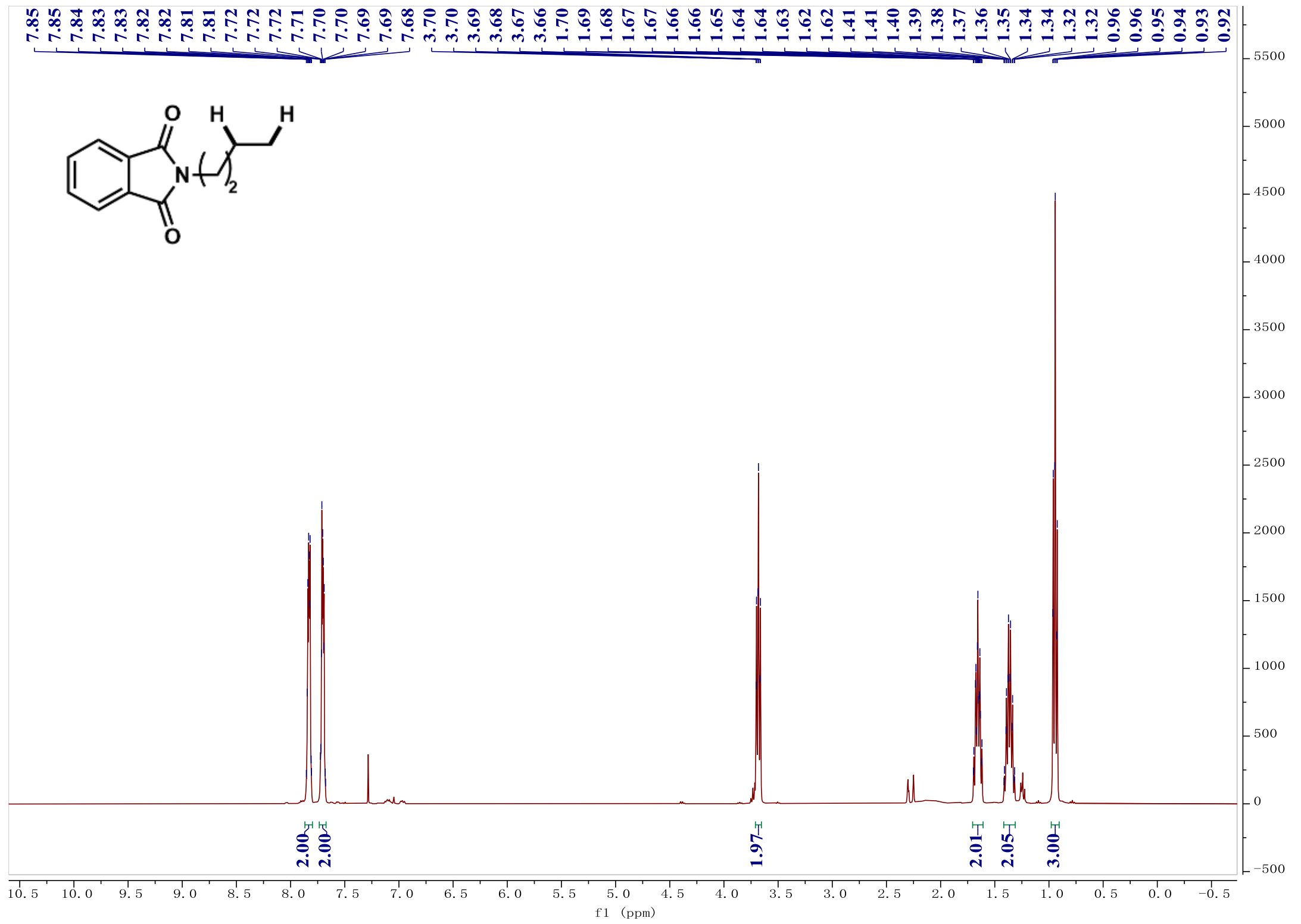


**$^{1}$H NMR** (400 MHz, CDCl$_3$) Spectrum of Compound **3y**

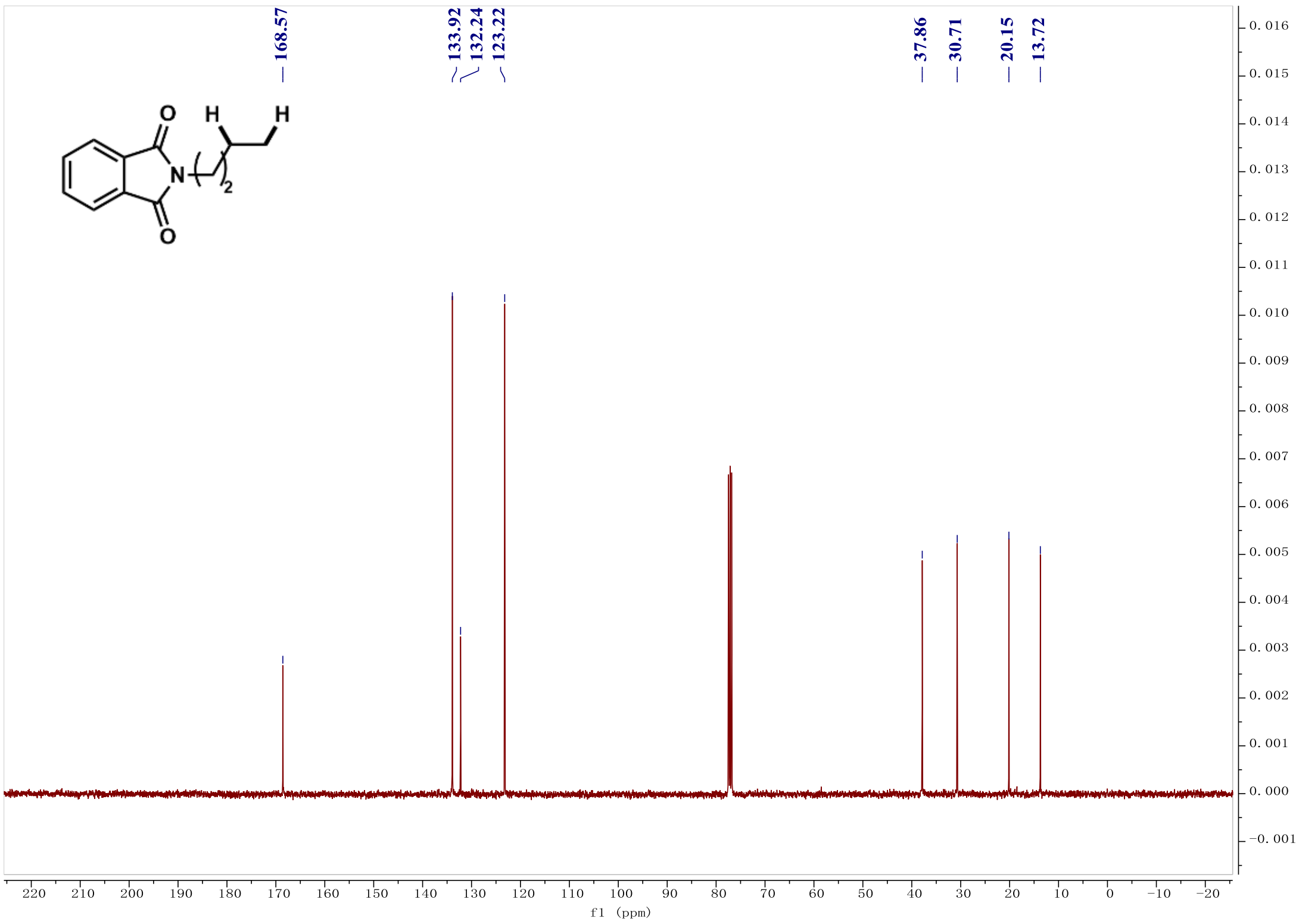

$^{13}$**C NMR** (101 MHz, CDCl$_3$) Spectrum of Compound **3y**

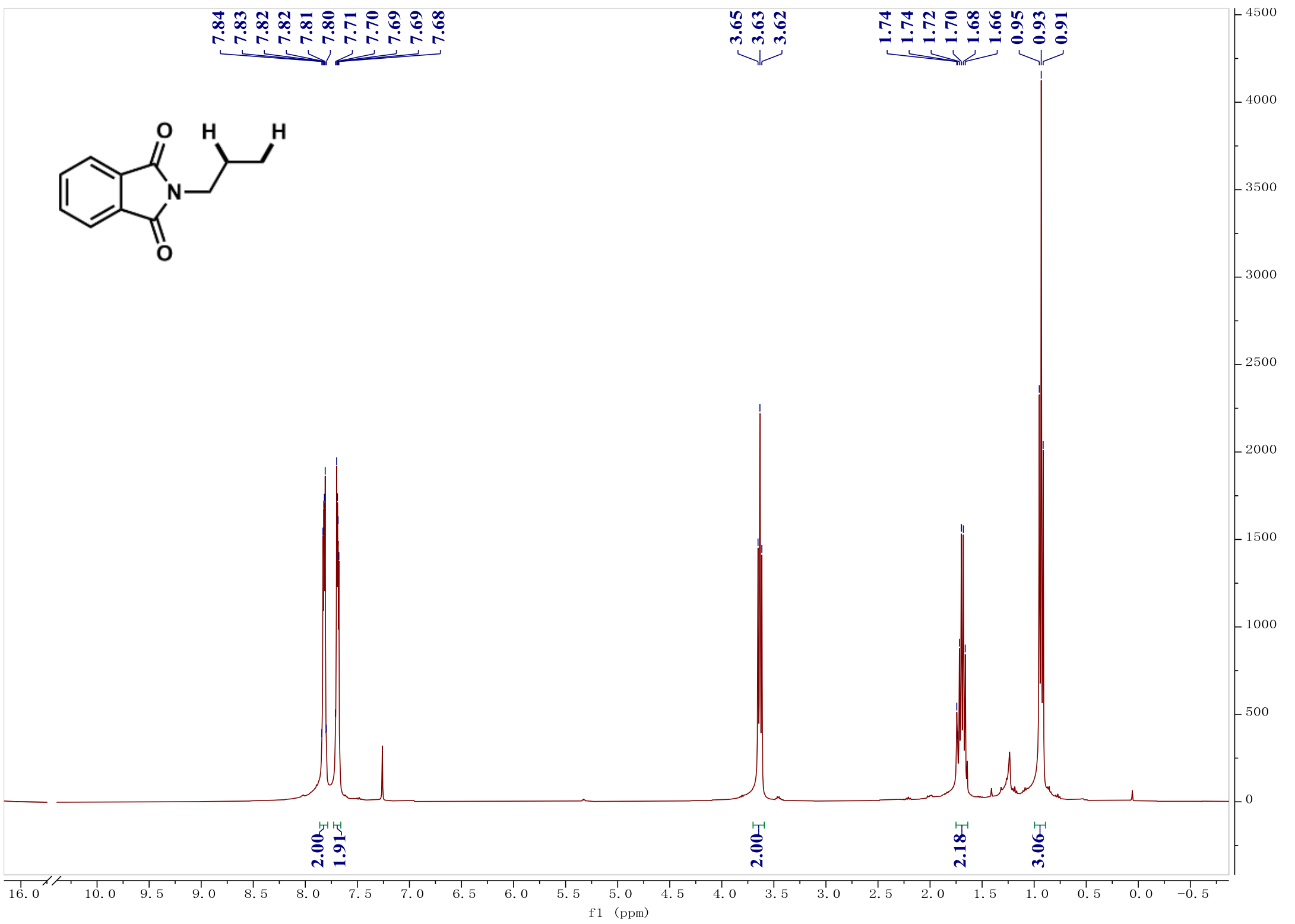

$^{1}$**H NMR** (400 MHz, CDCl$_3$) Spectrum of Compound **3z**

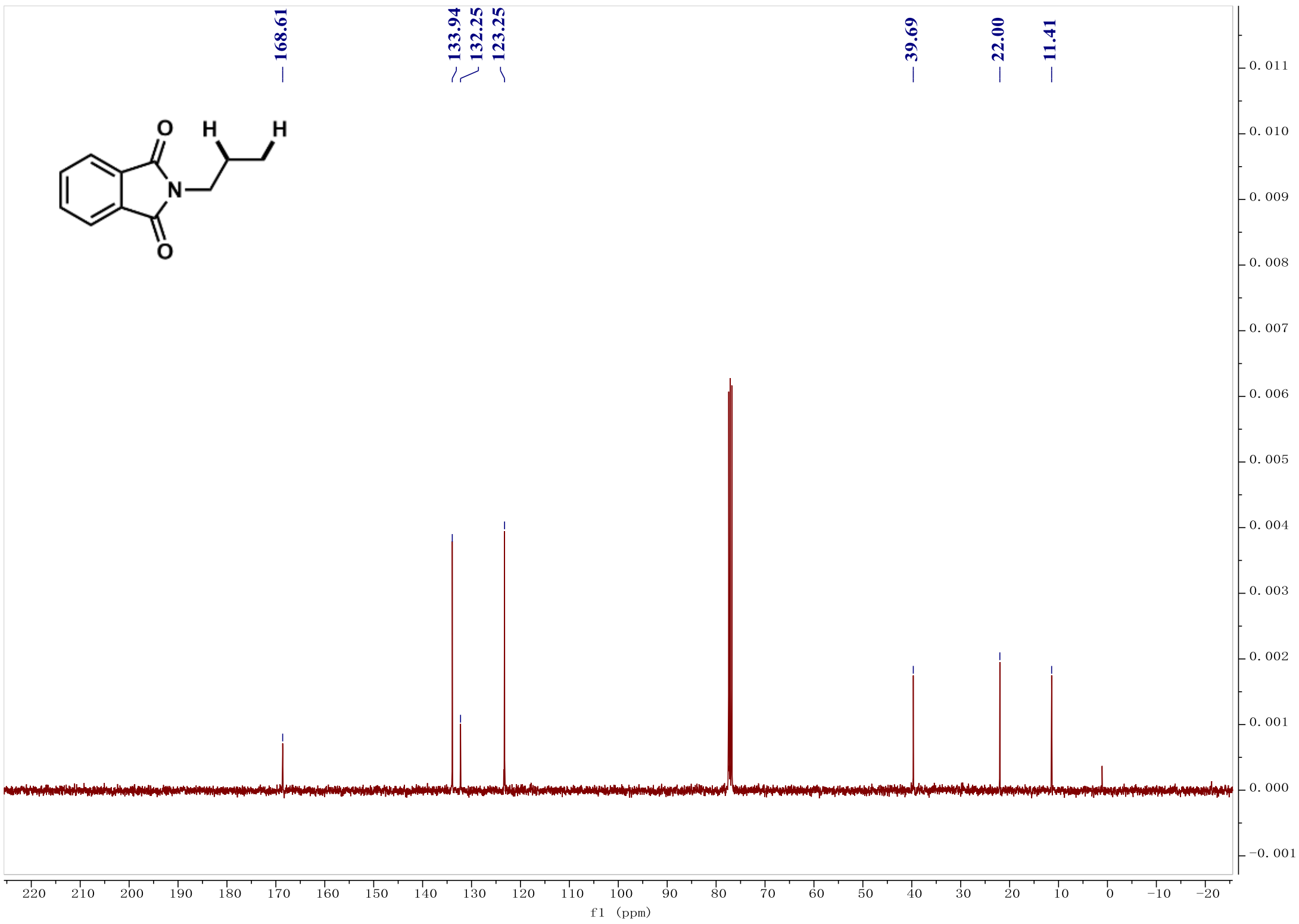


$^{1}$**H NMR** (400 MHz, $CDCl_3$) Spectrum of Compound **3z**

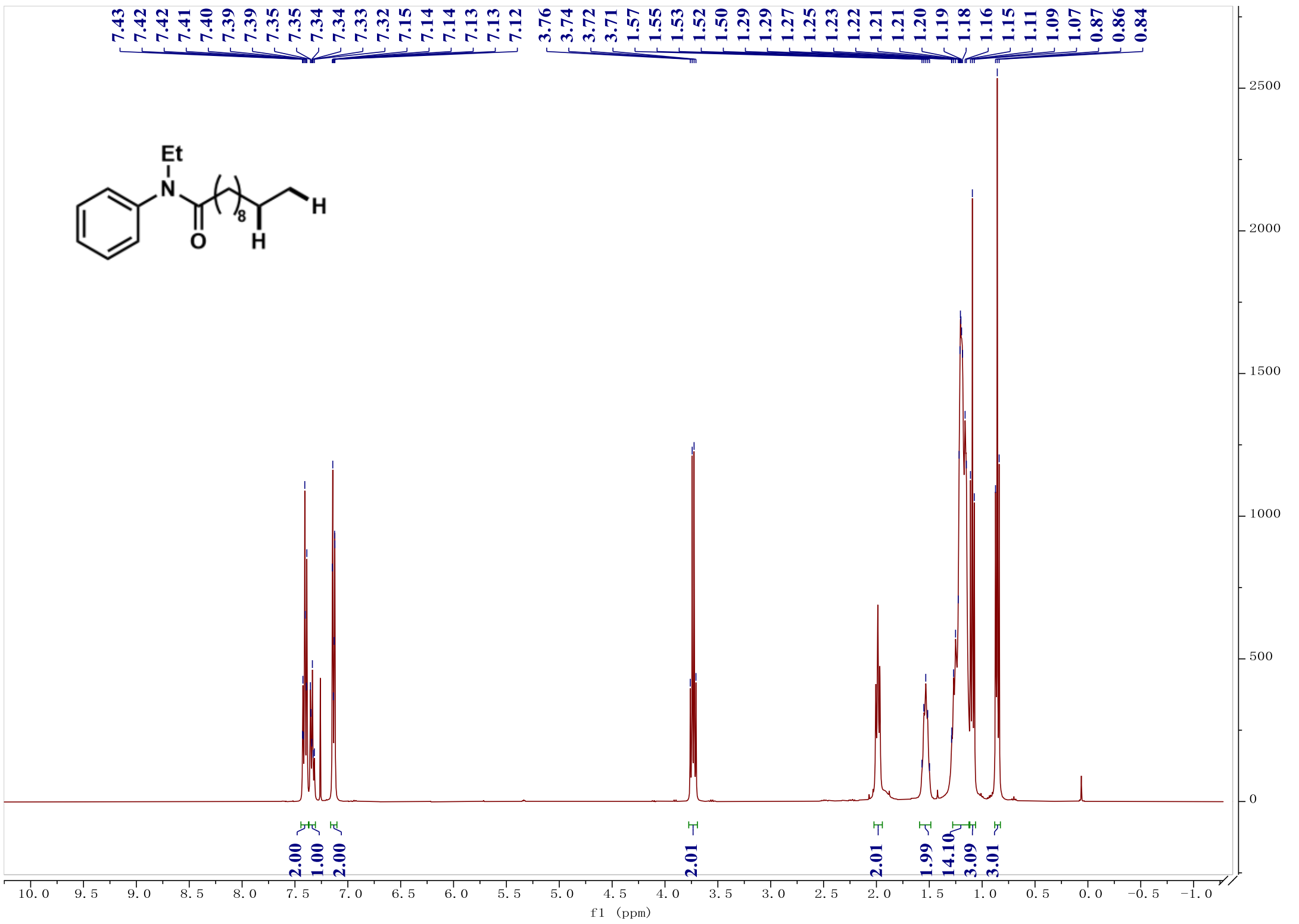


$^{1}$**H NMR** (400 MHz, $CDCl_3$) Spectrum of Compound **3aa**

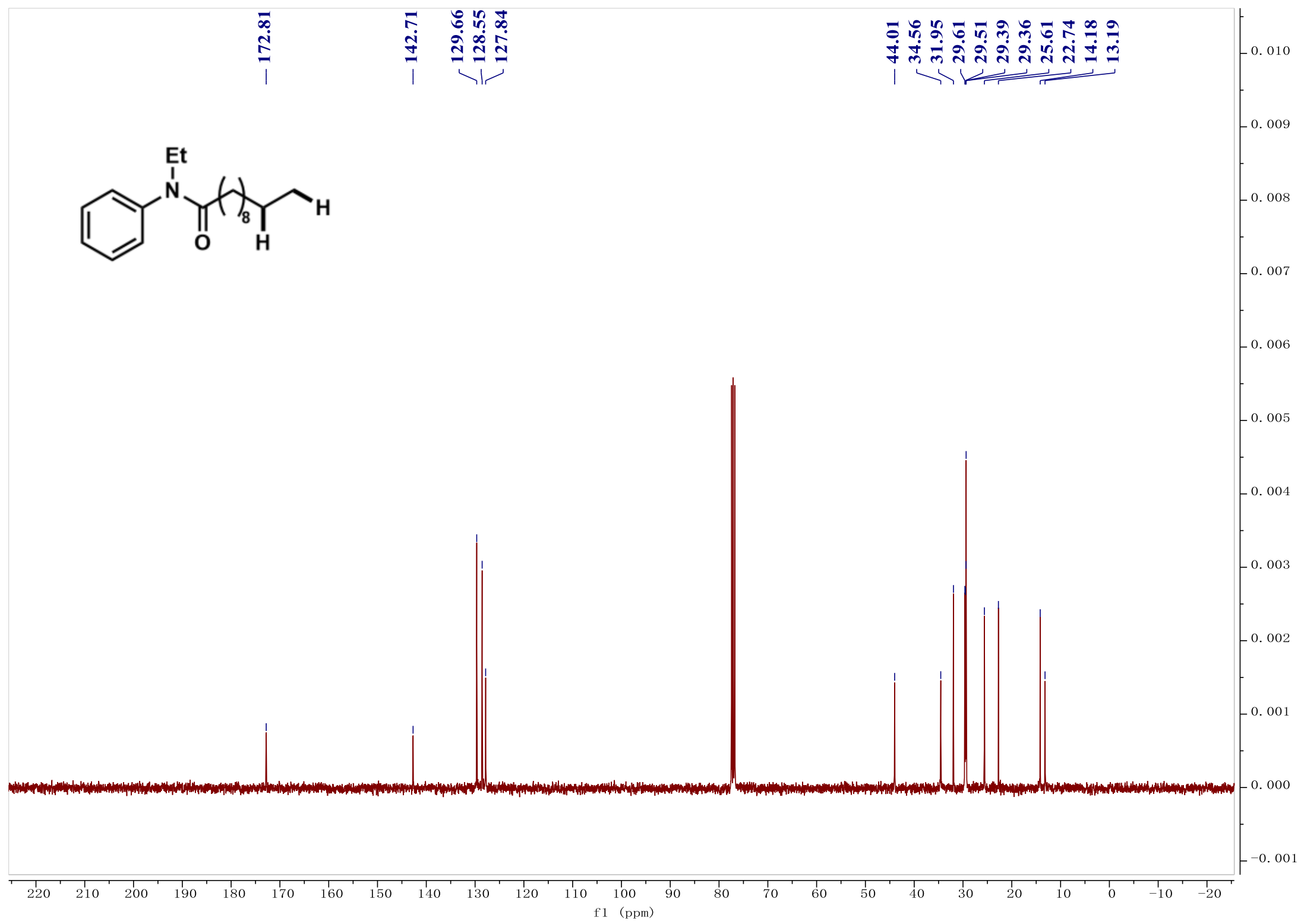

**$^{13}$C NMR** (101 MHz, CDCl$_3$) Spectrum of Compound **3aa**

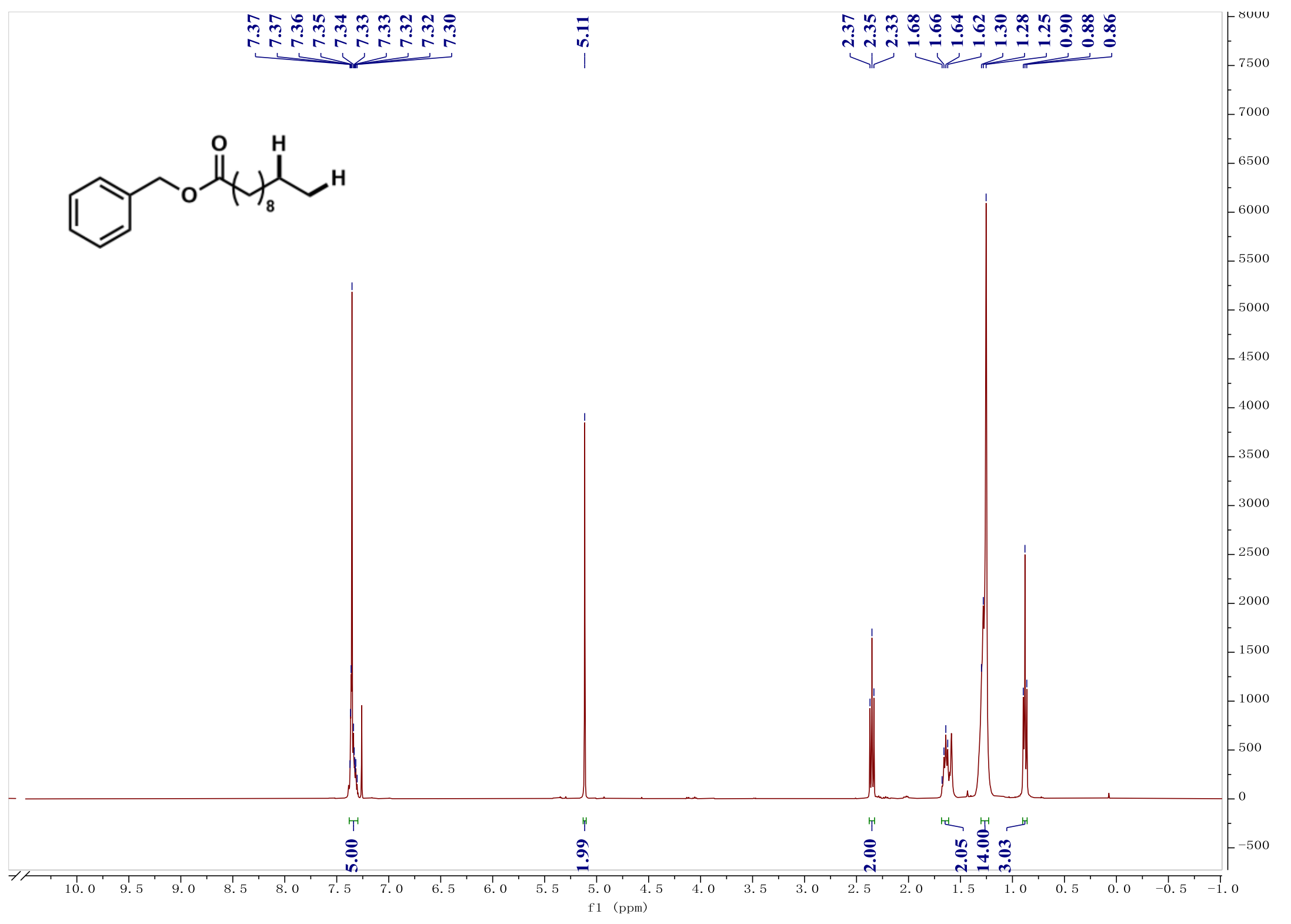

**$^{1}$H NMR** (400 MHz, CDCl$_3$) Spectrum of Compound **3ab**

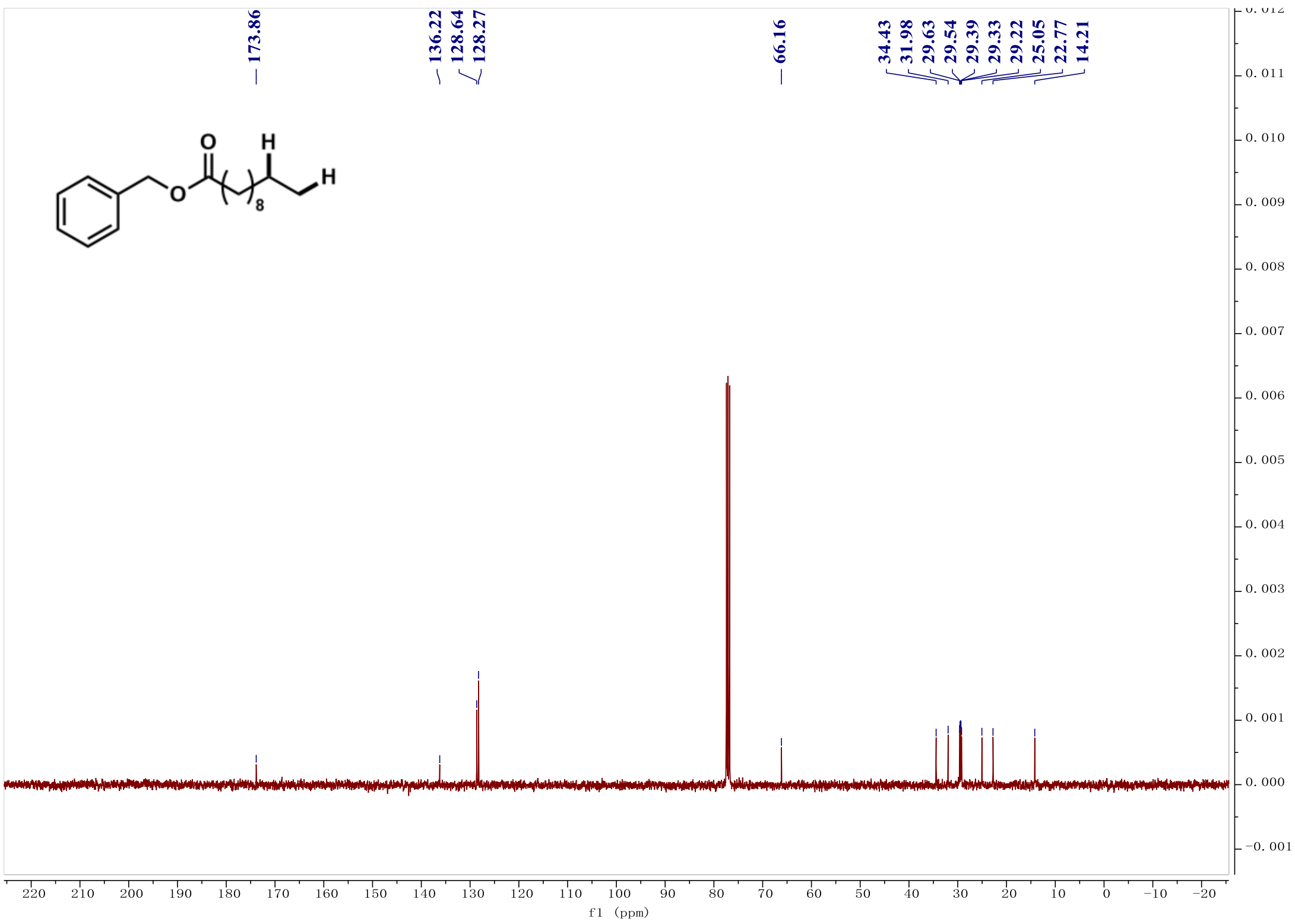

$^{13}$C NMR (101 MHz, CDCl$_3$) Spectrum of Compound **3ab**

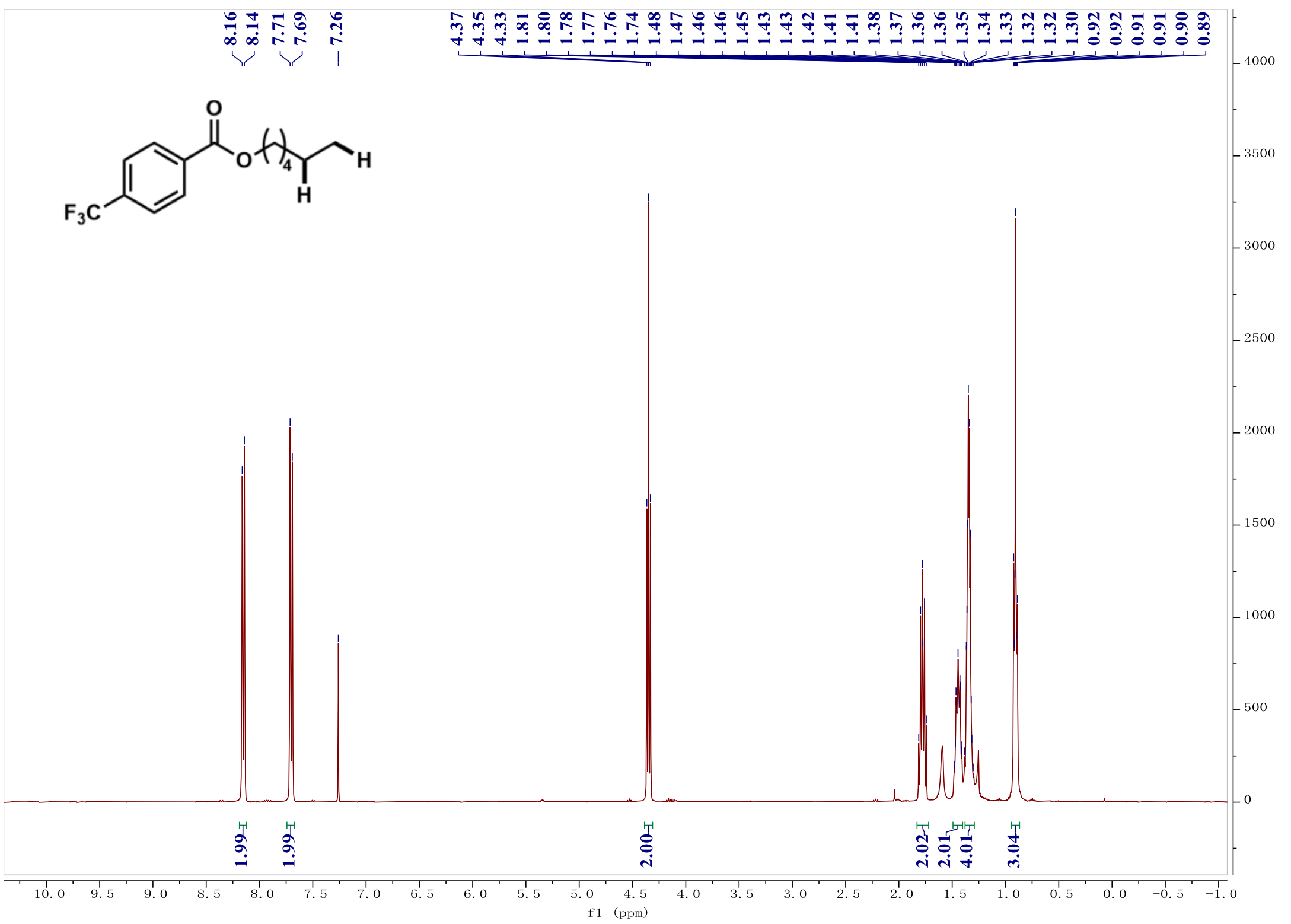

$^{1}$H NMR (400 MHz, CDCl$_3$) Spectrum of Compound **3ac**

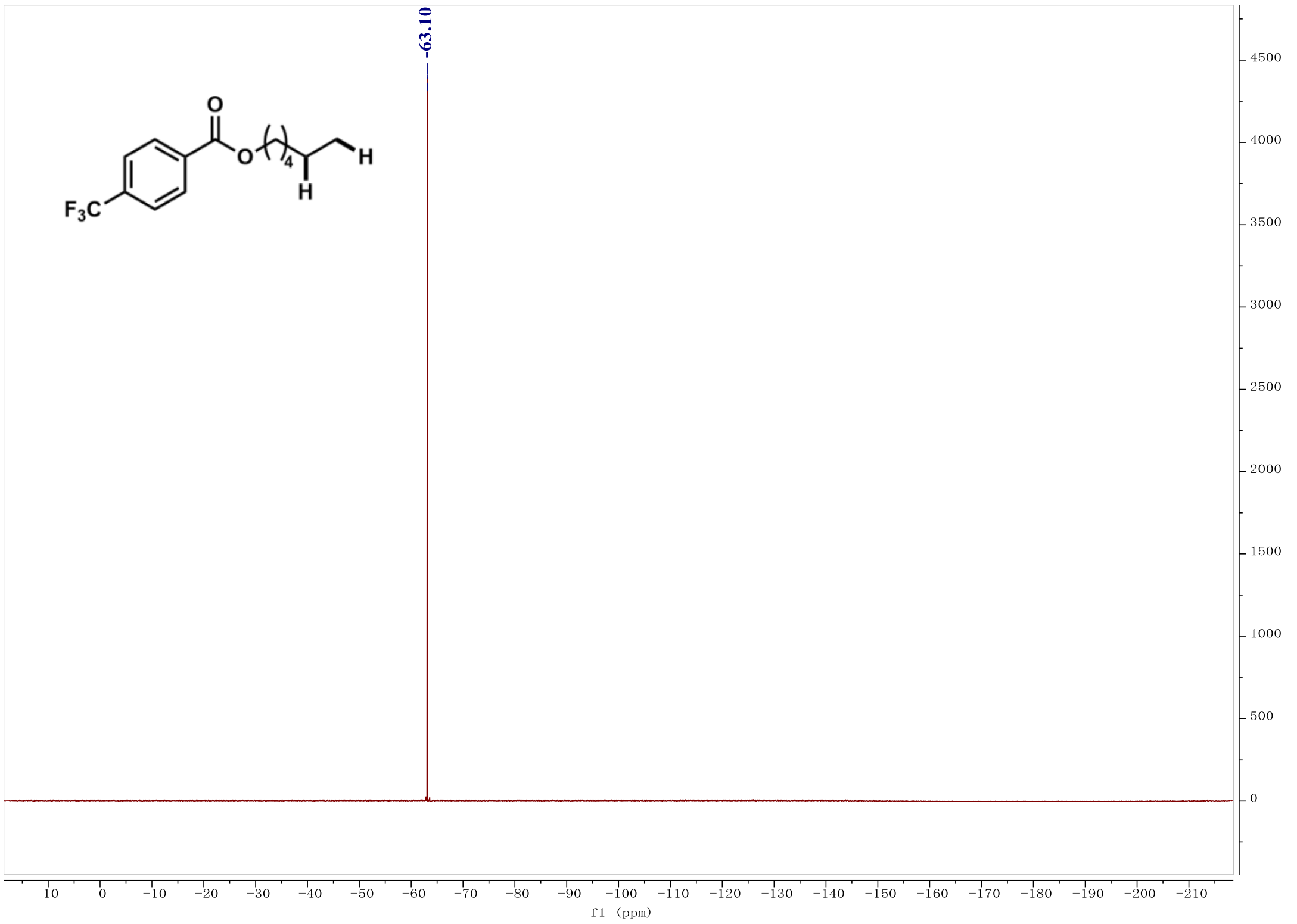


**$^{19}$F NMR** (376 MHz, $CDCl_3$) Spectrum of Compound **3ac**

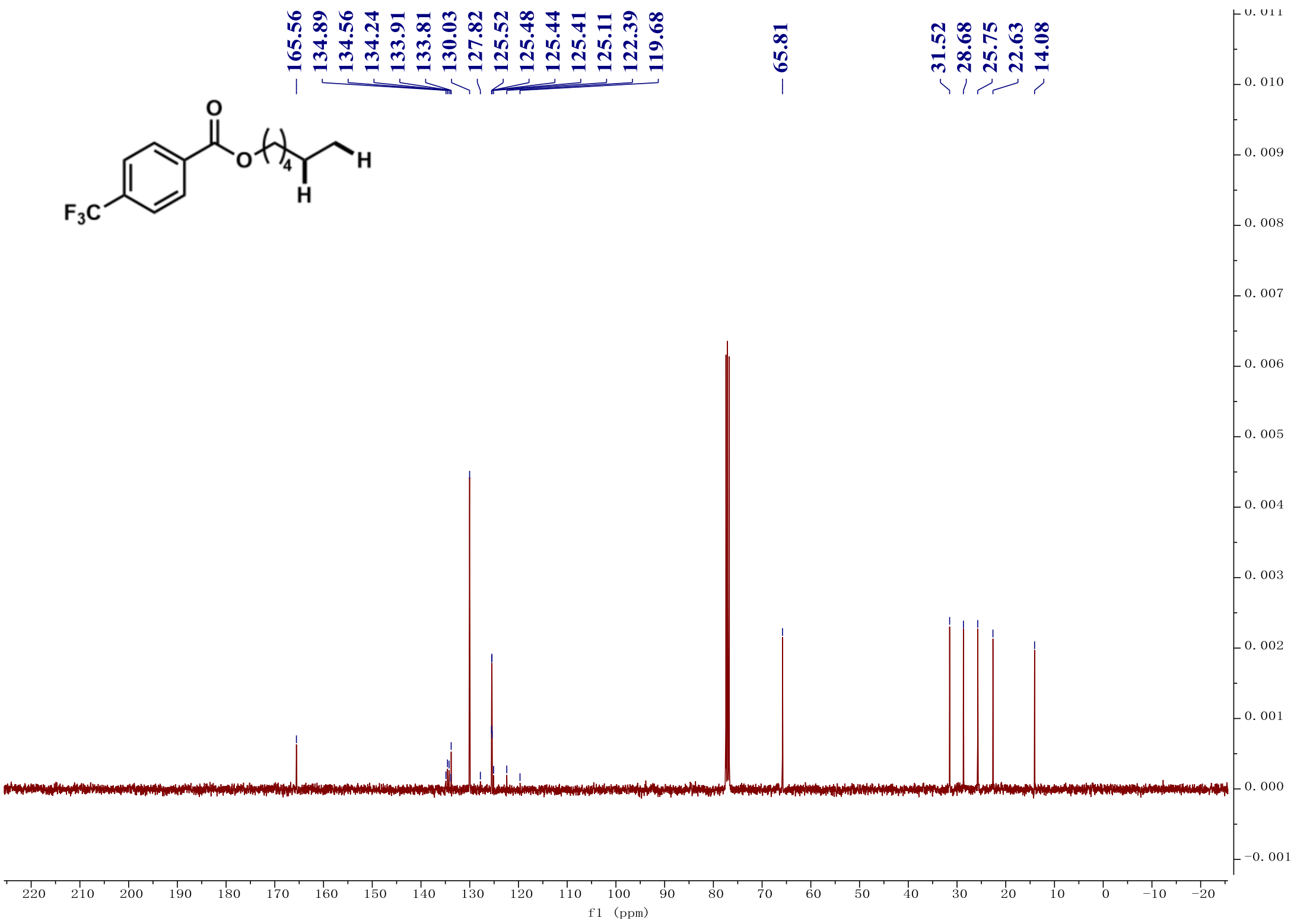


**$^{13}$C NMR** (101 MHz, $CDCl_3$) Spectrum of Compound **3ac**

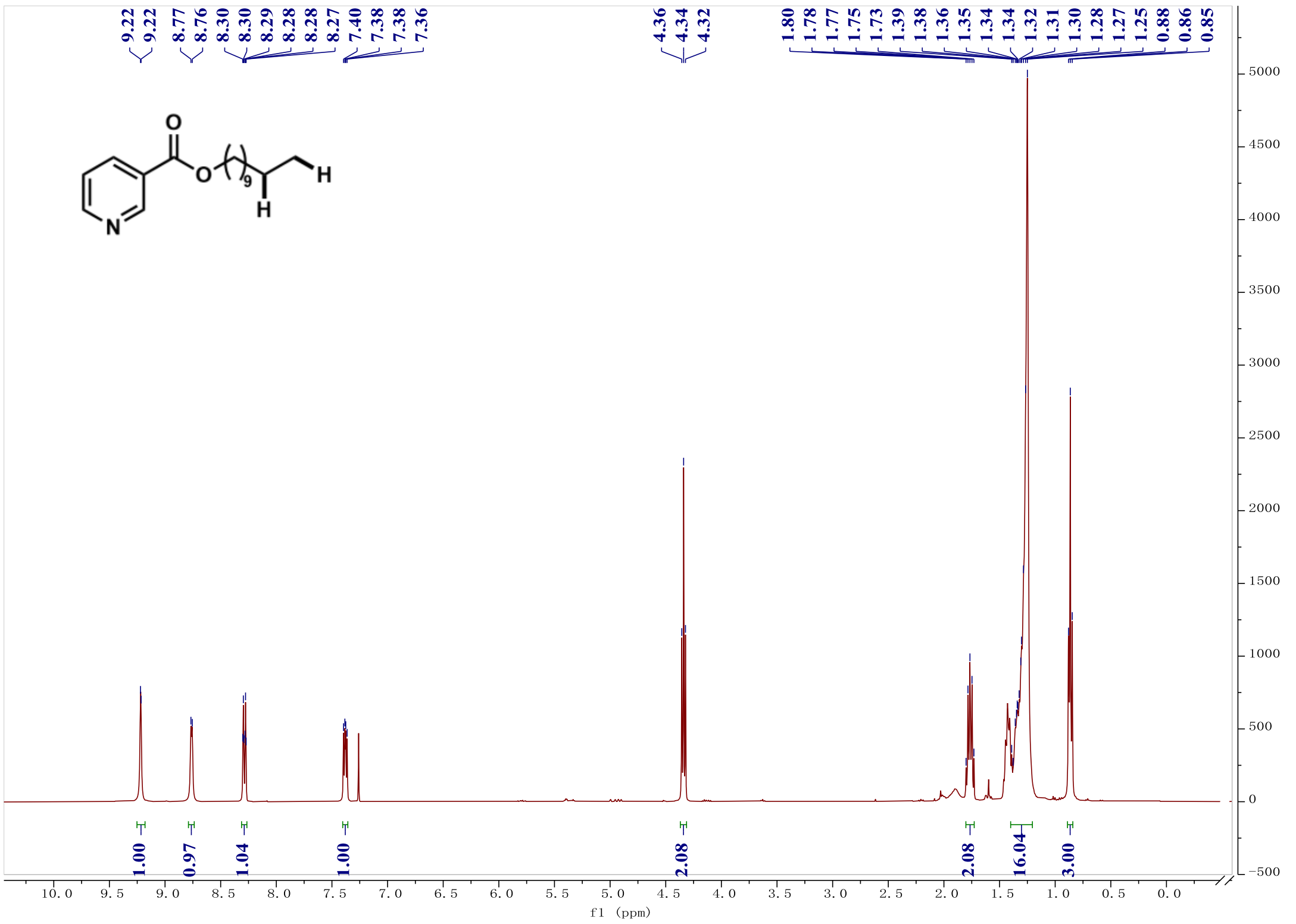


$^{1}$**H NMR** (400 MHz, $CDCl_3$) Spectrum of Compound **3ad**

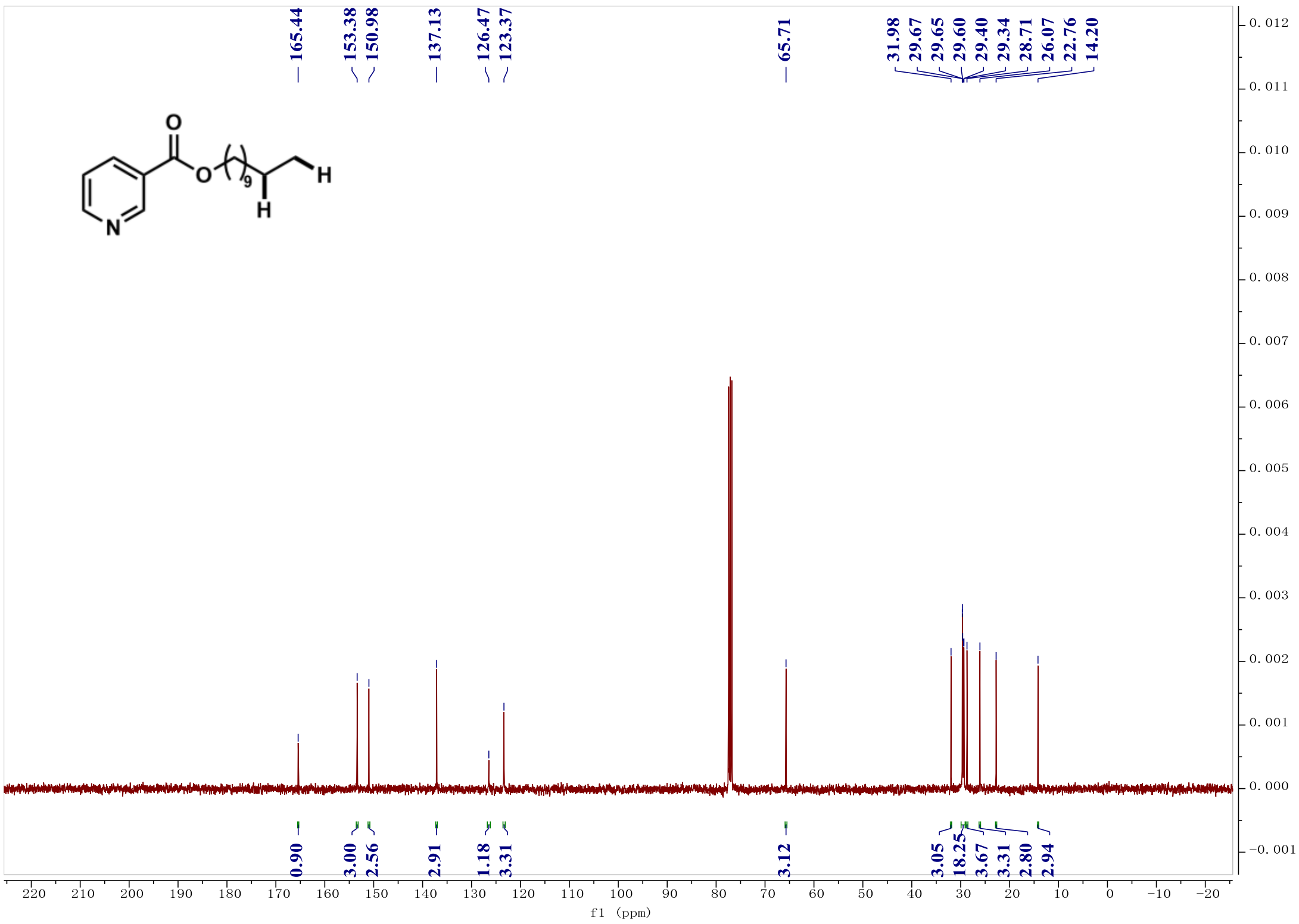


$^{13}$**C NMR** (101 MHz, $CDCl_3$) Spectrum of Compound **3ad**

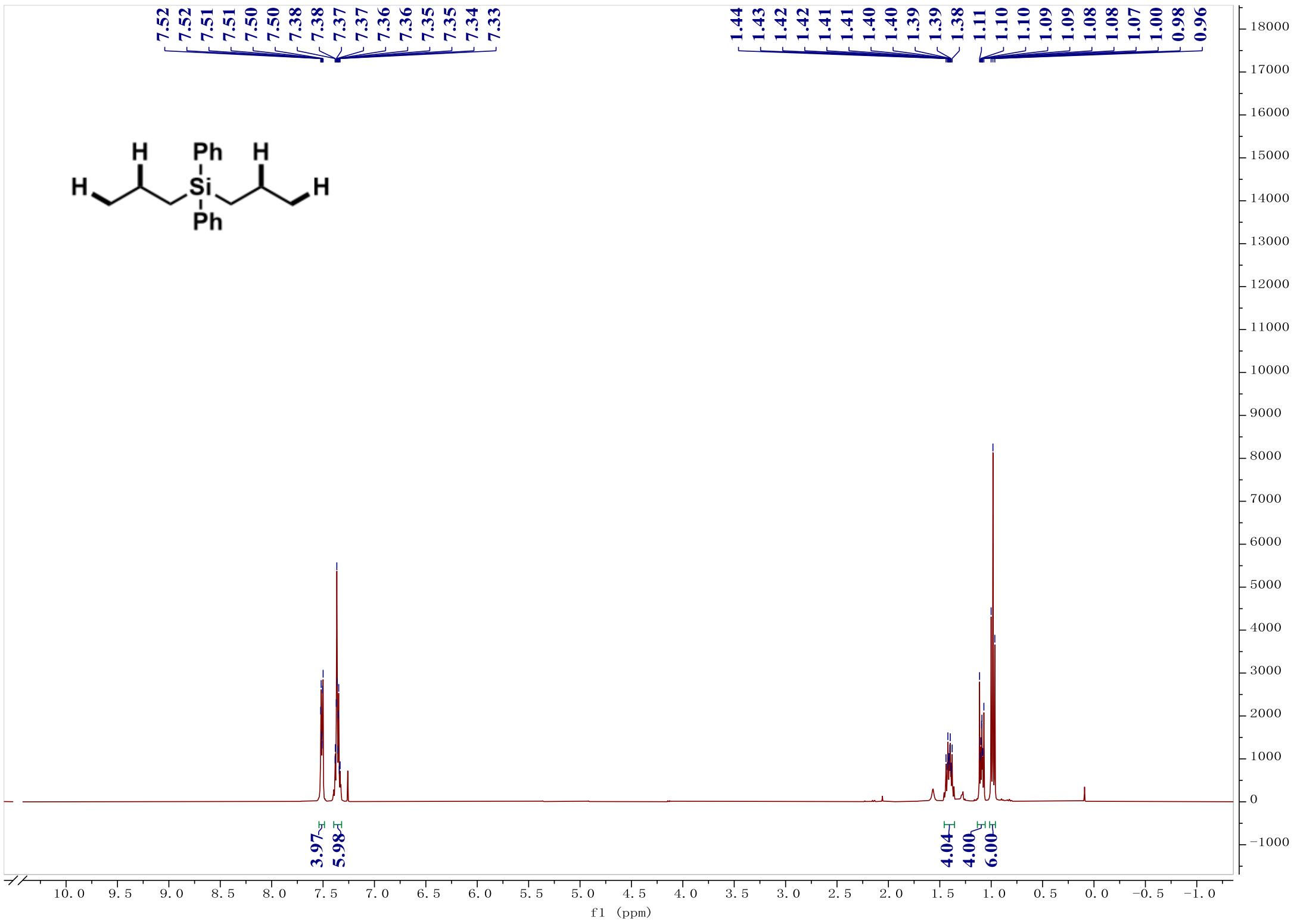



$^1$**H NMR** (400 MHz, $CDCl_3$) Spectrum of Compound **3ae**

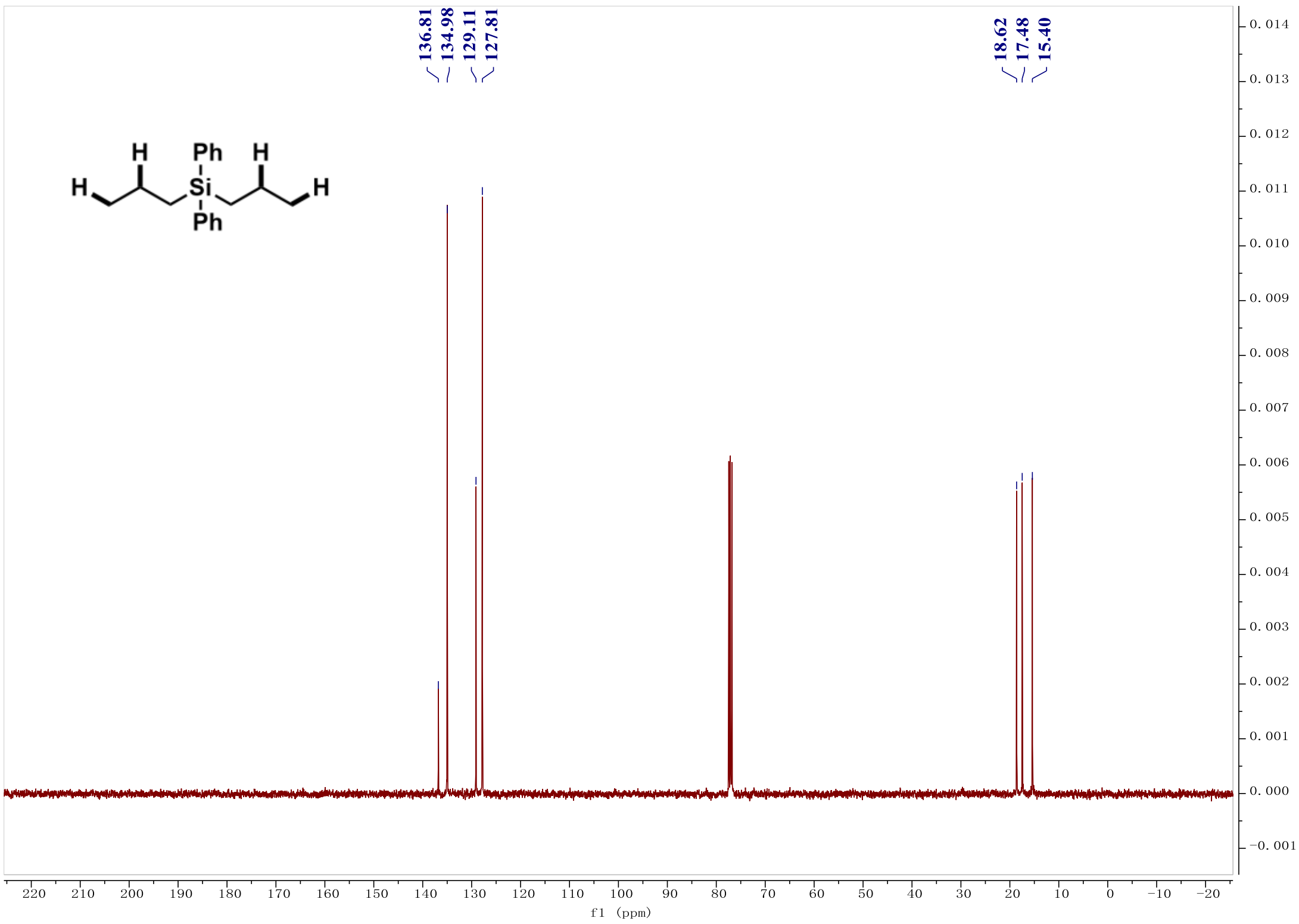



$^{13}$**C NMR** (101 MHz, $CDCl_3$) Spectrum of Compound **3ae**

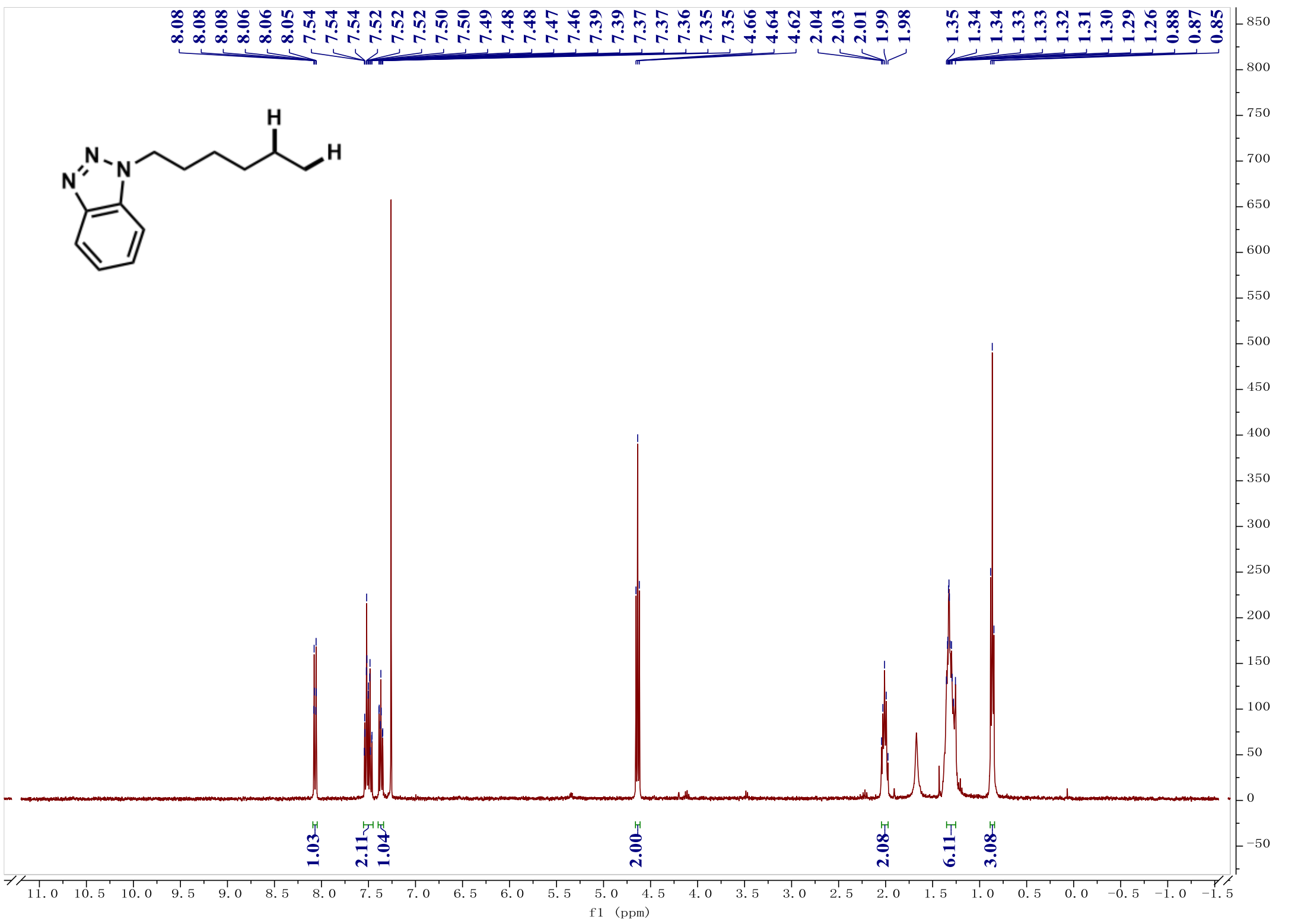

$^{1}$**H NMR** (400 MHz, $CDCl_3$) Spectrum of Compound **3af**

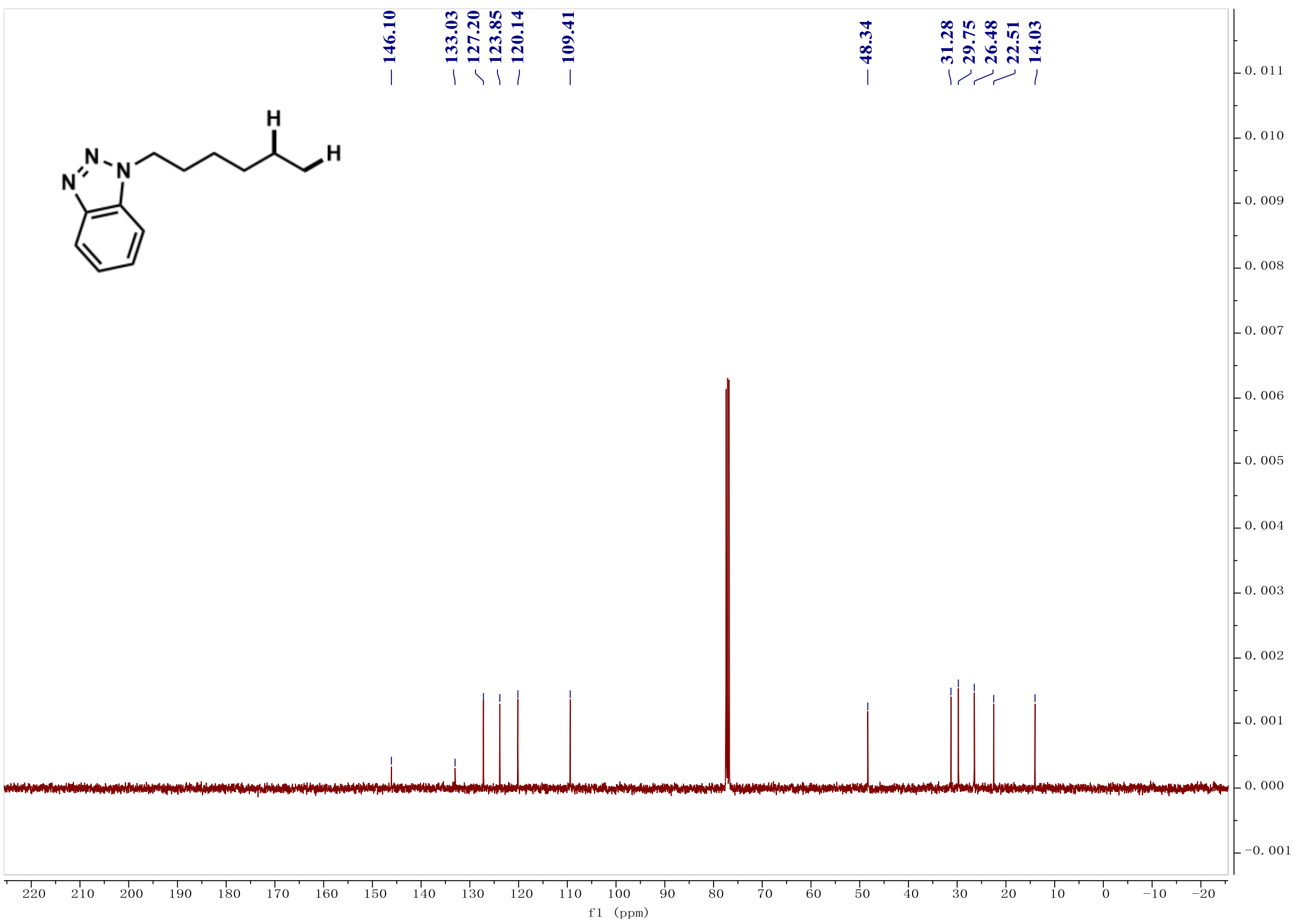

$^{13}$**C NMR** (101 MHz, $CDCl_3$) Spectrum of Compound **3af**

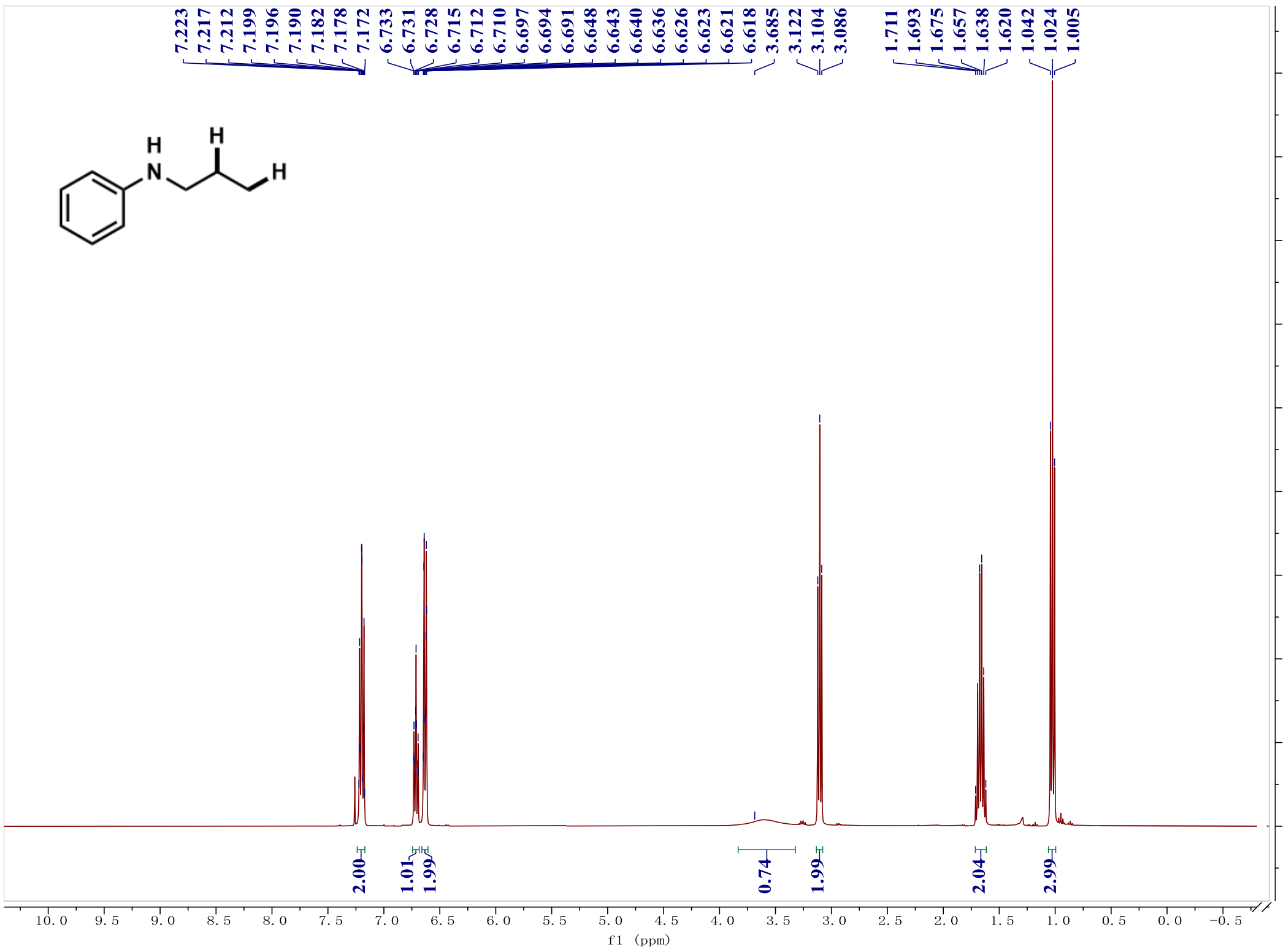

**$^{1}$H NMR** (400 MHz, $CDCl_3$) Spectrum of Compound **3ag**

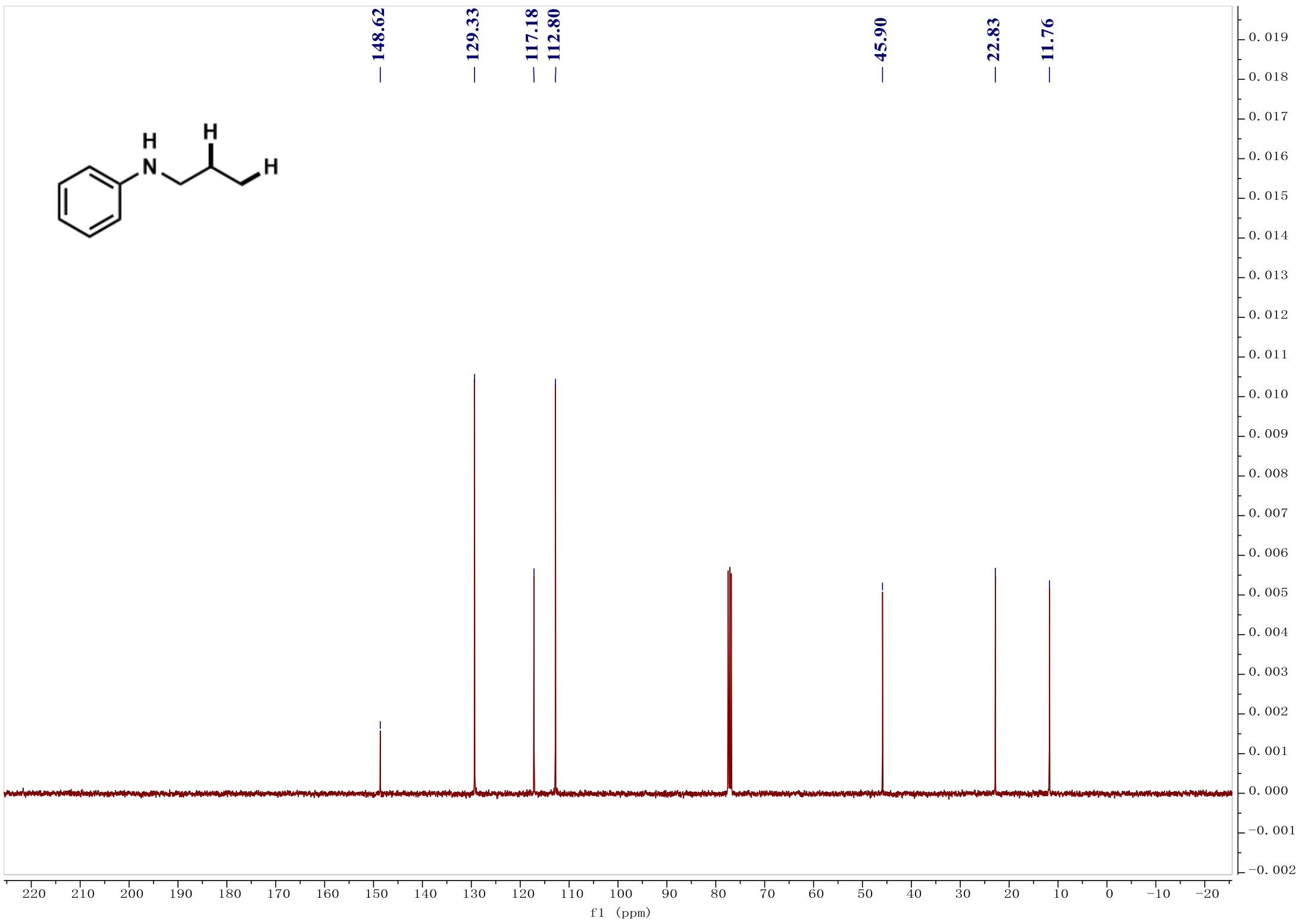

**$^{13}$C NMR** (101 MHz, $CDCl_3$) Spectrum of Compound **3ag**

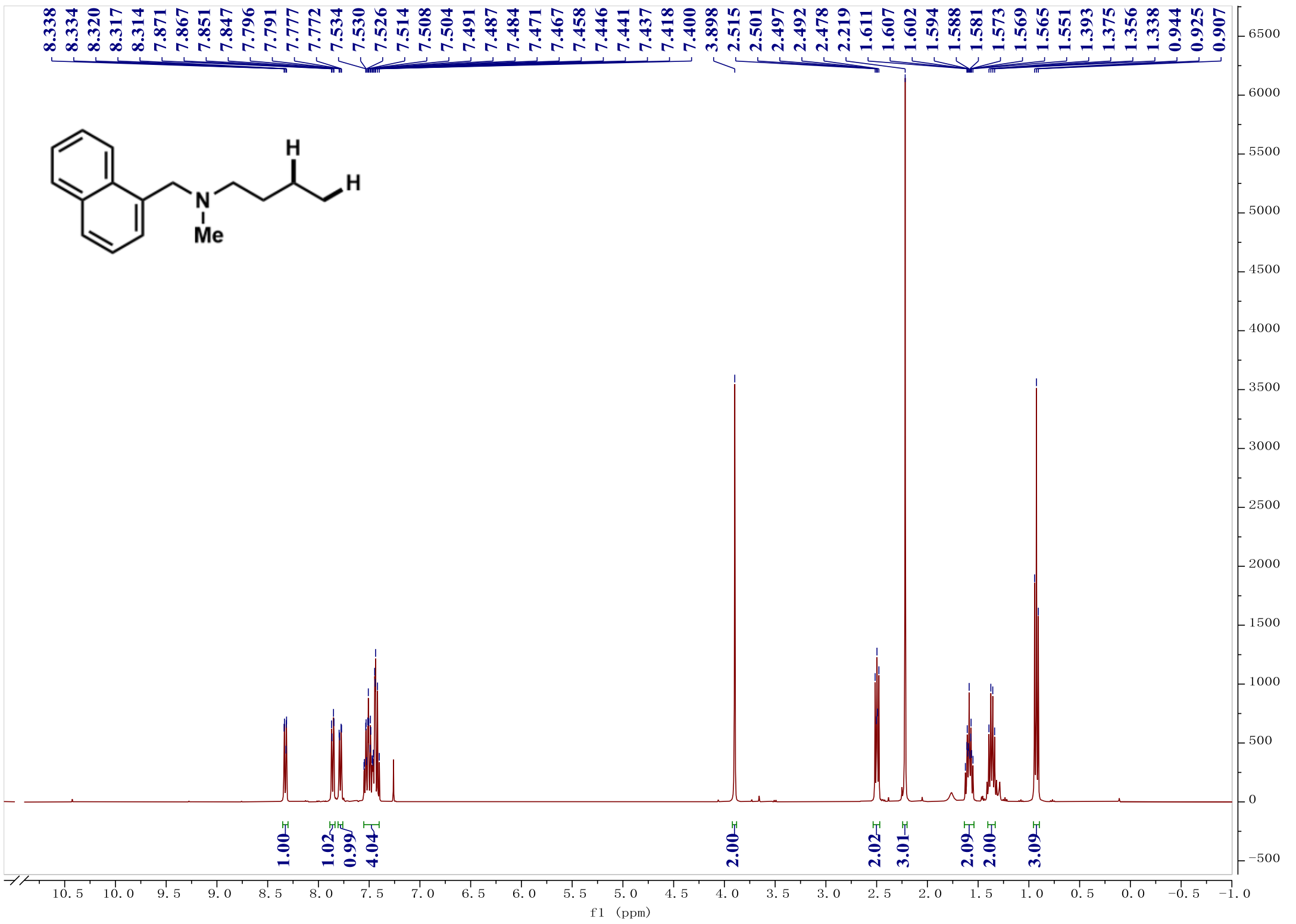


$^{1}$**H NMR** (400 MHz, $CDCl_3$) Spectrum of Compound **3ah**

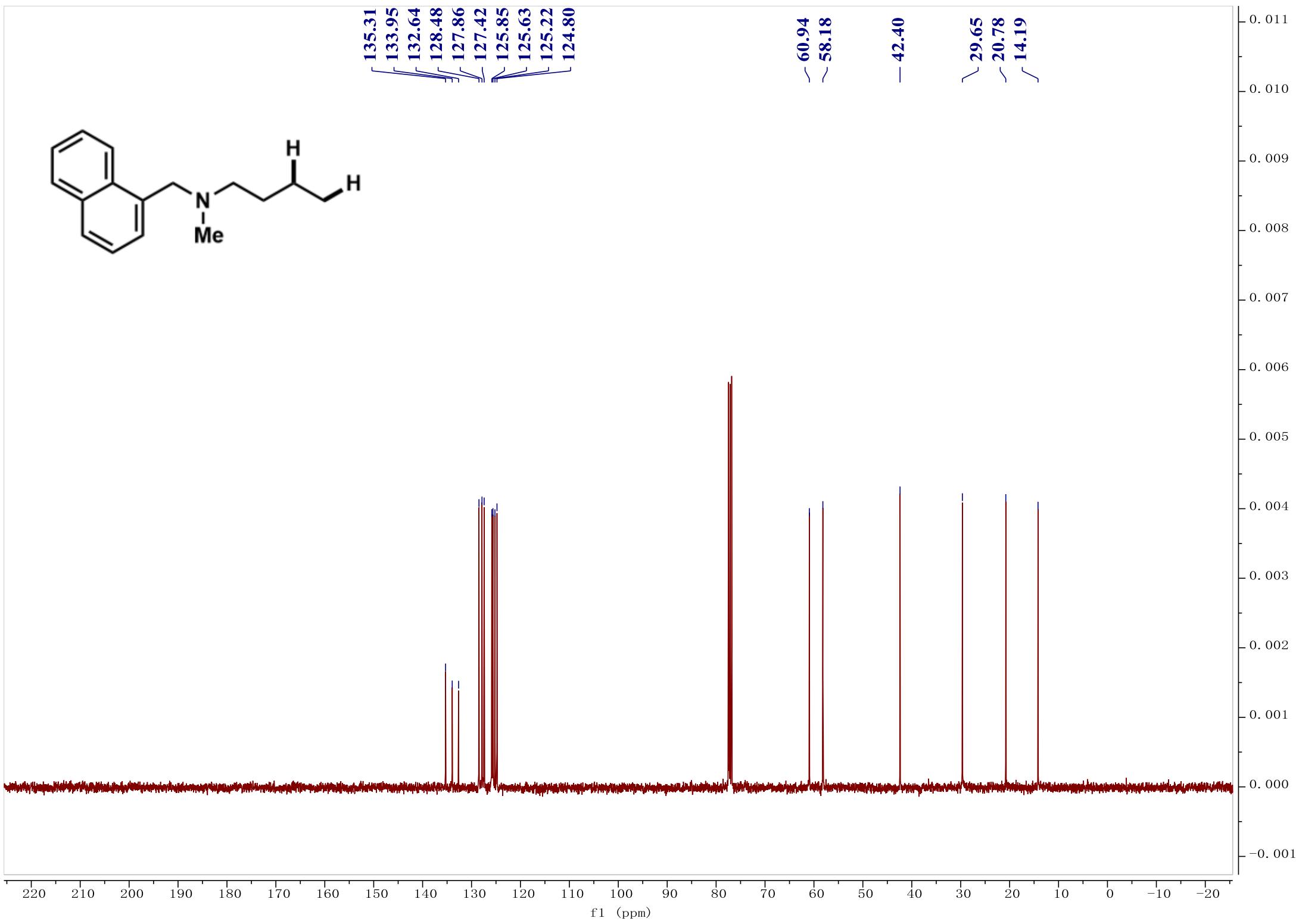


$^{13}$**C NMR** (101 MHz, $CDCl_3$) Spectrum of Compound **3ah**

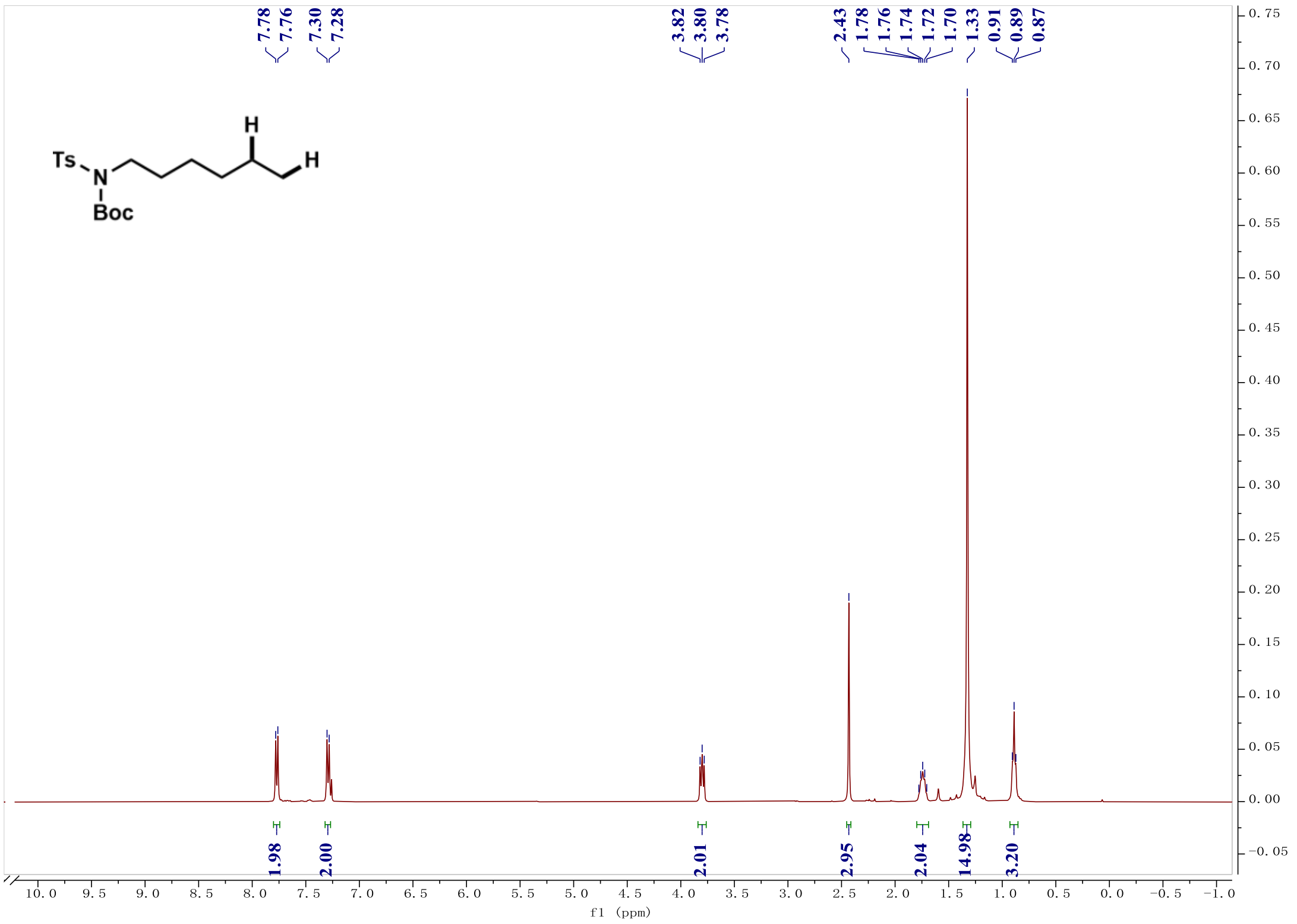

**¹H NMR** (400 MHz, $CDCl_3$) Spectrum of Compound **3ai**

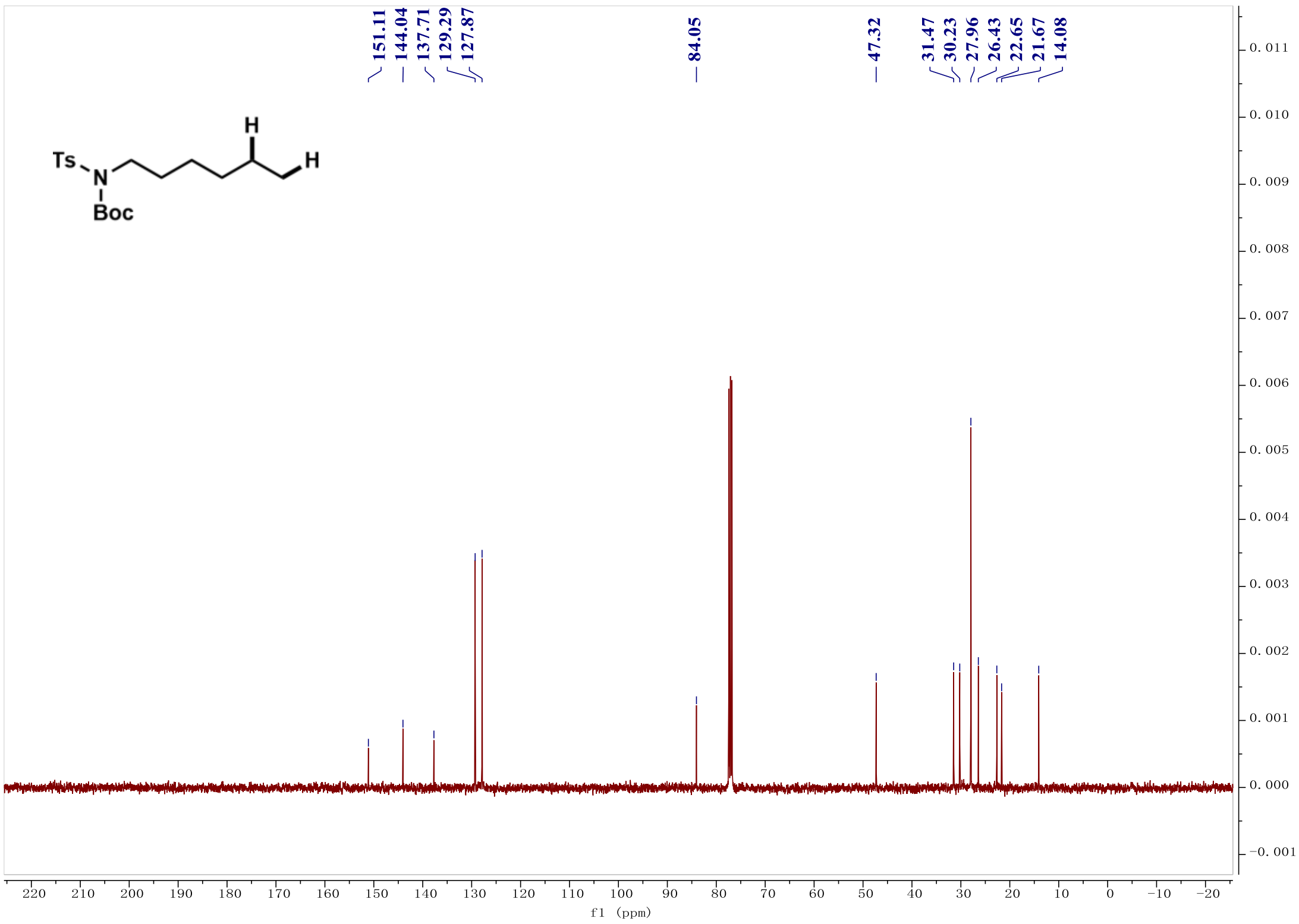

**¹³C NMR** (101 MHz, $CDCl_3$) Spectrum of Compound **3ai**

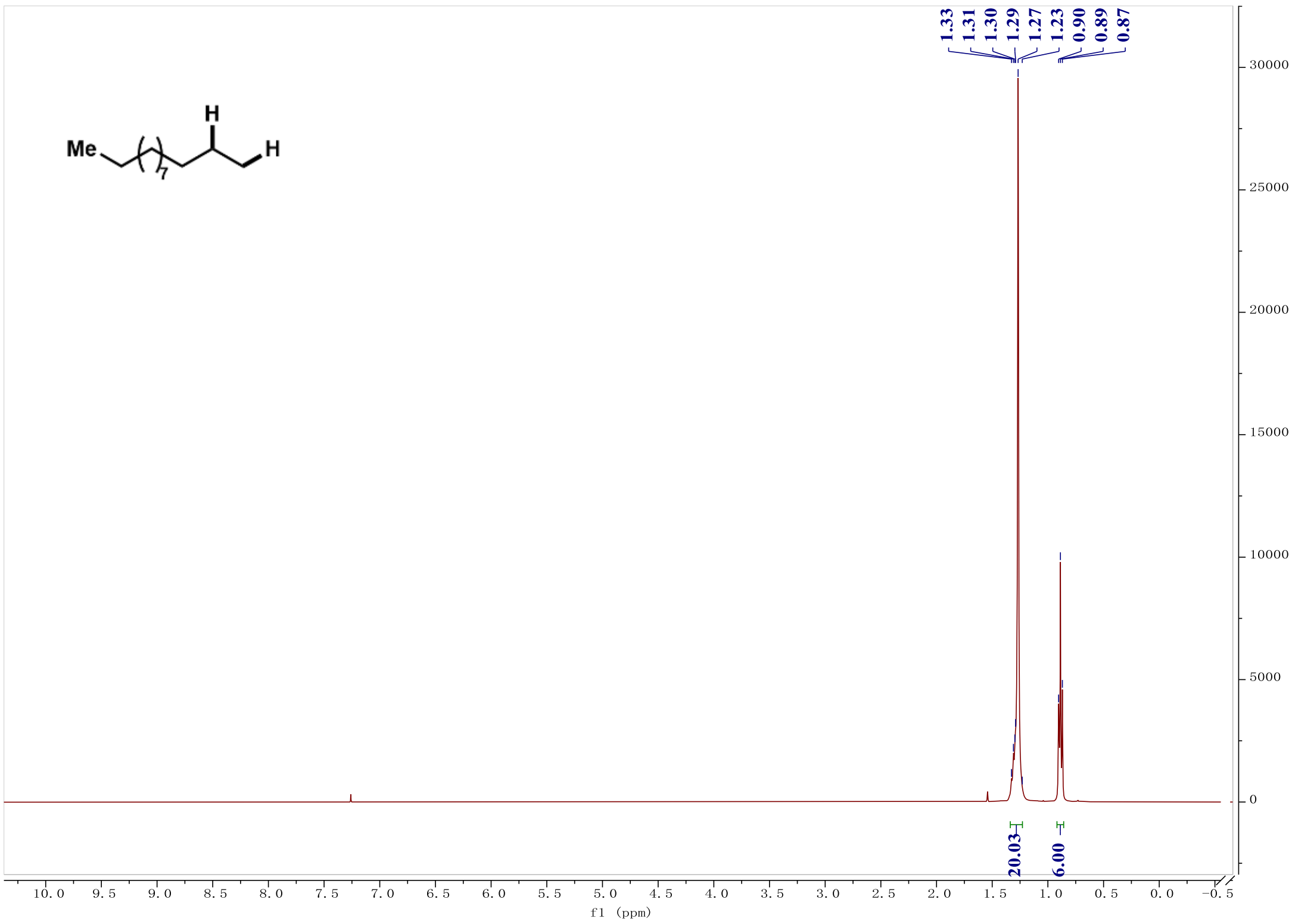


$^{1}$**H NMR** (400 MHz, $CDCl_3$) Spectrum of Compound **3aj**

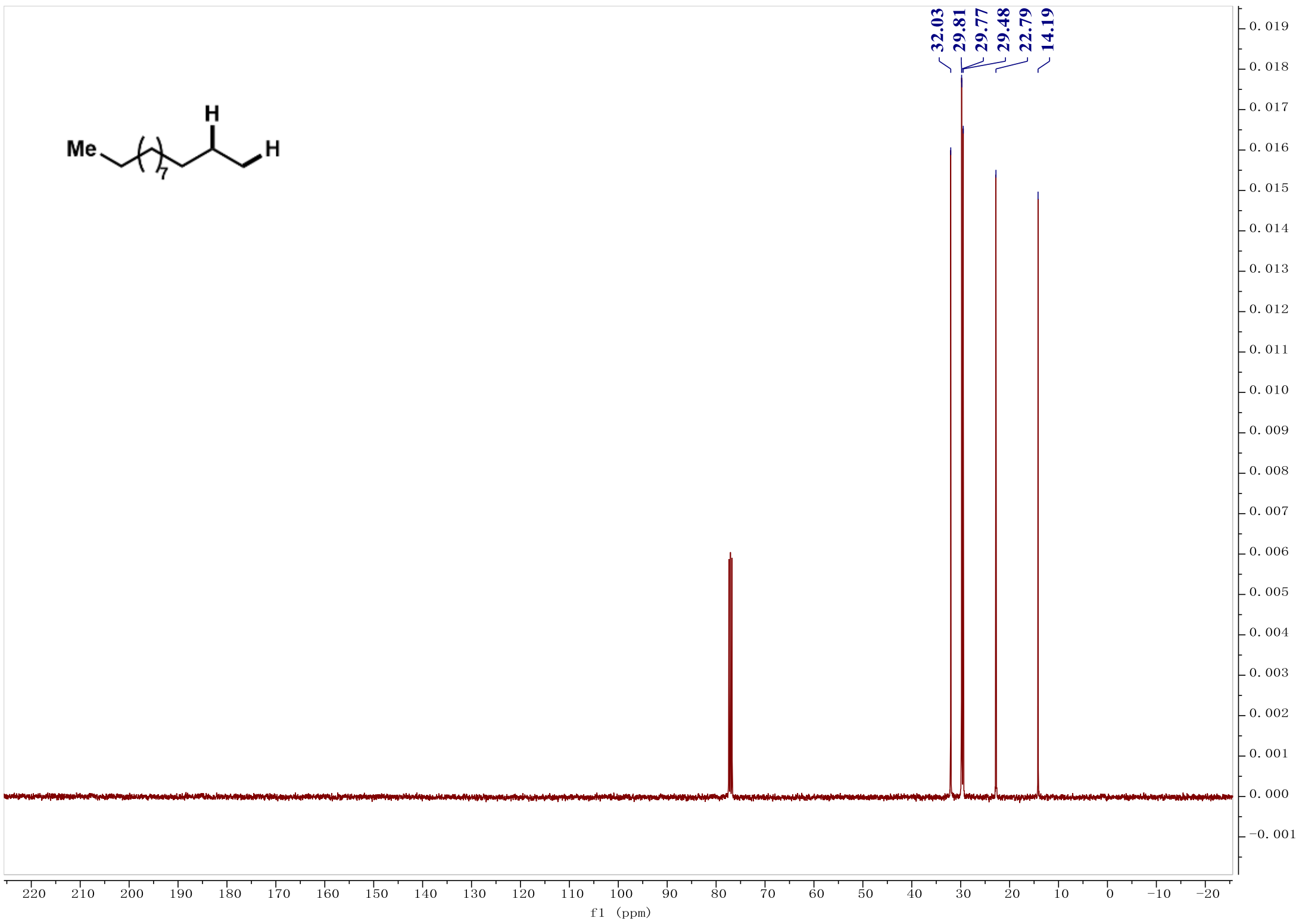


$^{13}$**C NMR** (101 MHz, $CDCl_3$) Spectrum of Compound **3aj**

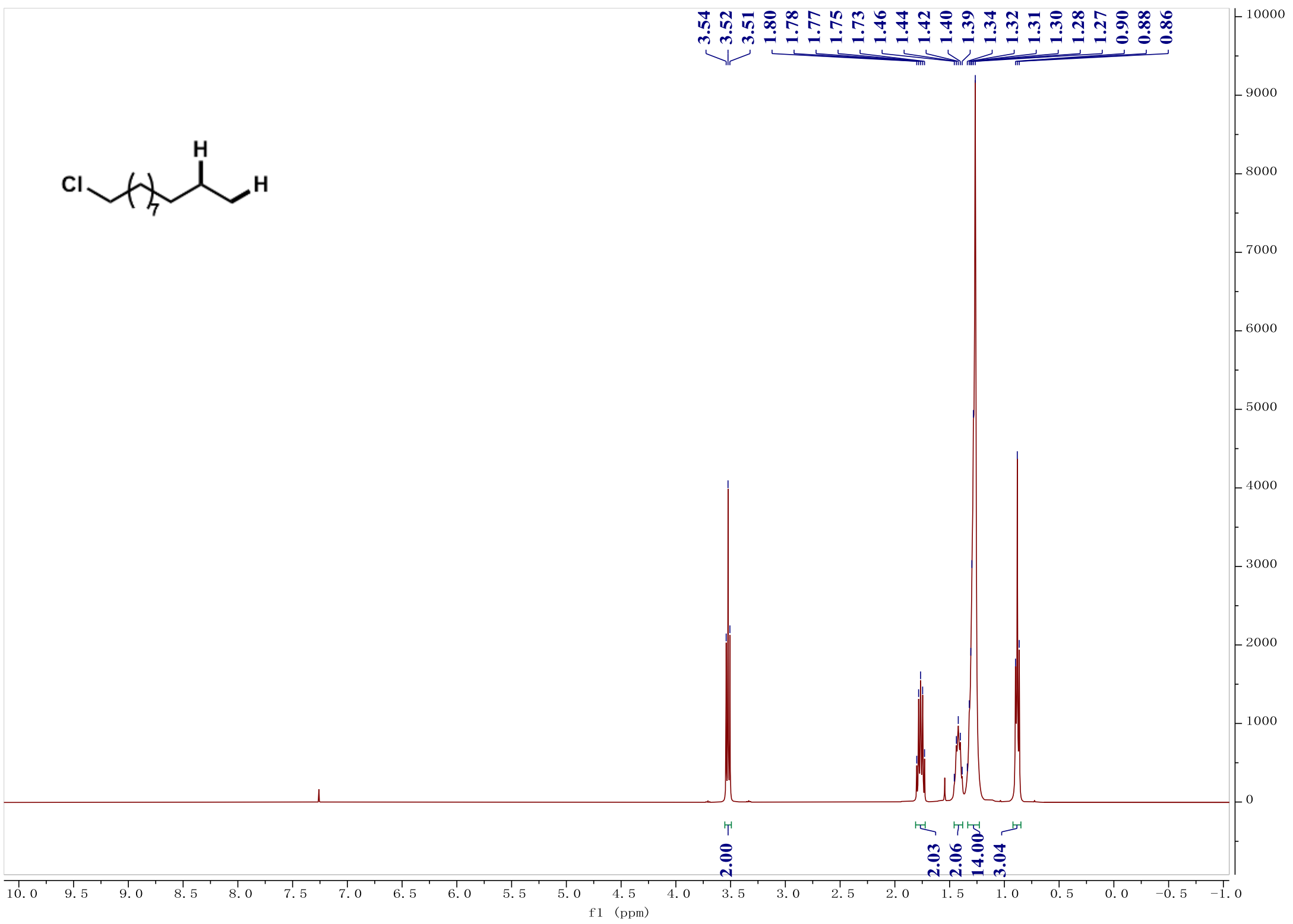

$^{1}$**H NMR** (400 MHz, $CDCl_3$) Spectrum of Compound **3ak**

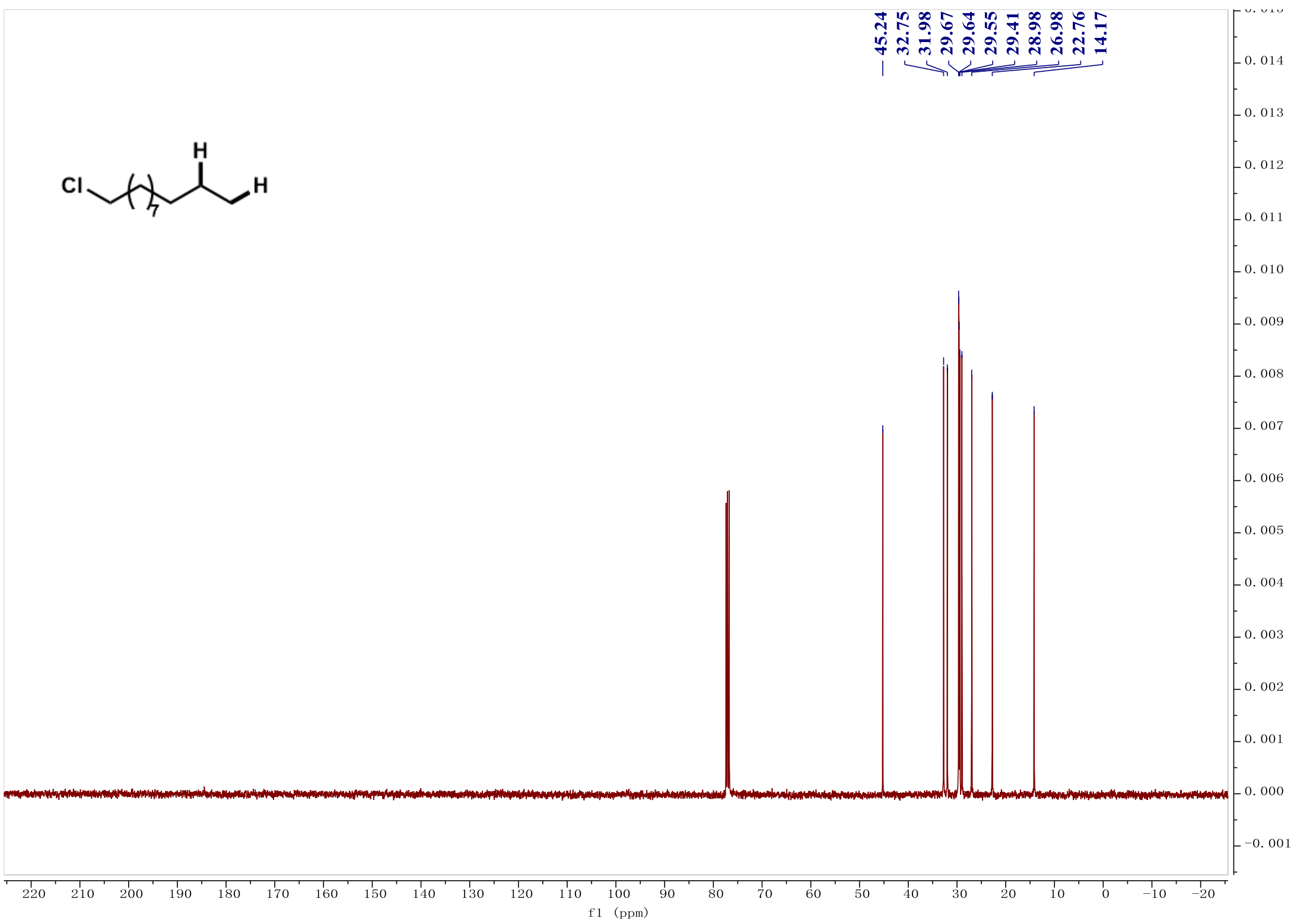

$^{13}$**C NMR** (101 MHz, $CDCl_3$) Spectrum of Compound **3ak**

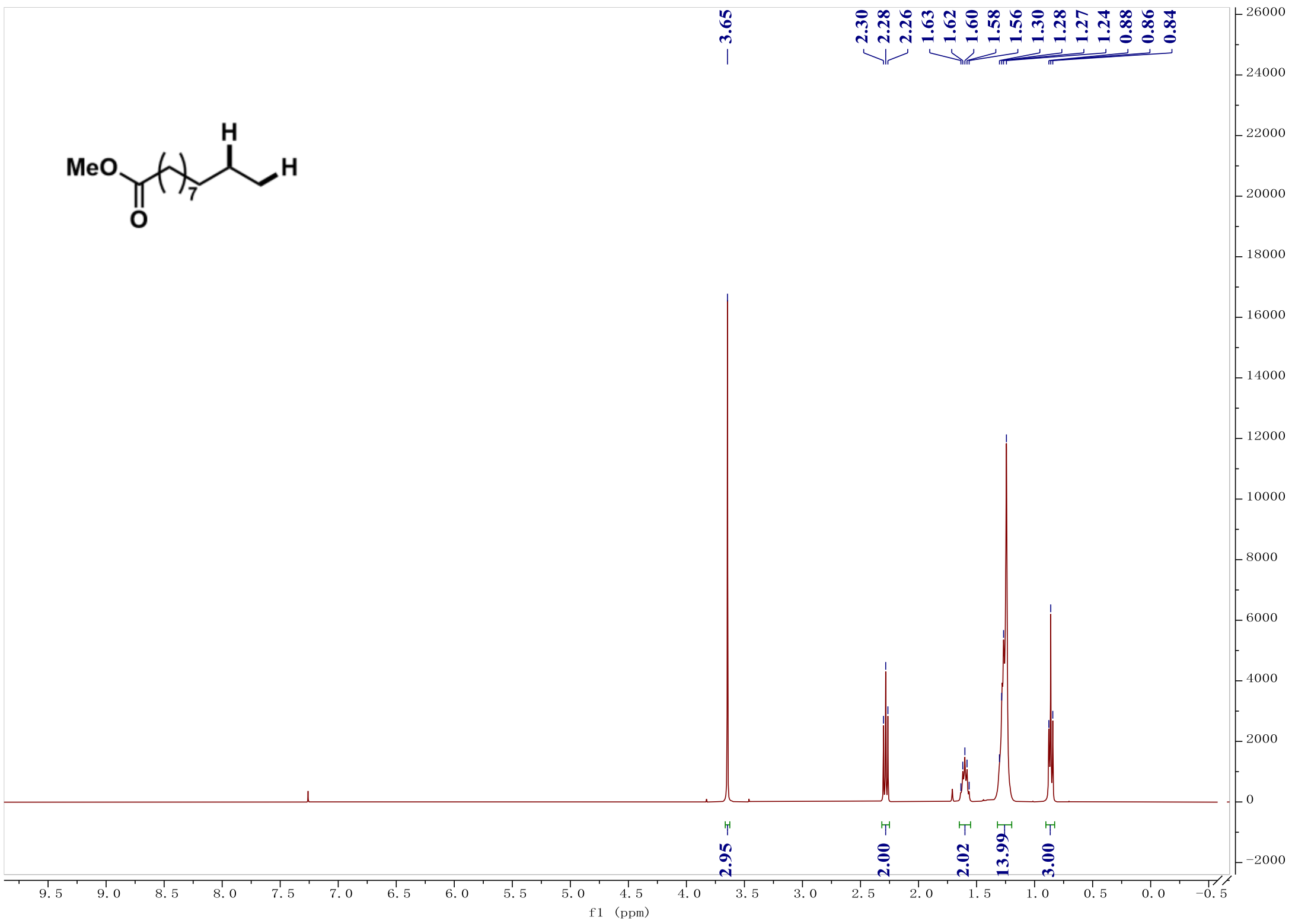

**$^1$H NMR** (400 MHz, $CDCl_3$) Spectrum of Compound **3al**

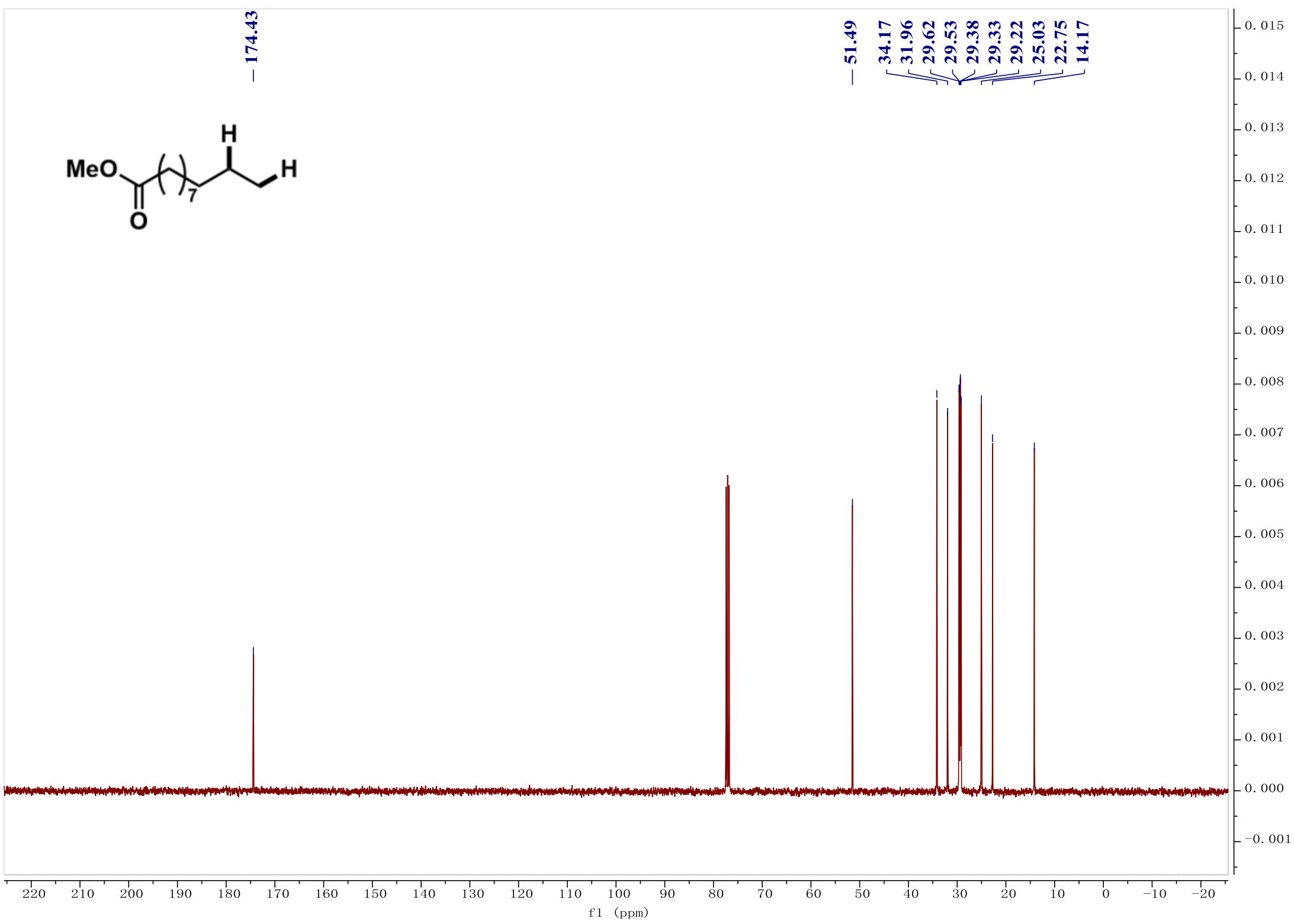

**$^{13}$C NMR** (101 MHz, $CDCl_3$) Spectrum of Compound **3al**

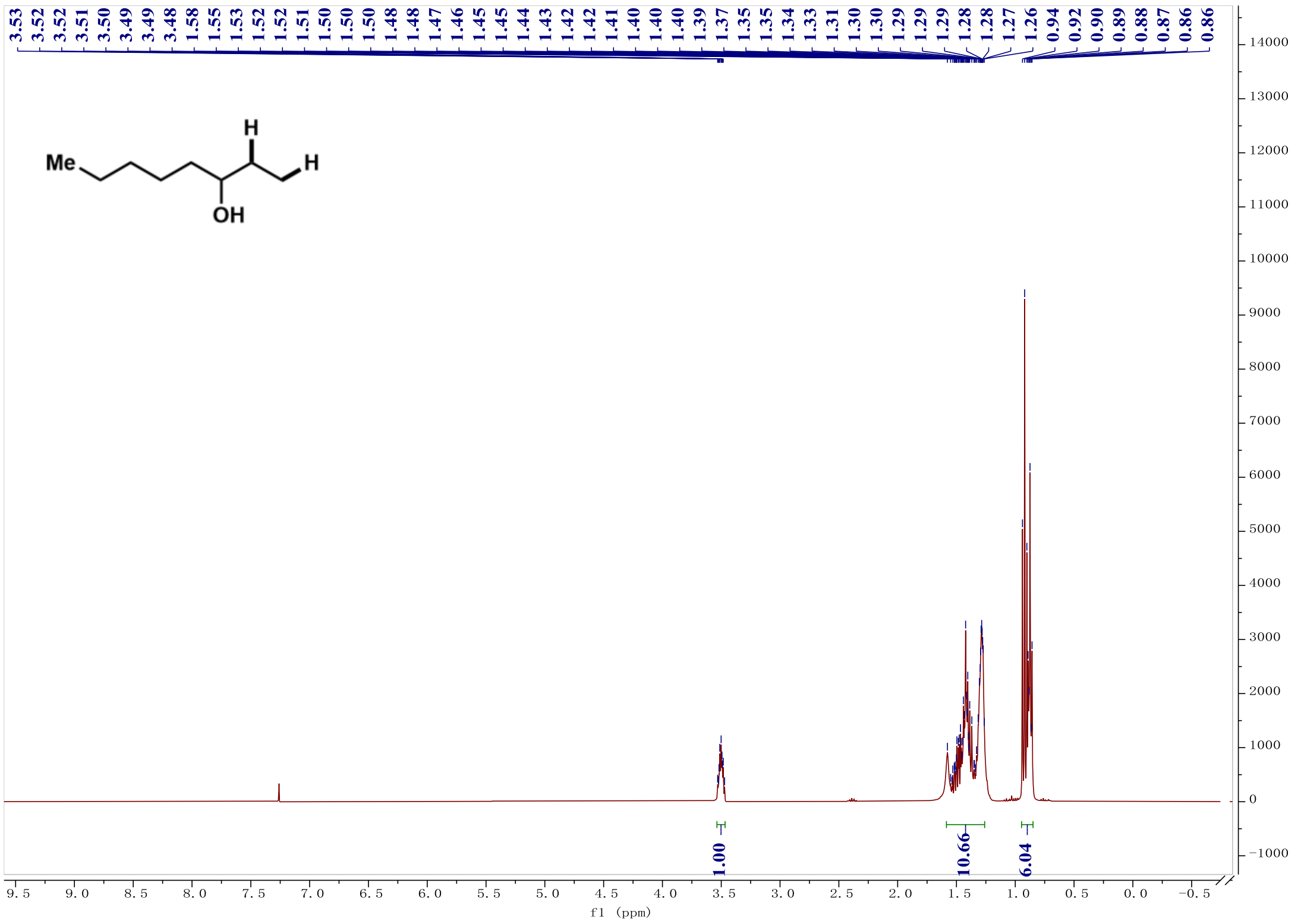


$^{1}$H NMR (400 MHz, $CDCl_3$) Spectrum of Compound **3am**

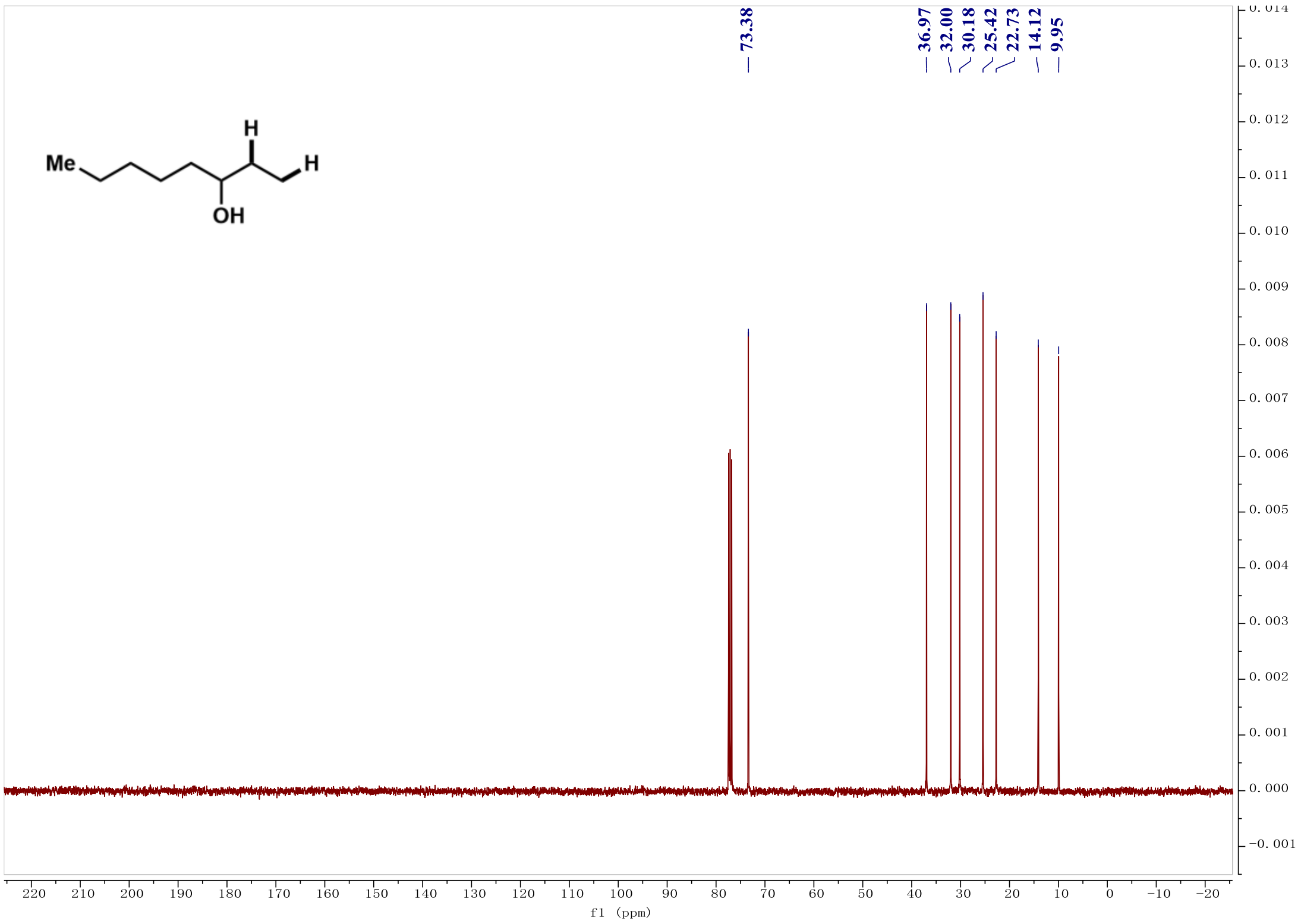


$^{13}$C NMR (101 MHz, $CDCl_3$) Spectrum of Compound **3am**

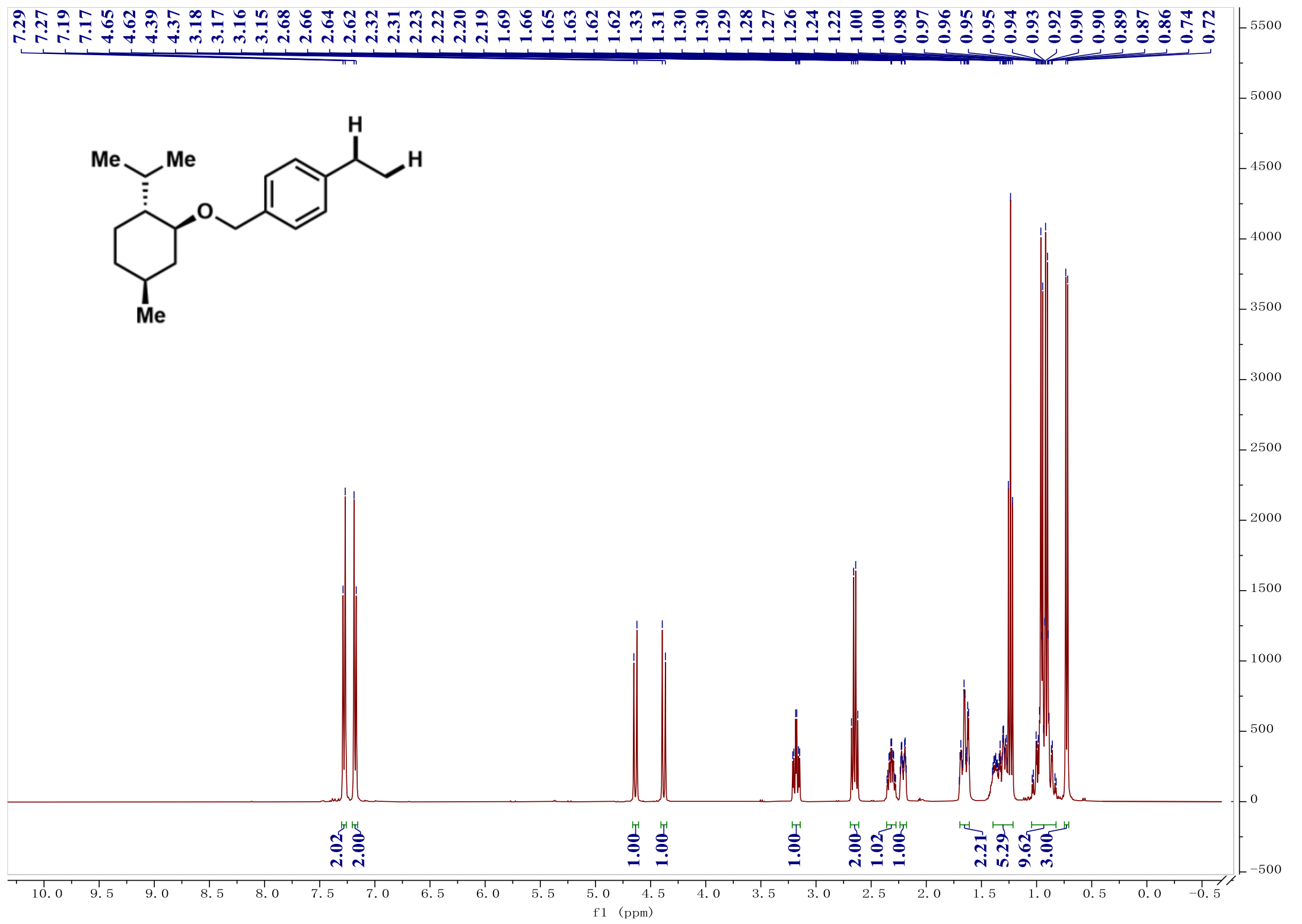


**$^{1}$H NMR** (400 MHz, $CDCl_3$) Spectrum of Compound **3ar**

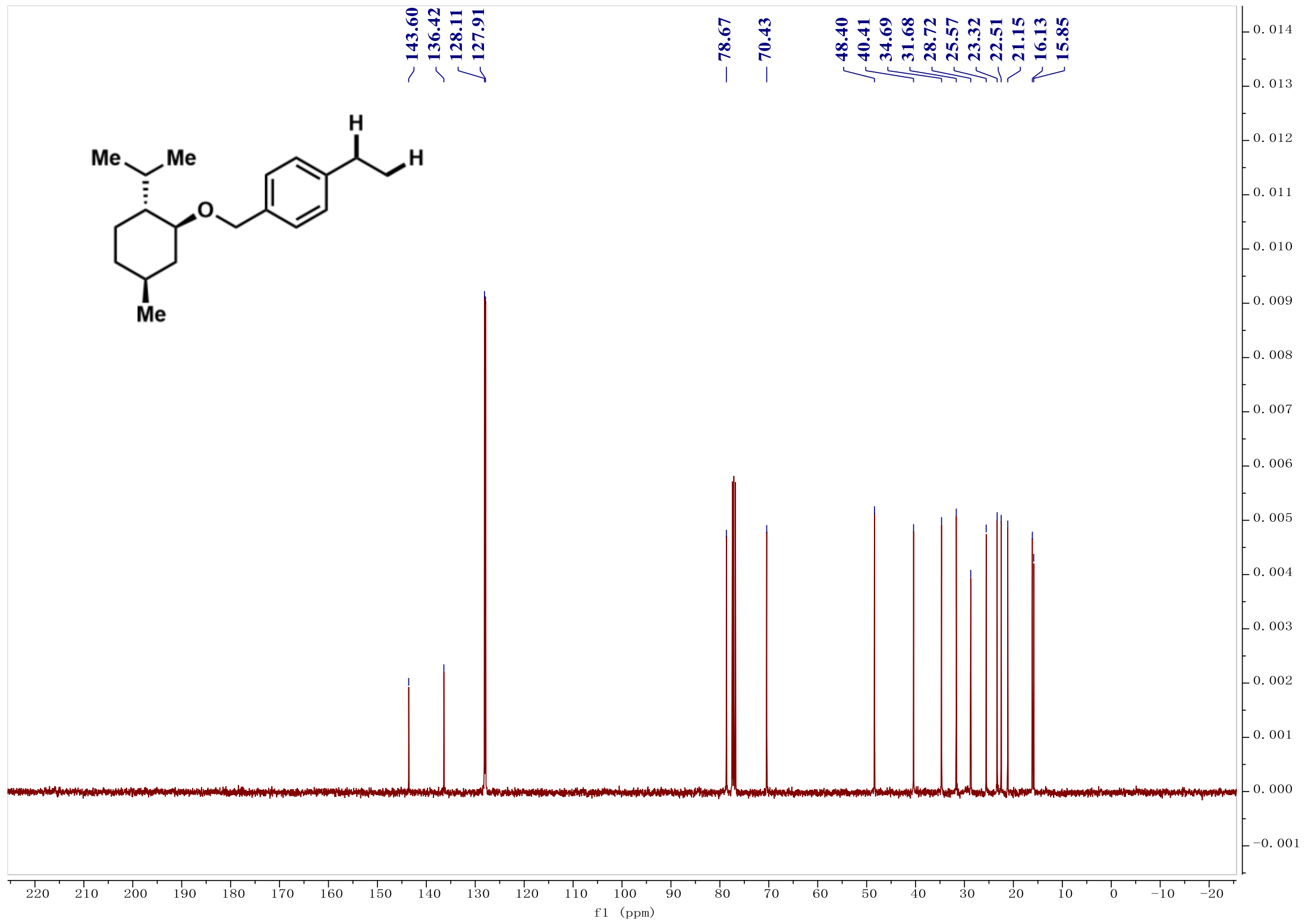


**$^{13}$C NMR** (101 MHz, $CDCl_3$) Spectrum of Compound **3ar**

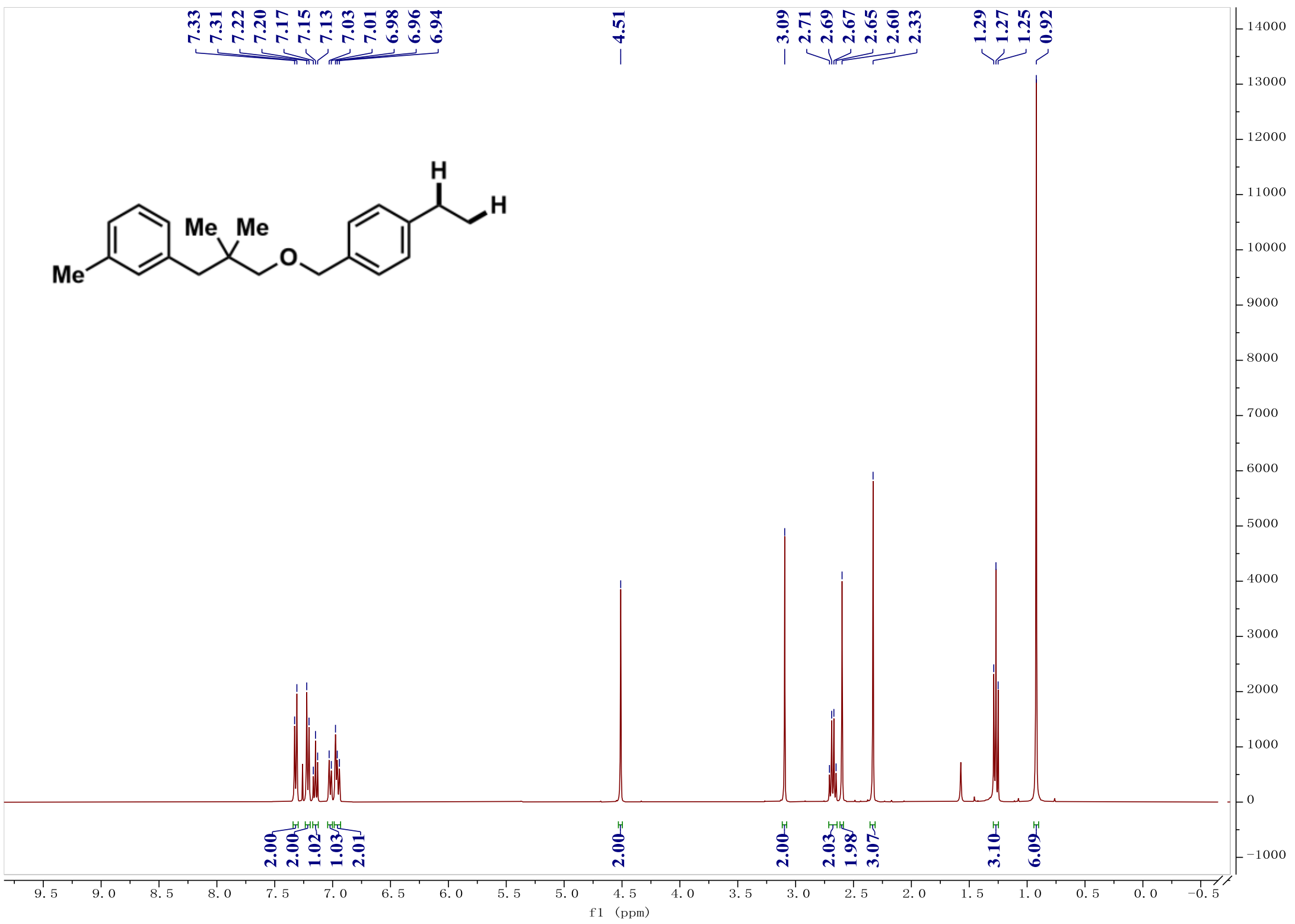

$^{1}$**H NMR** (400 MHz, $CDCl_3$) Spectrum of Compound **3as**

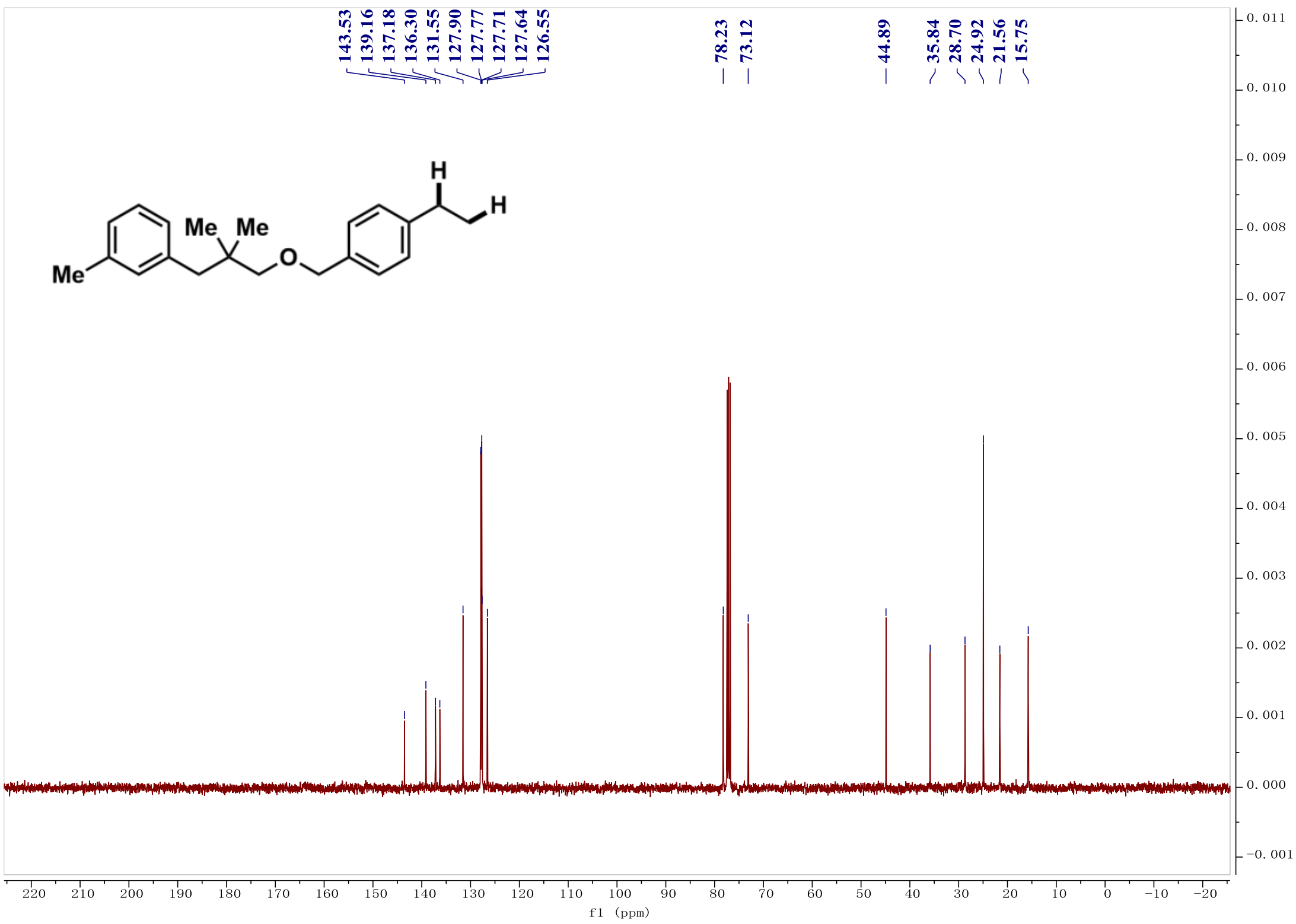

$^{13}$**C NMR** (101 MHz, $CDCl_3$) Spectrum of Compound **3as**

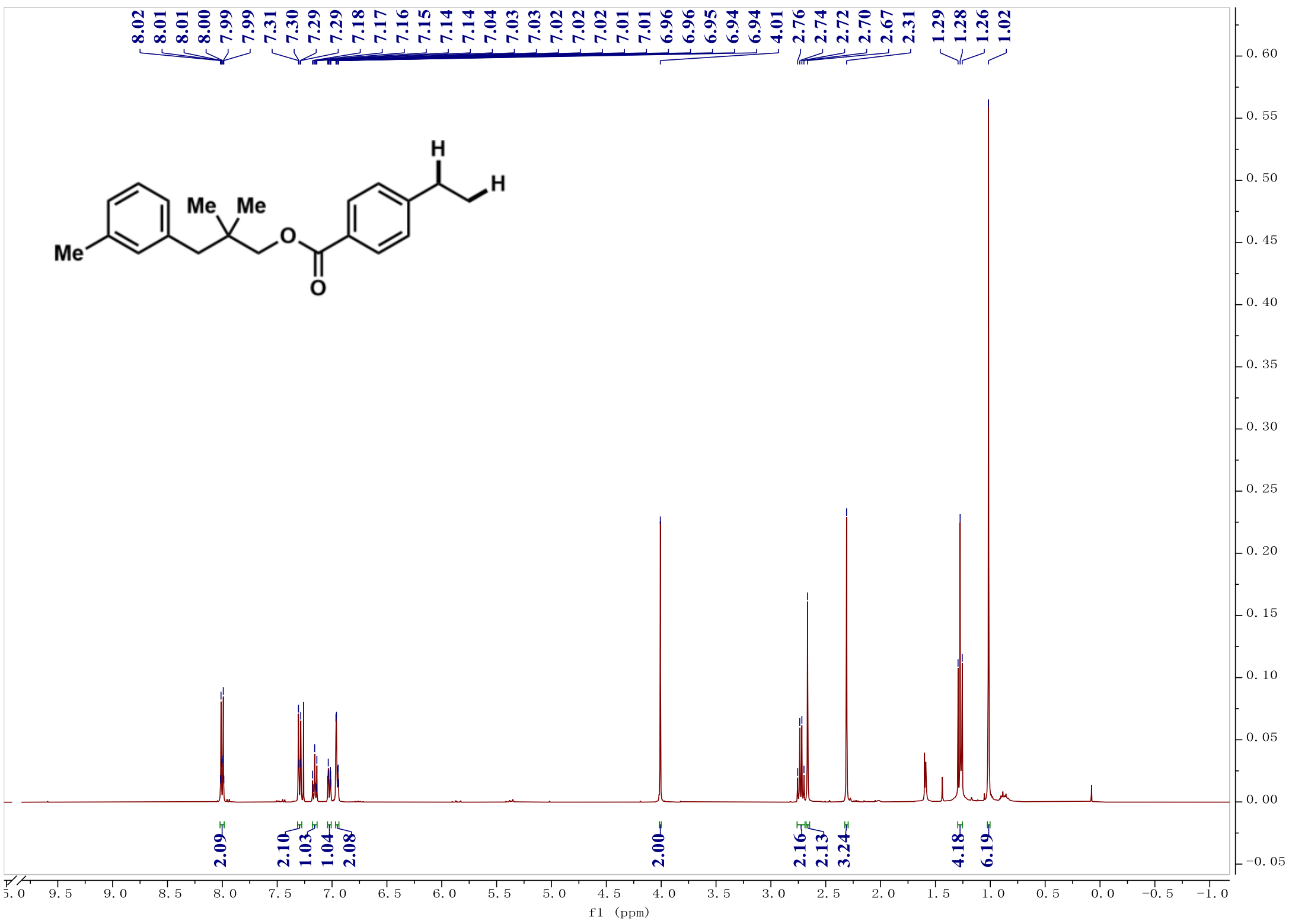


$^{1}$**H NMR** (400 MHz, $CDCl_3$) Spectrum of Compound **3at**

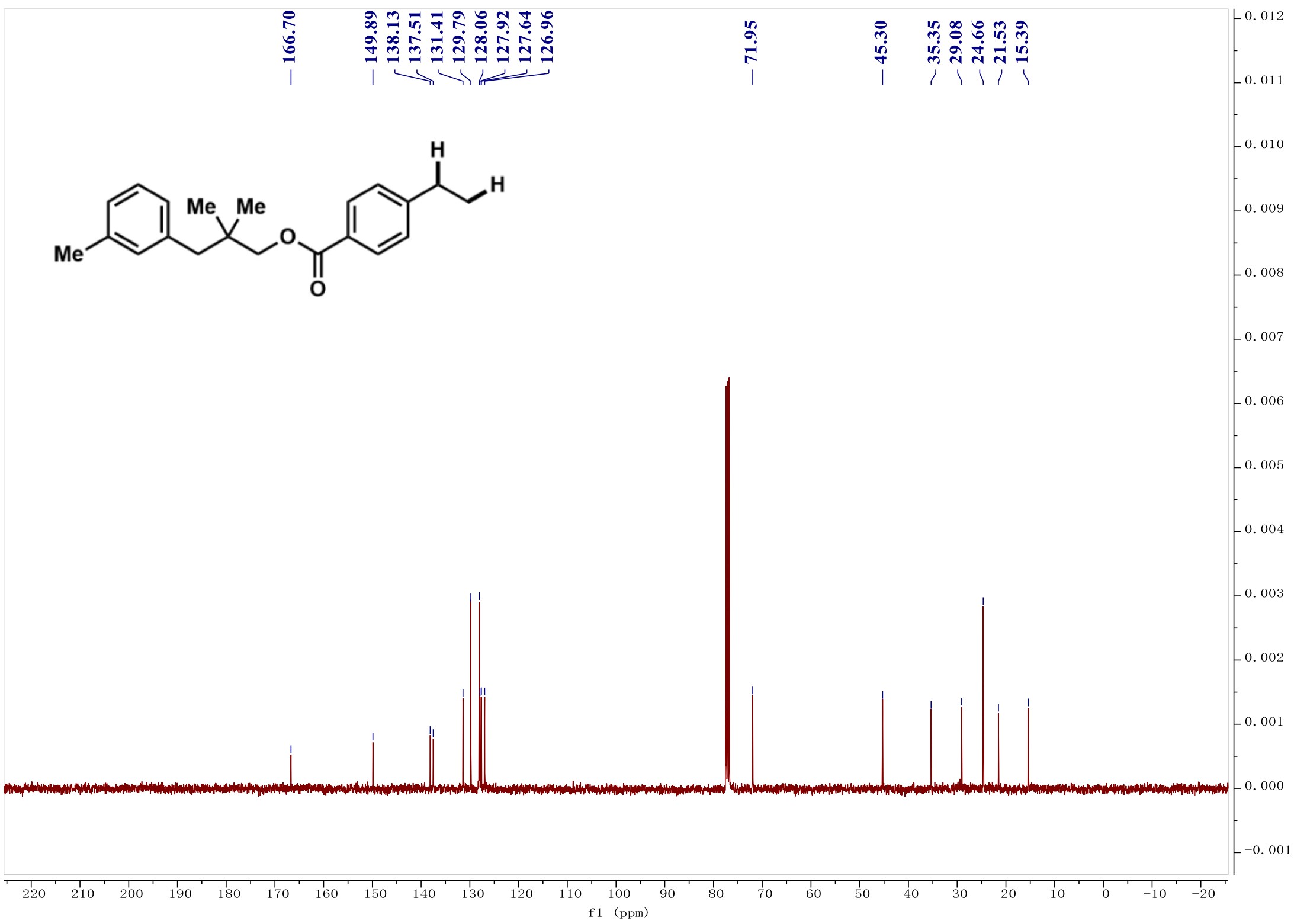


$^{13}$**C NMR** (101 MHz, $CDCl_3$) Spectrum of Compound **3at**

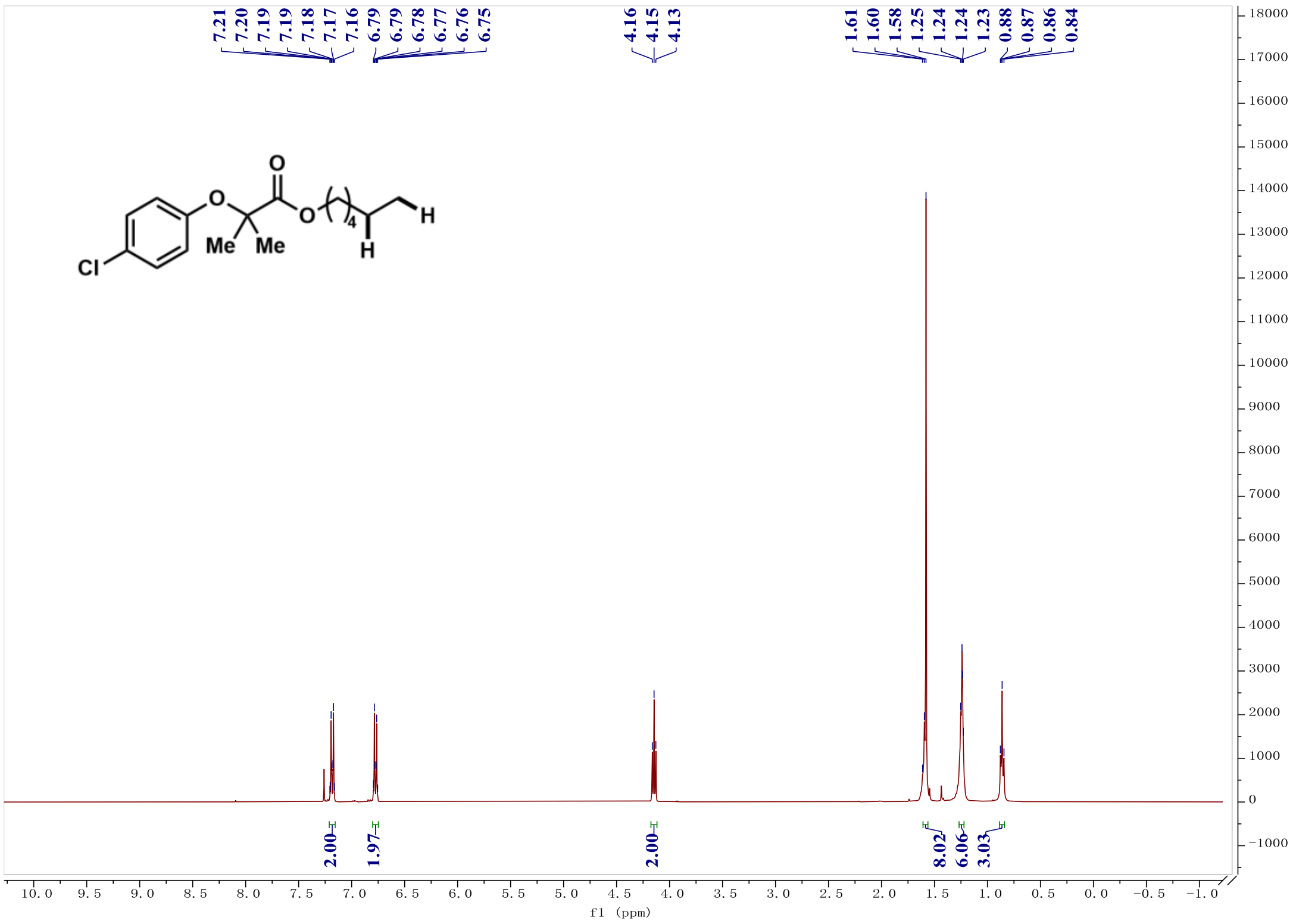

$^{1}$H NMR (400 MHz, $CDCl_3$) Spectrum of Compound **3au**

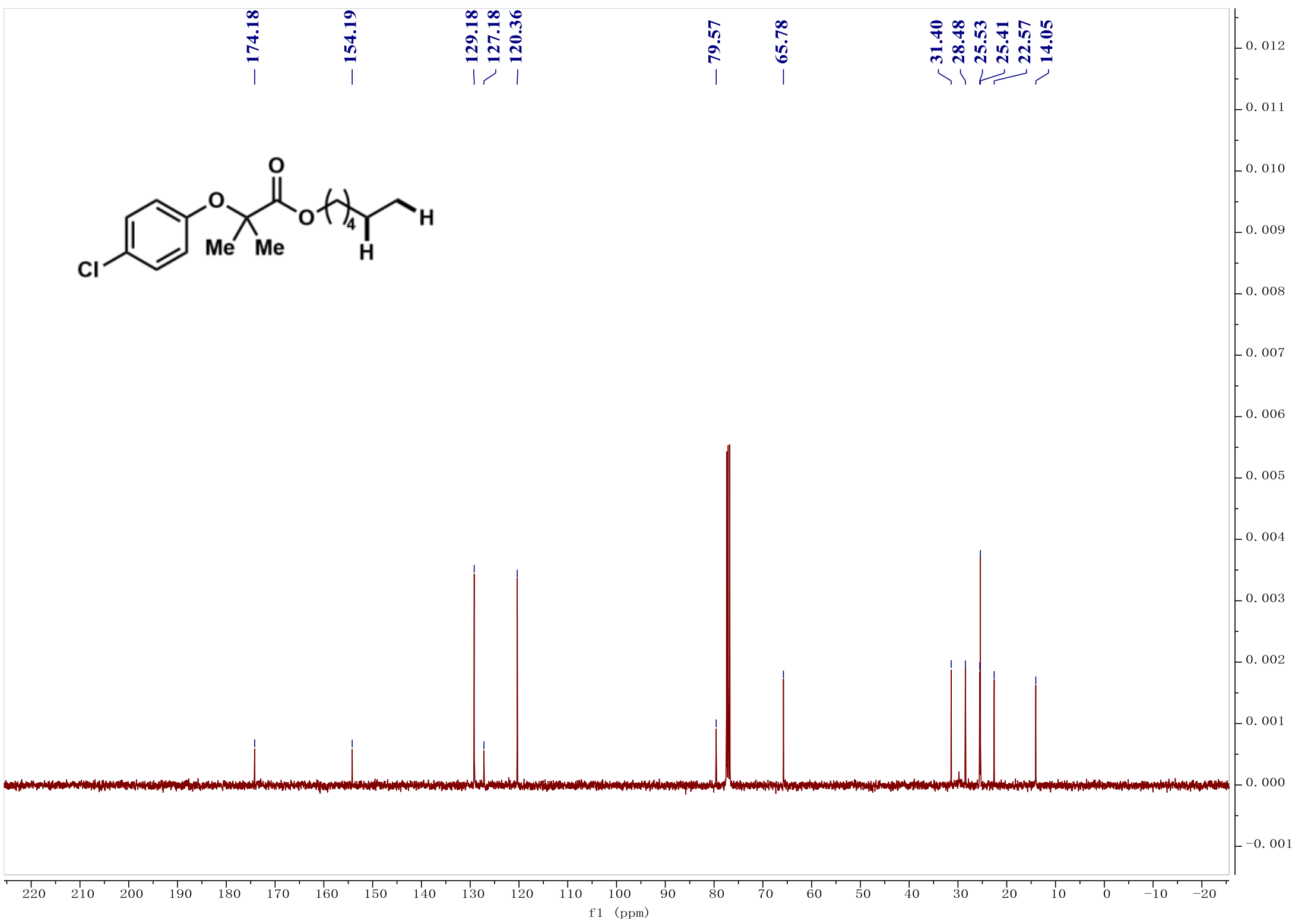

$^{13}$C NMR (101 MHz, $CDCl_3$) Spectrum of Compound **3au**

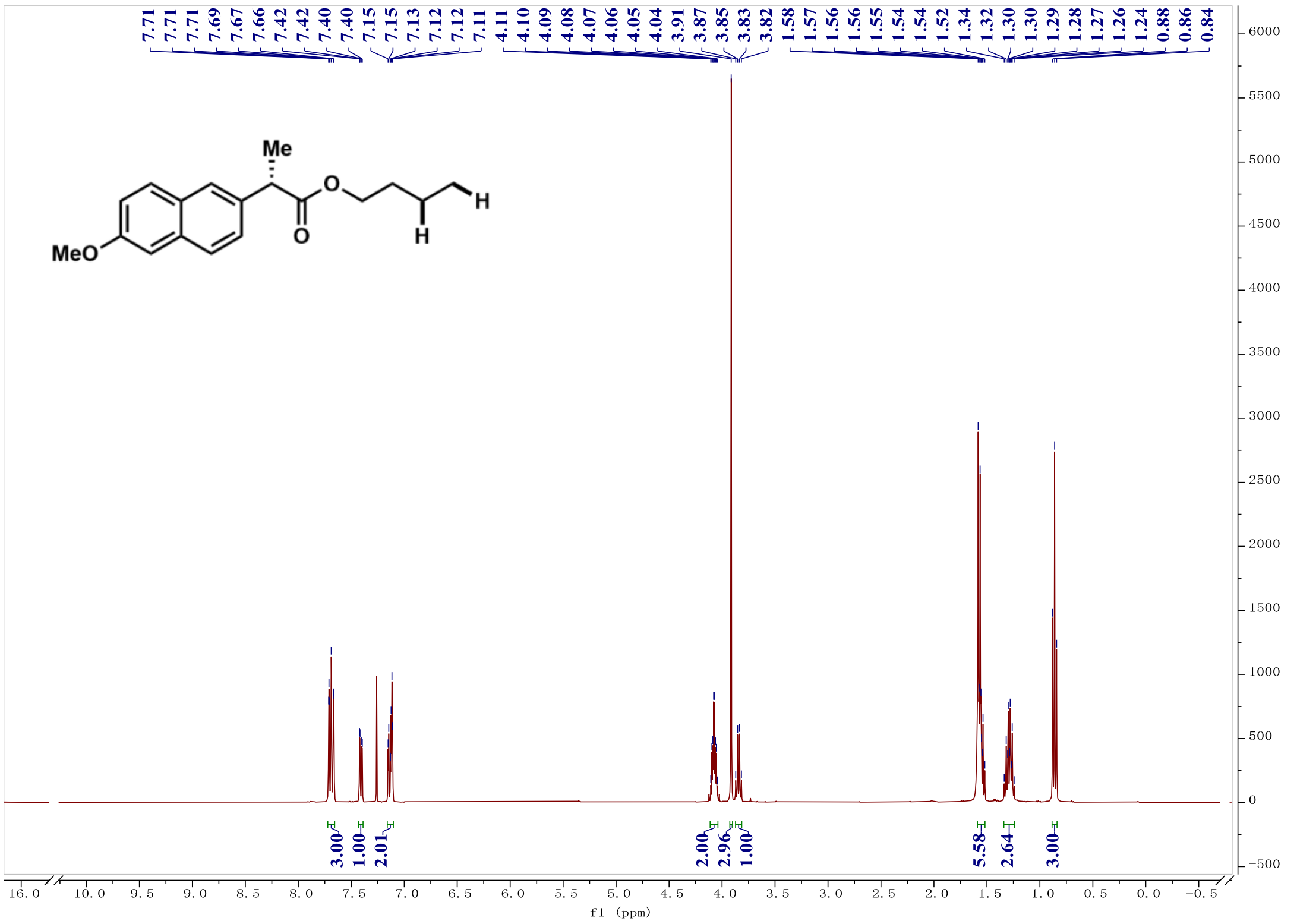

$^{1}$**H NMR** (400 MHz, $CDCl_3$) Spectrum of Compound **3av**

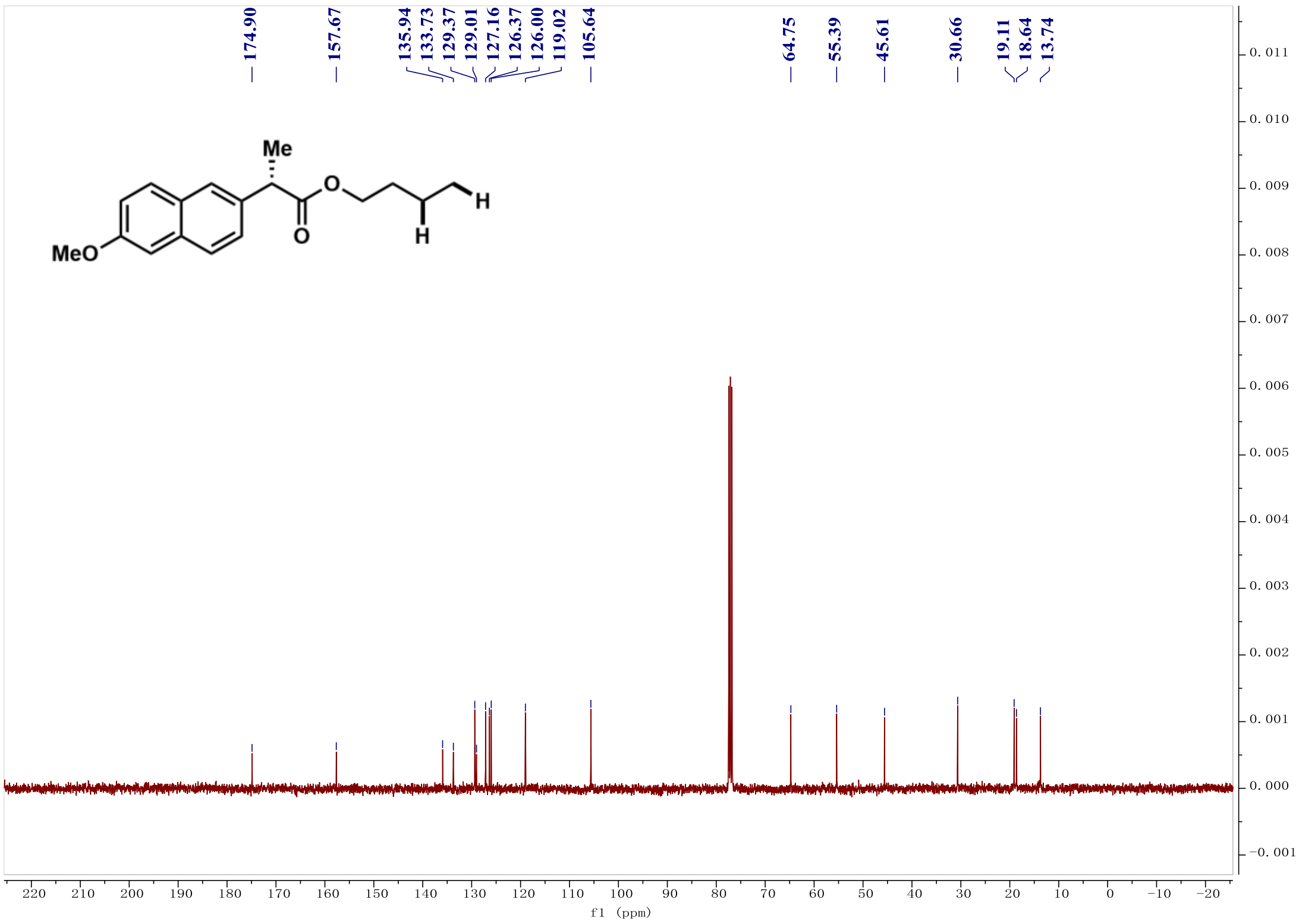

$^{13}$**C NMR** (101 MHz, $CDCl_3$) Spectrum of Compound **3av**

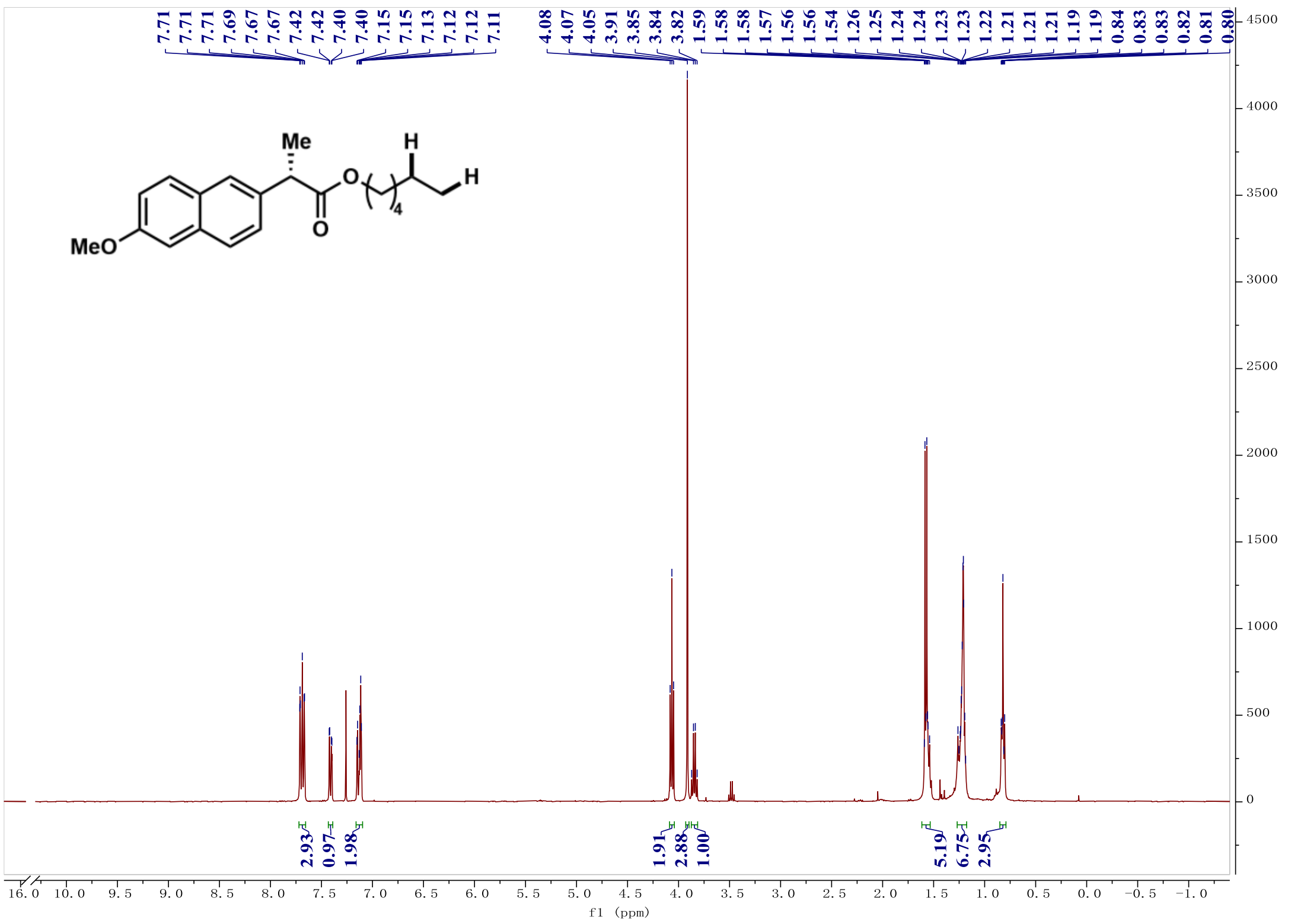

$^{1}$**H NMR** (400 MHz, $CDCl_3$) Spectrum of Compound **3aw**

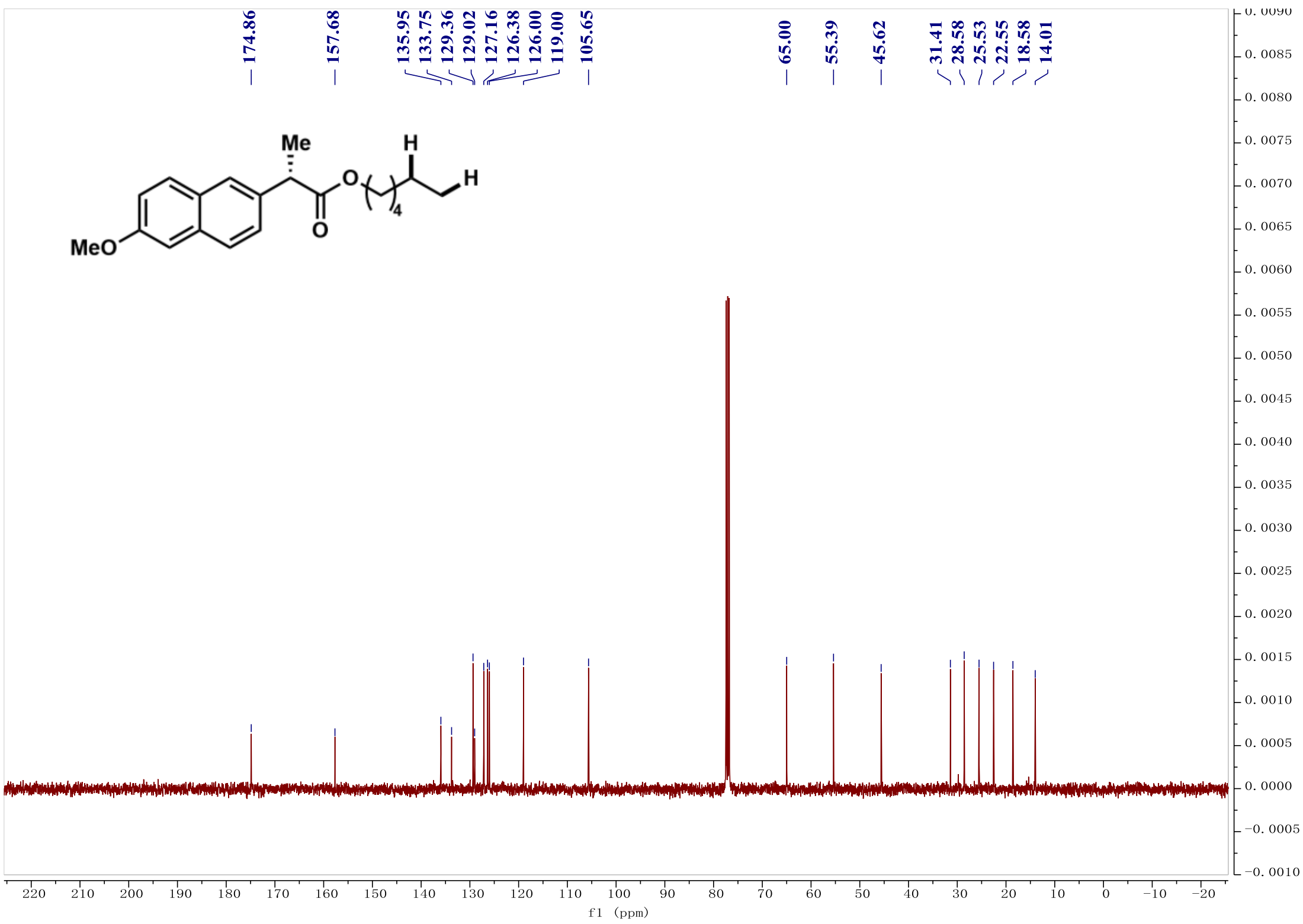

$^{13}$**C NMR** (101 MHz, $CDCl_3$) Spectrum of Compound **3aw**

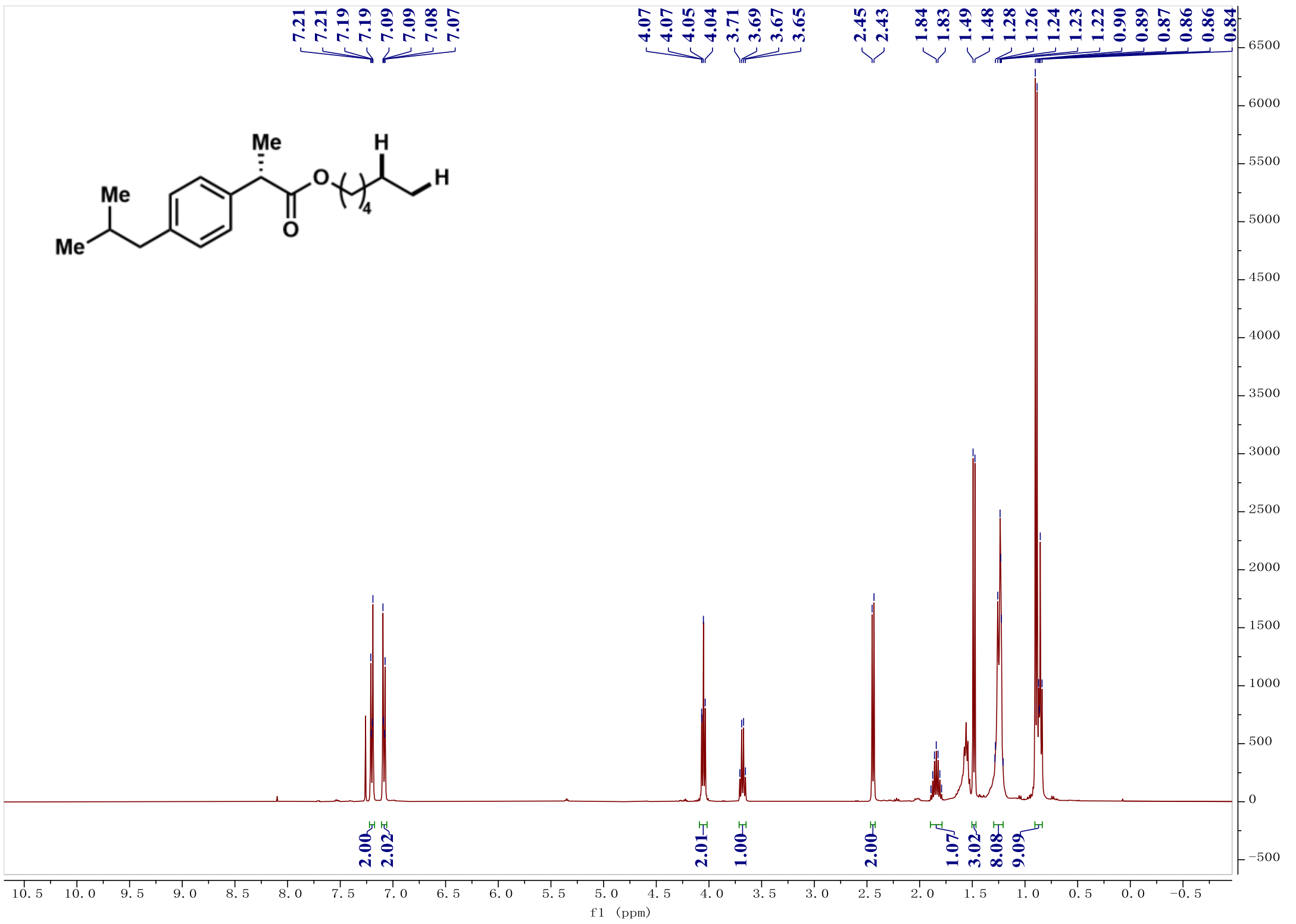


**$^{1}$H NMR** (400 MHz, $CDCl_3$) Spectrum of Compound **3ax**

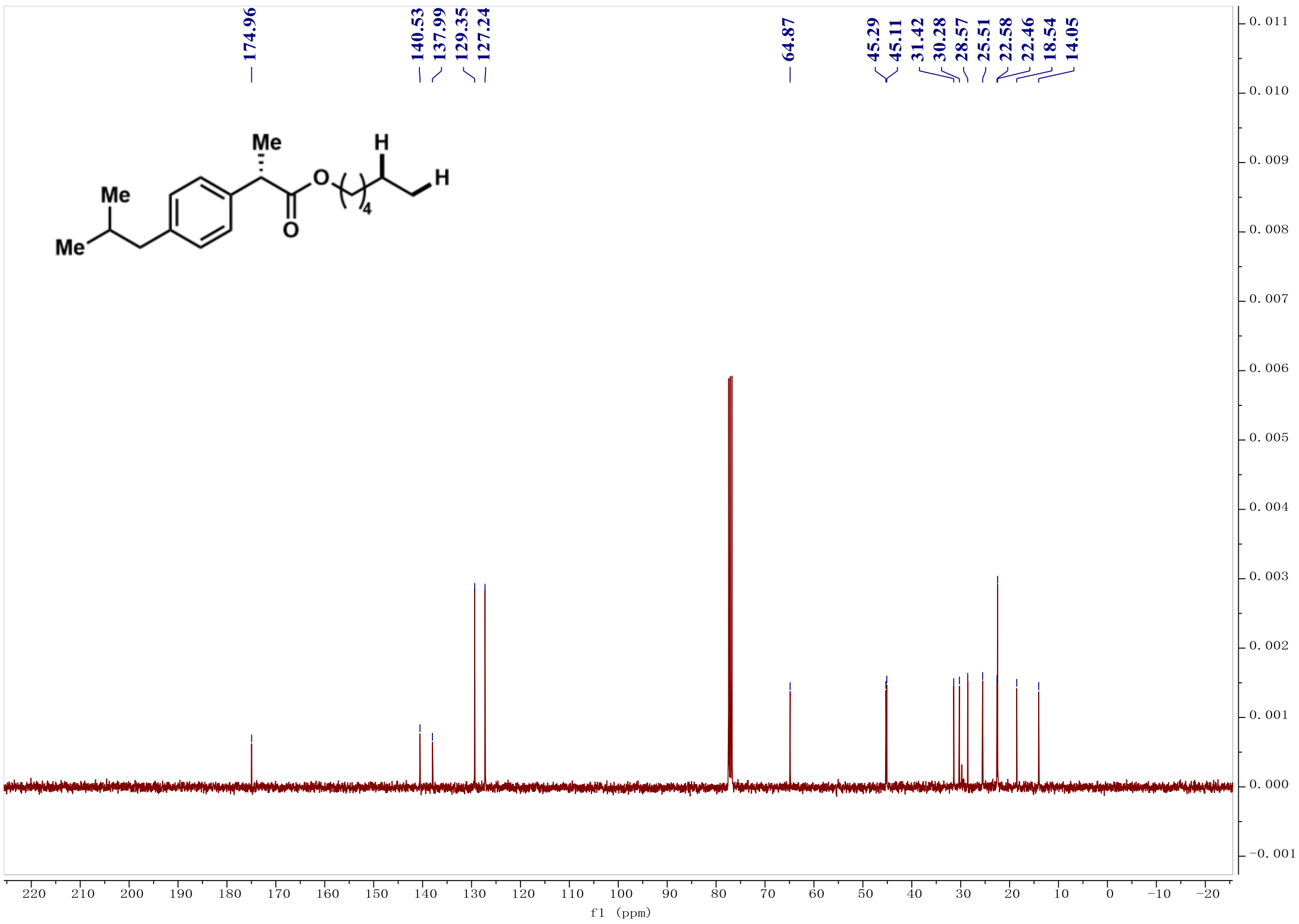


**$^{13}$C NMR** (101 MHz, $CDCl_3$) Spectrum of Compound **3ax**

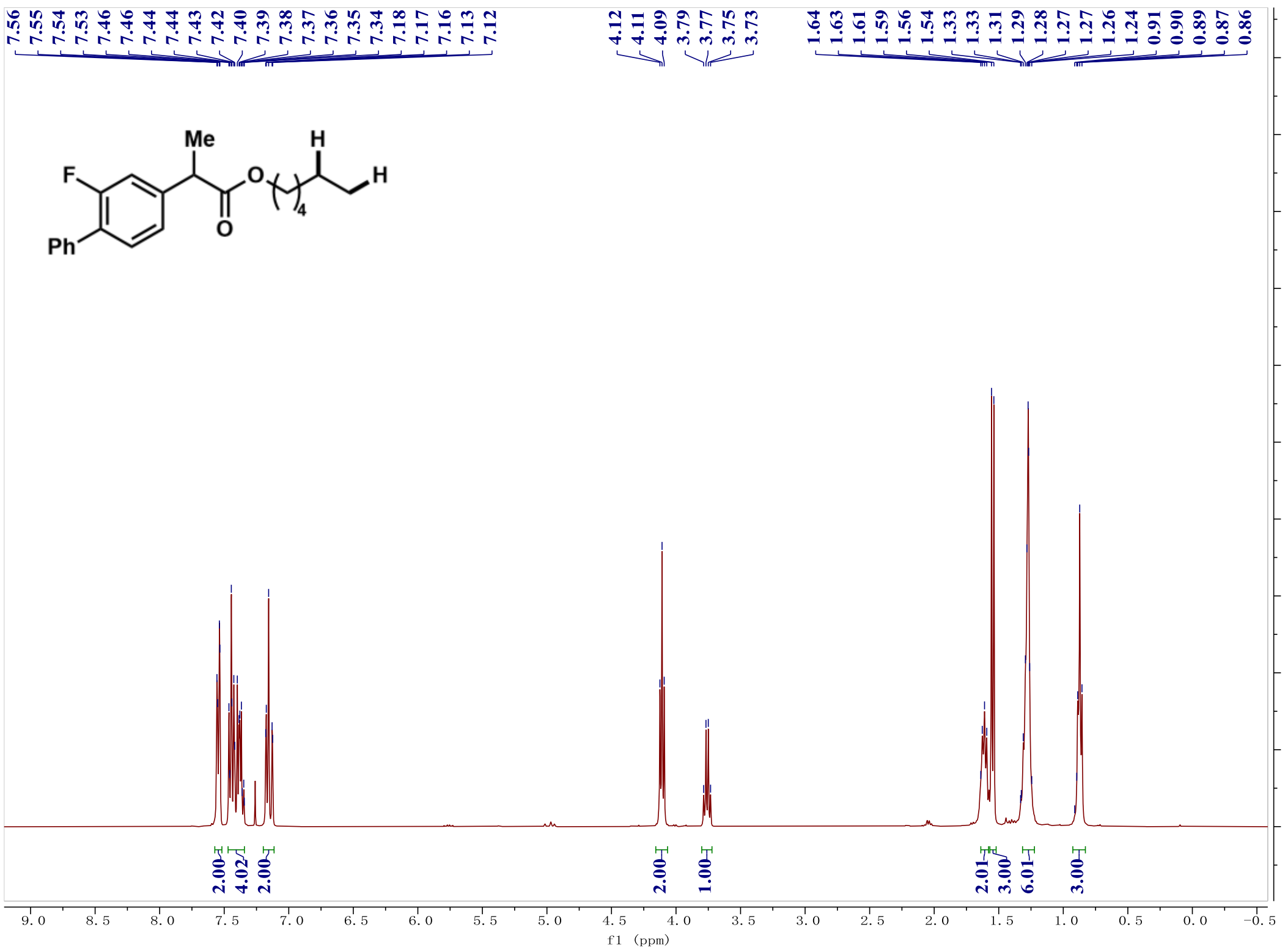

$^{1}$**H NMR** (400 MHz, $CDCl_3$) Spectrum of Compound **3ay**

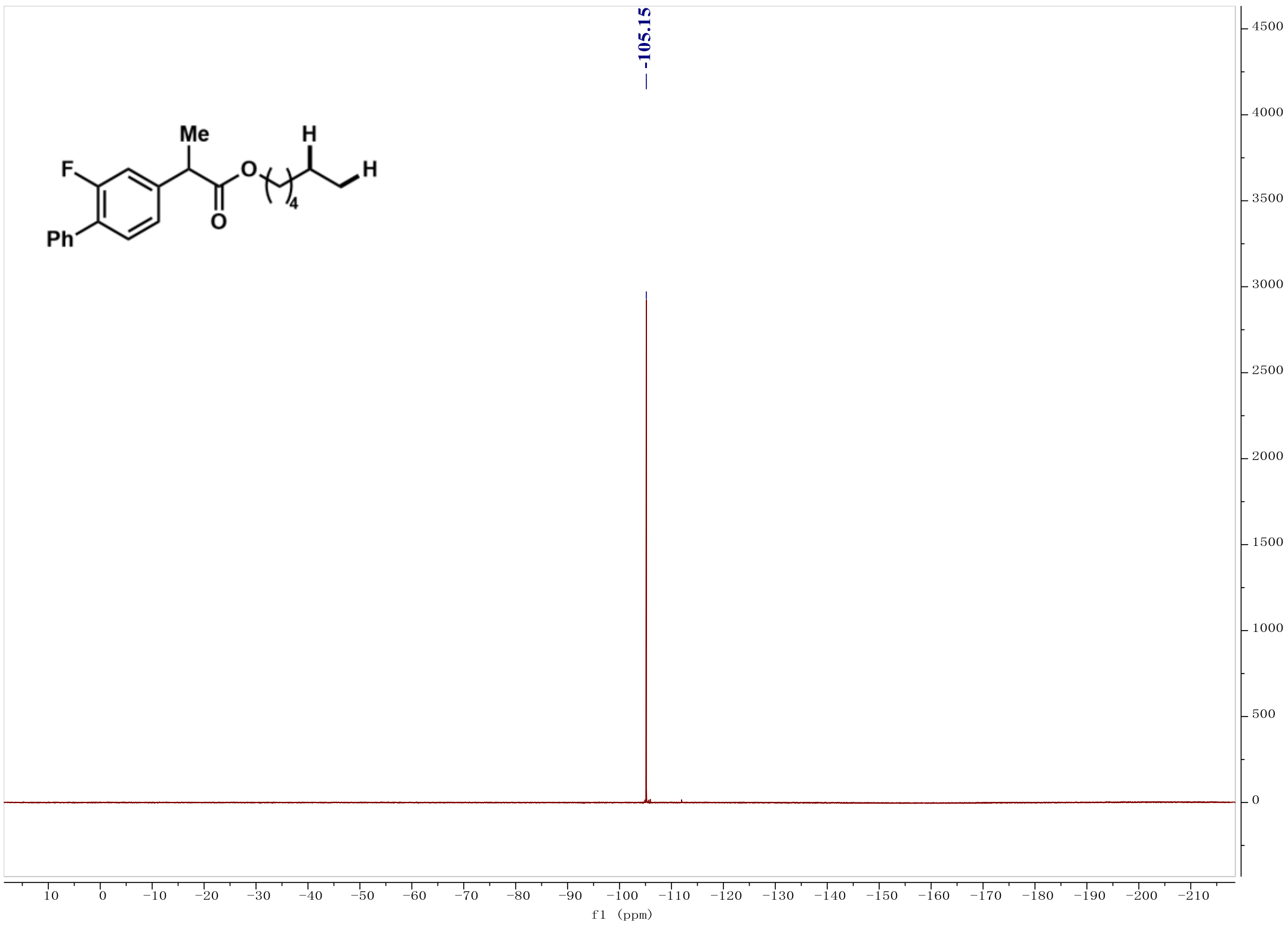

$^{19}$**F NMR** (376 MHz, CDCl3) Spectrum of Compound **3ay**

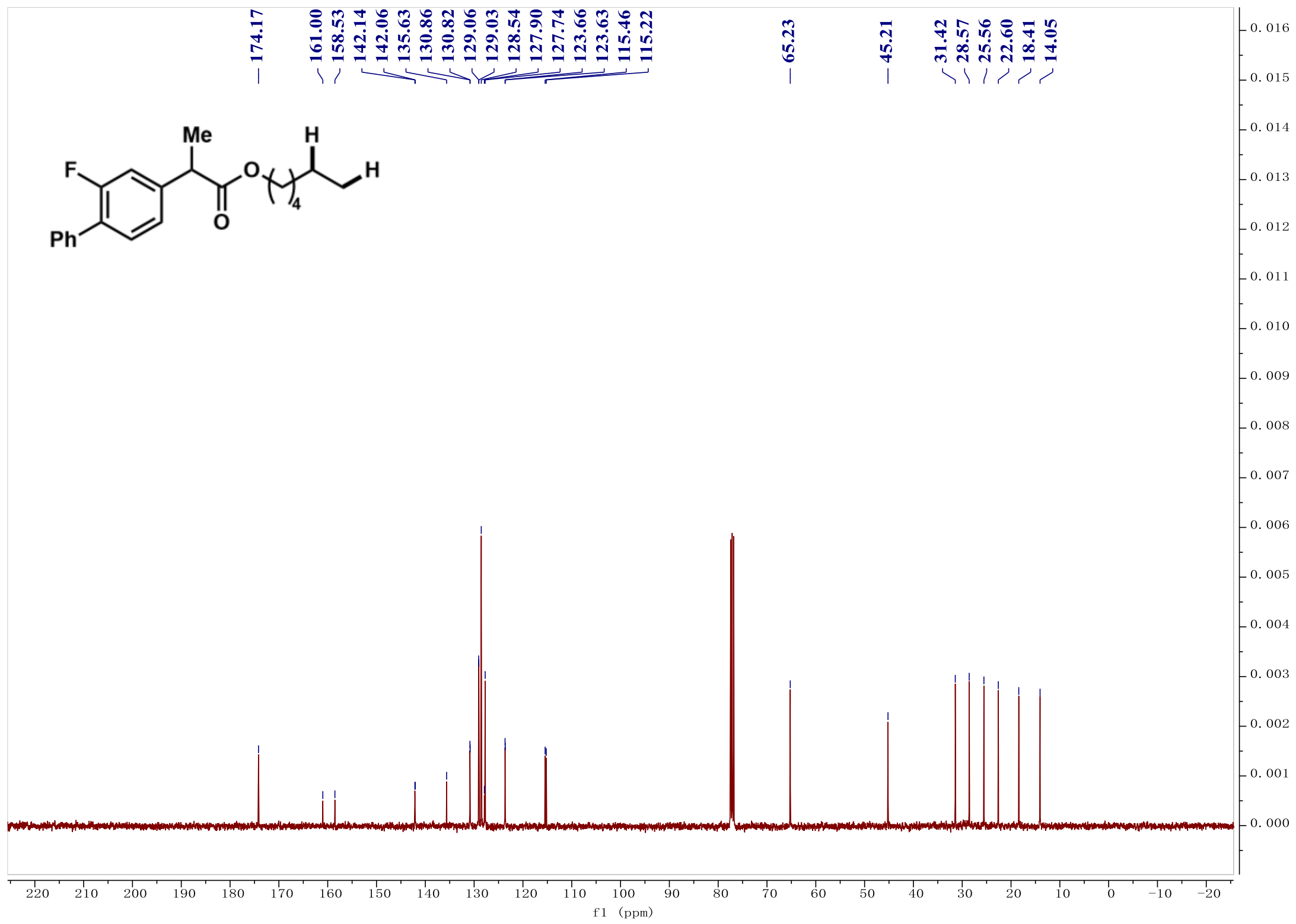


**$^{13}$C NMR** (101 MHz, $CDCl_3$) Spectrum of Compound **3ay**
